# Global, Regional, and National Burden of Nontraumatic Subarachnoid Hemorrhage: The Global Burden of Disease Study 2021

**DOI:** 10.1001/jamaneurol.2025.1522

**Published:** 2025-05-23

**Authors:** GBD 2021 Global Subarachnoid Hemorrhage Risk Factors Collaborators, Ilari Rautalin, Victor Volovici, Benjamin A. Stark, Catherine O. Johnson, Jaakko Kaprio, Miikka Korja, Rita V. Krishnamurthi, Balakrishnan Sukumaran Nair, Annemarei Ranta, Gabriel J. E. Rinkel, Mervyn D. I. Vergouwen, Yohannes Habtegiorgis Abate, Hedayat Abbastabar, Foad Abd-Allah, Atef Abdelkader, Parsa Abdi, Arash Abdollahi, Auwal Abdullahi, Olugbenga Olusola Abiodun, Richard Gyan Aboagye, Mohamed Abouzid, Dariush Abtahi, Samir Abu Rumeileh, Ahmed Abualhasan, Hasan Abualruz, Hana J. Abukhadijah, Ahmed Abu-Zaid, Lawan Hassan Adamu, Isaac Yeboah Addo, Rufus Adesoji Adedoyin, Oyelola A. Adegboye, Saryia Adra, Leticia Akua Adzigbli, Williams Agyemang-Duah, Bright Opoku Ahinkorah, Aqeel Ahmad, Danish Ahmad, Amir Mahmoud Ahmadzade, Ali Ahmed, Haroon Ahmed, Syed Anees Ahmed, Budi Aji, Mohammed Ahmed Akkaif, Yazan Al-Ajlouni, Ziyad Al-Aly, Mohammed Albashtawy, Mohammed Usman Ali, Sheikh Mohammad Alif, Yousef Alimohamadi, Syed Mohamed Aljunid, Mahmoud A. Alomari, Ahmad Alrawashdeh, Mohammed A. Alsabri, Rustam Al-Shahi Salman, Awais Altaf, Alaa B. Al-Tammemi, Nelson Alvis-Guzman, Hassan Alwafi, Mohammad Al-Wardat, Yaser Mohammed Al-Worafi, Hany Aly, Mohammad Sharif Ibrahim Alyahya, Karem H. Alzoubi, Reza Amani, Tarek Tawfik Amin, Alireza Amindarolzarbi, Ganiyu Adeniyi Amusa, Deanna Anderlini, Dhanalakshmi Angappan, Abhishek Anil, Boluwatife Stephen Anuoluwa, Saleha Anwar, Anayochukwu Edward Anyasodor, Geminn Louis Carace Apostol, Jalal Arabloo, Demelash Areda, Johan Ärnlöv, Anton A. Artamonov, Kurnia Dwi Artanti, Ashokan Arumugam, Zahra Aryan, Mohammad Asghari-Jafarabadi, Mubarek Yesse Ashemo, Tahira Ashraf, Mohammad Athar, Seyyed Shamsadin Athari, Avinash Aujayeb, Adedapo Wasiu Awotidebe, Sina Azadnajafabad, Shahkaar Aziz, Ahmed Y. Azzam, Giridhara Rathnaiah Babu, Nasser Bagheri, Pegah Bahrami Taghanaki, Saeed Bahramian, Ruhai Bai, Atif Amin Baig, Abdulaziz T. Bako, Ovidiu Constantin Baltatu, Kiran Bam, Maciej Banach, Soham Bandyopadhyay, Biswajit Banik, Mainak Bardhan, Suzanne Lyn Barker-Collo, Till Winfried Bärnighausen, Hiba Jawdat Barqawi, Lingkan Barua, Mohammad-Mahdi Bastan, Sanjay Basu, Shelly L. Bell, Isabela M. Bensenor, Alemshet Yirga Berhie, Kebede A. Beyene, Akshaya Srikanth Bhagavathula, Sonu Bhaskar, Ajay Nagesh Bhat, Vivek Bhat, Gurjit Kaur Bhatti, Jasvinder Singh Bhatti, Ali Bijani, Boris Bikbov, Mekuriaw Mesfin Birhan, Mulugeta M. Birhanu, Veera R. Bitra, Archith Boloor, Hamed Borhany, Susanne Breitner, Hermann Brenner, Raffaele Bugiardini, Norma B. Bulamu, Zahid A. Butt, Lucas Scotta Cabral, Florentino Luciano Caetano dos Santos, Daniela Calina, Luis Alberto Cámera, Luciana Aparecida Campos, Ismael Campos-Nonato, Angelo Capodici, Felix Carvalho, Carlos A. Castañeda-Orjuela, Alberico L. Catapano, Luca Cegolon, Joshua Chadwick, Chiranjib Chakraborty, Promit Ananyo Chakraborty, Sandip Chakraborty, Rama Mohan Chandika, Gashaw Sisay Chanie, Vijay Kumar Chattu, Anis Ahmad Chaudhary, Gerald Chi, Fatemeh Chichagi, Patrick R. Ching, Hitesh Chopra, Sonali Gajanan Choudhari, Enayet Karim Chowdhury, Dinh-Toi Chu, Sheng-Chia Chung, Alyssa Columbus, Michael H. Criqui, Alanna Gomes da Silva, Mohammad Amin Dabbagh Ohadi, Omid Dadras, Xiaochen Dai, Koustuv Dalal, Lachlan L. Dalli, Emanuele D’Amico, Mohsen Dashti, Kairat Davletov, Vanessa De la Cruz-Góngora, Shayom Debopadhaya, Ivan Delgado-Enciso, Emina Dervišević, Vinoth Gnana Chellaiyan Devanbu, Syed Masudur Rahman Dewan, Amol S. Dhane, Mahmoud Dibas, Thanh Chi Do, Thao Huynh Phuong Do, Sushil Dohare, Mohamed Fahmy Doheim, Klara Georgieva Dokova, Deepa Dongarwar, Mario D’Oria, Ojas Prakashbhai Doshi, Rajkumar Prakashbhai Doshi, Robert Kokou Dowou, Haneil Larson Dsouza, Siddhartha Dutta, Arkadiusz Marian Dziedzic, Abdel Rahman E’mar, David Edvardsson, Defi Efendi, Ferry Efendi, Nevine El Nahas, Islam Y. Elgendy, Muhammed Elhadi, Chadi Eltaha, Mohd. Elmagzoub Eltahir, Theophilus I. Emeto, Natalia Fabin, Adeniyi Francis Fagbamigbe, Ayesha Fahim, Ildar Ravisovich Fakhradiyev, Jawad Fares, Pawan Sirwan Faris, Nelsensius Klau Fauk, Timur Fazylov, Ginenus Fekadu, Nuno Ferreira, Getahun Fetensa, Florian Fischer, Matteo Foschi, Ni Kadek Yuni Fridayani, Abduzhappar Gaipov, Avi A. Gajjar, Aravind P. Gandhi, Balasankar Ganesan, Ravindra Kumar Garg, Miglas Welay Gebregergis, Mesfin Gebrehiwot, Teferi Gebru Gebremeskel, Molla Getie, Delaram J. Ghadimi, Fataneh Ghadirian, Sulmaz Ghahramani, Afsaneh Ghasemzadeh, Ramy Mohamed Ghazy, Maryam Gholamalizadeh, Sherief Ghozy, Artyom Urievich Gil, Jaleed Ahmed Gilani, Elena V. Gnedovskaya, Pouya Goleij, Alessandra C. Goulart, Barbara Niegia Garcia Goulart, Shi-Yang Guan, Sapna Gupta, Farrokh Habibzadeh, Mostafa Hadei, Najah R. Hadi, Samer Hamidi, Nasrin Hanifi, Graeme J. Hankey, Netanja I. Harlianto, Josep Maria Haro, Faizul Hasan, Hamidreza Hasani, Md Saquib Hasnain, Mahgol Sadat Hassan Zadeh Tabatabaei, Johannes Haubold, Rasmus J. Havmoeller, Simon I. Hay, Youssef Hbid, Golnaz Heidari, Mohammad Heidari, Mehdi Hemmati, Yuta Hiraike, Nguyen Quoc Hoan, Ramesh Holla, Mehdi Hosseinzadeh, Sorin Hostiuc, Junjie Huang, Hong-Han Huynh, Bing-Fang Hwang, Segun Emmanuel Ibitoye, Nayu Ikeda, Adalia Ikiroma, Mehran Ilaghi, Olayinka Stephen Ilesanmi, Irena M. Ilic, Milena D. Ilic, Md. Rabiul Islam, Nahlah Elkudssiah Ismail, Hiroyasu Iso, Gaetano Isola, Masao Iwagami, Louis Jacob, Abdollah Jafarzadeh, Akhil Jain, Ammar Abdulrahman Jairoun, Mihajlo Jakovljevic, Abubakar Ibrahim Jatau, Talha Jawaid, Sathish Kumar Jayapal, Jost B. Jonas, Nitin Joseph, Mikk Jürisson, Vidya Kadashetti, Rizwan Kalani, Vineet Kumar Kamal, Arun Kamireddy, Tanuj Kanchan, Himal Kandel, Jafar Karami, Ibraheem M. Karaye, Yeganeh Karimi, Arman Karimi Behnagh, Faizan Zaffar Kashoo, Gbenga A. Kayode, Foad Kazemi, Emmanuelle Kesse-Guyot, Yousef Saleh Khader, Inn Kynn Khaing, Fayaz Khan, Mohammad Jobair Khan, Haitham Khatatbeh, Moawiah Mohammad Khatatbeh, Hamid Reza Khayat Kashani, Khalid A. Kheirallah, Feriha Fatima Khidri, Moein Khormali, Atulya Aman Khosla, Kwanghyun Kim, Yun Jin Kim, Adnan Kisa, Sezer Kisa, Mika Kivimäki, Ali-Asghar Kolahi, Farzad Kompani, Oleksii Korzh, Karel Kostev, Nikhil Kothari, Kewal Krishan, Varun Krishna, Vijay Krishnamoorthy, Mohammed Kuddus, Mukhtar Kulimbet, Setor K. Kunutsor, Maria Dyah Kurniasari, Dian Kusuma, Ville Kytö, Carlo La Vecchia, Chandrakant Lahariya, Daphne Teck Ching Lai, Hanpeng Lai, Tri Laksono, Tea Lallukka, Kamaluddin Latief, Kaveh Latifinaibin, Nhi Huu Hanh Le, Thao Thi Thu Le, Munjae Lee, Seung Won Lee, Wei-Chen Lee, Yo Han Lee, Jacopo Lenzi, Matilde Leonardi, Ming-Chieh Li, Xiaopan Li, Stephen S. Lim, Jialing Lin, Xuefeng Liu, Valerie Lohner, László Lorenzovici, Paulo A. Lotufo, Giancarlo Lucchetti, Jay B. Lusk, Ricardo Lutzky Saute, Hawraz Ibrahim M. Amin, Armaan K. Malhotra, Kashish Malhotra, Ahmad Azam Malik, Deborah Carvalho Malta, Mohammad Ali Mansournia, Lorenzo Giovanni Mantovani, Emmanuel Manu, Hamid Reza Marateb, Mirko Marino, Seyed Farzad Maroufi, Ramon Martinez-Piedra, Santi Martini, Miquel Martorell, Roy Rillera Marzo, Yasith Mathangasinghe, Elezebeth Mathews, Andrea Maugeri, Steven M. McPhail, Asim Mehmood, Man Mohan Mehndiratta, Kamran Mehrabani-Zeinabad, Ritesh G. Menezes, Sultan Ayoub Meo, Atte Meretoja, Tomislav Mestrovic, Chamila Dinushi Kukulege Mettananda, Tomasz Miazgowski, Ana Carolina Micheletti Gomide Nogueira de Sá, Giuseppe Minervini, Le Huu Nhat Minh, Andreea Mirica, Erkin M. Mirrakhimov, Mohammad Mirza-Aghazadeh-Attari, Ajay Kumar Mishra, Prasanna Mithra, Abdalla Z. Mohamed, Ahmed Ismail Mohamed, Ameen Mosa Mohammad, Soheil Mohammadi, Abdollah Mohammadian-Hafshejani, Shafiu Mohammed, Ali H. Mokdad, Sabrina Molinaro, Shaher Momani, Mohammad Ali Moni, AmirAli Moodi Ghalibaf, Maryam Moradi, Yousef Moradi, Paula Moraga, Lidia Morawska, Ahmed Msherghi, Kavita Munjal, Christopher J. L. Murray, Ahamarshan Jayaraman Nagarajan, Ganesh R. Naik, Soroush Najdaghi, Noureddin Nakhostin Ansari, Shumaila Nargus, Delaram Narimani Davani, Zuhair S. Natto, Javaid Nauman, Nawsherwan, Vinod C. Nayak, Athare Nazri-Panjaki, Ruxandra Irina Negoi, Soroush Nematollahi, Charles Richard James Newton, Duc Hoang Nguyen, Hau Thi Hien Nguyen, Hien Quang Nguyen, Phat Tuan Nguyen, Van Thanh Nguyen, Robina Khan Niazi, Yeshambel T. Nigatu, Ali Nikoobar, Antonio Tolentino Nogueira de Sá, Shuhei Nomura, Jean Jacques Noubiap, Fred Nugen, Chimezie Igwegbe Nzoputam, Bogdan Oancea, Michael Safo Oduro, Tolulope R. Ojo-Akosile, Hassan Okati-Aliabad, Sylvester Reuben Okeke, Akinkunmi Paul Okekunle, Andrew T. Olagunju, Muideen Tunbosun Olaiya, Arão Belitardo Oliveira, Gláucia Maria Moraes Oliveira, Abdulhakeem Abayomi Olorukooba, Isaac Iyinoluwa Olufadewa, Raffaele Ornello, Esteban Ortiz-Prado, Uchechukwu Levi Osuagwu, Amel Ouyahia, Mayowa O. Owolabi, Ahmad Ozair, Mahesh Padukudru P A, Alicia Padron-Monedero, Jagadish Rao Padubidri, Demosthenes Panagiotakos, Georgios D. Panos, Leonidas D. Panos, Ioannis Pantazopoulos, Romil R. Parikh, Seoyeon Park, Jay Patel, Urvish K. Patel, Dimitrios Patoulias, Paolo Pedersini, Emmanuel K. Peprah, Gavin Pereira, Arokiasamy Perianayagam, Norberto Perico, Simone Perna, Fanny Emily Petermann-Rocha, Anil K. Philip, Michael A. Piradov, Evgenii Plotnikov, Roman V. Polibin, Maarten J. Postma, Jalandhar Pradhan, Manya Prasad, Jagadeesh Puvvula, Nameer Hashim Qasim, Gangzhen Qian, Alberto Raggi, Fakher Rahim, Vafa Rahimi-Movaghar, Mosiur Rahman, Muhammad Aziz Rahman, Amir Masoud Rahmani, Mohammad Rahmanian, Sathish Rajaa, Ali Rajabpour Sanati, Pushp Lata Rajpoot, Prashant Rajput, Mahmoud Mohammed Ramadan, Shakthi Kumaran Ramasamy, Sheena Ramazanu, Amey Rane, Sina Rashedi, Mohammad-Mahdi Rashidi, Devarajan Rathish, Salman Rawaf, Christian Razo, Murali Mohan Rama Krishna Reddy, Elrashdy Redwan, Giuseppe Remuzzi, Nazila Rezaei, Negar Rezaei, Mohsen Rezaeian, Hermano Alexandre Lima Rocha, Jefferson Antonio Buendia Rodriguez, Leonardo Roever, Michele Romoli, Marina Romozzi, Allen Guy Ross, Himanshu Sekhar Rout, Nitai Roy, Priyanka Roy, Aly M. A. Saad, Zahra Saadatian, Siamak Sabour, Simona Sacco, Basema Ahmad Saddik, Erfan Sadeghi, Usman Saeed, Fatemeh Saheb Sharif-Askari, Amirhossein Sahebkar, Pragyan Monalisa Sahoo, Md Refat Uz Zaman Sajib, Luciane B. Salaroli, Mohamed A. Saleh, Yoseph Leonardo Samodra, Vijaya Paul Samuel, Abdallah M. Samy, Milena M. Santric-Milicevic, Aswini Saravanan, Tanmay Sarkar, Gargi Sachin Sarode, Sachin C. Sarode, Benn Sartorius, Maheswar Satpathy, Markus P. Schlaich, Ione Jayce Ceola Schneider, Art Schuermans, Siddharthan Selvaraj, Subramanian Senthilkumaran, Sadaf G. Sepanlou, Yashendra Sethi, Allen Seylani, Ahmed Nabil Shaaban, Mahan Shafie, Moyad Jamal Shahwan, Masood Ali Shaikh, Summaiya Zareen Shaikh, Muhammad Aaqib Shamim, Anas Shamsi, Alfiya Shamsutdinova, Mohd Shanawaz, Mohammed Shannawaz, Amin Sharifan, Javad Sharifi Rad, Vishal Sharma, Bereket Beyene Shashamo, Mahabalesh Shetty, Premalatha K. Shetty, Mika Shigematsu, Aminu Shittu, Ivy Shiue, Nathan A. Shlobin, Seyed Afshin Shorofi, Emmanuel Edwar Siddig, Baljinder Singh, Paramdeep Singh, Puneetpal Singh, Surjit Singh, Farrukh Sobia, Ranjan Solanki, Shipra Solanki, Soroush Soraneh, Michael Spartalis, Suresh Kumar Srinivasamurthy, Jeffrey D. Stanaway, Muhammad Haroon Stanikzai, Antonina V. Starodubova, Jing Sun, Zhong Sun, Chandan Kumar Swain, Lukasz Szarpak, Payam Tabaee Damavandi, Seyyed Mohammad Tabatabaei, Seyed-Amir Tabatabaeizadeh, Celine Tabche, Jabeen Taiba, Iman M. Talaat, Jacques Lukenze Tamuzi, Ker-Kan Tan, Mohamad-Hani Temsah, Masayuki Teramoto, Ramna Thakur, Kavumpurathu Raman Thankappan, Rasiah Thayakaran, Sathish Thirunavukkarasu, Jansje Henny Vera Ticoalu, Krishna Tiwari, Marcello Tonelli, Roman Topor-Madry, Marcos Roberto Tovani-Palone, An Thien Tran, Jasmine T. Tran, Thang Huu Tran, Nguyen Tran Minh Duc, Thomas Clement Truelsen, Thien Tan Tri Tai Truyen, Daniel Hsiang-Te Tsai, Atta Ullah, Brigid Unim, Bhaskaran Unnikrishnan, Carolyn Anne Unsworth, Jibrin Sammani Usman, Sanaz Vahdati, Asokan Govindaraj Vaithinathan, Rohollah Valizadeh, Jef Van den Eynde, Joe Varghese, Tommi Juhani Vasankari, Narayanaswamy Venketasubramanian, Dominique Vervoort, Jorge Hugo Villafañe, Manish Vinayak, Sergey Konstantinovitch Vladimirov, Hatem A. Wafa, Yasir Waheed, Waseem Wahood, Mandaras Tariku Walde, Yanzhong Wang, Nuwan Darshana Wickramasinghe, Peter Willeit, Asrat Arja Wolde, Charles D. A. Wolfe, Yihun Miskir Wubie, Hong Xiao, Suowen Xu, Xiaoyue Xu, Kazumasa Yamagishi, Yuichiro Yano, Amir Yarahmadi, Habib Yaribeygi, Sanni Yaya, Pengpeng Ye, Dong Keon Yon, Naohiro Yonemoto, Chuanhua Yu, Aurora Zanghì, Iman Zare, Michael Zastrozhin, Chen Zhang, Yunquan Zhang, Zhi-Jiang Zhang, Zhiqiang Zhang, Hanqing Zhao, Shang Cheng Zhou, Abzal Zhumagaliuly, Hafsa Zia, Magdalena Zielińska, Samer H. Zyoud, Gregory A. Roth, Valery L. Feigin

**Affiliations:** 1 Department of Neurosurgery, Helsinki University Hospital, Helsinki, Finland; 2 The National Institute for Stroke and Applied Neurosciences, Auckland University of Technology, Auckland, New Zealand; 3 Department of Neurosurgery, Erasmus University Medical Center, Rotterdam, Netherlands; 4 Center for Experimental Microsurgery, Iuliu Hațieganu University of Medicine and Pharmacy, Cluj-Napoca, Romania; 5 Institute for Health Metrics and Evaluation, University of Washington, Seattle; 6 Institute for Molecular Medicine FIMM, University of Helsinki, Helsinki, Finland; 7 National Institute for Stroke and Applied Neurosciences, Auckland University of Technology, Auckland, New Zealand; 8 National Institute of Stroke and Applied Neurosciences, Auckland University of Technology, Auckland, New Zealand; 9 Department of Medicine, University of Otago, Wellington, New Zealand; 10 Department of Neurology, Capital & Coast District Health Board, Wellington, New Zealand; 11 Department of Neurology and Neurosurgery, University Medical Centre Utrecht, Utrecht University, Utrecht, Netherlands; 12 Department of Neurology and Neurosurgery, Utrecht University, Utrecht, Netherlands; 13 Department of Clinical Governance and Quality Improvement, Aleta Wondo General Hospital, Aleta Wondo, Ethiopia; 14 Advanced Diagnostic and Interventional Radiology Research Center, Tehran University of Medical Sciences, Tehran, Iran; 15 Department of Neurology, Cairo University, Cairo, Egypt; 16 Department of Mathematics and Sciences, Ajman University, Ajman, United Arab Emirates; 17 Department of Medicine, Memorial University, St John’s, Newfoundland and Labrador, Canada; 18 Minimally Invasive Surgery Research Center, Iran University of Medical Sciences, Tehran, Iran; 19 Department of Physiotherapy, Bayero University Kano, Kano, Nigeria; 20 Department of Physiotherapy, Federal University Wukari, Wukari, Nigeria; 21 Department of Internal Medicine, Federal Medical Centre, Abuja, Nigeria; 22 Department of Family and Community Health, University of Health and Allied Sciences, Ho, Ghana; 23 Department of Physical Pharmacy and Pharmacokinetics, Poznan University of Medical Sciences, Poznan, Poland; 24 Department of Anesthesiology, Shahid Beheshti University of Medical Sciences, Tehran, Iran; 25 Department of Neurology, Martin Luther University Halle-Wittenberg, Halle (Saale), Germany; 26 Department of Nursing, Al Zaytoonah University of Jordan, Amman, Jordan; 27 Medical Research Center, Hamad Medical Corporation, Doha, Qatar; 28 Department of Biochemistry and Molecular Medicine, Alfaisal University, Riyadh, Saudi Arabia; 29 College of Graduate Health Sciences, University of Tennessee, Memphis; 30 Department of Human Anatomy, Federal University Dutse, Dutse, Nigeria; 31 Department of Anatomy, Bayero University Kano, Kano, Nigeria; 32 School of Medicine, University of Sydney, Sydney, New South Wales, Australia; 33 Centre for Social Research in Health, University of New South Wales, Sydney, New South Wales, Australia; 34 Department of Medical Rehabilitation, Obafemi Awolowo University, Ile-Ife, Nigeria; 35 Menzies School of Health Research, Charles Darwin University, Darwin, Northern Territory, Australia; 36 Clinical Sciences Department, University of Sharjah, Sharjah, United Arab Emirates; 37 Department of Epidemiology and Biostatistics, University of Health and Allied Sciences, Ho, Ghana; 38 Department of Public Health Sciences, Queen’s University, Kingston, Ontario, Canada; 39 School of Public Health, University of Technology Sydney, Sydney, New South Wales, Australia; 40 Department of Medical Biochemistry, Shaqra University, Shaqra, Saudi Arabia; 41 School of Medicine and Psychology, Australian National University, Canberra, Australian Capital Territory, Australia; 42 Public Health Foundation of India, Gandhinagar, India; 43 Department of Neuroscience, Mashhad University of Medical Sciences, Mashhad, Iran; 44 Department of Pharmacy Practice, Riphah Institute of Pharmaceutical Sciences, Islamabad, Pakistan; 45 Division of Infectious Diseases and Global Public Health (IDGPH), University of California San Diego, San Diego, California; 46 Department of Biosciences, COMSATS Institute of Information Technology, Islamabad, Pakistan; 47 Brody School of Medicine, East Carolina University, Greenville, North Carolina; 48 Faculty of Medicine and Public Health, Jenderal Soedirman University, Purwokerto, Indonesia; 49 Department of Cardiology, Fudan University, Shanghai, China; 50 School of Medicine, New York Medical College, Valhalla; 51 Department of Epidemiology, Columbia University, New York, New York; 52 Department of Research and Development, Washington University in St Louis, St Louis, Missouri; 53 Clinical Epidemiology Center, US Department of Veterans Affairs (VA), St Louis, Missouri; 54 Department of Community and Mental Health, Al al-Bayt University, Mafraq, Jordan; 55 Department of Medical Rehabilitation (Physiotherapy), University of Maiduguri, Maiduguri, Nigeria; 56 Department of Rehabilitation Sciences, Hong Kong Polytechnic University, Hong Kong, China; 57 Institute of Health and Wellbeing, Federation University Australia, Melbourne, Victoria, Australia; 58 School of Public Health and Preventive Medicine, Monash University, Melbourne, Victoria, Australia; 59 Pars Advanced and Minimally Invasive Medical Manners Research Center, Iran University of Medical Sciences, Tehran, Iran; 60 Department of Public Health and Community Medicine, International Medical University, Kuala Lumpur, Malaysia; 61 International Centre for Casemix and Clinical Coding, National University of Malaysia, Bandar Tun Razak, Malaysia; 62 Department of Physical Therapy and Rehabilitation Sciences, Jordan University of Science and Technology, Irbid, Jordan; 63 Department of Rehabilitation Sciences and Physical Therapy, Jordan University of Science and Technology, Irbid, Jordan; 64 Department of Allied Medical Sciences, Jordan University of Science and Technology, Irbid, Jordan; 65 Department of Emergency Medicine, Sana’a University, Sanaa, Yemen; 66 Pediatric Emergency Medicine Department, St Christopher’s Hospital for Children, Philadelphia, Pennsylvania; 67 Centre for Clinical Brain Sciences, University of Edinburgh, Edinburgh, United Kingdom; 68 Institute of Molecular Biology and Biotechnology, The University of Lahore, Lahore, Pakistan; 69 Research, Policy, and Training Directorate, Jordan Center for Disease Control, Amman, Jordan; 70 Applied Science Research Center, Applied Science Private University, Amman, Jordan; 71 Research Group in Health Economics, Universidad de Cartagena, Cartagena, Colombia; 72 Research Group in Hospital Management and Health Policies, Universidad de la Costa (University of the Coast), Barranquilla, Colombia; 73 Department of Clinical Pharmacology and Toxicology, Umm Al-Qura University, Makkah, Saudi Arabia; 74 Department of Rehabilitation Sciences, Jordan University of Science and Technology, Irbid, Jordan; 75 Department of Medical Sciences, Azal University for Human Development, Sana’a, Yemen; 76 Department of Clinical Sciences, University of Science and Technology of Fujairah, Fujairah, United Arab Emirates; 77 Department of Pediatrics, Cleveland Clinic, Cleveland, Ohio; 78 Faculty of Medicine, Jordan University of Science and Technology, Irbid, Jordan; 79 Department of Pharmacy Practice and Pharmacotherapeutics, University of Sharjah, Sharjah, United Arab Emirates; 80 Department of Clinical Pharmacy, Jordan University of Science and Technology, Irbid, Jordan; 81 Interdisciplinary Graduate Program in Human Toxicology, University of Iowa, Iowa City; 82 Holden Comprehensive Cancer Center, University of Iowa Hospitals and Clinics, Iowa City, Iowa; 83 Public Health and Community Medicine Department, Cairo University, Cairo, Egypt; 84 Department of Radiology and Radiological Science, Johns Hopkins University, Baltimore, Maryland; 85 Department of Medicine, University of Jos, Jos, Nigeria; 86 Department of Internal Medicine, Jos University Teaching Hospital, Jos, Nigeria; 87 Centre for Sensorimotor Performance, The University of Queensland, Brisbane, Queensland, Australia; 88 Neurology Department, Royal Brisbane and Women’s Hospital, Brisbane, Queensland, Australia; 89 Department of Child Neurology, Oregon Health and Science University, Portland; 90 Department of Pharmacology, All India Institute of Medical Sciences, Jodhpur, India; 91 All India Institute of Medical Sciences, Bhubaneswar, India; 92 Department of Environmental and Occupational Health, University of Medical Sciences, Ondo, Ondo, Nigeria; 93 Centre for Interdisciplinary Research in Basic Sciences (CIRBSc), Jamia Millia Islamia, New Delhi, India; 94 School of Chemical and Life Sciences (SCLS), Jamia Hamdard, New Delhi, India; 95 Rural Health Research Institute, Charles Sturt University, Orange, New South Wales, Australia; 96 School of Medicine and Public Health, Ateneo De Manila University, Pasig City, Philippines; 97 Inter-Agency Committee on Environmental Health, Department of Health Philippines, Manila, Philippines; 98 Health Management and Economics Research Center, Iran University of Medical Sciences, Tehran, Iran; 99 College of Art and Science, Ottawa University, Surprise, Arizona; 100 School of Life Sciences, Arizona State University, Tempe; 101 Department of Neurobiology, Care Sciences and Society, Karolinska Institute, Stockholm, Sweden; 102 School of Health and Social Studies, Dalarna University, Falun, Sweden; 103 Institute for Biomedical Problems, Russian Academy of Sciences, Moscow, Russia; 104 Division of Epidemiology, Universitas Airlangga (Airlangga University), Surabaya, Indonesia; 105 Department of Physiotherapy, University of Sharjah, Sharjah, United Arab Emirates; 106 Department of Physiotherapy, Manipal Academy of Higher Education, Manipal, India; 107 Brigham and Women’s Hospital, Harvard University, Boston, Massachusetts; 108 Non-communicable Diseases Research Center, Tehran University of Medical Sciences, Tehran, Iran; 109 Cabrini Research, Cabrini Health, Malvern, Victoria, Australia; 110 School of Public Health and Preventative Medicine, Monash University, Melbourne, Victoria, Australia; 111 Department of Public Health, Jimma University, Jimma, Ethiopia; 112 Department of Public Health, Wachemo University, Hossana, Ethiopia; 113 University Institute of Radiological Sciences and Medical Imaging Technology, The University of Lahore, Lahore, Pakistan; 114 Department of Medical Genetics, Umm Al-Qura University, Makkah, Saudi Arabia; 115 Science and Technology Unit, Umm Al-Qura University, Makkah, Saudi Arabia; 116 Department of Immunology, Zanjan University of Medical Sciences, Zanjan, Iran; 117 Northumbria HealthCare NHS Foundation Trust, Newcastle upon Tyne, United Kingdom; 118 School of Nursing and Public Health, University of KwaZulu-Natal, Durban, South Africa; 119 Department of Surgery, Washington University in St Louis, St Louis, Missouri; 120 Leeds Institute of Rheumatic and Musculoskeletal Medicine, University of Leeds, Leeds, United Kingdom; 121 Institute of Biotechnology and Genetic Engineering, The University of Agriculture, Peshawar, Pakistan; 122 ASIDE Healthcare, Lewes, Delaware; 123 Faculty of Medicine, October 6 University, 6th of October City, Egypt; 124 Department of Population Medicine, Qatar University, Doha, Qatar; 125 Health Research Institute, University of Canberra, Canberra, Australian Capital Territory, Australia; 126 Department of Biostatistics, Mashhad University of Medical Sciences, Mashhad, Iran; 127 School of Medicine, Isfahan University of Medical Sciences, Isfahan, Iran; 128 School of Public Affairs, Nanjing University of Information Science and Technology, Nanjing, China; 129 International Medical School, Management and Science University, Alam, Malaysia; 130 Department of Neurosurgery, Houston Methodist Hospital, Houston, Texas; 131 College of Medicine, Alfaisal University, Riyadh, Saudi Arabia; 132 Center of Innovation, Technology and Education (CITE), Anhembi Morumbi University, São José dos Campos, Brazil; 133 Department of Medicine, Monash University, Clayton, Victoria, Australia; 134 Department of Hypertension, Medical University of Lodz, Lodz, Poland; 135 Polish Mothers’ Memorial Hospital Research Institute, Lodz, Poland; 136 Nuffield Department of Surgical Sciences, University of Oxford, Oxford, United Kingdom; 137 Department of Neurosurgery, University of Southampton, Southampton, United Kingdom; 138 Manna Institute, University of New England, Armidale, New South Wales, Australia; 139 Miller School of Medicine, University of Miami, Miami, Florida; 140 School of Psychology, University of Auckland, Auckland, New Zealand; 141 Heidelberg Institute of Global Health (HIGH), Heidelberg University, Heidelberg, Germany; 142 T.H. Chan School of Public Health, Harvard University, Boston, Massachusetts; 143 Department of Non-communicable Diseases, Bangladesh University of Health Sciences, Dhaka, Bangladesh; 144 School of Medicine, Iran University of Medical Sciences, Tehran, Iran; 145 Center for Primary Care, Harvard University, Boston, Massachusetts; 146 School of Public Health, Imperial College London, London, United Kingdom; 147 School of the Environment, Yale University, New Haven, Connecticut; 148 School of Health Policy and Management, Korea University, Seoul, South Korea; 149 Department of Internal Medicine, University of São Paulo, São Paulo, Brazil; 150 Department of Nursing, Bahir Dar University, Bahir Dar, Ethiopia; 151 Department of Pharmaceutical and Administrative Sciences, University of Health Sciences and Pharmacy in St Louis, St Louis, Missouri; 152 School of Pharmacy, University of Auckland, Auckland, New Zealand; 153 Department of Public Health, North Dakota State University, Fargo; 154 Division of Gastroenterology and Hepatology, Mayo Clinic, Jacksonville, Florida; 155 Global Health Neurology Lab, NSW Brain Clot Bank, Sydney, New South Wales, Australia; 156 Division of Cerebrovascular Medicine and Neurology, National Cerebral and Cardiovascular Center, Suita, Japan; 157 Department of General Medicine, Manipal Academy of Higher Education, Mangalore, India; 158 Department of Internal Medicine, St John’s National Academy of Health Sciences, Bangalore, India; 159 Department of Medical Lab Technology, Chandigarh University, Mohali, India; 160 Department of Human Genetics and Molecular Medicine, Central University of Punjab, Bathinda, India; 161 Social Determinants of Health Research Center, Babol University of Medical Sciences, Babol, Iran; 162 Scientific-Tools.Org, Bergamo, Italy; 163 Department of Biomedical Sciences, Bahir Dar University, Bahir Dar, Ethiopia; 164 Stroke and Ageing Research Group, Monash University, Clayton, Victoria, Australia; 165 Department of Nursing, St Paul’s Hospital Millennium Medical College, Addis Ababa, Ethiopia; 166 Faculty of Health Sciences, University of Botswana, Gaborone, Botswana; 167 Department of Internal Medicine, Manipal Academy of Higher Education, Mangalore, India; 168 Internal Medicine Department, Shahid Beheshti University of Medical Sciences, Tehran, Iran; 169 Institute for Medical Information Processing, Biometry, and Epidemiology, LMU Munich, Neuherberg, Germany; 170 Institute of Epidemiology, Helmholtz Zentrum München (German Research Center for Environmental Health), Neuherberg, Germany; 171 Division of Clinical Epidemiology and Aging Research, German Cancer Research Center, Heidelberg, Germany; 172 Department of Medical and Surgical Sciences, University of Bologna, Bologna, Italy; 173 Flinders Health and Medical Research Institute, Flinders University, Adelaide, South Australia, Australia; 174 School of Public Health Sciences, University of Waterloo, Waterloo, Ontario, Canada; 175 Al Shifa School of Public Health, Al Shifa Trust Eye Hospital, Rawalpindi, Pakistan; 176 Department of Neurology, Porto Alegre Clinical Hospital, Porto Alegre, Brazil; 177 Department of Interventional Neuroradiology, Hospital Moinhos de Vento, Porto Alegre, Brazil; 178 Harvard Business School, Harvard University, Boston, Massachusetts; 179 Department of Clinical Pharmacy, University of Medicine and Pharmacy of Craiova, Craiova, Romania; 180 Department of Internal Medicine, Hospital Italiano de Buenos Aires, Buenos Aires, Argentina; 181 Board of Directors, Argentine Society of Medicine, Buenos Aires, Argentina; 182 Institute of Biomedical Engineering, Anhembi Morumbi University, Sao Jose dos Campos, Brazil; 183 Department of Biomedical Engineering, Center of Innovation, Technology and Education (CITE) at São José dos Campos Technology Park, Sao Jose dos Campos, Brazil; 184 Center for Nutrition and Health Research, National Institute of Public Health, Cuernavaca, Mexico; 185 Department of Health Management (Direzione Sanitaria), IRCCS Istituto Ortopedico Rizzoli, Bologna, Italy; 186 Interdisciplinary Research Center for Health Science, Sant’Anna School of Advanced Studies, Pisa, Italy; 187 Research Unit on Applied Molecular Biosciences (UCIBIO), University of Porto, Porto, Portugal; 188 Colombian National Health Observatory, Instituto Nacional de Salud (National Institute of Health), Bogota, Colombia; 189 Epidemiology and Public Health Evaluation Group, National University of Colombia, Bogota, Colombia; 190 Department of Pharmacological and Biomolecular Sciences, University of Milan, Milan, Italy; 191 MultiMedica Sesto San Giovanni IRCCS, Sesto San Giovanni, Italy; 192 Department of Medical, Surgical, and Health Sciences, University of Trieste, Trieste, Italy; 193 Public Health Unit, University Health Agency Giuliano-Isontina (ASUGI), Trieste, Italy; 194 Non-communicable Diseases Division, National Institute of Epidemiology, Chennai, India; 195 Department of Biotechnology, Adamas University, Kolkata, India; 196 Institute for Skeletal Aging & Orthopedic Surgery, Hallym University, Chuncheon, South Korea; 197 School of Population and Public Health, University of British Columbia, Vancouver, British Columbia, Canada; 198 State Disease Investigation Laboratory, Animal Resources Development Department, Agartala, India; 199 Department of Clinical Nutrition, Jazan University, Jazan, Saudi Arabia; 200 Department of Clinical Pharmacy, University of Gondar, Gondar, Ethiopia; 201 Temerty Faculty of Medicine, University of Toronto, Toronto, Ontario, Canada; 202 Department of Community Medicine, Datta Meghe Institute of Medical Sciences, Sawangi, India; 203 Department of Biology, Al-Imam Mohammad Ibn Saud Islamic University, Riyadh, Saudi Arabia; 204 Division of Cardiovascular Medicine, Harvard University, Boston, Massachusetts; 205 Department of Scientific Research, Tehran University of Medical Sciences, Tehran, Iran; 206 Division of Infectious Diseases, Virginia Commonwealth University, Richmond; 207 Centre for Research Impact & Outcome, Chitkara University, Rajpura, India; 208 Department of Community Medicine, Jawaharlal Nehru Medical College, Wardha, India; 209 School of Public Health, Curtin University, Perth, Western Australia, Australia; 210 Department of Epidemiology and Preventative Medicine, Monash University, Melbourne, Victoria, Australia; 211 The Interdisciplinary Research Group on Biomedicine and Health, VNU International School (VNUIS), Hanoi, Vietnam; 212 Faculty of Applied Sciences, VNU International School (VNUIS), Hanoi, Vietnam; 213 Department of Health Informatics, University College London, London, United Kingdom; 214 Health Data Research UK, London, United Kingdom; 215 Department of Biostatistics, Johns Hopkins University, Baltimore, Maryland; 216 Department of Family Medicine and Public Health, University of California San Diego, La Jolla; 217 School of Nursing, Federal University of Minas Gerais, Belo Horizonte, Brazil; 218 Department of Neurosurgery, Tehran University of Medical Sciences, Tehran, Iran; 219 Department of Global Public Health and Primary Care, University of Bergen, Bergen, Norway; 220 Iranian Research Center for HIV/AIDS (IRCHA), Tehran University of Medical Sciences, Tehran, Iran; 221 Department of Health Metrics Sciences, School of Medicine, University of Washington, Seattle; 222 Institute for Health Sciences, Mid Sweden University, Sundsvall, Sweden; 223 Department of Medical and Surgical Sciences and Advanced Technologies “GF Ingrassia,” University of Catania, Catania, Italy; 224 Immunology Research Center, Tabriz University of Medical Sciences, Tabriz, Iran; 225 Health Research Institute, Kazakh National Medical University, Almaty, Kazakhstan; 226 Center for Evaluation and Surveys Research, National Institute of Public Health, Cuernavaca, Mexico; 227 Medical College, Albany Medical College, Albany, New York; 228 School of Medicine, University of Colima, Colima, Mexico; 229 Department of Research, State Cancerology Institute of Colima, IMSS-BIENESTAR, Colima, Mexico; 230 Department of Forensic Medicine, University of Sarajevo, Sarajevo, Bosnia and Herzegovina; 231 Chettinad Hospital & Research Institute, Chettinad Academy of Research and Education, Chennai, India; 232 Department of Pharmacy, United International University, Dhaka, Bangladesh; 233 Pharmacology Division, Center for Life Sciences Research Bangladesh, Dhaka, Bangladesh; 234 Research and Development Cell, Dr. D. Y. Patil Vidyapeeth, Pune (Deemed to be University), Pune, India; 235 Research Unit, Sulaiman Al Rajhi University, Qassim, Saudi Arabia; 236 Department of Medicine, Pham Ngoc Thach University of Medicine, Ho Chi Minh City, Vietnam; 237 Department of Medicine, Can Tho University of Medicine and Pharmacy, Can Tho, Vietnam; 238 Department of Epidemiology, Jazan University, Jazan, Saudi Arabia; 239 Neurology Department, Alexandria University, Alexandria, Egypt; 240 Department of Social Medicine and Health Care Organisation, Medical University of Varna, Varna, Bulgaria; 241 Health Science Center, University of Texas, Houston; 242 Cardio-Thoraco-Vascular Department, Azienda Sanitaria Universitaria Giuliano Isontina, Trieste, Italy; 243 Independent Consultant, South Plainfield, New Jersey; 244 Department of Cardiology, Hackettstown Medical Center, Hackettstown, New Jersey; 245 Newton Medical Center, Sparta, New Jersey; 246 Manipal Academy of Higher Education, Manipal, India; 247 Department of Forensic Medicine and Toxicology, Kasturba Medical College, Mangalore, Mangalore, India; 248 Department of Pharmacology, All India Institute of Medical Sciences, Rajkot, India; 249 Department of Conservative Dentistry with Endodontics, Medical University of Silesia, Katowice, Poland; 250 School of Nursing and Midwifery, La Trobe University, Melbourne, Victoria, Australia; 251 Department of Pediatric Nursing, University of Indonesia, Depok, Indonesia; 252 Neonatal Intensive Care Unit, University of Indonesia Hospital, Depok, Indonesia; 253 Advanced Nursing Department, Universitas Airlangga (Airlangga University), Surabaya, Indonesia; 254 Neurology Department, Ain Shams University, Cairo, Egypt; 255 Division of Cardiovascular Medicine, University of Kentucky, Lexington, Kentucky; 256 Division of Cardiology, Harvard University, Boston, Massachusetts; 257 Faculty of Medicine, University of Tripoli, Tripoli, Libya; 258 Houston Methodist Hospital, Houston, Texas; 259 Department of Pediatrics, University of Texas, Dallas; 260 Department of Education, Ajman University, Ajman, United Arab Emirates; 261 Department of Public Health and Tropical Medicine, James Cook University, Townsville, Queensland, Australia; 262 Independent Consultant, Bologna, Italy; 263 Department of Epidemiology and Medical Statistics, University of Ibadan, Ibadan, Nigeria; 264 Research Centre for Healthcare and Community, Coventry University, Coventry, United Kingdom; 265 Department of Oral Biology, Riphah International University, Islamabad, Pakistan; 266 Director of the Scientific and Technological Park, Kazakh National Medical University, Almaty, Kazakhstan; 267 Department of Neurological Surgery, Northwestern University, Chicago, Illinois; 268 Department of Biology, Salahaddin University-Erbil, Erbil, Iraq; 269 Department of Biology, Cihan University-Erbil, Erbil, Iraq; 270 Centre for Public Health, Equity and Human Flourishing, Torrens University Australia, Adelaide, South Australia, Australia; 271 Institute of Resource Governance and Social Change, Kupang, Indonesia; 272 Laboratory of Experimental Medicine, Kazakh National Medical University, Almaty, Kazakhstan; 273 Department of Infectious Diseases and Public Health, City University of Hong Kong, Hong Kong, China; 274 Department of Pharmacy, Wollega University, Nekemte, Ethiopia; 275 Department of Social Sciences, University of Nicosia, Nicosia, Cyprus; 276 Department of Nursing, Wollega University, Nekemte, Ethiopia; 277 Institute of Public Health, Charité Medical University Berlin, Berlin, Germany; 278 Department of Neuroscience, Multiple Sclerosis Research Center, Ravenna, Italy; 279 Department of Biotechnological and Applied Clinical Sciences, University of L’Aquila, L’Aquila, Italy; 280 Graduate Institute of Injury Prevention and Control, Taipei Medical University, Taipei, Taiwan; 281 Department of Medicine, Nazarbayev University, Astana, Kazakhstan; 282 Department of Neurosurgery, Hospital of the University of Pennsylvania, Penn Medicine, Philadelphia; 283 Neurosurgical Service, KK Women’s and Children’s Hospital, Singapore; 284 Department of Community Medicine and Family Medicine, All India Institute of Medical Sciences, Nagpur, India; 285 Institute of Health and Wellbeing, Federation University Australia, Churchill, Victoria, Australia; 286 Department of Neurology, King George’s Medical University, Lucknow, India; 287 Department of Midwifery, Adigrat University, Adigrat, Ethiopia; 288 Department of Environmental Health, Wollo University, Dessie, Ethiopia; 289 Department of Reproductive and Family Health, Axum College of Health Science, Axum, Ethiopia; 290 College of Medicine and Public Health, Flinders University, Adelaide, South Australia, Australia; 291 Department of Medical Laboratory Science, Addis Ababa University, Addis Ababa, Ethiopia; 292 School of Medicine, Shahid Beheshti University of Medical Sciences, Tehran, Iran; 293 Psychiatric Nursing and Management Department, Shahid Beheshti University of Medical Sciences, Tehran, Iran; 294 Health Policy Research Center, Shiraz University of Medical Sciences, Shiraz, Iran; 295 Tropical Health Department, Alexandria University, Alexandria, Egypt; 296 Family and Community Medicine Department, King Khalid University, Abha, Saudi Arabia; 297 Cancer Research Center, Shahid Beheshti University of Medical Sciences, Tehran, Iran; 298 Departments of Radiology and Neurosurgery, Mayo Clinic, Rochester, Minnesota; 299 Country Office, World Health Organization (WHO), Astana, Kazakhstan; 300 Department of Medicine, Aga Khan University, Karachi, Pakistan; 301 Third Department of Neurology, Research Center of Neurology, Moscow, Russia; 302 Department of Genetics, Sana Institute of Higher Education, Sari, Iran; 303 Universal Scientific Education and Research Network (USERN), Kermanshah University of Medical Sciences, Kermanshah, Iran; 304 Department of Epidemiology, University of São Paulo, São Paulo, Brazil; 305 Postgraduate Program in Epidemiology, Federal University of Rio Grande do Sul, Porto Alegre, Brazil; 306 Department of Epidemiology and Biostatistics, Anhui Medical University, Hefei, China; 307 Department of Toxicology, Shriram Institute for Industrial Research, Delhi, India; 308 Global Virus Network, Middle East Region, Shiraz, Iran; 309 Department of Health in Emergencies and Disasters, Tehran University of Medical Sciences, Tehran, Iran; 310 Department of Clinical Pharmacology and Medicine, University of Kufa, Najaf, Iraq; 311 School of Health and Environmental Studies, Hamdan Bin Mohammed Smart University, Dubai, United Arab Emirates; 312 Department of Critical Care and Emergency Nursing, Zanjan University of Medical Sciences, Zanjan, Iran; 313 Centre for Neuromuscular and Neurological Disorders, The University of Western Australia, Perth, Western Australia, Australia; 314 Stroke Research Centre, Perron Institute for Neurological and Translational Science, Perth, Western Australia, Australia; 315 Faculty of Medicine, Utrecht University, Utrecht, Netherlands; 316 Department of Radiology, University Medical Center Utrecht, Utrecht, Netherlands; 317 Research Unit, Parc Sanitari Sant Joan de Deu, Barcelona, Spain; 318 Department of Mental Health, Biomedical Research Networking Center for Mental Health Network (CiberSAM), Madrid, Spain; 319 Faculty of Nursing, Chulalongkorn University, Bangkok, Thailand; 320 Department of Ophthalmology, Iran University of Medical Sciences, Tehran, Iran; 321 Department of Pharmacy, Marwadi University, Rajkot, India; 322 Sina Trauma and Surgery Research Center, Tehran University of Medical Sciences, Tehran, Iran; 323 Department of Diagnostic and Interventional Radiology and Neuroradiology, University Hospital Essen, Essen, Germany; 324 Institute of Artificial Intelligence in Medicine, University Hospital Essen, Essen, Germany; 325 Skaane University Hospital, Skaane County Council, Malmö, Sweden; 326 UK Dementia Research Institute Care Research & Technology Centre, Imperial College London, London, United Kingdom; 327 Independent Consultant, Santa Clara, California; 328 Community-Oriented Nursing Midwifery Research Center, Shahrekord University of Medical Sciences, Shahrekord, Iran; 329 Department of Medicine, MedStar Health, Washington DC; 330 Department of Medicine, Georgetown University, Washington DC; 331 Graduate School of Medicine, University of Tokyo, Tokyo, Japan; 332 School of Dentistry, Hanoi Medical University, Hanoi, Vietnam; 333 Kasturba Medical College, Mangalore, Manipal Academy of Higher Education, Manipal, India; 334 School of Computer Science, Duy Tan University, Da Nang, Vietnam; 335 Mental Health Research Center, Iran University of Medical Sciences, Tehran, Iran; 336 Department of Legal Medicine and Bioethics, Carol Davila University of Medicine and Pharmacy, Bucharest, Romania; 337 Department of Clinical Legal Medicine, National Institute of Legal Medicine Mina Minovici, Bucharest, Romania; 338 Faculty of Medicine, The Chinese University of Hong Kong, Hong Kong, China; 339 International Master Program for Translational Science, Taipei Medical University, Taipei, Taiwan; 340 Department of Occupational Safety and Health, China Medical University, Taiwan, Taichung, Taiwan; 341 Department of Occupational Therapy, Asia University, Taiwan, Taichung, Taiwan; 342 Department of Health Promotion and Education, University of Ibadan, Ibadan, Nigeria; 343 International Center for Nutrition and Information, National Institutes of Biomedical Innovation, Health and Nutrition, Tokyo, Japan; 344 Collaborative Alliance Research and Education (CARE) Programme, Episcope Research Service, Aberdeen, Scotland; 345 Neurology Research Center, Kerman University of Medical Sciences, Kerman, Iran; 346 Kerman Neuroscience Research Center, Kerman University of Medical Sciences, Kerman, Iran; 347 West Africa RCC, Africa Centre for Disease Control and Prevention, Abuja, Nigeria; 348 Department of Community Medicine, University College Hospital, Ibadan, Ibadan, Nigeria; 349 Faculty of Medicine, University of Belgrade, Belgrade, Serbia; 350 Faculty of Medical Sciences, University of Kragujevac, Kragujevac, Serbia; 351 School of Pharmacy, BRAC University, Dhaka, Bangladesh; 352 Department of Clinical Pharmacy & Pharmacy Practice, Asian Institute of Medicine, Science and Technology, Bedong, Malaysia; 353 Malaysian Academy of Pharmacy, Puchong, Malaysia; 354 Public Health Department of Social Medicine, Osaka University, Suita, Japan; 355 Department of General Surgery and Medical-Surgical Specialties, University of Catania, Catania, Italy; 356 Department of Health Services Research, University of Tsukuba, Tsukuba, Japan; 357 Department of Non-Communicable Disease Epidemiology, London School of Hygiene & Tropical Medicine, London, United Kingdom; 358 Department of Physical and Medicine, Université Paris Cité, Paris, France; 359 Research and Development Unit, Biomedical Research Networking Center for Mental Health Network (CiberSAM), Barcelona, Spain; 360 Department of Immunology, Kerman University of Medical Sciences, Kerman, Iran; 361 Department of Immunology, Rafsanjan University of Medical Sciences, Rafsanjan, Iran; 362 Department of Leukemia, The University of MD Anderson Cancer Center, Houston, Texas; 363 Department of Health and Safety, Dubai Municipality, Dubai, United Arab Emirates; 364 The World Academy of Sciences UNESCO, Trieste, Italy; 365 Shaanxi University of Technology, Hanzhong, China; 366 School of Pharmacy and Pharmacology, University of Tasmania, Hobart, TAS, Australia; 367 Department of Pharmacology, Imam Mohammad Ibn Saud Islamic University, Riyadh, Saudi Arabia; 368 Centre of Studies and Research, Ministry of Health, Muscat, Oman; 369 Rothschild Foundation Hospital, Institute of Molecular and Clinical Ophthalmology Basel, Paris, France; 370 Singapore Eye Research Institute, Singapore, Singapore; 371 Department of Community Medicine, Manipal Academy of Higher Education, Mangalore, India; 372 Institute of Family Medicine and Public Health, University of Tartu, Tartu, Estonia; 373 Department of Oral and Maxillofacial Pathology, Krishna Vishwa Vidyapeeth (Deemed to be University), Karad, India; 374 Department of Neurology, University of Washington, Seattle, Washington; 375 Division of Epidemiology and Biostatistics, National Institute of Epidemiology, Chennai, India; 376 Department of Biostatistics, Indian Council of Medical Research, New Delhi, India; 377 Russell H. Morgan Department of Radiology and Radiological Science, Johns Hopkins University, Baltimore, Maryland; 378 Department of Forensic Medicine and Toxicology, All India Institute of Medical Sciences, Jodhpur, India; 379 Save Sight Institute, University of Sydney, Sydney, New South Wales, Australia; 380 Sydney Eye Hospital, South Eastern Sydney Local Health District, Sydney, New South Wales, Australia; 381 Laboratory Science Department, Khomein University of Medical Sciences, Khomein, Iran; 382 Department of Immunology, Tehran University of Medical Sciences, Tehran, Iran; 383 School of Health Professions and Human Services, Hofstra University, Hempstead, New York; 384 Department of Anesthesiology, Montefiore Medical Center, Bronx, New York; 385 Cardiovascular Diseases Research Institute, Tehran University of Medical Sciences, Tehran, Iran; 386 Endocrine Research Center, Iran University of Medical Sciences, Tehran, Iran; 387 Department of Echocardiography, Iran University of Medical Sciences, Tehran, Iran; 388 Department of Physical Therapy and Health Rehabilitation, Majmaah University, Majmaah, Saudi Arabia; 389 International Research Center of Excellence, Institute of Human Virology Nigeria, Abuja, Nigeria; 390 Julius Centre for Health Sciences and Primary Care, Utrecht University, Utrecht, Netherlands; 391 Department of Neurosurgery, Johns Hopkins University, Baltimore, Maryland; 392 Department of Human Nutrition, National Research Institute for Agriculture, Food and Environment, Jouy-en-Josas, France; 393 Sorbonne Paris Nord University, Bobigny, France; 394 Department of Public Health, Jordan University of Science and Technology, Irbid, Jordan; 395 Department of Public Health and Health Policy, Hiroshima University, Hiroshima, Japan; 396 Department of Physical Therapy, King Abdulaziz University, Jeddah, Saudi Arabia; 397 Faculty of Nursing, Yarmouk University, Irbid, Jordan; 398 Department of Basic Medical Sciences, Yarmouk University, Irbid, Jordan; 399 Department of Neurosurgery, Shahid Beheshti University of Medical Sciences, Tehran, Iran; 400 Department of Biochemistry, Liaquat University of Medical and Health Sciences, Jamshoro, Pakistan; 401 Department of Internal Medicine, Corewell Health East William Beaumont University Hospital, Royal Oak, Michigan; 402 Department of Medical Oncology, Miami Cancer Institute, Miami, Florida; 403 Graduate School of Public Health, Yonsei University, Busan, South Korea; 404 School of Traditional Chinese Medicine, Xiamen University Malaysia, Sepang, Malaysia; 405 School of Health Sciences, Kristiania University College, Oslo, Norway; 406 Department of International Health and Sustainable Development, Tulane University, New Orleans, Louisiana; 407 Department of Nursing and Health Promotion, Oslo Metropolitan University, Oslo, Norway; 408 Brain Sciences, University College London, London, United Kingdom; 409 Department of Public Health, University of Helsinki, Helsinki, Finland; 410 Social Determinants of Health Research Center, Shahid Beheshti University of Medical Sciences, Tehran, Iran; 411 Children’s Medical Center, Tehran University of Medical Sciences, Tehran, Iran; 412 Department of General Practice and Family Medicine, Kharkiv National Medical University, Kharkiv, Ukraine; 413 Department of Epidemiology, IQVIA, Frankfurt am Main, Germany; 414 University Hospital Marburg, Marburg, Germany; 415 Department of Anaesthesiology and Critical Care, All India Institute of Medical Sciences, Jodhpur, India; 416 Department of Anthropology, Panjab University, Chandigarh, India; 417 Department of Forensic Medicine and Toxicology, Pondicherry University, Puducherry, India; 418 Department of Anesthesiology, Duke University, Durham, North Carolina; 419 Department of Anesthesiology & Pain Medicine, University of Washington, Seattle; 420 Department of Biochemistry, University of Hail, Hail, Saudi Arabia; 421 Atchabarov Scientific-Research Institute of Fundamental and Applied Medicine, Kazakh National Medical University, Almaty, Kazakhstan; 422 Center of Medicine and Public Health, Asfendiyarov Kazakh National Medical University, Almaty, Kazakhstan; 423 Section of Cardiology, University of Manitoba, Winnipeg, Manitoba, Canada; 424 Translational Health Sciences, University of Bristol, Bristol, United Kingdom; 425 Faculty of Medicine and Health Science, Universitas Kristen Satya Wacana, Salatiga, Indonesia; 426 Nursing School, Taipei Medical University, Taipei, Taiwan; 427 Department of Health Services Research and Management, City University of London, London, United Kingdom; 428 Faculty of Public Health, University of Indonesia, Depok, Indonesia; 429 Clinical Research Center, Turku University Hospital, Turku, Finland; 430 Heart Center, University of Turku, Turku, Finland; 431 Department of Clinical Sciences and Community Health, University of Milan, Milan, Italy; 432 Integrated Department of Epidemiology, Health Policy, Preventive Medicine and Pediatrics, Foundation for People-centric Health Systems, New Delhi, India; 433 Centre for Health: The Specialty Practice, New Delhi, India; 434 School of Digital Science, Universiti Brunei Darussalam (University of Brunei Darussalam), Bandar Seri Begawan, Brunei; 435 Institute of Applied Data Analytics, Universiti Brunei Darussalam (University of Brunei Darussalam), Bandar Seri Begawan, Brunei; 436 Department of Occupational and Environmental Health, Yangzhou University, Yangzhou, China; 437 Department of Respiratory and Critical Care Medicine, Northern Jiangsu People’s Hospital, Yangzhou, China; 438 Department of Physiotherapy, Universitas Aisyiyah Yogyakarta, Yogyakarta, Indonesia; 439 Institute of Allied Health Sciences, National Cheng Kung University, Tainan, Taiwan; 440 Centre for Family Welfare, University of Indonesia, Depok, Indonesia; 441 Department of Global Health and Health Security, Taipei Medical University, Taipei, Taiwan; 442 Department of Anesthesiology, Iran University of Medical Sciences, Tehran, Iran; 443 Faculty of Medicine, University of Medicine and Pharmacy at Ho Chi Minh City, Ho Chi Minh City, Vietnam; 444 Department of Cardiovascular Research, Methodist Hospital, Merrillville, Indiana; 445 University of Medicine and Pharmacy at Ho Chi Minh City, Ho Chi Minh City, Vietnam; 446 Department of Medical Science, Ajou University School of Medicine, Suwon, South Korea; 447 Department of Precision Medicine, Sungkyunkwan University, Suwon-si, South Korea; 448 Department of Family Medicine, University of Texas Medical Branch, Galveston; 449 Department of Preventive Medicine, Korea University, Seoul, South Korea; 450 Department of Biomedical and Neuromotor Sciences, University of Bologna, Bologna, Italy; 451 UO Neurologia, Salute Pubblica e Disabilità (The Neurology, Public Health and Disability Unit), Fondazione IRCCS Istituto Neurologico Carlo Besta (IRCCS Foundation Carlo Besta Neurological Institute), Milan, Italy; 452 Department of Health Promotion and Health Education, National Taiwan Normal University, Taipei, Taiwan; 453 Department of Health Management Center, Fudan University, Shanghai, China; 454 International Centre for Future Health Systems, University of New South Wales, Sydney, New South Wales, Australia; 455 Lerner Research Institute, Cleveland Clinic, Cleveland, Ohio; 456 Department of Quantitative Health Science, Case Western Reserve University, Cleveland, Ohio; 457 Department of Cardiology, University of Cologne, Cologne, Germany; 458 Department of Health Economics, Syreon Research Romania, Targu Mures, Romania; 459 Department of Doctoral Studies, George Emil Palade University of Medicine, Pharmacy, Science, and Technology of Targu Mures, Targu Mures, Romania; 460 Department of Medicine, University of São Paulo, São Paulo, Brazil; 461 School of Medicine, Federal University of Juiz de Fora, Juiz de Fora, Brazil; 462 Department of Population Health Sciences, Duke University, Durham, North Carolina; 463 Department of Neurosciences and Behavioral Sciences, University of São Paulo, Ribeirão Preto, Brazil; 464 Department of Chemistry, Salahaddin University-Erbil, Erbil, Iraq; 465 Department of Medical Biochemical Analysis, Cihan University-Erbil, Erbil, Iraq; 466 Department of Neurosurgery, University of Toronto, Toronto, Ontario, Canada; 467 Rama Medical College Hospital and Research Centre, Uttar Pradesh, India; 468 Institute of Applied Health Research, University of Birmingham, Birmingham, United Kingdom; 469 Rabigh Faculty of Medicine, King Abdulaziz University, Jeddah, Saudi Arabia; 470 Department of Maternal-Child Nursing and Public Health, Federal University of Minas Gerais, Belo Horizonte, Brazil; 471 Department of Epidemiology and Biostatistics, Tehran University of Medical Sciences, Tehran, Iran; 472 School of Medicine and Surgery, University of Milan Bicocca, Monza, Italy; 473 Laboratory of Public Health, Instituto Auxologico Italiano IRCCS (Italian Auxological Institute), Milan, Italy; 474 Department of Population and Behavioural Sciences, University of Health and Allied Sciences, Ho, Ghana; 475 Department of Biomedical Engineering, University of Isfahan, Isfahan, Iran; 476 Biomedical Engineering Research Center (CREB), Universitat Politècnica de Catalunya (Barcelona Tech - UPC), Barcelona, Spain; 477 Department of Food, Environmental and Nutritional Sciences (DeFENS), University of Milan, Milano, Italy; 478 Faculty of Medicine, Tehran University of Medical Sciences, Tehran, Iran; 479 Non-Communicable Disease Research Center, Tehran University of Medical Sciences, Tehran, Iran; 480 Department of Non-communicable Diseases and Mental Health, Pan American Health Organization, Washington, DC; 481 Faculty of Public Health, Universitas Airlangga (University of Airlangga), Surabaya, Indonesia; 482 Indonesian Public Health Association, Surabaya, Indonesia; 483 Department of Nutrition and Dietetics, University of Concepción, Concepción, Chile; 484 Centre for Healthy Living, University of Concepción, Concepción, Chile; 485 Faculty of Humanities and Health Sciences, Curtin University, Sarawak, Malaysia; 486 Jeffrey Cheah School of Medicine and Health Sciences, Monash University, Subang Jaya, Malaysia; 487 Department of Anatomy and Developmental Biology, Monash University, Clayton, Victoria, Australia; 488 Department of Anatomy, Genetics and Biomedical Informatics, University of Colombo, Colombo, Sri Lanka; 489 Department of Public Health and Community Medicine, Central University of Kerala, Kasaragod, India; 490 Australian Centre for Health Services Innovation, Queensland University of Technology, Kelvin Grove, Queensland, Australia; 491 Digital Health and Informatics Directorate, Queensland Health, Brisbane, Queensland, Australia; 492 Department of Public Health, Jazan University, Jazan, Saudi Arabia; 493 Neurology Department, Janakpuri Super Specialty Hospital Society, New Delhi, India; 494 Department of Neurology, Govind Ballabh Institute of Medical Education and Research, New Delhi, India; 495 Department of Epidemiology and Biostatistics, Isfahan University of Medical Sciences, Isfahan, Iran; 496 Division of Forensic Medicine, Imam Abdulrahman Bin Faisal University, Dammam, Saudi Arabia; 497 Department of Physiology, King Saud University, Riyadh, Saudi Arabia; 498 General Administration Department, Helsinki University Hospital, Helsinki, Finland; 499 School of Health Sciences, University of Melbourne, Melbourne, Victoria, Australia; 500 University Centre Varazdin, University North, Varazdin, Croatia; 501 Department of Pharmacology, University of Kelaniya, Ragama, Sri Lanka; 502 Clinical Medicine Department, Colombo North Teaching Hospital, Ragama, Sri Lanka; 503 Department of Propedeutics of Internal Diseases & Arterial Hypertension, Pomeranian Medical University, Szczecin, Poland; 504 Multidisciplinary Department of Medical-Surgical and Dental Specialties, Univerity of Campania “Luigi Vanvitelli,” Naples, Italy; 505 Saveetha Dental College and Hospitals, Saveetha University, Chennai, India; 506 International Ph.D. Program in Medicine, Taipei Medical University, Taipei, Taiwan; 507 Research Center for Artificial Intelligence in Medicine, Taipei Medical University, Taipei, Taiwan; 508 Department of Statistics and Econometrics, Bucharest University of Economic Studies, Bucharest, Romania; 509 Internal Medicine Programme, Kyrgyz State Medical Academy, Bishkek, Kyrgyzstan; 510 Department of Atherosclerosis and Coronary Heart Disease, National Center of Cardiology and Internal Disease, Bishkek, Kyrgyzstan; 511 Department of Radiology, Tabriz University of Medical Sciences, Tabriz, Iran; 512 Social Determinants of Health Center, Urmia University of Medical Sciences, Urmia, Iran; 513 Division of Cardiology, St Vincent College, Worcester, Massachusetts; 514 Center for Brain and Health, New York University Abu Dhabi, Abu Dhabi, United Arab Emirates; 515 College of Health Science, University of Hargeisa, Hargeisa, Somalia; 516 Institute of Health Science, Jimma University, Jimma, Ethiopia; 517 College of Medicine, University of Duhok, Duhok, Iraq; 518 School of Medicine, Tehran University of Medical Sciences, Tehran, Iran; 519 Modeling in Health Research Center, Shahrekord University of Medical Sciences, Shahrekord, Iran; 520 Health Systems and Policy Research Unit, Ahmadu Bello University, Zaria, Nigeria; 521 Institute of Clinical Physiology, National Research Council, Pisa, Italy; 522 Department of Mathematics, The University of Jordan, Amman, Jordan; 523 Nonlinear Dynamics Research Center (NDRC), Ajman University, Ajman, United Arab Emirates; 524 AI & Cyber Futures Institute, Charles Sturt University, Bathurst, New South Wales, Australia; 525 The University of Queensland, Brisbane, Queensland, Australia; 526 Faculty of Medicine, Birjand University of Medical Sciences, Birjand, Iran; 527 Iran University of Medical Sciences, Tehran, Iran; 528 Department of Epidemiology and Biostatistics, Kurdistan University of Medical Sciences, Sanandaj, Iran; 529 Computer, Electrical, and Mathematical Sciences and Engineering Division, King Abdullah University of Science and Technology, Thuwal, Saudi Arabia; 530 International Laboratory for Air Quality and Health, Queensland University of Technology, Brisbane, Queensland, Australia; 531 Department of Radiology, University of Tripoli, Tripoli, Libya; 532 Amity Institute of Pharmacy, Amity University, Noida, India; 533 Research and Analytics Department, Initiative for Financing Health and Human Development, Chennai, India; 534 Department of Research and Analytics, Bioinsilico Technologies, Chennai, India; 535 Department of Engineering, Western Sydney University, Sydney, New South Wales, Australia; 536 Heart Failure Research Center, Isfahan University of Medical Sciences, Isfahan, Iran; 537 Neuroscience Research Center, Isfahan University of Medical Sciences, Isfahan, Iran; 538 Department of Physiotherapy, Tehran University of Medical Sciences, Tehran, Iran; 539 Research Center for War-affected People, Tehran University of Medical Sciences, Tehran, Iran; 540 University Institute of Public Health, The University of Lahore, Lahore, Pakistan; 541 Department of Dental Public Health, King Abdulaziz University, Jeddah, Saudi Arabia; 542 Department of Health Policy and Oral Epidemiology, Harvard University, Boston, Massachusetts; 543 College of Medicine and Health Sciences, United Arab Emirates University, Al Ain, United Arab Emirates; 544 Department of Circulation and Medical Imaging, Norwegian University of Science and Technology, Trondheim, Norway; 545 School of Medicine, Xiamen University, Xiamen, China; 546 Department of Forensic Medicine, Manipal Academy of Higher Education, Manipal, India; 547 Department of Health Promotion, Zahedan University of Medical Sciences, Zahedan, Iran; 548 Department of Anatomy and Embryology, Carol Davila University of Medicine and Pharmacy, Bucharest, Romania; 549 Department of Cardiology, Cardio-Aid, Bucharest, Romania; 550 Department of Cardiovascular Diseases, Tehran University of Medical Sciences, Tehran, Iran; 551 Department of Psychiatry, University of Oxford, Oxford, United Kingdom; 552 Department of Neurosciences, Kenya Medical Research Institute Wellcome Trust Research Programme, Kilifi, Kenya; 553 Cardiovascular Laboratory, Methodist Hospital, Merrillville, Merrillville, Indiana; 554 Department of Allergy, Immunology and Dermatology, Hanoi Medical University, Hanoi, Vietnam; 555 Faculty of Medicine, Duy Tan University, Da Nang, Vietnam; 556 Institute for Research and Training in Medicine, Biology and Pharmacy, Duy Tan University, Da Nang, Vietnam; 557 Cardiovascular Research Department, Methodist Hospital, Merrillville, Illinois; 558 Department of Surgery, Danang Family Hospital, Danang, Vietnam; 559 Department of General Medicine, University of Medicine and Pharmacy at Ho Chi Minh City, Ho Chi Minh City, Vietnam; 560 International Islamic University Islamabad, Islamabad, Pakistan; 561 Institute for Mental Health Policy Research, Centre for Addiction and Mental Health, Toronto, Ontario, Canada; 562 Departamento de Clínica Médica, Federal University of Minas Gerais, Belo Horizonte, Brazil; 563 Global Research Institute, Keio University, Tokyo, Japan; 564 Department of Global Health Policy, University of Tokyo, Tokyo, Japan; 565 Division of Cardiology, University of California San Francisco; 566 Department of Radiology, Mayo Clinic, Rochester, Minnesota; 567 School of Information, University of California Berkeley, Berkeley; 568 Center of Excellence in Reproductive Health Innovation (CERHI), University of Benin, Benin City, Nigeria; 569 Department of Applied Economics and Quantitative Analysis, University of Bucharest, Bucharest, Romania; 570 PSSM Data Sciences, Pfizer Inc, Groton, Connecticut; 571 Department of Gynecology and Obstetrics, Emory University, Atlanta, Georgia; 572 Health Promotion Research Center, Zahedan University of Medical Sciences, Zahedan, Iran; 573 University of Sydney, Sydney, New South Wales, Australia; 574 Department of Food and Nutrition, Seoul National University, Seoul, South Korea; 575 College of Medicine, University of Ibadan, Ibadan, Nigeria; 576 Department of Psychiatry and Behavioural Neurosciences, McMaster University, Hamilton, Ontario, Canada; 577 Department of Psychiatry, University of Lagos, Lagos, Nigeria; 578 Center for Clinical and Epidemiological Research, University of São Paulo, São Paulo, Brazil; 579 Associação Brasileira de Cefaleia em Salvas e Enxaqueca (ABRACES), São Paulo, Brazil; 580 Cardiology Department, Federal University of Rio de Janeiro, Rio de Janeiro, Brazil; 581 Department of Community Medicine, Ahmadu Bello University, Zaria, Nigeria; 582 Slum and Rural Health Initiative Research Academy, Slum and Rural Health Initiative, Ibadan, Nigeria; 583 Faculty of Public Health, University of Ibadan, Ibadan, Nigeria; 584 Department of Biotechnological and Applied Clinical Sciences, University of L’Aquila, L’Aquila, Italy; 585 Department of Neurology, ASL Avezzano-Sulmona-L’Aquila, L’Aquila, Italy; 586 One Health Global Research Group, Universidad de las Americas (University of the Americas), Quito, Ecuador; 587 School of Medicine, Western Sydney University, Bathurst, New South Wales, Australia; 588 Department of Optometry and Vision Science, University of KwaZulu-Natal, KwaZulu-Natal, South Africa; 589 Faculty of Medicine, University Ferhat Abbas of Setif, Setif, Algeria; 590 Division of Infectious Diseases, University Hospital of Setif, Setif, Algeria; 591 Department of Medicine, University of Ibadan, Ibadan, Nigeria; 592 Department of Medicine, University College Hospital, Ibadan, Ibadan, Nigeria; 593 School of Medicine, Johns Hopkins University, Baltimore, Maryland; 594 Miami Cancer Institute, Baptist Health South Florida, Miami; 595 Department of Respiratory Medicine, Jagadguru Sri Shivarathreeswara University, Mysore, India; 596 National School of Public Health, Institute of Health Carlos III, Madrid, Spain; 597 Department of Forensic Medicine and Toxicology, Manipal Academy of Higher Education, Mangalore, India; 598 Department of Nutrition and Dietetics, Harokopio University, Athens, Greece; 599 Board of Directors, National Public Health Organization, Athens, Greece; 600 School of Medicine, University of Nottingham, Nottingham, United Kingdom; 601 First Department of Ophthalmology, Aristotle University of Thessaloniki, Thessaloniki, Greece; 602 Department of Neurology, University of Bern, Bern, Switzerland; 603 Department of Neurology, University of Cyprus, Nicosia, Cyprus; 604 Department of Emergency Medicine, University of Thessaly, Larissa, Greece; 605 Department of Emergency Medicine, University of Bern, Bern, Switzerland; 606 Department of Epidemiology and Community Health, University of Minnesota, Minneapolis; 607 Department of Biomedical Data Science, Stanford University, Stanford, California; 608 Global Health Governance Programme, University of Edinburgh, Edinburgh, United Kingdom; 609 School of Dentistry, University of Leeds, Leeds, United Kingdom; 610 Department of Neurology and Public Health, Icahn School of Medicine at Mount Sinai, New York, New York; 611 Second Department of Cardiology, Aristotle University of Thessaloniki, Thessaloniki, Greece; 612 Clinical Research Department, IRCCS Fondazione Don Carlo Gnocchi, Milan, Italy; 613 School of Global Public Health, New York University, New York; 614 School of Population Health, Curtin University, Bentley, Western Australia, Australia; 615 Centre for Fertility and Health, Norwegian Institute of Public Health, Oslo, Norway; 616 Social and Economic Survey Research Institute, Qatar University, Doha, Qatar; 617 Mario Negri Institute for Pharmacological Research, Bergamo, Italy; 618 Department of Food, Environmental and Nutritional Sciences, University of Milan, Milano, Italy; 619 Facultad de Medicina (Faculty of Medicine), Diego Portales University, Santiago, Chile; 620 School of Cardiovascular and Metabolic Health, University of Glasgow, Glasgow, United Kingdom; 621 School of Pharmacy, University of Nizwa, Nizwa, Oman; 622 Research Center of Neurology, Moscow, Russia; 623 Research School of Chemistry and Applied Biomedical Sciences, Tomsk Polytechnic University, Tomsk, Russia; 624 Siberian State Medical University, Tomsk, Russia; 625 Department of Epidemiology and Evidence-Based Medicine, I.M. Sechenov First Moscow State Medical University, Moscow, Russia; 626 University Medical Center Groningen, University of Groningen, Groningen, Netherlands; 627 Center of Excellence in Higher Education for Pharmaceutical Care Innovation, Universitas Padjadjaran (Padjadjaran University), Bandung, Indonesia; 628 Department of Humanities and Social Sciences, National Institute of Technology Rourkela, Rourkela, India; 629 Department of Clinical Research and Epidemiology, Institute of Liver and Biliary Sciences, New Delhi, India; 630 Department of Biostatistics, Epidemiology, and Informatics, University of Pennsylvania, Philadelphia; 631 Cihan University-Sulaimaniya Research Center, Cihan University-Sulaimaniya, Sulaymaniyah, Iraq; 632 Department of Cardiology, Guiqian International General Hospital, Guiyang, China; 633 UO Neurologia, Salute Pubblica e Disabilità (The Neurology, Public Health and Disability Unit), Fondazione IRCCS Istituto Neurologico Carlo Besta (IRCCS Foundation Carlo Besta Neurological Institute), Milan, Italy; 634 Department of Medical Laboratory Technologies, Alnoor University, Mousl, Iraq; 635 Al-Noor Center of Research and Innovation, Alnoor University, Mousl, Iraq; 636 Department of Population Science and Human Resource Development, University of Rajshahi, Rajshahi, Bangladesh; 637 Institute of Health and Wellbeing, Federation University Australia, Berwick, Victoria, Australia; 638 Future Technology Research Center, National Yunlin University of Science and Technology, Yunlin, Taiwan; 639 Student Research Committee, Shahid Beheshti University of Medical Sciences, Tehran, Iran; 640 Department of Community Medicine, Employees’ State Insurance Model Hospital, Chennai, India; 641 Centre for Chronic Disease Control, New Delhi, India; 642 Department of Clinical Sciences, University of Sharjah, Sharjah, United Arab Emirates; 643 Department of Cardiology, Mansoura University, Mansoura, Egypt; 644 Department of Radiology, Stanford University, Stanford, California; 645 School of Nursing & Health Sciences, Hong Kong Metropolitan University, Hong Kong, China; 646 Saw Swee Hock School of Public Health, National University of Singapore, Singapore, Singapore; 647 Health Economics and Outcomes Research Department, Agios Pharmaceuticals, Cambridge, Massachusetts; 648 Department of Pharmaceutical Economics and Policy, Massachusetts College of Pharmacy and Health Sciences, Boston; 649 Brigham and Women’s Hospital, Harvard Medical School, Boston, Massachusetts; 650 Department of Epidemiology, Non-Communicable Diseases Research Center (NCDRC), Tehran, Iran; 651 Department of Family Medicine, Rajarata University of Sri Lanka, Anuradhapura, Sri Lanka; 652 Department of Primary Care and Public Health, Imperial College London, London, United Kingdom; 653 Academic Public Health England, Public Health England, London, United Kingdom; 654 Department of Biological Sciences, King Abdulaziz University, Jeddah, Egypt; 655 Department of Protein Research, Research and Academic Institution, Alexandria, Egypt; 656 Endocrinology and Metabolism Research Institute, Tehran University of Medical Sciences, Tehran, Iran; 657 Department of Epidemiology and Biostatistics, Rafsanjan University of Medical Sciences, Rafsanjan, Iran; 658 Community Health Department, Federal University of Ceará, Fortaleza, Brazil; 659 Department of Pharmacology and Toxicology, University of Antioquia, Medellin, Colombia; 660 Warwick Medical School, University of Warwick, Coventry, United Kingdom; 661 Department of Clinical Research, University of Sao Paulo, Ribeirão Preto, Brazil; 662 Gilbert and Rose-Marie Chagoury School of Medicine, Lebanese American University, Beirut, Lebanon; 663 Maurizio Bufalini Hospital, Cesena, Italy; 664 Fondazione Policlinico Universitario A. Gemelli, Cuore Università Cattolica del Sacro Cuore (Catholic University of Sacred Heart), Rome, Italy; 665 Department of Analytical and Applied Economics, Utkal University, Bhubaneswar, India; 666 RUSA Centre of Excellence in Public Policy and Governance, Utkal University, Bhubaneswar, India; 667 Department of Biochemistry and Food Analysis, Patuakhali Science and Technology University, Patuakhali, Bangladesh; 668 Department of Labour, Directorate of Factories, Government of West Bengal, Kolkata, India; 669 Cardiovascular Department, Zagazig University, Zagazig, Egypt; 670 Faculty of Medicine, Gonabad University of Medical Sciences, Gonabad, Iran; 671 Infectious Diseases Research Center, Gonabad University of Medical Sciences, Gonabad, Iran; 672 Department of Epidemiology, Shahid Beheshti University of Medical Sciences, Tehran, Iran; 673 Department of Neurology, University of L’Aquila, L’Aquila, Italy; 674 College of Medicine, University of Sharjah, Sharjah, United Arab Emirates; 675 School of Population Health, University of New South Wales, Sydney, New South Wales, Australia; 676 Department of Biostatistics, Shiraz University of Medical Sciences, Shiraz, Iran; 677 Institute of Medical Science, University of Toronto, Toronto, Ontario, Canada; 678 Hurvitz Brain Sciences Research Program, Sunnybrook Research Institute, Toronto, Ontario, Canada; 679 Sharjah Institute of Medical Sciences, University of Sharjah, Sharjah, United Arab Emirates; 680 Center for Global Health Research, Saveetha University, Chennai, India; 681 Biotechnology Research Center, Mashhad University of Medical Sciences, Mashhad, Iran; 682 Department of Analytical & Applied Economics, Utkal University, Bhubaneswar, India; 683 Department of Health and Kinesiology, University of Illinois, Urbana-Champaign; 684 Department of Integrated Health Education, Federal University of Espirito Santo, Vitória, Brazil; 685 Faculty of Pharmacy, Mansoura University, Mansoura, Egypt; 686 Institute of Epidemiology and Preventive Medicine, National Taiwan University, Taipei, Taiwan; 687 Benang Merah Research Center (BMRC), Minahasa Utara, Indonesia; 688 Department of Anatomy, Ras Al Khaimah Medical and Health Sciences University, Ras Al Khaimah, United Arab Emirates; 689 Department of Entomology, Ain Shams University, Cairo, Egypt; 690 Medical Ain Shams Research Institute (MASRI), Ain Shams University, Cairo, Egypt; 691 School of Public Health and Health Management, University of Belgrade, Belgrade, Serbia; 692 Indira Gandhi Medical College and Research Institute, Puducherry, India; 693 Department of Food Processing Technology, West Bengal State Council of Technical Education, Malda, India; 694 Department of Oral Pathology and Microbiology, Dr. D. Y. Patil Vidyapeeth, Pune (Deemed to be University), Pune, India; 695 Faculty of Medicine, The University of Queensland, Brisbane, Queensland, Australia; 696 Nuffield Department of Medicine, University of Oxford, Oxford, United Kingdom; 697 UGC Centre of Advanced Study in Psychology, Utkal University, Bhubaneswar, India; 698 Udyam-Global Association for Sustainable Development, Bhubaneswar, India; 699 Dobney Hypertension Centre, The University of Western Australia, Perth, Western Australia, Australia; 700 Hypertension and Kidney Disease Laboratory, Baker Heart and Diabetes Institute, Melbourne, Victoria, Australia; 701 Department of Health Sciences, Federal University of Santa Catarina, Araranguá, Brazil; 702 Cardiovascular Research Center, Massachusetts General Hospital, Boston, Massachusetts; 703 Department of Cardiovascular Sciences, Katholieke Universiteit Leuven, Leuven, Belgium; 704 Department of Community Oral Health and Clinical Prevention, University of Malaya, Kuala Lumpur, Malaysia; 705 Emergency Department, Manian Medical Centre, Erode, India; 706 Digestive Diseases Research Institute, Tehran University of Medical Sciences, Tehran, Iran; 707 Non-communicable Disease Research Center, Shiraz University of Medical Sciences, Shiraz, Iran; 708 Department of Medicine and Surgery, Government Doon Medical College, Dehradun, India; 709 National Heart, Lung, and Blood Institute, National Institutes of Health, Rockville, Maryland; 710 Department of Global Public Health, Karolinska Institute, Stockholm, Sweden; 711 Department of Neurology, Tehran University of Medical Sciences, Tehran, Iran; 712 Center for Medical and Bio-Allied Health Sciences Research, Ajman University, Ajman, United Arab Emirates; 713 Independent Consultant, Karachi, Pakistan; 714 Department of Neuro-Physiotherapy, Independent Consultant, Thane, India; 715 Centre For Interdisciplinary Research In Basic Sciences (CIRBSc), Jamia Millia Islamia, New Delhi, India; 716 Science Department, Kazakh National Medical University, Almaty, Kazakhstan; 717 College of Nursing and Health Sciences, Jazan University, Jazan, Saudi Arabia; 718 Amity Institute of Public Health, Amity University, Noida, India; 719 Department for Evidence-based Medicine and Evaluation, University for Continuing Education Krems, Krems, Austria; 720 Sina Hospital, Tehran University of Medical Sciences, Tehran, Iran; 721 Department of Medicine, Korea University, Seoul, South Korea; 722 Institute of Forensic Science & Criminology, Panjab University, Chandigarh, India; 723 Department of Nursing, Arba Minch University, Arba Minch, Ethiopia; 724 K S Hegde Medical Academy, Nitte University, Mangalore, India; 725 Manipal College of Dental Sciences, Mangalore, Manipal Academy of Higher Education, Mangalore, India; 726 National Institute of Infectious Diseases, Tokyo, Japan; 727 Department of Veterinary Public Health and Preventive Medicine, Usmanu Danfodiyo University, Sokoto, Sokoto, Nigeria; 728 Oulu Business School, University of Oulu, Oulu, Finland; 729 Martti Ahtisaari Institute, University of Oulu, Oulu, Finland; 730 Neurological Surgery, Northwestern University, Chicago, Illinois; 731 Department of Medical-Surgical Nursing, Mazandaran University of Medical Sciences, Sari, Iran; 732 Department of Nursing and Health Sciences, Flinders University, Adelaide, South Australia, Australia; 733 Unit of Basic Medical Sciences, University of Khartoum, Khartoum, Sudan; 734 Department of Medical Microbiology and Infectious Diseases, Erasmus University, Rotterdam, Netherlands; 735 Department of Biochemistry, Central University of Punjab, Bathinda, India; 736 Department of Radiodiagnosis, All India Institute of Medical Sciences, Bathinda, India; 737 Department of Human Genetics, Punjabi University, Patiala, India; 738 Department of Health Education and Promotion, Jazan University, Jazan, Saudi Arabia; 739 Department of Systemic Pathology, Touro College of Osteopathic Medicine, Middletown, New York; 740 Department of Pathology, American University of the Caribbean School of Medicine, Cupecoy, Saint Martin; 741 Department of Biochemistry, American University of Integrative Sciences, Bridgetown, Barbados; 742 Student Research Committee, Urmia University of Medical Sciences, Urmia, Iran; 743 School of Medicine, Babol University of Medical Sciences, Babol, Iran; 744 3rd Department of Cardiology, University of Athens, Athens, Greece; 745 Department of Pharmacology, RAK Medical and Health Sciences University, Ras Al Khaimah, United Arab Emirates; 746 Department of Public Health, Kandahar University, Kandahar, Afghanistan; 747 Nutrition and Dietetics Department, Federal Research Institute of Nutrition, Biotechnology and Food Safety, Moscow, Russia; 748 Department of Internal Disease, Pirogov Russian National Research Medical University, Moscow, Russia; 749 Institute of Integrated Intelligence and Systems, Griffith University, Brisbane, Queensland, Australia; 750 Department of Biomedical Sciences, Universiti Putra Malaysia, Selangor, Malaysia; 751 Department of Clinical Research and Development, LUXMED Group, Warsaw, Poland; 752 Collegium Medicul, John Paul II Catholic University of Lublin, Lublin, Poland; 753 Department of Neurology, Neurocenter of Southern Switzerland (NSI), Lugano, Switzerland; 754 Department of Medical Informatics, Mashhad University of Medical Sciences, Mashhad, Iran; 755 Clinial Research Development Unit, Mashhad University of Medical Sciences, Mashhad, Iran; 756 Department of Basic Medical Sciences, Islamic Azad University, Mashhad, Iran; 757 Department of Internal Medicine, Islamic Azad University, Mashhad, Iran; 758 Department of Environmental, Agricultural and Occupational Health, University of Nebraska Medical Center, Omaha; 759 Sri Ramachandra Medical College and Research Institute, Chennai, India; 760 Department of Pathology, Alexandria University, Alexandria, Egypt; 761 Department of Epidemiology, Stellenbosch University, Cape Town, South Africa; 762 Department of Medicine, Northlands Medical Group, Omuthiya, Namibia; 763 Department of Surgery, National University of Singapore, Singapore, Singapore; 764 Pediatric Intensive Care Unit, King Saud University, Riyadh, Saudi Arabia; 765 Department of Epidemiology and Biostatistics, University of California San Francisco; 766 School of Humanities and Social Sciences, Indian Institute of Technology Mandi, Mandi, India; 767 Public Health Department, Amrita Institute of Medical Sciences, Kochi, India; 768 Faculty of Medicine, University of Southampton, Southampton, United Kingdom; 769 Department of Family and Preventive Medicine, Emory University, Atlanta, Georgia; 770 Faculty of Public Health, Universitas Sam Ratulangi (Sam Ratulangi University), Manado, Indonesia; 771 Department of Medicine, University of Calgary, Calgary, Alberta, Canada; 772 Institute of Public Health, Jagiellonian University Medical College, Kraków, Poland; 773 Agency for Health Technology Assessment and Tariff System, Warsaw, Poland; 774 School of Medicine, Indiana University, Indianapolis; 775 Department of Internal Medicine, University of Medicine and Pharmacy at Ho Chi Minh City, Ho Chi Minh City, Vietnam; 776 Department of Business Analytics, University of Massachusetts Dartmouth, Dartmouth; 777 Molecular Neuroscience Research Center, Shiga University of Medical Science, Shiga, Japan; 778 Rigshospitalet, University of Copenhagen, Copenhagen, Denmark; 779 Faculty of Medicine, Nam Can Tho University, Can Tho, Vietnam; 780 School of Pharmacy, National Cheng Kung University, Tainan, Taiwan; 781 Centre for Neonatal and Paediatric Infection, St George’s University of London, London, United Kingdom; 782 Natural and Medical Sciences Research Center, University of Nizwa, Nizwa, Oman; 783 Department of Cardiovascular, Endocrine-metabolic Diseases and Aging, National Institute of Health, Rome, Italy; 784 Department of Neuroscience, Monash University, Clayton, Victoria, Australia; 785 Department of Informatics and Radiology, Mayo Clinic, Rochester, Minnesota; 786 College of Health and Sport Sciences, University of Bahrain, Zallaq, Bahrain; 787 Urmia University of Medical Sciences, Urmia, Iran; 788 College of Public Health and Tropical Medicine, Jazan University, Jazan, Saudi Arabia; 789 UKK Institute, Tampere, Finland; 790 Faculty of Medicine and Health Technology, Tampere University, Tampere, Finland; 791 Raffles Neuroscience Centre, Raffles Hospital, Singapore, Singapore; 792 Yong Loo Lin School of Medicine, National University of Singapore, Singapore, Singapore; 793 Department of Health Policy and Management, Johns Hopkins University, Baltimore, Maryland; 794 Department of Physiotherapy, Universidad Europea de Madrid (European University of Madrid), Villaviciosa de Odón, Spain; 795 Department of Cardiology, Icahn School of Medicine at Mount Sinai, New York, New York; 796 Department of Molecular Epidemiology, Research Institute for Systems Biology and Medicine, Moscow, Russia; 797 Department of Information Technologies and Management, Moscow Institute of Physics and Technology, Dolgoprudny, Russia; 798 School of Population Health and Environmental Sciences, King’s College London, London, United Kingdom; 799 NUST School of Health Sciences, National University of Sciences and Technology (NUST), Islamabad, Pakistan; 800 Operational Research Center in Healthcare, Near East University, Nicosia, Turkiye; 801 Department of Interventional Radiology, University of Miami, Miami, Florida; 802 Department of Psychiatry, Haramaya University, Harar, Ethiopia; 803 School of Life Course and Population Sciences, King’s College London, London, United Kingdom; 804 Department of Community Medicine, Rajarata University of Sri Lanka, Anuradhapura, Sri Lanka; 805 Institute of Clinical Epidemiology, Public Health, Health Economics, Medical Statistics and Informatics, Medical University Innsbruck, Innsbruck, Austria; 806 Department of Public Health and Primary Care, University of Cambridge, Cambridge, United Kingdom; 807 National Data Management Center for Health (NDMC), Ethiopian Public Health Institute, Addis Ababa, Ethiopia; 808 NIHR Biomedical Research Centre, Guy’s and St Thomas’ Hospital and Kings College London, London, United Kingdom; 809 Emergency medicine and critical care nursing, Bahir Dar University, Bahir Dar, Ethiopia; 810 School of Public Health, Zhejiang University, Zhejiang, China; 811 Department of Public Health Science, Fred Hutchinson Cancer Research Center, Seattle, Washington; 812 Department of Endocrinology, University of Science and Technology of China, Hefei, China; 813 School of Medicine, University of Rochester, Rochester, New York; 814 Cardiovascular Program, The George Institute for Global Health, Sydney, New South Wales, Australia; 815 Department of Public Health, Juntendo University, Tokyo, Japan; 816 Department of Public Health Medicine, University of Tsukuba, Tsukuba, Japan; 817 Faculty of Medicine, Juntendo University, Tokyo, Japan; 818 Department of Medicine, Shiraz University of Medical Sciences, Shiraz, Iran; 819 Department of Medicine, Mashhad University of Medical Sciences, Mashhad, Iran; 820 Research Center of Physiology, Semnan University of Medical Sciences, Semnan, Iran; 821 The George Institute for Global Health, Imperial College London, London, United Kingdom; 822 National Center for Chronic and Noncommunicable Disease Control and Prevention, Chinese Center for Disease Control and Prevention, Beijing, China; 823 The George Institute for Global Health, University of New South Wales, Sydney, New South Wales, Australia; 824 Department of Pediatrics, Kyung Hee University, Seoul, South Korea; 825 Department of Biostatistics, University of Toyama, Toyama, Japan; 826 Department of Epidemiology and Biostatistics, Wuhan University, Wuhan, China; 827 Sant’Elia Hospital, University of Catania, Caltanissetta, Italy; 828 Research and Development Department, Sina Medical Biochemistry Technologies, Shiraz, Iran; 829 Department of Bioengineering and Therapeutical Sciences, University of California San Francisco; 830 Department of Administration, PGxAI, San Francisco, California; 831 Department of Neurology, Xuanwu Hospital, Beijing, China; 832 Department of Neurology, PLA Rocket Force Characteristic Medical Center, Beijing, China; 833 School of Public Health, Wuhan University of Science and Technology, Wuhan, China; 834 Hubei Province Key Laboratory of Occupational Hazard Identification and Control, Wuhan University of Science and Technology, Wuhan, China; 835 School of Public Health, Wuhan University, Wuhan, China; 836 Tianjin Medical University General Hospital, Tianjin Centers for Disease Control and Prevention, Tianjin, China; 837 College of Traditional Chinese Medicine, Hebei University, Baoding, China; 838 School of Humanities and Management, Zhejiang Chinese Medical University, Hangzhou, China; 839 Department of Epidemiology, University of Washington, Seattle; 840 Institute of Public Health and Social Sciences, Khyber Medical University, Peshawar, Pakistan; 841 Department of Biochemistry and Pharmacogenomics, Medical University of Warsaw, Warsaw, Poland; 842 Division of Cardiology, University of Washington, Seattle

## Abstract

**Importance:**

Nontraumatic subarachnoid hemorrhage (SAH) represents the third most common stroke type with unique etiologies, risk factors, diagnostics, and treatments. Nevertheless, epidemiological studies often cluster SAH with other stroke types leaving its distinct burden estimates obscure.

**Objective:**

To estimate the worldwide burden of SAH.

**Design, Setting, and Participants:**

Based on the repeated cross-sectional Global Burden of Disease (GBD) 2021 study, the global burden of SAH in 1990 to 2021 was estimated. Moreover, the SAH burden was compared with other diseases, and its associations with 14 individual risk factors were investigated with available data in the GBD 2021 study. The GBD study included the burden estimates of nontraumatic SAH among all ages in 204 countries and territories between 1990 and 2021.

**Exposures:**

SAH and 14 modifiable risk factors.

**Main Outcomes and Measures:**

Absolute numbers and age-standardized rates with 95% uncertainty intervals (UIs) of SAH incidence, prevalence, mortality, and disability-adjusted life-years (DALYs) as well as risk factor–specific population attributable fractions (PAFs).

**Results:**

In 2021, the global age-standardized SAH incidence was 8.3 (95% UI, 7.3-9.5), prevalence was 92.2 (95% UI, 84.1-100.6), mortality was 4.2 (95% UI, 3.7-4.8), and DALY rate was 125.2 (95% UI, 110.5-142.6) per 100 000 people. The highest burden estimates were found in Latin America, the Caribbean, Oceania, and high-income Asia Pacific. Although the absolute number of SAH cases increased, especially in regions with a low sociodemographic index, all age-standardized burden rates decreased between 1990 and 2021: the incidence by 28.8% (95% UI, 25.7%-31.6%), prevalence by 16.1% (95% UI, 14.8%-17.7%), mortality by 56.1% (95% UI, 40.7%-64.3%), and DALY rate by 54.6% (95% UI, 42.8%-61.9%). Of 300 diseases, SAH ranked as the 36th most common cause of death and 59th most common cause of DALY in the world. Of all worldwide SAH-related DALYs, 71.6% (95% UI, 63.8%-78.6%) were associated with the 14 modeled risk factors of which high systolic blood pressure (population attributable fraction [PAF] = 51.6%; 95% UI, 38.0%-62.6%) and smoking (PAF = 14.4%; 95% UI, 12.4%-16.5%) had the highest attribution.

**Conclusions and Relevance:**

Although the global age-standardized burden rates of SAH more than halved over the last 3 decades, SAH remained one of the most common cardiovascular and neurological causes of death and disabilities in the world, with increasing absolute case numbers. These findings suggest evidence for the potential health benefits of proactive public health planning and resource allocation toward the prevention of SAH.

## Key Points

**Question:**

What is the global burden of nontraumatic subarachnoid hemorrhage (SAH)?

**Findings:**

Results of this cross-sectional study, based on the Global Burden of Disease 2021 study, reveal that in 2021, there were 700 000 new SAH cases, almost 8 million patients with prevalent SAH, 350 000 SAH deaths, and over 10 million SAH-related disability-adjusted life-years globally. Despite decreasing age-standardized burden rates, SAH remained one of the most common cardiovascular and neurological causes of death and disability in the world.

**Meaning:**

Given the high proportional burden of SAH, study results suggest evidence for the potential health benefits of proactive public health planning and resource allocation for SAH prevention.

## Teaser

This cross-sectional study investigates the global burden of nontraumatic subarachnoid hemorrhage in 2021.

## Introduction

Nontraumatic subarachnoid hemorrhage (SAH) represents the third most common stroke type after ischemic stroke and intracerebral hemorrhage, accounting for 5% to 10% of all strokes.^1,2^ Of all nontraumatic SAHs, approximately 85% are caused by the rupture of an intracranial aneurysm, which distinguishes its etiology, risk factors, symptoms, diagnostics, treatments, and outcomes from other types of stroke.^3^ Even though comprehensive SAH-specific burden and risk factor estimates would be crucial for its accurate evidence-based health care planning and resource allocation, SAH is still frequently clustered with other stroke types leaving its unique epidemiology and prevention strategies obscure.

Consistent with various population-based studies worldwide,^4,5,6^ a recent Global Burden of Diseases (GBD) 2021 stroke study reported that the age-standardized burden rates of SAH and other stroke types decreased globally with a substantial geographical variation.^2^ However, because the study focused mainly on stroke in general,^2^ many SAH-specific findings such as its rankings against the burden estimates of other critical health outcomes were not reported. Therefore, we decided to use the GBD 2021 dataset and focus solely on the global, regional, and national burden of SAH and its risk factors over the last 3 decades. Primarily, we hypothesized that even though the age-standardized burden rates of SAH are decreasing, they still consist of a substantial proportion of the burden related to cardiovascular, neurological, and noncommunicable diseases. This article was produced as part of the GBD Collaborator Network, and in accordance with the GBD protocol.^7^

## Methods

### Overview

Details of the GBD methodology are presented elsewhere.^8,9^ In brief, GBD studies have been conducted since 1990 to provide annual standardized burden estimates of critical health outcomes and their attribution to behavioral, environmental, and metabolic risks worldwide. By aiming to use all available evidence via its repeated cross-sectional study design, the GBD studies use censuses, household surveys, vital registrations, administrative data collections, disease registers, verbal autopsy tools, air pollution monitors, satellite imaging, and scientific literature as its primary data sources. Based on the actual data points and relevant predictive covariates, the final and missing data are further estimated using various statistical models for fatal and nonfatal burden estimates (eAppendix, eMethods, and eTable 1 in Supplement 1). Because the data sources and assessments of the whole time series are reevaluated and updated in pursuance of every annual release, the latest GBD results supersede the preceding estimates. In the most recent data release, the GBD 2021 study used over 607 billion data points to illustrate the annual burden of 371 diseases and injuries as well as 88 risk factors from 204 countries and territories between 1990 and 2021. The exact data sources are publicly available through the website of the Institute for Health Metrics and Evaluation.^10^ Because GBD studies rely on the analysis of aggregated secondary data without the direct involvement of human subjects, individual studies based on the publicly available database do not require separate approvals from institutional review boards or informed consent from individuals whose health conditions are studied. The reporting of this manuscript followed the Guidelines for Accurate and Transparent Health Estimates Reporting (GATHER) recommendations.^11^

### SAH Definition and Data Sources

In line with the definition of the World Health Organization, the GBD 2021 study defines SAH as a nontraumatic stroke type caused by bleeding into the subarachnoid space of the brain. This definition includes first-ever SAHs with aneurysmal and nonaneurysmal origins but excludes recurrent SAHs and secondary SAHs caused by intracranial traumas. Correspondingly, the study uses primarily code 430 from the *International Classification of Diseases, Ninth Revision *(*ICD-9*) and code I60 from the *Tenth Revision* (*ICD-10*) to identify relevant data sources and outcomes. According to the 4-level categorization of the GBD studies, SAH belongs to the most specific level 4 category being also part of the categories of noncommunicable diseases (level 1), cardiovascular diseases (level 2), and strokes (level 3). Overall, the GBD 2021 study comprises 2563 data sources for fatal SAHs, 311 data sources for nonfatal SAHs, and 36 data sources for SAH risk factors from 132 different countries/regions between 1963 and 2022 (eAppendix, eMethods, and eFigures 1-4 in Supplement 1).

### Risk Factor Estimation

Similar to causes, the GBD classifies risk factors into 4 different levels from the broadest level 1 to the most detailed level 4. For SAH, the GBD 2021 study has available data for all 3 risk clusters of level 1 and 14 individual risk factors from levels 2 to 4 (eAppendix, eMethods, and eTable 2 in Supplement 1). To evaluate the association of 3 risk clusters and 14 available risk factors with SAH-specific burden, the GBD 2021 study uses a comparative assessment framework to calculate population attributable fractions (PAFs) defined as a theoretical proportion of burden that could be prevented by changing the exposure of the risk to the theoretical minimum risk exposure level in the population (eAppendix and eMethods in Supplement 1).^12^

### Statistical Analysis

Based on the available data input sources and assumptions of geospatial relationships between relevant covariates such as smoking prevalence, systolic blood pressure, and lag distributed income per capita, the GBD uses primarily 2 statistical modeling tools, namely, cause of death ensemble modeling (fatal estimates) and disease-model bayesian meta-regression 2.1 (nonfatal estimates), to produce annual burden estimates of SAH across different population groups and geographical locations between 1990 and 2021 (Appendix, eMethods, and eTable 1 in Supplement 1).^8,9^ Consistent with previous GBD stroke reports,^2,13,14^ we used the absolute numbers and age-standardized (adjusted to the age structure of the GBD standard population) rates per 100 000 people of 4 outcomes to illustrate the burden of SAH as follows: (1) incidence, (2) prevalence, (3) deaths, and (4) DALYs (eAppendix, eMethods, and eTable 3 in Supplement 1). According to the cause-specific number of deaths and DALYs, we also compared the absolute burden of SAH within 3 stroke types, 11 neurological disorders including strokes, 18 cardiovascular diseases, 192 noncommunicable diseases, and 300 individual diseases/injuries on the same hierarchy level with associated deaths or DALYs (eAppendix, eMethods, and eTable 3 in Supplement 1). To present the attributions of risk factors to SAH-related DALY estimates, we presented the age-standardized PAFs in percentages and DALY rates per 100 000 people attributed to each included risk factor or cluster. Besides average global estimates in 2021, we presented all burden estimates with 95% uncertainty intervals (UIs) and stratified by sex, 4 age groups, 5 Sociodemographic Index (SDI) levels, 7 GBD super regions, 21 GBD regions, and 204 individual countries and territories (eAppendix, eMethods, and eTable 3 in Supplement 1). Lastly, we evaluated the temporal trends by comparing the burden and risk factor estimates between 1990 and 2021. All analyses of the current study are based on the publicly available GBD results^15^ and visualization tools.^16^ Data were analyzed from 1990 to 2021.

## Results

### Global Burden of SAH in 2021

Based on the overall global estimates, we observed 0.7 (95% UI, 0.6-0.8) million new SAH cases, 7.9 (95% UI, 7.2-8.6) million patients with prevalent SAH, 0.4 (95% UI, 0.3-0.4) million SAH deaths, and 10.6 (95% UI, 9.4-12.1) million SAH-related DALYs in 2021 (Table 1). These resulted in the age-standardized SAH incidence of 8.3 (95% UI, 7.3-9.5), prevalence of 92.2 (95% UI, 84.1-100.6), mortality of 4.2 (95% UI, 3.7-4.8), and DALY rate of 125.2 (95% UI, 110.5-142.6) per 100 000 people. Although the prevalence of SAH was higher in female individuals than in male individuals, the point estimates of other age-standardized burden figures were higher in male patients (Table 1). The rates of all burden estimates of both sexes increased along with increasing age (Table 1 and eFigure 5 in Supplement 1).

**Table 1.** Global Number and Age-Standardized Rates With 95% Uncertainty Intervals (UIs) of Subarachnoid Hemorrhage Incidence, Prevalence, Mortality, and Disability-Adjusted Life-Years (DALYs) in 2021^a^

Group	Incidence	Prevalence	Mortality	DALY
**Overall**				
Absolute No. in millions (95% UI)	0.70 (0.61-0.80)	7.85 (7.16-8.58)	0.35 (0.31-0.40)	10.64 (9.40-12.12)
Age-standardized rate per 100 000 people (95% UI)	8.33 (7.34-9.48)	92.17 (84.08-100.60)	4.18 (3.66-4.76)	125.20 (110.54-142.61)
**Female**				
Absolute No. in millions (95% UI)	0.36 (0.32-0.41)	4.31 (3.95-4.69)	0.18 (0.16-0.21)	5.16 (4.62-5.89)
Age-standardized rate per 100 000 people (95% UI)	8.17 (7.21-9.35)	97.88 (89.66-106.58)	3.91 (3.41-4.55)	116.35 (104.22-133.10)
**Male**				
Absolute No. in millions (95% UI)	0.34 (0.30-0.39)	3.54 (3.22-3.89)	0.17 (0.14-0.22)	5.48 (4.50-6.90)
Age-standardized rate per 100 000 people (95% UI)	8.51 (7.48-9.65)	85.52 (77.67-93.74)	4.48 (3.64-5.56)	134.07 (109.87-167.87)
**Children (0-14 y)**				
Absolute No. in millions (95% UI)	0.034 (0.023-0.046)	0.21 (0.17-0.25)	0.0033 (0.0026-0.0041)	0.31 (0.25-0.37)
Rate per 100 000 people (95% UI)	1.67 (1.16-2.29)	10.27 (8.49-12.50)	0.16 (0.13-0.20)	15.29 (12.34-18.51)
**Young adults (15-49 y)**				
Absolute No. in millions (95% UI)	0.24 (0.19-0.30)	2.71 (2.43-3.04)	0.055 (0.047-0.067)	3.19 (2.74-3.82)
Rate per 100 000 people (95% UI)	6.13 (4.83-7.50)	68.63 (61.46-76.87)	1.39 (1.19-1.69)	80.75 (69.42-96.78)
**Old adults (50-74 y)**				
Absolute No. in millions (95% UI)	0.29 (0.24-0.37)	3.97 (3.55-4.42)	0.17 (0.15-0.20)	5.49 (4.85-6.26)
Rate per 100 000 people (95% UI)	17.90 (14.67-22.24)	241.76 (216.37-268.90)	10.57 (9.25-12.15)	334.32 (295.19-381.09)
**Very old adults (≥75 y)**				
Absolute No. in millions (95% UI)	0.13 (0.10-0.16)	0.97 (0.83-1.11)	0.12 (0.10-0.14)	1.66 (1.43-1.86)
Rate per 100 000 people (95% UI)	44.35 (35.94-54.15)	334.57 (286.56-386.05)	41.93 (35.80-47.51)	573.62 (494.79-643.62)

^a^ The results are presented overall and separately for female and male individuals and 4 age groups.

### Regional and National Burden of SAH in 2021

**Figure 1.**
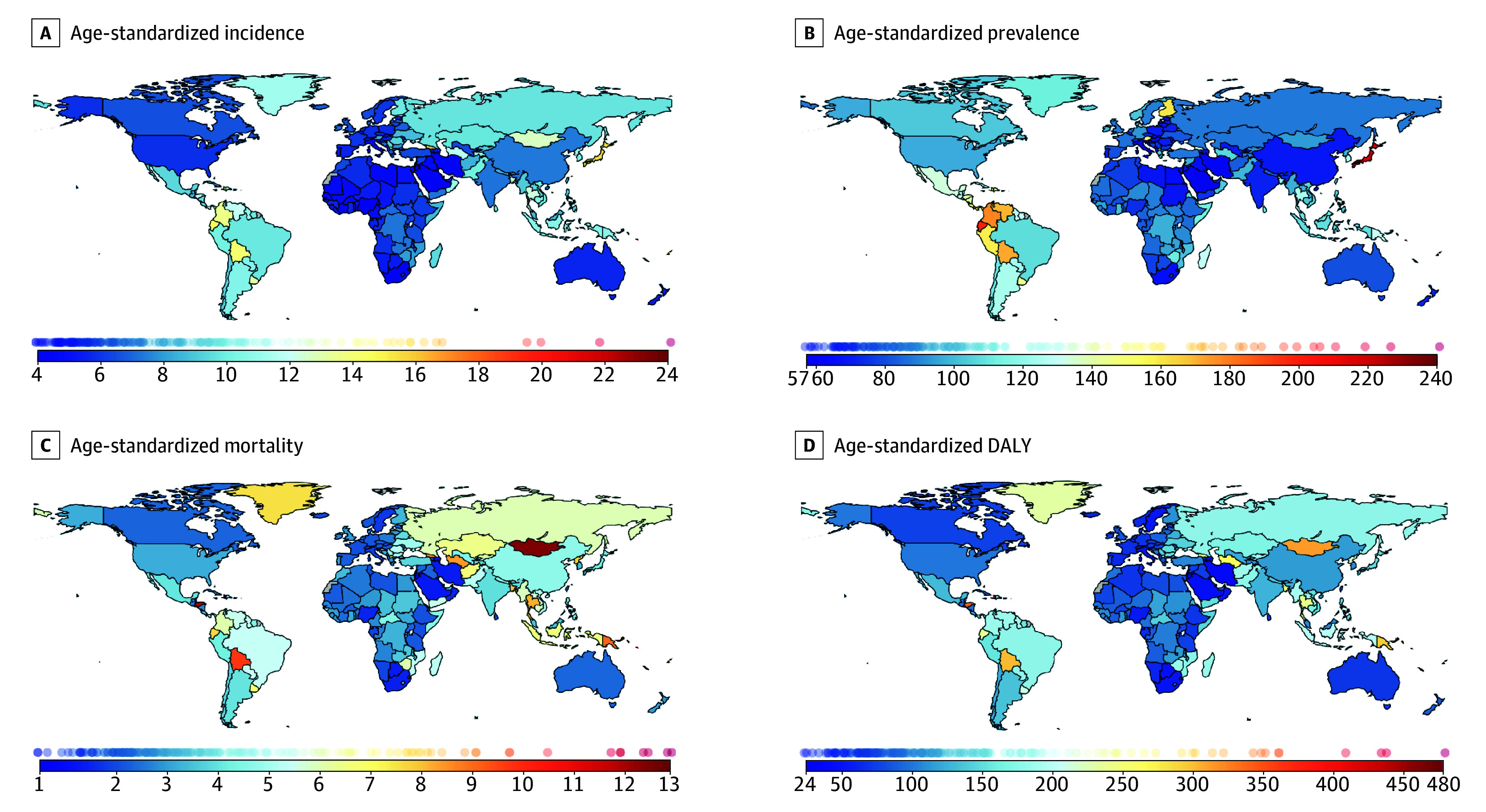
Worldwide Burden of Subarachnoid Hemorrhage in 2021 Age-standardized incidence (A), prevalence (B), mortality (C), and disability-adjusted life-years (DALYs) (D) rates of subarachnoid hemorrhage per 100 000 people in 204 countries and territories in 2021. The circles above the scales represent the estimates from individual countries. Figure created with The Institute for Health Metrics and Evaluation, Global Burden of Diseases Study 2021.^16^

All age-standardized burden estimates of SAH varied substantially between 204 countries and territories worldwide (Figure 1 and eTables 4-11 in Supplement 1). By SDI level, we found the highest age-standardized prevalence as well as the lowest mortality and DALY rates of SAH in high-SDI regions (eTable 5 in Supplement 1). On the other hand, the highest age-standardized incidence, mortality, and DALY rates occurred in low-middle– and middle-SDI regions (eTable 5 in Supplement 1). According to the 7 GBD super regions, we found the lowest age-standardized mortality and DALY rates in North Africa and the Middle East, Sub-Saharan Africa, and high-income super regions, whereas the lowest incidence rates were observed in North Africa, the Middle East, and Sub-Saharan Africa. Latin America and the Caribbean had, in turn, the highest age-standardized rates of all 4 burden estimates (eTable 5 in Supplement 1). These geographical differences also varied slightly between male and female individuals (eTables 6 and 7 in Supplement 1) as well as between different age groups (eTables 8-11 in Supplement 1). In high-SDI regions, we observed that female individuals had higher incidence, prevalence, mortality, and DALY rates compared with males, but these differences were not observed in other SDI regions.

### Temporal Changes in SAH Burden

**Figure 2.**
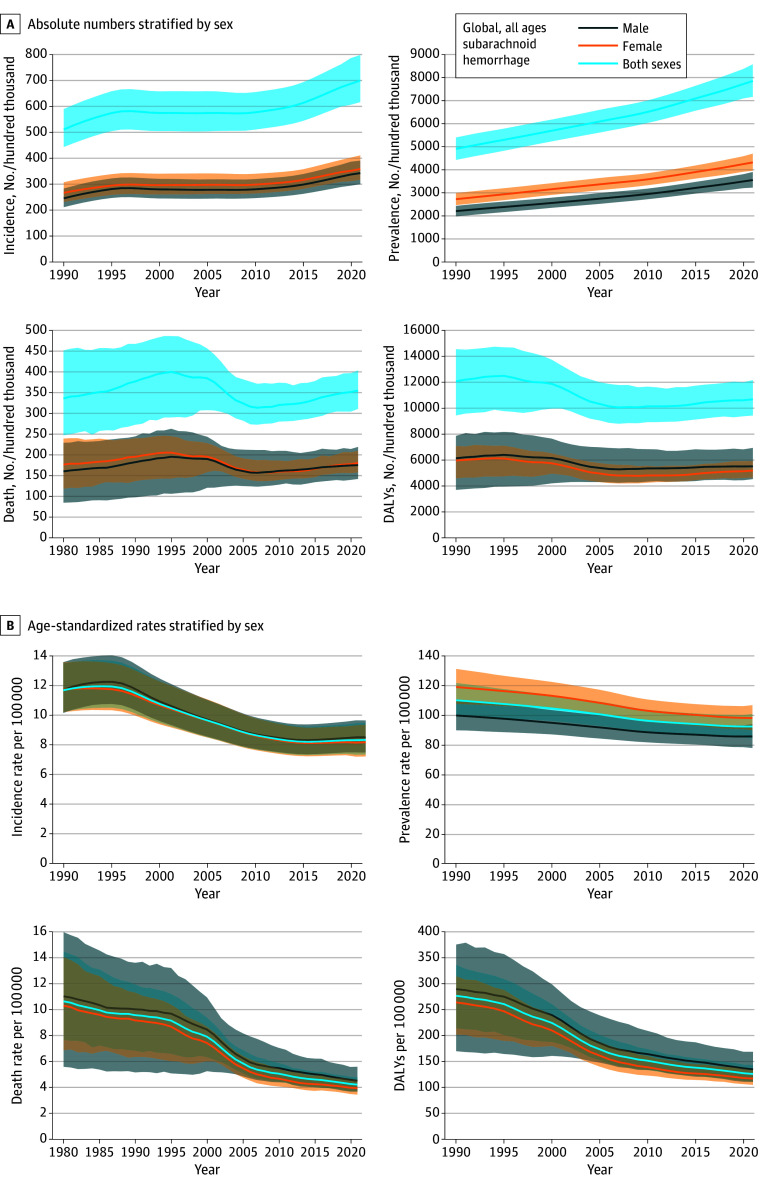
Changes in the Incidence, Prevalence, Deaths, and Disability-Adjusted Life-Years (DALYs) of Subarachnoid Hemorrhage in the World Between 1990 (Deaths Since 1980) and 2021 Results are presented as absolute number (A) and age-standardized rates (B) per 100 000 people as well as stratified by sex. Solid lines represent the changes in point estimates, and shaded areas represent 95% uncertainty intervals. Figures created with The Institute for Health Metrics and Evaluation, Global Burden of Diseases Study 2021.^15^

Between 1990 and 2021, the absolute number of annual new SAH incidents increased from 0.5 to 0.7 million (37.1%; 95% UI 32.2%-42.4%) and prevalent cases from 4.9 to 7.9 million (60.2%; 95% UI, 56.9%-63.4%). Moreover, the absolute number of global deaths and DALYs due to SAH had an increasing trend since 2005 (Figure 2A). However, according to the age-standardized rates per 100 000 people, all burden estimates of SAH decreased worldwide between 1990 and 2021, with incidence from 11.7 to 8.3 (28.8%; 95% UI, 25.7%-31.6%), prevalence from 109.9 to 92.2 (16.1%; 95% UI, 14.8%-17.7%), mortality from 9.5 to 4.2 (56.1%; 95% UI, 40.7%-64.3%), and DALY rate from 275.9 to 125.2 (54.6%; 95% UI, 42.8%-61.9%) (Figure 2B and eTable 12 in Supplement 1). Although the absolute number of new SAH incidents and prevalence increased among all SDI levels, the increases were more evident in low- and low-middle–SDI regions where the absolute number of deaths and DALYs also increased over the whole study period (eFigure 10 and eTables 13 and 14 in Supplement 1). Moreover, we found decreasing age-standardized burden estimates in all SDI categories, but the decreases were the most evident in middle- and high-middle–SDI regions (eFigure 10 and eTables 15 and 16 in Supplement 1).

### SAH Burden Compared With Other Causes

**Figure 3.**
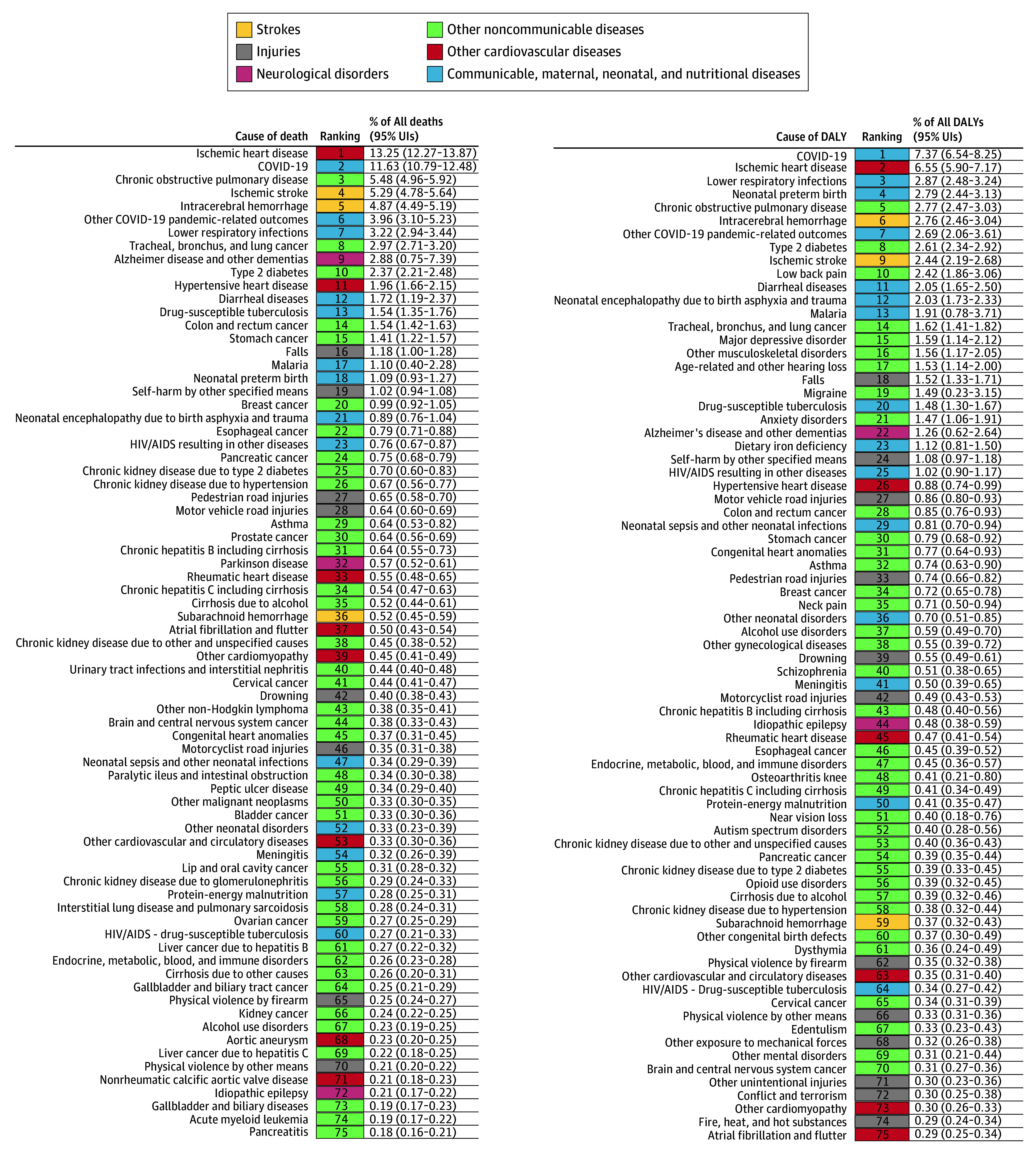
The Most Common Causes of Death and Disability-Adjusted Life-Years (DALYs) in the World in 2021 Rankings of the 75 most common causes of death and DALYs in the world in 2021 presented as percentages with 95% uncertainty intervals.

Of 300 level 4 diseases/injuries modeled by the GBD 2021 study, SAH ranked as the 36th most common cause of death (0.5%; 95% UI, 0.5%-0.6% of all deaths) and 59th most common cause of DALY (0.4%; 95% UI, 0.3%-0.4% of all DALYs) in the world (Figure 3). The corresponding rankings were 23rd and 39th among 192 noncommunicable diseases, 6th and 6th among 18 cardiovascular diseases, and 5th and 6th among 11 neurological disorders including strokes (eTable 17 in Supplement 1). We observed the highest rankings and proportional burdens of SAH in many middle-SDI regions such as Latin America and East Asia but also in the high-income Asian Pacific. The lowest rankings and proportional burdens occurred in low-SDI regions, especially in Sub-Saharan Africa (eTables 18 in Supplement 1).

### Risk Factors Attributed to SAH Burden

Of all worldwide SAH-related DALYs in 2021, 71.6% (95% UI, 63.8%-78.6%) attributed to the 14 modeled risk factors by the GBD study (Table 2). By the 3 level 1 risk clusters, metabolic risks accounted for the most risk-attributed DALYs of SAH, followed by the environmental/occupational risks and behavioral risks (Table 2 and eFigure 6 in Supplement 1). The top 3 individual risk factors were high SBP (PAF = 51.6%; 95% UI, 38.0%-62.6%), smoking (PAF = 14.4%; 95% UI, 12.4%-16.5%), and ambient particulate matter pollution (PAF = 14.2%; 95% UI, 9.8%-18.0%). The rankings varied slightly between male and female individuals (eFigure 7 in Supplement 1). By SDI levels, the attribution of all risk factors combined to SAH-related DALYs was highest in the low-SDI level (PAF = 77.3%; 95% UI, 70.2%-82.7%) and the lowest in the high-SDI level (PAF = 64.3%; 95% UI, 52.3%-74.0%). This was mainly attributed to the increased proportion of environmental/occupational risks and especially the increase in the association of household air pollution, which was attributed to 35.8% (95% UI, 28.5%-42.9%) of the SAH-related DALYs in the low-SDI level and less than 0.1% (95% UI, 0-0.3%) of the SAH-related DALYs in the high-SDI level (eFigures 6-8 in Supplement 1). Between 1990 and 2021, the age-standardized DALY rate of SAH that attributed to all risk factors combined decreased by 56.6% (95% UI, 44.7%-63.7%), and this decrease was more evident in environmental/occupational and behavioral risks than in metabolic risks (Table 2).

**Table 2.** Age-Standardized Proportions, Absolute Numbers, and Age-Standardized Rates of Subarachnoid Hemorrhage (SAH)–Related Disability-Adjusted Life-Years (DALYs) Attributed to Risk Factors in 2021 and Their Changes Between 1990-2021

Risk factors	Age-standardized proportion of SAH-related DALYs attributed to risk factors		Absolute number of SAH-related DALYs attributed to risk factors		Age-standardized SAH-related DALY rate per 100 000 people attributed to risk factors	
	In 2021, PAF (95% UI)	Change between 1990 and 2021, % (95% UI)	In 2021, No. in millions (95% UI)	Change between 1990 and 2021, % (95% UI)	In 2021, rate per 100 000 (95% UI)	Change between 1990 and 2021, % (95% UI)
All risk factors	71.54 (63.17 to 76.16)	−4.31 (−9.28 to −0.69)	7.72 (6.52 to 9.11)	−10.97 (−25.53 to 12.05)	89.60 (75.68 to 105.80)	−56.59 (−63.73 to −44.65)
Behavioral risks	28.69 (19.40 to 38.78)	−21.44 (−32.59 to −12.40)	3.11 (2.08 to 4.32)	−28.15 (−43.50 to −5.46)	35.96 (23.98 to 49.92)	−64.42 (−71.68 to −52.92)
Diet high in red meat	−7.03 (−29.02 to 9.97)	11.16 (−7.28 to 65.75)	−0.75 (−3.10 to 1.06)	−3.20 (−22.94 to 41.12)	−8.77 (−36.12 to 12.25)	−49.39 (−60.41 to −25.46)
Diet high in sodium	8.93 (2.00 to 19.81)	−26.72 (−55.54 to −10.93)	0.98 (0.22 to 2.24)	−30.57 (−60.70 to 3.40)	11.19 (2.54 to 25.86)	−66.82 (−81.00 to −51.07)
Diet low in fiber	4.01 (−1.20 to 8.35)	−17.91 (−23.83 to −10.94)	0.43 (−0.13 to 0.91)	−29.03 (−39.30 to −13.66)	5.03 (−1.50 to 10.68)	−62.79 (−68.34 to −54.13)
Diet low in fruits	9.01 (−0.67 to 16.39)	−6.80 (−10.25 to −2.27)	0.97 (−0.07 to 1.82)	−17.90 (−28.77 to −0.63)	11.29 (−0.80 to 21.15)	−57.75 (−63.42 to −48.31)
Diet low in vegetables	1.44 (−0.16 to 2.99)	−12.35 (−27.08 to 0.82)	0.16 (−0.02 to 0.33)	−24.39 (−36.82 to −10.52)	1.82 (−0.19 to 3.81)	−60.01 (−66.55 to −52.45)
Secondhand smoke	4.66 (3.20 to 6.15)	−21.57 (−29.18 to −14.28)	0.51 (0.34 to 0.67)	−29.33 (−40.62 to −10.15)	5.84 (3.91 to 7.85)	−64.39 (−70.47 to −54.14)
Smoking	14.43 (12.36 to 16.45)	−24.05 (−33.60 to −6.70)	1.57 (1.28 to 1.90)	−31.23 (−44.19 to −4.46)	18.07 (14.72 to 21.86)	−65.67 (−72.32 to −51.79)
Environmental/ occupational risks	32.73 (25.80 to 39.46)	−24.89 (−31.47 to −17.71)	3.53 (2.64 to 4.59)	−30.29 (−43.65 to −5.99)	41.05 (30.76 to 53.24)	−65.95 (−72.61 to −53.59)
Ambient particulate matter pollution	14.20 (9.82 to 17.97)	44.32 (9.41 to 92.37)	1.53 (1.03 to 1.97)	34.74 (−1.61 to 87.79)	17.77 (11.98 to 22.81)	−34.50 (−52.60 to −8.18)
High temperature	1.13 (0.21 to 2.46)	79.32 (−116.49 to 446.78)	0.12 (0.02 to 0.27)	52.84 (−49.56 to 310.51)	1.43 (0.27 to 3.14)	−18.67 (−107.51 to 159.93)
Household air pollution from solid fuels	10.29 (5.50 to 17.36)	−59.68 (−75.02 to −40.14)	1.12 (0.58 to 1.96)	−62.11 (−77.49 to −40.99)	12.96 (6.67 to 22.68)	−81.68 (−89.17 to −71.32)
Lead exposure	6.18 (−0.81 to 13.75)	−14.54 (−19.14 to −7.03)	0.67 (−0.09 to 1.49)	−20.21 (−34.56 to 6.26)	7.76 (−1.03 to 17.25)	−61.32 (−68.44 to −48.24)
Low temperature	4.48 (3.76 to 5.27)	−27.51 (−32.60 to −22.58)	0.48 (0.39 to 0.58)	−34.82 (−46.98 to −13.73)	5.60 (4.62 to 6.77)	−67.19 (−73.42 to −56.35)
Metabolic risks	52.54 (38.93 to 63.50)	12.43 (5.88 to 20.57)	5.68 (4.15 to 7.12)	6.67 (−11.02 to 32.31)	65.78 (48.06 to 82.57)	−48.93 (−57.56 to −36.01)
High body mass index	4.92 (0.00 to 10.99)	233.52 (−671.23 to 1668.40)	0.53 (0.00 to 1.19)	199.02 (−624.52 to 1533.99)	6.14 (0.00 to 13.85)	53.22 (−346.64 to 686.75)
High systolic blood pressure	51.57 (37.95 to 62.61)	10.96 (4.62 to 18.43)	5.58 (4.05 to 7.06)	5.35 (−12.47 to 30.77)	64.58 (46.84 to 81.79)	−49.59 (−58.30 to −37.25)

Abbreviations: PAF, population attributable fraction; UI, uncertainty interval.

## Discussion

According to the GBD 2021 study estimates, in 2021, there were 700 000 new SAH cases, almost 8 million patients with prevalent SAH, 350 000 SAH deaths, and over 10 million SAH-related DALYs globally. This ranked SAH as the 36th most common cause of death and 59th most common cause of death and disability in the world among 300 critical health outcomes. Although the global age-standardized mortality and DALY rates of SAH more than halved during the last 3 decades, the absolute number of patients with prevalent SAH increased by over 60% during the same period. This increase reached 105% in low-SDI levels where the absolute number of SAH-related deaths and disabilities also increased by more than 50%. Although these increases may be partially explained by improved diagnostic and documentation of SAH cases, some of these increases can be attributed to increased aging and population growth offering opportunities to reduce globally increasing SAH-related health consequences through improved prevention strategies. Without such urgent, international, and interdisciplinary actions, we can expect the absolute burden of SAH to continue to increase, particularly in low-SDI regions.

Observations about decreasing incidence and mortality rates of SAH are consistent with previous systematic reviews and pooled analyses of individual population-based cohort studies around the world.^4,5,6^ Such favorable trends have mainly been related to the decreasing prevalence of smoking,^5,17^ improved management of hypertension,^5^ improved management of SAH (eg, shortened treatment delays, increasing access to neurointensive care units, and evolvements of endovascular aneurysm treatments),^18^ as well as improvements in the pre-SAH interventions of persons with unruptured intracranial aneurysms (UIAs).^5,19^ Notably, most of these findings originate from high-SDI regions, whereas low- and middle-SDI areas continue to face significant challenges in accessing and delivering quality health care services for patients with SAH. For example, approximately 90% of the countries in GBD regions such as Sub-Saharan Africa, East Asia, and Oceania have been reported to lack urgent access to advanced microneurosurgery.^20^ In comparison with population-adjusted figures, previous literature on trends of absolute SAH cases, deaths, and disabilities is limited yet such estimates act as a cornerstone of worldwide public health planning and resource allocation. According to a recent policy view by the World Stroke Organization,^21^ the absolute burden of SAH was predicted to increase by over 40% by 2050, which was more than the corresponding estimates of other stroke types. However, although the policy view^21^ introduced extensive pragmatic solutions to reduce the global burden of stroke overall by improving its surveillance, prevention, acute care, and rehabilitation, the recommendations did not consider any SAH-specific characteristics. For instance, lower median age, female predisposition, distinct etiologies, high sudden-death rates, and unique neurosurgical treatment modalities of SAH as well as prevention possibilities of persons with UIAs were not assessed. Similarly, other key prevention guidelines on stroke, cardiovascular diseases, and neurological disorders have clustered all stroke types disregarding the distinct features of SAH,^22,23,24,25^ whereas the SAH-specific guidelines have focused on in-hospital diagnostics and management rather than prevention strategies of SAH.^3,26,27^ Given the distinct characteristics of SAH, its substantial proportion of global health burden, and large attribution to modifiable risk factors, guidelines on SAH-specific primary, secondary, and tertiary prevention that also consider the patients and high-risk individuals outside of the neurosurgical tertiary care are warranted in the future.

Besides surpassing the burden of most cardiovascular diseases (eg, atrial fibrillation/flutter, aortic aneurysms, heart valve diseases, peripheral arterial diseases, endocarditis, and myocarditis) and neurological disorders (eg, Parkinson disease, tension-type headache, motor neuron diseases, and multiple sclerosis), the global number of SAH deaths and disabilities also exceeded various other life-threatening infections (eg, encephalitis, hepatitis, and deaths due to meningitis), cancers (eg, brain, prostate and cervical cancers, leukemias and lymphomas), and injuries (eg, physical violence by firearms and sharp objects, sexual violence and nature disasters). Given that most of these causes in GBD studies are combinations of various 3-character *ICD* health conditions, our findings suggest that SAH is not only a common but also one of the most common cardiovascular and neurological causes of death and disabilities. Similarly high proportional burdens have also been reported in previous studies,^28,29^ which is understandable given the relatively low median age of SAH combined with high sudden-death, short-term case-fatality, and morbidity rates. For example, according to a previous nationwide study from Finland,^28^ aneurysmal SAH represented the 18th most common cause of death in middle-aged people (40-64 years old). By similar age stratification in the GBD 2021 study, consistent findings occurred not only in Finland but also in other Nordic and Western European countries with universal health care, and registration structures emphasizing the substantial role of SAH among the most common causes of premature mortality in working-age individuals (eFigure 9 in Supplement 1).

In line with various population-based cohort studies reporting not only associative^30,31,32,33^ but also causal relationships,^34,35,36,37^ high SBP and smoking were the 2 leading risk factors with the largest attribution to the burden of SAH. Because our findings suggest that eliminating these 2 risk factors would more than halve the burden of SAH, their prioritization is justified when placing prevention strategies for SAH. For example, advertisement bans, age restrictions, increased taxation, health education, cessation support, and prohibition of smoking in public places, and work environments including the hospitality industry represent evidence-based strategies that reduce smoking initiation and prevalence in populations.^38^ Similarly, improvements in diagnostics, medications, and lifestyle interventions on weight, salt intake and diet, especially among people with a high risk of hypertension, are known to decrease the disease burden of high blood pressure.^39^ In addition, the GBD 2021 estimates suggest that ambient and household air pollution have a comparable attribution to SAH burden with smoking which further highlights the importance of system-level disease prevention through policy changes rather than placing all burden on the individuals. Having said that, previous studies on the associations of air pollution and stroke risk have often clustered SAH with other stroke types and do not consider the potential confounding/mediating effects of other concurrent SAH risk factors.^37,40,41,42,43,44^ Similar limitations occur in the evidence of many dietary factors,^45^ including moderate to high consumption of alcohol.^31,32,37,46^ Regarding high BMI, previous evidence suggests that after considering the indirect effects via smoking and hypertension, the independent role of BMI on SAH risk is negligible^47^ but it may be associated with poor SAH outcomes.^48^ As additional potential risk factors that were assessed for stroke in general but not for SAH in the GBD 2021 study, low physical activity^34,37,49,50,51^ and adverse lipid profile^52,53^ have also been associated with a higher risk of SAH whereas the independent effect of high blood glucose and diabetes on SAH risk is more controversial.^32,34,54,55^

### Limitations

Even though the present study constitutes, to our knowledge, the most comprehensive analysis of the global, regional, and national burden estimates of SAH and their time trends, attributions to modifiable risk factors, and comparisons to other critical health outcomes, it also has limitations. First, due to the limited amount of SAH-specific data sources from various individual countries and population groups, many reported burden and risk factor estimates rely more on predictive covariates and assumptions of geographical similarities than actual high-quality observations. Given that the incidence and mortality estimates of SAH vary substantially both between^4,5,6^ and within countries,^56,57,58^ findings from population groups with limited or no actual data sources (eg, many individual countries) should be interpreted with caution and against other available evidence due to the increased risk of systematic selection, measurement, and detection biases. For example, of all 2910 SAH-specific data sources in the GBD 2021 study, only 22 originated from Sub-Saharan Africa including door-to-door prevalence surveys from Benin,^59^ Ghana,^60^ and Nigeria,^61^ admission data from a rural Nigerian hospital,^62^ and administrative cause-of-death registrations from Cape Verde, Ghana, South Africa, and Zimbabwe. Based on such scattered and sporadic data collections that invariably miss, eg, sudden-death SAHs without routinely conducted postmortem examinations, it seems probable that the GBD 2021 study underestimates the burden of SAH in many low-SDI regions from Sub-Saharan Africa. This lack of high-quality input data may also explain why female predisposition was not observed outside of high-SDI regions despite the consistent evidence from previous population-based studies.^5,32,63,64^ Nevertheless, our findings about the exceptionally low burden of SAH in many low-SDI regions rather emphasize the importance of high-quality disease surveillance than support a favorable situation in these regions. Second, even though the GBD 2021 study uses numerous data sources across the world, the data from several relevant publications especially focusing on SAH risk factors have not been incorporated as part of its prediction models. In fact, all SAH-specific risk factor data sources of the GBD 2021 study focus on dietary risks or lead exposure, whereas the relative risk estimates of other exposures are based on the stroke literature in general. Therefore, the reliability of covariate-driven risk factor estimations of the GBD study could likely be improved by performing an updated systematic review gathering the most recent and relevant published risk factor data for SAH.^37^ This would also enable the assessment of independent pathways and interactions of relevant SAH risk factors more comprehensively. Third, because the GBD 2021 study did not record the different etiologies of nontraumatic SAHs and most data sources were based on registration codes without external case validation, our findings should be applied cautiously to aneurysmal SAHs. Fourth, because the current GBD dataset did not include information about the regional and temporal changes in SAH diagnostics and treatment, future studies are still needed to determine the exact reasons for our epidemiological observations and establish pragmatic solutions for decreasing the global burden of SAH. For example, high-quality comparisons focusing on worldwide and temporal differences in prehospital, in-hospital, and posthospital care of SAH would be of great importance. Lastly, the current GBD data release included a limited number of data sources for the most recent years, particularly after the COVID-19 pandemic. Although no significant changes in the SAH burden were observed during the peak pandemic years of 2020 and 2021, future data releases may offer more comprehensive insights into the potential effects of COVID-19 and related shifts in health care systems on the global burden of SAH.

## Conclusions

Despite decreasing age-standardized burden rates, SAH remains one of the most common cardiovascular and neurological causes of death and disability globally with constantly increasing absolute case numbers. Moreover, over 70% of the SAH-related burden appears to be attributed to modifiable risk factors, most importantly to high systolic blood pressure and smoking. Given the substantial and potentially preventable impact of SAH on global health, consideration of its distinct features from other stroke types in evidence-based public health planning, resource allocation, and prevention strategies is warranted. Besides efforts to decrease global hypertension and smoking rates, enhancing in-hospital patient care, the availability of diagnostic tools, neurosurgical tertiary care, and identification of UIAs among patients with a high SAH risk could serve as justified targets for future improvements, especially in low and middle SDI regions. At the same time, many countries, especially from Sub-Saharan Africa, do not have any SAH-specific data sources; this emphasizes the importance of international and interdisciplinary collaboration to produce more reliable burden estimates of SAH from these regions.

## Notes

Supplement 1. **eAppendix.** Contributions of Authors
eMethods.

eReferences.
**eFigure 1.** The Numbers and Distributions of Data Sources for Subarachnoid Hemorrhage Included in the Global Burden of Diseases 2021 Study by Year, Type, and Region **eFigure 2.** The Numbers and Distributions of Data Sources for Fatal Subarachnoid Hemorrhage Included in the Global Burden of Diseases 2021 Study by Year and Region **eFigure 3.** The Numbers and Distributions of Data Sources for Nonfatal Subarachnoid Hemorrhage Included in the Global Burden of Diseases 2021 Study by Year, Type, and Region **eFigure 4.** The Numbers and Distributions of Data Sources for Subarachnoid Hemorrhage Risk Factors Included in the Global Burden of Diseases 2021 Study by Year, Region, and Risk Factor **eFigure 5.** Incidence, Prevalence, Mortality, and Disability-Adjusted Life-Years Rates of Subarachnoid Hemorrhage per 100,000 People by Age and Separately for Men and Women **eFigure 6.** Rankings of Risk Factors by Their Age-Standardized Population-Attributable Fractions on Subarachnoid Hemorrhage-Related Disability-Adjusted Life-Years in the World, 5 Sociodemographic Index Levels, 7 GBD Super Regions, and 21 GBD Regions **eFigure 7.** Rankings of Risk Factors by Their Age-Standardized Population-Attributable Fractions on SAH-Related Disability-Adjusted Life-Years in the World, by 5 Sociodemographic Index Levels, 7 GBD Super Regions, and 21 GBD Regions, Separately for Men and Women **eFigure 8.** Age-Standardized Population-Attributable Fractions of Environmental/Occupational Risks, Behavioral Risks, and Metabolic Risks on Disability-Adjusted Life-Years Related to Subarachnoid Hemorrhage in 204 Countries and Territories of the World in 2021 **eFigure 9.** Rankings of Causes of Death in the Age Group of 20-54 Years Across 24 Western European Countries in 2021 **eFigure 10.** Changes in the Incidence, Prevalence, Deaths, and Disability-Adjusted Life-Years of Subarachnoid Hemorrhage in the World Between 1990 (Deaths Since 1980) and 2021 **eTable 1.** Selected Covariates for SAH-Specific Modeling in the GBD 2021 Study **eTable 2.** Fourteen Individual Risk Factors for Subarachnoid Hemorrhage in the Global Burden of Disease 2021 Study **eTable 3.** Key Variables/Terms Used in the Current Manuscript Regarding the Global Burden of SAH **eTable 4.** Age-Standardized Incidence, Prevalence, Mortality, and Disability-Adjusted Life-Years Rates (With 95% Uncertainty Intervals) of Subarachnoid Hemorrhage per 100,000 People for 204 Countries and Territories in 2021 **eTable 5.** Age-Standardized Incidence, Prevalence, Mortality, and Disability-Adjusted Life-Year Rates of Subarachnoid Hemorrhage per 100,000 People in 2021 by 5 Sociodemographic Index Levels, 7 Global Burden of Disease Super Regions, and 21 Global Burden of Disease Regions **eTable 6.** Age-Standardized Incidence, Prevalence, Mortality, and DALY Rates (With 95% Uncertainty Intervals) of SAH per 100,000 People in 2021 for Men by 5 Country-Specific Sociodemographic Index Levels, 7 GBD Super Regions, and 21 GBD Regions **eTable 7.** Age-Standardized Incidence, Prevalence, Mortality, and DALY Rates (With 95% Uncertainty Intervals) of SAH per 100,000 People in 2021 for Women by 5 Country-Specific Sociodemographic Index Levels, 7 GBD Super Regions, and 21 GBD Regions **eTable 8.** Incidence, Prevalence, Mortality, and DALY Rates (With 95% Uncertainty Intervals) of SAH per 100,000 People in 2021 for Children (0-14 Years of Age) by 5 Country-Specific Sociodemographic Index Levels, 7 GBD Super Regions, and 21 GBD Regions **eTable 9.** Incidence, Prevalence, Mortality, and DALY Rates (With 95% Uncertainty Intervals) of SAH per 100,000 People in 2021 for Young Adults (15-49 Years of Age) by 5 Country-Specific Sociodemographic Index Levels, 7 GBD Super Regions, and 21 GBD Regions **eTable 10.** Incidence, Prevalence, Mortality, and DALY Rates (With 95% Uncertainty Intervals) of SAH per 100,000 People in 2021 for Old Adults (50-74 Years of Age) by 5 Country-Specific Sociodemographic Index Levels, 7 GBD Super Regions, and 21 GBD Regions **eTable 11.** Incidence, Prevalence, Mortality, and DALY Rates (With 95% Uncertainty Intervals) of SAH per 100,000 People in 2021 for Very Old Adults (≥75 Years of Age) by 5 Country-Specific Sociodemographic Index Levels, 7 GBD Super Regions, and 21 GBD Regions **eTable 12.** Percentage Changes (With 95% Uncertainty Intervals) of the Incidence, Prevalence, Mortality, and DALYs of Subarachnoid Hemorrhage in the World Between 1990 and 2021, Separately for Men, Women, and Different Age Groups **eTable 13.** Percentage Changes (With 95% Uncertainty Intervals) in the Absolute Number of SAH Incidents, Prevalent Cases, Deaths, and DALYs Between 1990 and 2021 by 5 Country-Specific Sociodemographic Index Levels, 7 GBD Super Regions, and 21 GBD Regions **eTable 14.** Percentage Changes (With 95% Uncertainty Intervals) in the Absolute Number of SAH Incidents, Prevalent Cases, Deaths, and DALYs in 204 Countries and Territories of the World Between 1990 and 2021 **eTable 15.** Percentage Changes (With 95% Uncertainty Intervals) in the Age-Standardized Incidence, Prevalence, Mortality, and DALY Rates of SAH per 100,000 People Between 1990 and 2021 by 5 Country-Specific Sociodemographic Index Levels, 7 GBD Super Regions, and 21 GBD Regions **eTable 16.** Percentage Changes (With 95% Uncertainty Intervals) in the Age-Standardized Incidence, Prevalence, Mortality, and DALY Rates of SAH per 100,000 People in 204 Countries and Territories of the World Between 1990 and 2021 **eTable 17.** Rankings of Subarachnoid Hemorrhage by the Number of Global Deaths and Disability-Adjusted Life-Years in 1990 and 2021 Among All Diseases, Noncommunicable Diseases, Cardiovascular Diseases, Neurological Disorders, and Strokes **eTable 18.** Regional Rankings and Proportions of Subarachnoid Hemorrhage-Related Deaths and Disability-Adjusted Life-Years in 2021 Among All 300 Diseases/Injuries by 5 Country-Specific Sociodemographic Index Levels, 7 GBD Super Regions, and 21 GBD Regions

Supplement 2.
Data Sharing Statement.
