# Supplementary material for: Global, Regional, and National Burden of Nontraumatic Subarachnoid Hemorrhage: The Global Burden of Disease Study 2021
Source: JAMA Neurol. 2025 May 23;82(8):765–87. doi: 10.1001/jamaneurol.2025.1522 (PMC12557468; doi:10.1001/jamaneurol.2025.1522)
Supplement: Supplement 1. — eAppendix. Contributions of Authors eMethods. eReferences. eFigure 1. The Numbers and Distributions of Data Sources for Subarachnoid Hemorrhage Included in the Global Burden of Diseases 2021 Study by Year, Type, and Region eFigure 2. The Numbers and Distributions of Data Sources for Fatal Subarachnoid Hemorrhage Included in the Global Burden of Diseases 2021 Study by Year and Region eFigure 3. The Numbers and Distributions of Data Sources for Nonfatal Subarachnoid Hemorrhage Included in the Global Burden of Diseases 2021 Study by Year, Type, and Region eFigure 4. The Numbers and Distributions of Data Sources for Subarachnoid Hemorrhage Risk Factors Included in the Global Burden of Diseases 2021 Study by Year, Region, and Risk Factor eFigure 5. Incidence, Prevalence, Mortality, and Disability-Adjusted Life-Years Rates of Subarachnoid Hemorrhage per 100,000 People by Age and Separately for Men and Women eFigure 6. Rankings of Risk Factors by Their Age-Standardized Population-Attributable Fractions on Subarachnoid Hemorrhage-Related Disability-Adjusted Life-Years in the World, 5 Sociodemographic Index Levels, 7 GBD Super Regions, and 21 GBD Regions eFigure 7. Rankings of Risk Factors by Their Age-Standardized Population-Attributable Fractions on SAH-Related Disability-Adjusted Life-Years in the World, by 5 Sociodemographic Index Levels, 7 GBD Super Regions, and 21 GBD Regions, Separately for Men and Women eFigure 8. Age-Standardized Population-Attributable Fractions of Environmental/Occupational Risks, Behavioral Risks, and Metabolic Risks on Disability-Adjusted Life-Years Related to Subarachnoid Hemorrhage in 204 Countries and Territories of the World in 2021 eFigure 9. Rankings of Causes of Death in the Age Group of 20-54 Years Across 24 Western European Countries in 2021 eFigure 10. Changes in the Incidence, Prevalence, Deaths, and Disability-Adjusted Life-Years of Subarachnoid Hemorrhage in the World Between 1990 (Deaths Since 1980) and 2021 eTable 1. Selected Cova [file jamaneurol-e251522-s001.pdf]

Supplementary Online Content

GBD 2021 Global Subarachnoid Hemorrhage Risk Factors Collaborators. Global, regional, and national burden of nontraumatic subarachnoid hemorrhage: the Global Burden of Disease Study 2021. *JAMA Neurol*. Published online May 23, 2025. doi:10.1001/jamaneurol.2025.1522

eAppendix. Contributions of Authors

eMethods.

eReferences.

eFigure 1. The Numbers and Distributions of Data Sources for Subarachnoid Hemorrhage Included in the Global Burden of Diseases 2021 Study by Year, Type, and Region

eFigure 2. The Numbers and Distributions of Data Sources for Fatal Subarachnoid Hemorrhage Included in the Global Burden of Diseases 2021 Study by Year and Region

eFigure 3. The Numbers and Distributions of Data Sources for Nonfatal Subarachnoid Hemorrhage Included in the Global Burden of Diseases 2021 Study by Year, Type, and Region

eFigure 4. The Numbers and Distributions of Data Sources for Subarachnoid Hemorrhage Risk Factors Included in the Global Burden of Diseases 2021 Study by Year, Region, and Risk Factor

eFigure 5. Incidence, Prevalence, Mortality, and Disability-Adjusted Life-Years Rates of Subarachnoid Hemorrhage per 100,000 People by Age and Separately for Men and Women

eFigure 6. Rankings of Risk Factors by Their Age-Standardized Population-Attributable Fractions on Subarachnoid Hemorrhage-Related Disability-Adjusted Life-Years in the World, 5 Sociodemographic Index Levels, 7 GBD Super Regions, and 21 GBD Regions

eFigure 7. Rankings of Risk Factors by Their Age-Standardized Population-Attributable Fractions on SAH-Related Disability-Adjusted Life-Years in the World, by 5 Sociodemographic Index Levels, 7 GBD Super Regions, and 21 GBD Regions, Separately for Men and Women

eFigure 8. Age-Standardized Population-Attributable Fractions of Environmental/Occupational Risks, Behavioral Risks, and Metabolic Risks on Disability-Adjusted Life-Years Related to Subarachnoid Hemorrhage in 204 Countries and Territories of the World in 2021

eFigure 9. Rankings of Causes of Death in the Age Group of 20-54 Years Across 24 Western European Countries in 2021

© 2025 GBD 2021 Global Subarachnoid Hemorrhage Risk Factors Collaborators. *JAMA Neurology*.

eFigure 10. Changes in the Incidence, Prevalence, Deaths, and Disability-Adjusted Life-Years of Subarachnoid Hemorrhage in the World Between 1990 (Deaths Since 1980) and 2021

eTable 1. Selected Covariates for SAH-Specific Modeling in the GBD 2021 Study

eTable 2. Fourteen Individual Risk Factors for Subarachnoid Hemorrhage in the Global Burden of Disease 2021 Study

eTable 3. Key Variables/Terms Used in the Current Manuscript Regarding the Global Burden of SAH

eTable 4. Age-Standardized Incidence, Prevalence, Mortality, and Disability-Adjusted Life-Years Rates (With 95% Uncertainty Intervals) of Subarachnoid Hemorrhage per 100,000 People for 204 Countries and Territories in 2021

eTable 5. Age-Standardized Incidence, Prevalence, Mortality, and Disability-Adjusted Life-Year Rates of Subarachnoid Hemorrhage per 100,000 People in 2021 by 5 Sociodemographic Index Levels, 7 Global Burden of Disease Super Regions, and 21 Global Burden of Disease Regions

eTable 6. Age-Standardized Incidence, Prevalence, Mortality, and DALY Rates (With 95% Uncertainty Intervals) of SAH per 100,000 People in 2021 for Men by 5 Country-Specific Sociodemographic Index Levels, 7 GBD Super Regions, and 21 GBD Regions

eTable 7. Age-Standardized Incidence, Prevalence, Mortality, and DALY Rates (With 95% Uncertainty Intervals) of SAH per 100,000 People in 2021 for Women by 5 Country-Specific Sociodemographic Index Levels, 7 GBD Super Regions, and 21 GBD Regions

eTable 8. Incidence, Prevalence, Mortality, and DALY Rates (With 95% Uncertainty Intervals) of SAH per 100,000 People in 2021 for Children (0-14 Years of Age) by 5 Country-Specific Sociodemographic Index Levels, 7 GBD Super Regions, and 21 GBD Regions

eTable 9. Incidence, Prevalence, Mortality, and DALY Rates (With 95% Uncertainty Intervals) of SAH per 100,000 People in 2021 for Young Adults (15-49 Years of Age) by 5 Country-Specific Sociodemographic Index Levels, 7 GBD Super Regions, and 21 GBD Regions

eTable 10. Incidence, Prevalence, Mortality, and DALY Rates (With 95% Uncertainty Intervals) of SAH per 100,000 People in 2021 for Old Adults (50-74 Years of Age) by 5 Country-Specific Sociodemographic Index Levels, 7 GBD Super Regions, and 21 GBD Regions

eTable 11. Incidence, Prevalence, Mortality, and DALY Rates (With 95% Uncertainty Intervals) of SAH per 100,000 People in 2021 for Very Old Adults (≥75 Years of Age) by 5 Country-Specific Sociodemographic Index Levels, 7 GBD Super Regions, and 21 GBD Regions

© 2025 GBD 2021 Global Subarachnoid Hemorrhage Risk Factors Collaborators. *JAMA Neurology*.

eTable 12. Percentage Changes (With 95% Uncertainty Intervals) of the Incidence, Prevalence, Mortality, and DALYs of Subarachnoid Hemorrhage in the World Between 1990 and 2021, Separately for Men, Women, and Different Age Groups

eTable 13. Percentage Changes (With 95% Uncertainty Intervals) in the Absolute Number of SAH Incidents, Prevalent Cases, Deaths, and DALYs Between 1990 and 2021 by 5 Country-Specific Sociodemographic Index Levels, 7 GBD Super Regions, and 21 GBD Regions

eTable 14. Percentage Changes (With 95% Uncertainty Intervals) in the Absolute Number of SAH Incidents, Prevalent Cases, Deaths, and DALYs in 204 Countries and Territories of the World Between 1990 and 2021

eTable 15. Percentage Changes (With 95% Uncertainty Intervals) in the Age-Standardized Incidence, Prevalence, Mortality, and DALY Rates of SAH per 100,000 People Between 1990 and 2021 by 5 Country-Specific Sociodemographic Index Levels, 7 GBD Super Regions, and 21 GBD Regions

eTable 16. Percentage Changes (With 95% Uncertainty Intervals) in the Age-Standardized Incidence, Prevalence, Mortality, and DALY Rates of SAH per 100,000 People in 204 Countries and Territories of the World Between 1990 and 2021

eTable 17. Rankings of Subarachnoid Hemorrhage by the Number of Global Deaths and Disability-Adjusted Life-Years in 1990 and 2021 Among All Diseases, Noncommunicable Diseases, Cardiovascular Diseases, Neurological Disorders, and Strokes

eTable 18. Regional Rankings and Proportions of Subarachnoid Hemorrhage-Related Deaths and Disability-Adjusted Life-Years in 2021 Among All 300 Diseases/Injuries by 5 Country-Specific Sociodemographic Index Levels, 7 GBD Super Regions, and 21 GBD Regions

This supplementary material has been provided by the authors to give readers additional information about their work.

© 2025 GBD 2021 Global Subarachnoid Hemorrhage Risk Factors Collaborators. *JAMA Neurology*.

eAppendix.

1. Contributions of Authors

Core/writing team

Ilari Rautalin, Victor Volovici, Jaakko Kaprio, Miikka Korja, Rita Krishnamurthi, Balakrishnan Nair, Anna Ranta, Gabriel Rinkel, Mervyn Vergouwen, Valery Feigin

First author: Ilari Rautalin
Second Author: Victor Volovici
Last/Senior Author: Valery L. Feigin

Providing data or critical feedback on data sources

Yohannes Habtegiorgis Abate, Hedayat Abbastabar, Parsa Abdi, Auwal Abdullahi, Olugbenga Olusola Abiodun, Richard Gyan Aboagye, Dariush Abtahi, Samir Abu Rumeileh, Ahmed Abualhasan, Ahmed Abu-Zaid, Oyelola A Adegboye, Leticia Akua Adzigbli, Bright Opoku Ahinkorah, Danish Ahmad, Ali Ahmed, Haroon Ahmed, Budi Aji, Yazan Al-Ajlouni, Mohammed Albashtawy, Sheikh Mohammad Alif, Syed Mohamed Aljunid, Sami Almustanyir, Awais Altaf, Alaa B. Al-Tammemi, Nelson Alvis-Guzman, Hassan Alwafi, Mohammad Al-Wardat, Hany Aly, Alireza Amindarolzarbi, Ganiyu Adeniyi Amusa, Deanna Anderlini, Boluwatife Stephen Anuoluwa, Saleha Anwar, Geminn Louis Carace Apostol, Jalal Arabloo, Johan Ärnlöv, Anton A Artamonov, Tahira Ashraf, Seyyed Shamsadin Athari, Ahmed Y. Azzam, Atif Amin Baig, Abdulaziz T Bako, Ovidiu Constantin Baltatu, Maciej Banach, Mainak Bardhan, Till Winfried Bärnighausen, Hiba Jawdat Barqawi, Mohammad-Mahdi Bastan, Sanjay Basu, Shelly L Bell, Akshaya Srikanth Bhagavathula, Sonu Bhaskar, Ajay Nagesh Bhat, Vivek Bhat, Gurjit Kaur Bhatti, Jasvinder Singh Singh Bhatti, Boris Bikbov, Archith Boloor, Hamed Borhany, Florentino Luciano Caetano dos Santos, Luis Alberto Cámera, Carlos A Castañeda-Orjuela, Joshua Chadwick, Gashaw Sisay Chanie, Vijay Kumar Chattu, Hitesh Chopra, Dinh-Toi Chu, Alyssa Columbus, Michael H Criqui, Xiaochen Dai, Ivan Delgado-Enciso, Emina Dervišević, Vinoth Gnana Chellaiyan Devanbu, Michael J Diaz, Thanh Chi Do, Thao Huynh Phuong Do, Mohamed Fahmy Doheim, Klara Georgieva Dokova, Ojas Prakashbhai Doshi, Rajkumar Prakashbhai Doshi, Abdel Douiri, Robert Kokou Dowou, Haneil Larson Dsouza, Abdel Rahman E'mar, Nevine El Nahas, Chadi Eltaha, Adeniyi Francis Fagbamigbe, Ildar Ravisovich Fakhradiyev, Jawad Fares, Valery L Feigin, Ginenus Fekadu, Avi A Gajjar, Balasankar Ganesan, Ravindra Kumar Garg, Teferi Gebru Gebremeskel, Molla Getie, Sulmaz Ghahramani, Sherief Ghozy, Jaleed Ahmed Gilani, Pouya Goleij, Alessandra C Goulart, Barbara Niegia Garcia Goulart, Shi-Yang Guan, Sapna Gupta, Nasrin Hanifi, Josep Maria Haro, Faizul Hasan, Mahgol Sadat Hassan Zadeh Tabatabaei, Johannes Haubold, Simon I Hay, Golnaz Heidari, Mehdi Hemmati, Nguyen Quoc Hoan, Mehdi Hosseinzadeh, Mowafa Househ, Hong-Han Huynh, Segun Emmanuel Ibitoye, Nayu Ikeda, Olayinka Stephen Ilesanmi, Nahlah Elkudssiah Ismail, Gaetano Isola, Ammar Abdulrahman Jairoun, Mihajlo Jakovljevic, Talha Jawaid, Sathish Kumar Jayapal, Jost B Jonas, Mikk Jürisson, Vidya Kadashetti, Himal Kandel, Faizan Zaffar Kashoo, Gbenga A Kayode, Yousef Saleh Khader, Haitham Khatatbeh, Feriha Fatima Khidri, Atulya Aman Khosla, Yun Jin Kim, Adnan Kisa, Sezer Kisa, Oleksii Korzh, Nikhil Kothari, Kewal Krishan, Vijay Krishnamoorthy, Maria Dyah Kurniasari, Dian Kusuma, Ville Kytö, Chandrakant Lahariya, Tri Laksono, Kamaluddin Latief, Kaveh Latifinaibin, Nhi Huu Hanh Le, Thao Thi Thu Le, Munjae Lee, Seung Won Lee, Stephen S Lim, Xuefeng Liu, László Lorenzovici, Paulo A Lotufo, Jay B Lusk, Kashish Malhotra, Deborah Carvalho Malta, Mohammad Ali Mansournia, Lorenzo Giovanni Mantovani, Seyed Farzad Maroufi, Roy Rillera Marzo, Andrea Maugeri, Man Mohan Mehndiratta, Ritesh G Menezes, Atte Meretoja, Le Huu Nhat Minh, Erkin M Mirrakhimov, Ajay Kumar Mishra, Prasanna Mithra, Ameen Mosa Mohammad, Soheil Mohammadi, Abdollah Mohammadian-Hafshejani, Shafiu Mohammed, Ali H Mokdad, Shaher Momani, Mohammad Ali Moni, Maryam Moradi, Yousef Moradi, Rohith Motappa, Ahmed Msherghi, Efren Murillo-Zamora, Christopher J L Murray, Ahamarshan Jayaraman Nagarajan, Ganesh R Naik, Balakrishnan Sukumaran Nair, Sreenivas Narasimha Swamy, Shumaila Nargus, Zuhair S Natto, Vinod C Nayak, Ruxandra Irina Negoi, Duc Hoang Nguyen, Hien Quang Nguyen, Phat Tuan Nguyen, Van Thanh Nguyen, Robina Khan Niazi, Antonio Tolentino Nogueira de Sá, Shuhei Nomura, Jean Jacques Noubiap, Fred Nugen, Chimezie Igwegbe Nzoputam, Bogdan Oancea, Michael Safo Oduro, Akinkunmi Paul Okekunle, Andrew T Olagunju, Abdulhakeem Abayomi Olorukooba, Uchechukwu Levi Osuagwu, Amel Ouyahia, Mayowa O Owolabi, Mahesh Padukudru P A, Jagadish Rao Padubidri, Demosthenes Panagiotakos, Romil R Parikh, Jay Patel, Urvish K Patel, Paolo Pedersini, Emmanuel K Peprah, Gavin Pereira, Simone Perna, Anil K Philip, Maarten J Postma, Jalandhar Pradhan, Manya Prasad, Jagadeesh Puvvula, Fakher Rahim, Vafa Rahimi-Movaghar, Amir Masoud Rahmani, Mohammad Rahmanian, Sathish Rajaa, Ali Rajabpour

© 2025 GBD 2021 Global Subarachnoid Hemorrhage Risk Factors Collaborators. *JAMA Neurology*.

Sanati, Mahmoud Mohammed Ramadan, Shakthi Kumaran Ramasamy, Sheena Ramazanu, Sina Rashedi, Ilari Rautalin, Salman Rawaf, Christian Razo, Jefferson Antonio Buendia Rodriguez, Leonardo Roever, Allen Guy Ross, Priyanka Roy, Aly M A Saad, Siamak Sabour, Basema Ahmad Saddik, Pragyan Monalisa Sahoo, Vijaya Paul Samuel, Abdallah M Samy, Milena M Santric-Milicevic, Maheswar Satpathy, Markus P Schlaich, Siddharthan Selvaraj, Subramanian Senthilkumaran, Yashendra Sethi, Allen Seylani, Ahmed Nabil Shaaban, Masood Ali Shaikh, Summaiya Zareen Shaikh, Muhammad Aaqib Shamim, Anas Shamsi, Mohammed Shannawaz, Amin Sharifan, Javad Sharifi Rad, Vishal Sharma, Aminu Shittu, Nathan A Shlobin, Baljinder Singh, Paramdeep Singh, Michael Spartalis, Jeffrey D Stanaway, Muhammad Haroon Stanikzai, Benjamin A Stark, Antonina V Starodubova, Chandan Kumar Swain, Lukasz Szarpak, Jabeen Taiba, Ker-Kan Tan, Roman Topor-Madry, Marcos Roberto Tovani-Palone, Jasmine T Tran, Thomas Clement Truelsen, Bhaskaran Unnikrishnan, Jibrin Sammani Usman, Jef Van den Eynde, Tommi Juhani Vasankari, Narayanaswamy Venketasubramanian, Sergey Konstantinovitch Vladimirov, Victor Volovici, Yasir Waheed, Waseem Wahood, Mandaras Tariku Walde, Charles D A Wolfe, Hong Xiao, Suowen Xu, Kazumasa Yamagishi, Yuichiro Yano, Sanni Yaya, Pengpeng Ye, Naohiro Yonemoto, Chuanhua Yu, Iman Zare, Michael Zastrozhin, Abzal Zhumagaliuly, and Magdalena Zielińska.

Developing methods or computational machinery

Ali Ahmed, Ahmed Y. Azzam, Hiba Jawdat Barqawi, Mohammad-Mahdi Bastan, Akshaya Srikanth Bhagavathula, Hamed Borhany, Hitesh Chopra, Dinh-Toi Chu, Xiaochen Dai, Thanh Chi Do, Adeniyi Francis Fagbamigbe, Ayesha Fahim, Valery L Feigin, Sherief Ghozy, Shi-Yang Guan, Nasrin Hanifi, Mahgol Sadat Hassan Zadeh Tabatabaei, Mohammad Heidari, Mehdi Hosseinzadeh, Hong-Han Huynh, Gaetano Isola, Talha Jawaid, Sathish Kumar Jayapal, Faizan Zaffar Kashoo, Atulya Aman Khosla, Adnan Kisa, Chandrakant Lahariya, Hanpeng Lai, Kamaluddin Latief, Nhi Huu Hanh Le, Thao Thi Thu Le, Le Huu Nhat Minh, Abdollah Mohammadian-Hafshejani, Ali H Mokdad, Mohammad Ali Moni, Yousef Moradi, Ahmed Msherghi, Christopher J L Murray, Balakrishnan Sukumaran Nair, Shumaila Nargus, Phat Tuan Nguyen, Van Thanh Nguyen, Amel Ouyahia, Amir Masoud Rahmani, Sina Rashedi, Christian Razo, Abdallah M Samy, Maheswar Satpathy, Austin E Schumacher, Yashendra Sethi, Mohammed Shannawaz, Javad Sharifi Rad, Michael Spartalis, Jeffrey D Stanaway, Muhammad Haroon Stanikzai, Benjamin A Stark, Chandan Kumar Swain, Jorge Hugo Villafañe, and Naohiro Yonemoto.

Providing critical feedback on methods or results

Yohannes Habtegiorgis Abate, Hedayat Abbastabar, Foad Abd-Allah, Atef Abdelkader, Parsa Abdi, Arash Abdollahi, Auwal Abdullahi, Olugbenga Olusola Abiodun, Richard Gyan Aboagye, Mohamed Abouzid, Samir Abu Rumeileh, Ahmed Abualhasan, Hasan Abualruz, Hana J Abukhadijah, Ahmed Abu-Zaid, Lawan Hassan Adamu, Isaac Yeboah Addo, Rufus Adesoji Adedoyin, Oyelola A Adegboye, Saryia Adra, Leticia Akua Adzigbli, Williams Agyemang-Duah, Bright Opoku Ahinkorah, Aqeel Ahmad, Danish Ahmad, Amir Mahmoud Ahmadzade, Ali Ahmed, Haroon Ahmed, Syed Anees Ahmed, Budi Aji, Mohammed Ahmed Akkaif, Yazan Al-Ajlouni, Ziyad Al-Aly, Mohammed Albashtawy, Mohammed Usman Ali, Sheikh Mohammad Alif, Yousef Alimohamadi, Syed Mohamed Aljunid, Sami Almustanyir, Mahmoud A Alomari, Mohammed A Alsabri, Rustam Al-Shahi Salman, Awais Altaf, Alaa B. Al-Tammemi, Nelson Alvis-Guzman, Hassan Alwafi, Mohammad Al-Wardat, Yaser Mohammed Al-Worafi, Hany Aly, Karem H Alzoubi, Reza Amani, Tarek Tawfik Amin, Alireza Amindarolzarbi, Ganiyu Adeniyi Amusa, Deanna Anderlini, Dhanalakshmi Angappan, Boluwatife Stephen Anuoluwa, Saleha Anwar, Anayochukwu Edward Anyasodor, Geminn Louis Carace Apostol, Jalal Arabloo, Demelash Areda, Anton A Artamonov, Kurnia Dwi Artanti, Ashokan Arumugam, Zahra Aryan, Mohammad Asghari-Jafarabadi, Mubarek Yesse Ashemo, Tahira Ashraf, Mohammad Athar, Seyyed Shamsadin Athari, Adedapo Wasiu Awotidebe, Sina Azadnajafabad, Shahkaar Aziz, Ahmed Y. Azzam, Giridhara Rathnaiah Babu, Nasser Bagheri, Pegah Bahrami Taghanaki, Saeed Bahramian, Ruhai Bai, Atif Amin Baig, Abdulaziz T Bako, Ovidiu Constantin Baltatu, Maciej Banach, Soham Bandyopadhyay, Biswajit Banik, Mainak Bardhan, Till Winfried Bärnighausen, Hiba Jawdat Barqawi, Lingkan Barua, Mohammad-Mahdi Bastan, Sanjay Basu, Shelly L Bell, Isabela M Bensenor, Alemshet Yirga Berhie, Akshaya Srikanth Bhagavathula, Sonu Bhaskar, Ajay Nagesh Bhat, Vivek Bhat, Gurjit Kaur Bhatti, Jasvinder Singh Singh Bhatti, Ali Bijani, Boris Bikbov, Mekuriaw Mesfin Birhan, Mulugeta M Birhanu, Veera R Bitra, Archith Boloor, Hamed Borhany, Hermann Brenner, Raffaele Bugiardini, Norma B Bulamu, Zahid A Butt, Lucas Scotta Cabral, Florentino Luciano Caetano dos Santos, Luis Alberto Cámera, Luciana Aparecida Campos, Ismael Campos-Nonato, Angelo Capodici, Carlos A Castañeda-Orjuela, Alberico L Catapano, Luca Cegolon, Joshua Chadwick, Chiranjib Chakraborty, Promit Ananyo Chakraborty, Rama Mohan Chandika, Gashaw Sisay Chanie, Vijay Kumar Chattu, Gerald Chi, Fatemeh Chichagi, Hitesh Chopra, Sonali Gajanan Choudhari, Enayet Karim Chowdhury, Dinh-Toi Chu, Sheng-Chia Chung, Alyssa Columbus, Michael H Criqui, Alanna Gomes da Silva, Mohammad Amin Dabbagh Ohadi, Omid Dadras, Xiaochen Dai, Koustuv Dalal, Lachlan L Dalli, Emanuele D'Amico, Mohsen Dashti, Kairat Davletov, Vanessa

© 2025 GBD 2021 Global Subarachnoid Hemorrhage Risk Factors Collaborators. *JAMA Neurology*.

De la Cruz-Góngora, Shayom Debopadhaya, Ivan Delgado-Enciso, Emina Dervišević, Vinoth Gnana Chellaiyan Devanbu, Syed Masudur Rahman Dewan, Amol S Dhane, Michael J Diaz, Mahmoud Dibas, Thanh Chi Do, Thao Huynh Phuong Do, Klara Georgieva Dokova, Deepa Dongarwar, Mario D'Oria, Ojas Prakashbhai Doshi, Rajkumar Prakashbhai Doshi, Abdel Douiri, Robert Kokou Dowou, Haneil Larson Dsouza, Siddhartha Dutta, Arkadiusz Marian Dziedzic, Abdel Rahman E'mar, David Edvardsson, Defi Efendi, Ferry Efendi, Nevine El Nahas, Islam Y Elgendy, Muhammed Elhadi, Chadi Eltaha, Mohd. Elmagzoub Eltahir, Theophilus I Emeto, Natalia Fabin, Adeniyi Francis Fagbamigbe, Ayesha Fahim, Ildar Ravisovich Fakhradiyev, Jawad Fares, Pawan Sirwan Faris, Timur Fazylov, Valery L Feigin, Ginenus Fekadu, Getahun Fetensa, Florian Fischer, Matteo Foschi, Ni Kadek Yuni Fridayani, Abduzhappar Gaipov, Avi A Gajjar, Aravind P Gandhi, Balasankar Ganesan, Ravindra Kumar Garg, Miglas Welay Gebregergis, Mesfin Gebrehiwot, Teferi Gebru Gebremeskel, Molla Getie, Delaram J Ghadimi, Sulmaz Ghahramani, Afsaneh Ghasemzadeh, Ramy Mohamed Ghazy, Maryam Gholamalizadeh, Sherief Ghozy, Jaleed Ahmed Gilani, Elena V Gnedovskaya, Barbara Niegia Garcia Goulart, Shi-Yang Guan, Sapna Gupta, Farrokh Habibzadeh, Mostafa Hadei, Najah R Hadi, Samer Hamidi, Ahmad Hammoud, Nasrin Hanifi, Netanja I Harlianto, Faizul Hasan, Hamidreza Hasani, Md Saquib Hasnain, Mahgol Sadat Hassan Zadeh Tabatabaei, Johannes Haubold, Rasmus J Havmoeller, Simon I Hay, Youssef Hbid, Golnaz Heidari, Mohammad Heidari, Mehdi Hemmati, Yuta Hiraike, Nguyen Quoc Hoan, Ramesh Holla, Mehdi Hosseinzadeh, Mowafa Househ, Hong-Han Huynh, Bing-Fang Hwang, Segun Emmanuel Ibitoye, Mehran Ilaghi, Olayinka Stephen Ilesanmi, Irena M Ilic, Milena D Ilic, Md. Rabiul Islam, Nahlah Elkudssiah Ismail, Gaetano Isola, Masao Iwagami, Louis Jacob, Akhil Jain, Ammar Abdulrahman Jairoun, Mihajlo Jakovljevic, Abubakar Ibrahim Jatau, Sathish Kumar Jayapal, Jost B Jonas, Nitin Joseph, Mikk Jürisson, Vidya Kadashetti, Rizwan Kalani, Vineet Kumar Kamal, Tanuj Kanchan, Himal Kandel, Jaakko Kaprio, Jafar Karami, Ibraheem M Karaye, Yeganeh Karimi, Arman Karimi Behnagh, Faizan Zaffar Kashoo, Gbenga A Kayode, Foad Kazemi, Emmanuelle Kesse-Guyot, Yousef Saleh Khader, Inn Kynn Khaing, Fayaz Khan, Mohammad Jobair Khan, Haitham Khatatbeh, Moawiah Mohammad Khatatbeh, Feriha Fatima Khidri, Moein Khormali, Atulya Aman Khosla, Kwanghyun Kim, Yun Jin Kim, Adnan Kisa, Sezer Kisa, Mika Kivimäki, Ali-Asghar Kolahi, Farzad Kompani, Oleksii Korzh, Karel Kostev, Nikhil Kothari, Kewal Krishan, Varun Krishna, Vijay Krishnamoorthy, Rita V Krishnamurthi, Mohammed Kuddus, Setor K Kunutsor, Maria Dyah Kurniasari, Dian Kusuma, Ville Kytö, Carlo La Vecchia, Chandrakant Lahariya, Daphne Teck Ching Lai, Hanpeng Lai, Tri Laksono, Tea Lallukka, Kamaluddin Latief, Kaveh Latifinaibin, Nhi Huu Hanh Le, Thao Thi Thu Le, Munjae Lee, Seung Won Lee, Wei-Chen Lee, Yo Han Lee, Jacopo Lenzi, Matilde Leonardi, Ming-Chieh Li, Xiaopan Li, Stephen S Lim, Jialing Lin, Xuefeng Liu, Valerie Lohner, László Lorenzovici, Paulo A Lotufo, Giancarlo Lucchetti, Jay B Lusk, Hawraz Ibrahim M. Amin, Armaan K Malhotra, Kashish Malhotra, Ahmad Azam Malik, Deborah Carvalho Malta, Mohammad Ali Mansournia, Lorenzo Giovanni Mantovani, Emmanuel Manu, Hamid Reza Marateb, Mirko Marino, Seyed Farzad Maroufi, Ramon Martinez-Piedra, Santi Martini, Miquel Martorell, Roy Rillera Marzo, Yasith Mathangasinghe, Elezebeth Mathews, Andrea Maugeri, Steven M McPhail, Asim Mehmood, Kamran Mehrabani-Zeinabad, Ritesh G Menezes, Sultan Ayoub Meo, Atte Meretoja, Tomislav Mestrovic, Chamila Dinushi Kukulege Mettananda, Tomasz Miazgowski, Ana Carolina Micheletti Gomide Nogueira de Sá, Giuseppe Minervini, Le Huu Nhat Minh, Andreea Mirica, Erkin M Mirrakhimov, Mohammad Mirza-Aghazadeh-Attari, Ajay Kumar Mishra, Prasanna Mithra, Abdalla Z Mohamed, Ahmed Ismail Mohamed, Ameen Mosa Mohammad, Soheil Mohammadi, Abdollah Mohammadian-Hafshejani, Shafiu Mohammed, Ali H Mokdad, Sabrina Molinaro, Shaher Momani, Mohammad Ali Moni, AmirAli Moodi Ghalibaf, Maryam Moradi, Yousef Moradi, Paula Moraga, Lidia Morawska, Rohith Motappa, Ahmed Msherghi, Kavita Munjal, Efren Murillo-Zamora, Christopher J L Murray, Ahamarshan Jayaraman Nagarajan, Ganesh R Naik, Balakrishnan Sukumaran Nair, Soroush Najdaghi, Noureddin Nakhostin Ansari, Sreenivas Narasimha Swamy, Shumaila Nargus, Delaram Narimani Davani, Zuhair S Natto, Javaid Nauman, Nawsherwan Nawsherwan, Vinod C Nayak, Athare Nazri-Panjaki, Ruxandra Irina Negoi, Soroush Nematollahi, Charles Richard James Newton, Duc Hoang Nguyen, Hau Thi Hien Nguyen, Hien Quang Nguyen, Phat Tuan Nguyen, Van Thanh Nguyen, Robina Khan Niazi, Yeshambel T Nigatu, Ali Nikoobar, Antonio Tolentino Nogueira de Sá, Jean Jacques Noubiap, Fred Nugen, Chimezie Igwegbe Nzoputam, Bogdan Oancea, Michael Safo Oduro, Tolulope R Ojo-Akosile, Hassan Okati-Aliabad, Akinkunmi Paul Okekunle, Andrew T Olagunju, Muideen Tunbosun Olaiya, Arão Belitardo Oliveira, Gláucia Maria Moraes Oliveira, Abdulhakeem Abayomi Olorukooba, Isaac Iyinoluwa Olufadewa, Abdulahi Opejin, Raffaele Ornello, Uchechukwu Levi Osuagwu, Amel Ouyahia, Mayowa O Owolabi, Ahmad Ozair, Mahesh Padukudru P A, Jagadish Rao Padubidri, Georgios D Panos, Leonidas D Panos, Ioannis Pantazopoulos, Romil R Parikh, Seoyeon Park, Jay Patel, Urvish K Patel, Dimitrios Patoulias, Paolo Pedersini, Emmanuel K Peprah, Gavin Pereira, Arokiasamy Perianayagam, Fanny Emily Petermann-Rocha, Anil K Philip, Michael A Piradov, Evgenii Plotnikov, Roman V Polibin, Maarten J Postma, Jalandhar Pradhan, Manya Prasad, Jagadeesh Puvvula, Nameer Hashim Qasim, Gangzhen Qian, Alberto Raggi, Fakher Rahim, Vafa Rahimi-Movaghar, Mosiur Rahman, Muhammad Aziz Rahman, Amir Masoud Rahmani, Mohammad Rahmanian, Sathish Rajaa, Ali Rajabpour Sanati, Pushp Lata Rajpoot, Prashant Rajput, Mahmoud Mohammed Ramadan, Shakthi Kumaran Ramasamy, Amey Rane, Annemarei Ranta, Sina Rashedi, Mohammad-Mahdi Rashidi, Devarajan Rathish, Ilari Rautalin, Salman Rawaf, Christian Razo, Murali Mohan

© 2025 GBD 2021 Global Subarachnoid Hemorrhage Risk Factors Collaborators. *JAMA Neurology*.

Rama Krishna Reddy, Elrashdy M. Moustafa Mohamed Redwan, Nazila Rezaei, Negar Rezaei, Mohsen Rezaeian, Gabriel J E Rinkel, Hermano Alexandre Lima Rocha, Jefferson Antonio Buendia Rodriguez, Leonardo Roever, Michele Romoli, Marina Romozzi, Allen Guy Ross, Himanshu Sekhar Rout, Priyanka Roy, Aly M A Saad, Zahra Saadatian, Siamak Sabour, Basema Ahmad Saddik, Erfan Sadeghi, Mohammad Reza Saeb, Usman Saeed, Fatemeh Saheb Sharif-Askari, Pragyan Monalisa Sahoo, Md Refat Uz Zaman Sajib, Mohamed A Saleh, Yoseph Leonardo Samodra, Vijaya Paul Samuel, Abdallah M Samy, Milena M Santric-Milicevic, Tanmay Sarkar, Gargi Sachin Sarode, Sachin C Sarode, Benn Sartorius, Maheswar Satpathy, Markus P Schlaich, Ione Jayce Ceola Schneider, Art Schuermans, Subramanian Senthilkumaran, Sadaf G Sepanlou, Yashendra Sethi, Ahmed Nabil Shaaban, Mahan Shafie, Masood Ali Shaikh, Summaiya Zareen Shaikh, Muhammad Aaqib Shamim, Anas Shamsi, Alfiya Shamsutdinova, Mohd Shanawaz, Mohammed Shannawaz, Amin Sharifan, Javad Sharifi Rad, Vishal Sharma, Bereket Beyene Shashamo, Mika Shigematsu, Aminu Shittu, Ivy Shiue, Nathan A Shlobin, Seyed Afshin Shorofi, Emmanuel Edwar Siddig, Baljinder Singh, Paramdeep Singh, Puneetpal Singh, Farrukh Sobia, Soroush Soraneh, Michael Spartalis, Suresh Kumar Srinivasamurthy, Jeffrey D Stanaway, Muhammad Haroon Stanikzai, Benjamin A Stark, Antonina V Starodubova, Jing Sun, Zhong Sun, Chandan Kumar Swain, Lukasz Szarpak, Payam Tabaee Damavandi, Seyyed Mohammad Tabatabaei, Celine Tabche, Jabeen Taiba, Iman M Talaat, Jacques Lukenze Tamuzi, Ker-Kan Tan, Ingan Ukur Tarigan, Mohamad-Hani Temsah, Masayuki Teramoto, Ramna Thakur, Kavumpurathu Raman Thankappan, Rasiah Thayakaran, Sathish Thirunavukkarasu, Jansje Henny Vera Ticoalu, Marcello Tonelli, Roman Topor-Madry, Marcos Roberto Tovani-Palone, An Thien Tran, Jasmine T Tran, Nguyen Tran Minh Duc, Thomas Clement Truelsen, Daniel Hsiang-Te Tsai, Atta Ullah, Bhaskaran Unnikrishnan, Jibrin Sammani Usman, Sanaz Vahdati, Rohollah Valizadeh, Jef Van den Eynde, Joe Varghese, Siavash Vaziri, Narayanaswamy Venketasubramanian, Mervyn D I Vergouwen, Dominique Vervoort, Jorge Hugo Villafañe, Manish Vinayak, Victor Volovici, Hatem A Wafa, Yasir Waheed, Waseem Wahood, Mandaras Tariku Walde, Yanzhong Wang, Nuwan Darshana Wickramasinghe, Peter Willeit, Asrat Arja Wolde, Charles D A Wolfe, Yihun Miskir Wubie, Hong Xiao, Suowen Xu, Xiaoyue Xu, Amir Yarahmadi, Habib Yaribeygi, Sanni Yaya, Pengpeng Ye, Dong Keon Yon, Naohiro Yonemoto, Chuanhua Yu, Aurora Zanghì, Michael Zastrozhin, Chen Zhang, Yunquan Zhang, Zhi-Jiang Zhang, Zhiqiang Zhang, Hanqing Zhao, Shang Cheng Zhou, and Magdalena Zielińska.

Drafting the work or revising it critically for important intellectual content

Yohannes Habtegiorgis Abate, Hedayat Abbastabar, Foad Abd-Allah, Atef Abdelkader, Parsa Abdi, Auwal Abdullahi, Olugbenga Olusola Abiodun, Mohamed Abouzid, Samir Abu Rumeileh, Ahmed Abualhasan, Hasan Abualruz, Hana J Abukhadijah, Ahmed Abu-Zaid, Lawan Hassan Adamu, Isaac Yeboah Addo, Rufus Adesoji Adedoyin, Oyelola A Adegboye, Saryia Adra, Bright Opoku Ahinkorah, Danish Ahmad, Ali Ahmed, Haroon Ahmed, Syed Anees Ahmed, Mohammed Ahmed Akkaif, Yazan Al-Ajlouni, Mohammed Albashtawy, Mohammed Usman Ali, Sami Almustanyir, Mahmoud A Alomari, Ahmad Alrawashdeh, Rustam Al-Shahi Salman, Awais Altaf, Alaa B. Al-Tammemi, Nelson Alvis-Guzman, Hassan Alwafi, Yaser Mohammed Al-Worafi, Hany Aly, Mohammad Sharif Ibrahim Alyahya, Karem H Alzoubi, Reza Amani, Tarek Tawfik Amin, Alireza Amindarolzarbi, Ganiyu Adeniyi Amusa, Deanna Anderlini, Dhanalakshmi Angappan, Abhishek Anil, Boluwatife Stephen Anuoluwa, Saleha Anwar, Anayochukwu Edward Anyasodor, Geminn Louis Carace Apostol, Jalal Arabloo, Johan Ärnlöv, Kurnia Dwi Artanti, Ashokan Arumugam, Zahra Aryan, Seyyed Shamsadin Athari, Avinash Aujayeb, Adedapo Wasiu Awotidebe, Sina Azadnajafabad, Shahkaar Aziz, Ahmed Y. Azzam, Giridhara Rathnaiah Babu, Ruhai Bai, Atif Amin Baig, Abdulaziz T Bako, Ovidiu Constantin Baltatu, Kiran Bam, Maciej Banach, Soham Bandyopadhyay, Biswajit Banik, Mainak Bardhan, Suzanne Lyn Barker-Collo, Till Winfried Bärnighausen, Hiba Jawdat Barqawi, Mohammad-Mahdi Bastan, Sanjay Basu, Shelly L Bell, Isabela M Bensenor, Kebede A Beyene, Akshaya Srikanth Bhagavathula, Sonu Bhaskar, Ajay Nagesh Bhat, Vivek Bhat, Gurjit Kaur Bhatti, Jasvinder Singh Singh Bhatti, Boris Bikbov, Veera R Bitra, Hamed Borhany, Susanne Breitner, Hermann Brenner, Raffaele Bugiardini, Norma B Bulamu, Lucas Scotta Cabral, Florentino Luciano Caetano dos Santos, Daniela Calina, Luciana Aparecida Campos, Ismael Campos-Nonato, Angelo Capodici, Felix Carvalho, Carlos A Castañeda-Orjuela, Luca Cegolon, Joshua Chadwick, Chiranjib Chakraborty, Sandip Chakraborty, Rama Mohan Chandika, Vijay Kumar Chattu, Anis Ahmad Chaudhary, Fatemeh Chichagi, Patrick R Ching, Dinh-Toi Chu, Alyssa Columbus, Michael H Criqui, Alanna Gomes da Silva, Koustuv Dalal, Emanuele D'Amico, Mohsen Dashti, Ivan Delgado-Enciso, Syed Masudur Rahman Dewan, Amol S Dhane, Michael J Diaz, Mahmoud Dibas, Thanh Chi Do, Sushil Dohare, Klara Georgieva Dokova, Deepa Dongarwar, Mario D'Oria, Ojas Prakashbhai Doshi, Rajkumar Prakashbhai Doshi, Abdel Douiri, Haneil Larson Dsouza, Siddhartha Dutta, Arkadiusz Marian Dziedzic, Abdel Rahman E'mar, David Edvardsson, Defi Efendi, Nevine El Nahas, Islam Y Elgendy, Muhammed Elhadi, Chadi Eltaha, Mohd. Elmagzoub Eltahir, Theophilus I Emeto, Natalia Fabin, Adeniyi Francis Fagbamigbe, Ayesha Fahim, Jawad Fares, Nelsensius Klau Fauk, Nuno Ferreira, Getahun Fetensa, Florian Fischer, Matteo Foschi, Ni Kadek Yuni Fridayani, Abduzhappar Gaipov, Avi A Gajjar, Balasankar Ganesan, Ravindra Kumar Garg, Miglas Welay Gebregergis, Molla Getie, Delaram J Ghadimi, Fataneh Ghadirian, Sulmaz Ghahramani,

© 2025 GBD 2021 Global Subarachnoid Hemorrhage Risk Factors Collaborators. *JAMA Neurology*.

Afsaneh Ghasemzadeh, Ramy Mohamed Ghazy, Sherief Ghozy, Artyom Urievich Gil, Jaleed Ahmed Gilani, Elena V Gnedovskaya, Barbara Niegia Garcia Goulart, Shi-Yang Guan, Sapna Gupta, Farrokh Habibzadeh, Mostafa Hadei, Najah R Hadi, Ahmad Hammoud, Nasrin Hanifi, Graeme J Hankey, Netanja I Harlianto, Josep Maria Haro, Faizul Hasan, Hamidreza Hasani, Md Saquib Hasnain, Mahgol Sadat Hassan Zadeh Tabatabaei, Johannes Haubold, Rasmus J Havmoeller, Simon I Hay, Golnaz Heidari, Mehdi Hemmati, Yuta Hiraike, Nguyen Quoc Hoan, Ramesh Holla, Sorin Hostiuc, Junjie Huang, Hong-Han Huynh, Segun Emmanuel Ibitoye, Adalia Ikiroma, Mehran Ilaghi, Olayinka Stephen Ilesanmi, Irena M Ilic, Milena D Ilic, Md. Rabiul Islam, Nahlah Elkudssiah Ismail, Hiroyasu Iso, Gaetano Isola, Louis Jacob, Abdollah Jafarzadeh, Akhil Jain, Mihajlo Jakovljevic, Abubakar Ibrahim Jatau, Sathish Kumar Jayapal, Jost B Jonas, Nitin Joseph, Mikk Jürisson, Vidya Kadashetti, Rizwan Kalani, Arun Kamireddy, Himal Kandel, Jaakko Kaprio, Yeganeh Karimi, Faizan Zaffar Kashoo, Gbenga A Kayode, Foad Kazemi, Emmanuelle Kesse-Guyot, Fayaz Khan, Mohammad Jobair Khan, Haitham Khatatbeh, Moawiah Mohammad Khatatbeh, Hamid Reza Khayat Kashani, Khalid A Kheirallah, Feriha Fatima Khidri, Atulya Aman Khosla, Kwanghyun Kim, Yun Jin Kim, Adnan Kisa, Sezer Kisa, Mika Kivimäki, Farzad Kompani, Miikka Korja, Oleksii Korzh, Karel Kostev, Nikhil Kothari, Kewal Krishan, Varun Krishna, Rita V Krishnamurthi, Mohammed Kuddus, Mukhtar Kulimbet, Setor K Kunutsor, Maria Dyah Kurniasari, Dian Kusuma, Ville Kytö, Carlo La Vecchia, Chandrakant Lahariya, Daphne Teck Ching Lai, Hanpeng Lai, Tea Lallukka, Kamaluddin Latief, Kaveh Latifinaibin, Nhi Huu Hanh Le, Thao Thi Thu Le, Jacopo Lenzi, Matilde Leonardi, Valerie Lohner, László Lorenzovici, Giancarlo Lucchetti, Ricardo Lutzky Saute, Armaan K Malhotra, Kashish Malhotra, Ahmad Azam Malik, Deborah Carvalho Malta, Lorenzo Giovanni Mantovani, Emmanuel Manu, Hamid Reza Marateb, Mirko Marino, Ramon Martinez-Piedra, Miquel Martorell, Roy Rillera Marzo, Yasith Mathangasinghe, Andrea Maugeri, Steven M McPhail, Asim Mehmood, Ritesh G Menezes, Sultan Ayoub Meo, Atte Meretoja, Tomislav Mestrovic, Chamila Dinushi Kukulege Mettananda, Tomasz Miazgowski, Ana Carolina Micheletti Gomide Nogueira de Sá, Le Huu Nhat Minh, Mohammad Mirza-Aghazadeh-Attari, Prasanna Mithra, Abdalla Z Mohamed, Ameen Mosa Mohammad, Abdollah Mohammadian-Hafshejani, Shafiu Mohammed, Ali H Mokdad, Sabrina Molinaro, Mohammad Ali Moni, AmirAli Moodi Ghalibaf, Maryam Moradi, Yousef Moradi, Paula Moraga, Rohith Motappa, Ahmed Msherghi, Efren Murillo-Zamora, Ahamarshan Jayaraman Nagarajan, Soroush Najdaghi, Noureddin Nakhostin Ansari, Sreenivas Narasimha Swamy, Shumaila Nargus, Delaram Narimani Davani, Zuhair S Natto, Javaid Nauman, Nawsherwan Nawsherwan, Vinod C Nayak, Ruxandra Irina Negoi, Soroush Nematollahi, Charles Richard James Newton, Duc Hoang Nguyen, Hau Thi Hien Nguyen, Hien Quang Nguyen, Phat Tuan Nguyen, Van Thanh Nguyen, Robina Khan Niazi, Yeshambel T Nigatu, Antonio Tolentino Nogueira de Sá, Jean Jacques Noubiap, Fred Nugen, Chimezie Igwegbe Nzoputam, Bogdan Oancea, Sylvester Reuben Okeke, Andrew T Olagunju, Arão Belitardo Oliveira, Abdulhakeem Abayomi Olorukooba, Raffaele Ornello, Esteban Ortiz-Prado, Uchechukwu Levi Osuagwu, Amel Ouyahia, Mayowa O Owolabi, Mahesh Padukudru P A, Alicia Padron-Monedero, Jagadish Rao Padubidri, Demosthenes Panagiotakos, Leonidas D Panos, Ioannis Pantazopoulos, Romil R Parikh, Jay Patel, Urvish K Patel, Dimitrios Patoulias, Paolo Pedersini, Gavin Pereira, Arokiasamy Perianayagam, Norberto Perico, Fanny Emily Petermann-Rocha, Michael A Piradov, Maarten J Postma, Jalandhar Pradhan, Manya Prasad, Jagadeesh Puvvula, Nameer Hashim Qasim, Alberto Raggi, Fakher Rahim, Vafa Rahimi-Movaghar, Mohammad Rahmanian, Sathish Rajaa, Ali Rajabpour Sanati, Prashant Rajput, Mahmoud Mohammed Ramadan, Shakthi Kumaran Ramasamy, Annemarei Ranta, Mohammad-Mahdi Rashidi, Devarajan Rathish, Ilari Rautalin, Salman Rawaf, Elrashdy M. Moustafa Mohamed Redwan, Giuseppe Remuzzi, Nazila Rezaei, Gabriel J E Rinkel, Hermano Alexandre Lima Rocha, Jefferson Antonio Buendia Rodriguez, Leonardo Roever, Marina Romozzi, Allen Guy Ross, Nitai Roy, Aly M A Saad, Siamak Sabour, Simona Sacco, Basema Ahmad Saddik, Usman Saeed, Fatemeh Saheb Sharif-Askari, Amirhossein Sahebkar, Pragyan Monalisa Sahoo, Md Refat Uz Zaman Sajib, Luciane B Salaroli, Vijaya Paul Samuel, Abdallah M Samy, Milena M Santric-Milicevic, Aswini Saravanan, Tanmay Sarkar, Gargi Sachin Sarode, Sachin C Sarode, Maheswar Satpathy, Markus P Schlaich, Ione Jayce Ceola Schneider, Art Schuermans, Siddharthan Selvaraj, Sadaf G Sepanlou, Yashendra Sethi, Allen Seylani, Ahmed Nabil Shaaban, Mahan Shafie, Moyad Jamal Shahwan, Summaiya Zareen Shaikh, Muhammad Aaqib Shamim, Alfiya Shamsutdinova, Mohd Shanawaz, Mohammed Shannawaz, Amin Sharifan, Javad Sharifi Rad, Vishal Sharma, Mahabalesh Shetty, Premalatha K Shetty, Mika Shigematsu, Aminu Shittu, Nathan A Shlobin, Seyed Afshin Shorofi, Emmanuel Edwar Siddig, Paramdeep Singh, Puneetpal Singh, Surjit Singh, Ranjan Solanki, Shipra Solanki, Michael Spartalis, Muhammad Haroon Stanikzai, Benjamin A Stark, Antonina V Starodubova, Chandan Kumar Swain, Lukasz Szarpak, Payam Tabaee Damavandi, Seyed-Amir Tabatabaeizadeh, Celine Tabche, Iman M Talaat, Jacques Lukenze Tamuzi, Ker-Kan Tan, Mohamad-Hani Temsah, Masayuki Teramoto, Ramna Thakur, Sathish Thirunavukkarasu, Krishna Tiwari, Marcello Tonelli, Roman Topor-Madry, Marcos Roberto Tovani-Palone, An Thien Tran, Jasmine T Tran, Thang Huu Tran, Nguyen Tran Minh Duc, Thomas Clement Truelsen, Thien Tan Tri Tai Truyen, Daniel Hsiang-Te Tsai, Brigid Unim, Bhaskaran Unnikrishnan, Carolyn Anne Unsworth, Jibrin Sammani Usman, Sanaz Vahdati, Asokan Govindaraj Vaithinathan, Jef Van den Eynde, Tommi Juhani Vasankari, Narayanaswamy Venketasubramanian, Mervyn D I Vergouwen, Dominique Vervoort, Manish Vinayak, Victor Volovici, Hatem A Wafa, Waseem Wahood, Yanzhong Wang, Nuwan Darshana Wickramasinghe, Peter Willeit,
© 2025 GBD 2021 Global Subarachnoid Hemorrhage Risk Factors Collaborators. *JAMA Neurology*.

Charles D A Wolfe, Kazumasa Yamagishi, Amir Yarahmadi, Sanni Yaya, Dong Keon Yon, Naohiro Yonemoto, Aurora Zanghì, Iman Zare, Michael Zastrozhin, Chen Zhang, Zhi-Jiang Zhang, Zhiqiang Zhang, Abzal Zhumagaliuly, Hafsa Zia, Magdalena Zielińska, and Samer H Zyoud.

Managing the estimation or publications process
Simon I Hay, Ali H Mokdad, Christopher J L Murray, and Ilari Rautalin.

© 2025 GBD 2021 Global Subarachnoid Hemorrhage Risk Factors Collaborators. *JAMA Neurology*.

2. eMethods

In the following section, we have provided some methodological appendices to supply the information reported in the manuscript: “Global, Regional and National Burden of Non-traumatic Subarachnoid Hemorrhage – A Systematic Analysis for the Global Burden of Disease Study 2021”. This document only summarizes the key methodological aspects of the current manuscript while the details of the Global Burden of Disease (GBD) 2021 study have been presented in previous capstone articles. Since the details of many subarachnoid hemorrhage (SAH) -specific methodologies have also been reported in previous GBD publications focusing on stroke and its pathological types,[1,2] we have not duplicated this information. This manuscript including its appendix materials has been produced as part of the GBD Collaborator Network following the GBD Protocol[3] and Guidelines for Accurate and Transparent Health Estimates Reporting (GATHER) recommendations.[4]

2.1 Data Sources

To identify the most relevant and updated data sources for SAH burden, the GBD 2021 study included a systematic review according to the following search terms:

- PubMed: ("stroke"[TIAB] OR "ischaemic stroke"[TIAB] OR "ischaemic stroke"[TIAB] OR "cerebral infarction"[TIAB] OR "intracerebral hemorrhage"[TIAB] OR "intracerebral haemorrhage"[TIAB] OR "subarachnoid hemorrhage"[TIAB] OR "subarachnoid haemorrhage"[TIAB]) AND (incidence[TIAB] OR prevalence[TIAB] OR "excess mortality"[TIAB] OR "case fatality"[TIAB] OR "mortality ratio"[TIAB]) AND ("2017/09/01"[PDAT] : "2020/02/25"[PDAT])

Moreover, previous GBD studies[1] have utilized the following search terms to identify relevant data sources:

- Google Scholar search: ("subarachnoid hemorrhage" OR “subarachnoid haemorrhage”) AND (incidence OR prevalence OR mortality OR epidemiology). Reviewed first 1000 hits, sorted by relevance.
- GIM search: (tw:("subarachnoid hemorrhage") OR tw:(“subarachnoid haemorrhage”)) AND (tw:(incidence) OR tw:(prevalence) OR tw:(mortality) OR tw:(epidemiology)) AND NOT (tw:(rats) OR tw:(mice) OR tw:(dogs) OR tw:(apes) OR tw:(monkeys)). Dates of search: 01Jan2010 – 31Aug2017

All used data input sources for the GBD 2021 study are publicly available on the website: https://ghdx.healthdata.org/gbd-2021/sources. As data sources for causes of death, nonfatal health outcomes and relative risks can be sorted by causes, SAH-specific sources can also be found on the same website. Besides crude references, each data source includes metadata describing its location, year, and type. Overall, there were 2,563 data sources for fatal SAHs, 311 data sources for non-fatal SAHs, and 36 data sources for SAH risk factors from 132 different countries/regions between 1963 and 2022. All data sources for fatal SAH were based on vital registrations comprising 88% of overall data sources while data sources for non-fatal SAHs and SAH-specific risk factors have primarily been gathered via systematic reviews of available literature. Most sources originate from high-income regions where 97% of the countries have at least one SAH-specific data source. On the other hand, 72 countries (35%) have no SAH-specific data source and the proportion in the 46 countries of Sub-Saharan Africa is as high as 87%. In comparison to previous systematic reviews on the time trends of SAH incidence[5] and case-fatality,[6] the GBD 2021 study includes over 80% of the identified population-based publications (or overlapping studies) as its data sources emphasizing its comprehensive coverage of the previously published evidence on non-fatal and fatal burden estimates of SAH. The distributions of data sources by location, year and type have been presented in Supplementary Figures 1 (all sources combined), 2 (fatal sources), 3 (nonfatal sources) and 4 (relative risk sources). Since a single source may include data from several years and/or locations, the annual and regional number of sources exceeds the total number of data sources.

© 2025 GBD 2021 Global Subarachnoid Hemorrhage Risk Factors Collaborators. *JAMA Neurology*.

Supplementary Figure 1. The numbers and distributions of data sources for subarachnoid hemorrhage (SAH) included in the Global Burden of Diseases 2021 study by year, type, and region.

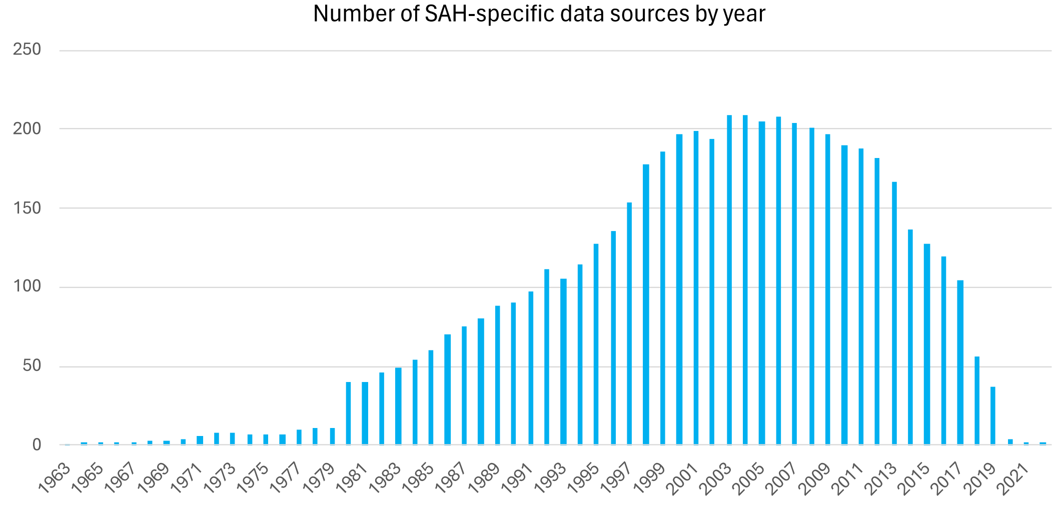

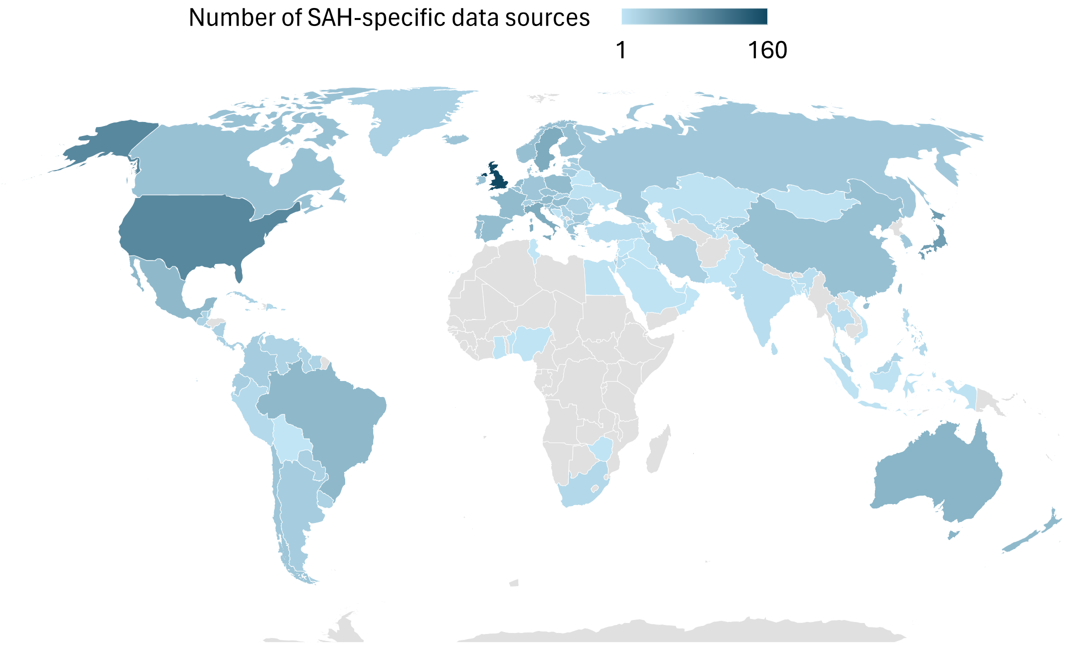

© 2025 GBD 2021 Global Subarachnoid Hemorrhage Risk Factors Collaborators. *JAMA Neurology*.

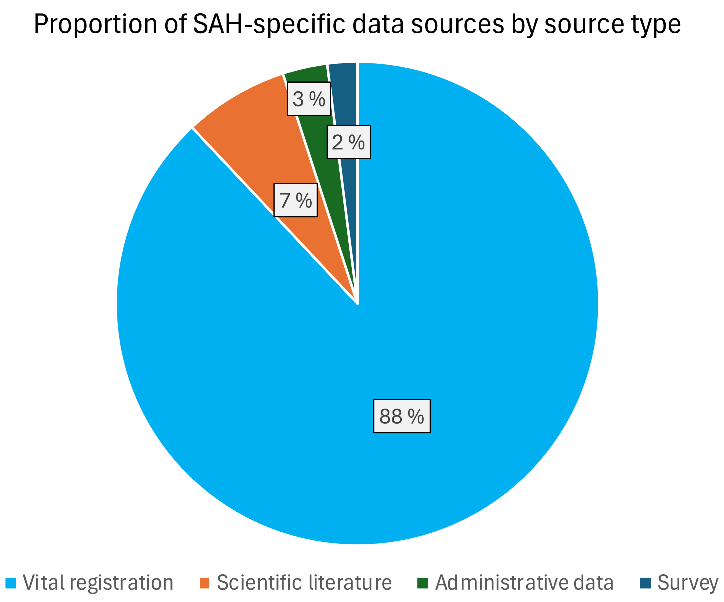

© 2025 GBD 2021 Global Subarachnoid Hemorrhage Risk Factors Collaborators. *JAMA Neurology*.

Supplementary Figure 2. The numbers and distributions of data sources for fatal subarachnoid hemorrhage (SAH) included in the Global Burden of Diseases 2021 study by year and region.

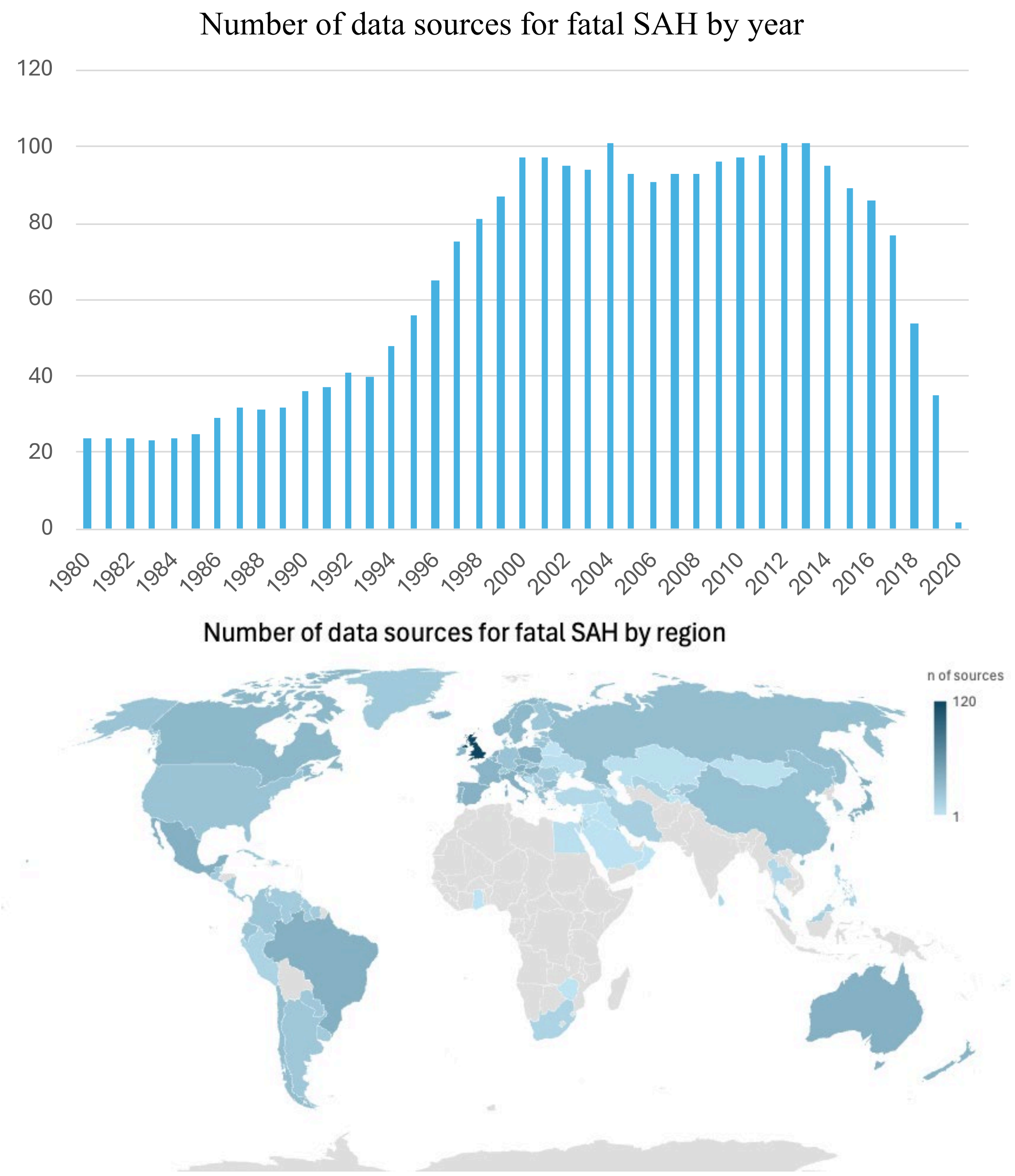

© 2025 GBD 2021 Global Subarachnoid Hemorrhage Risk Factors Collaborators. *JAMA Neurology*.

Supplementary Figure 3. The numbers and distributions of data sources for non-fatal subarachnoid hemorrhage (SAH) included in the Global Burden of Diseases 2021 study by year, type, and region.

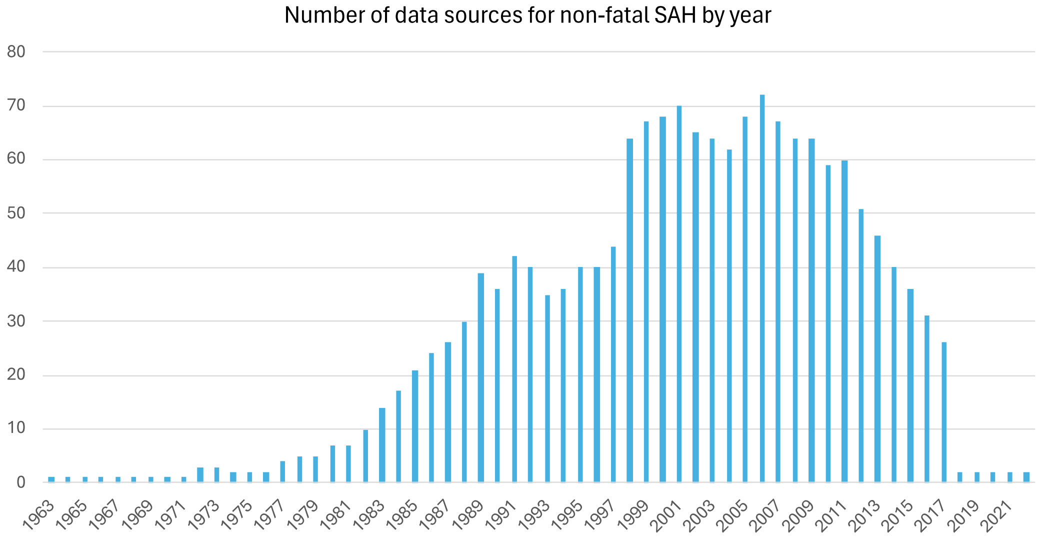

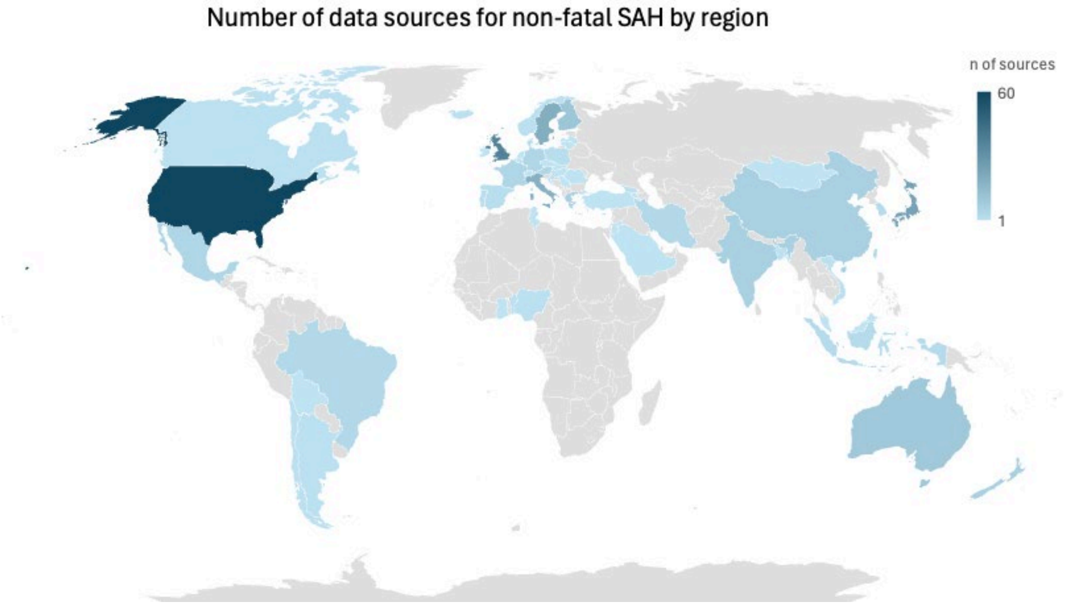

© 2025 GBD 2021 Global Subarachnoid Hemorrhage Risk Factors Collaborators. *JAMA Neurology*.

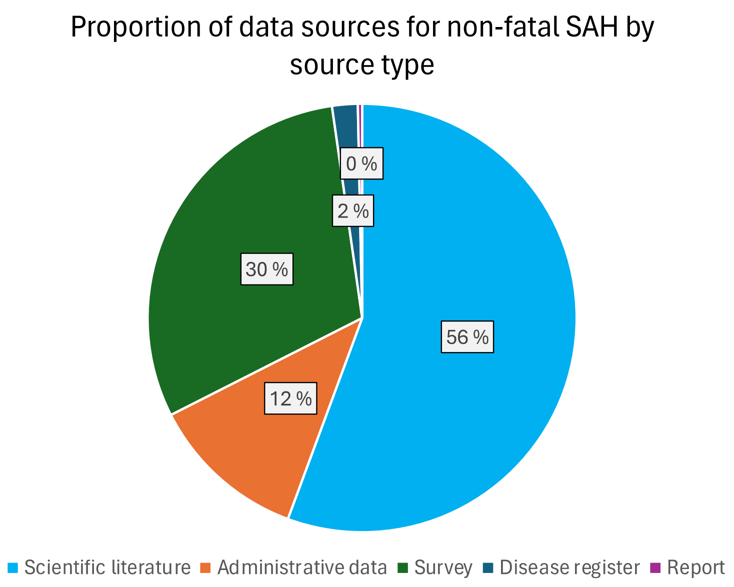

© 2025 GBD 2021 Global Subarachnoid Hemorrhage Risk Factors Collaborators. *JAMA Neurology*.

Supplementary Figure 4. The numbers and distributions of data sources for subarachnoid hemorrhage (SAH) risk factors included in the Global Burden of Diseases 2021 study by year, region and risk factor.

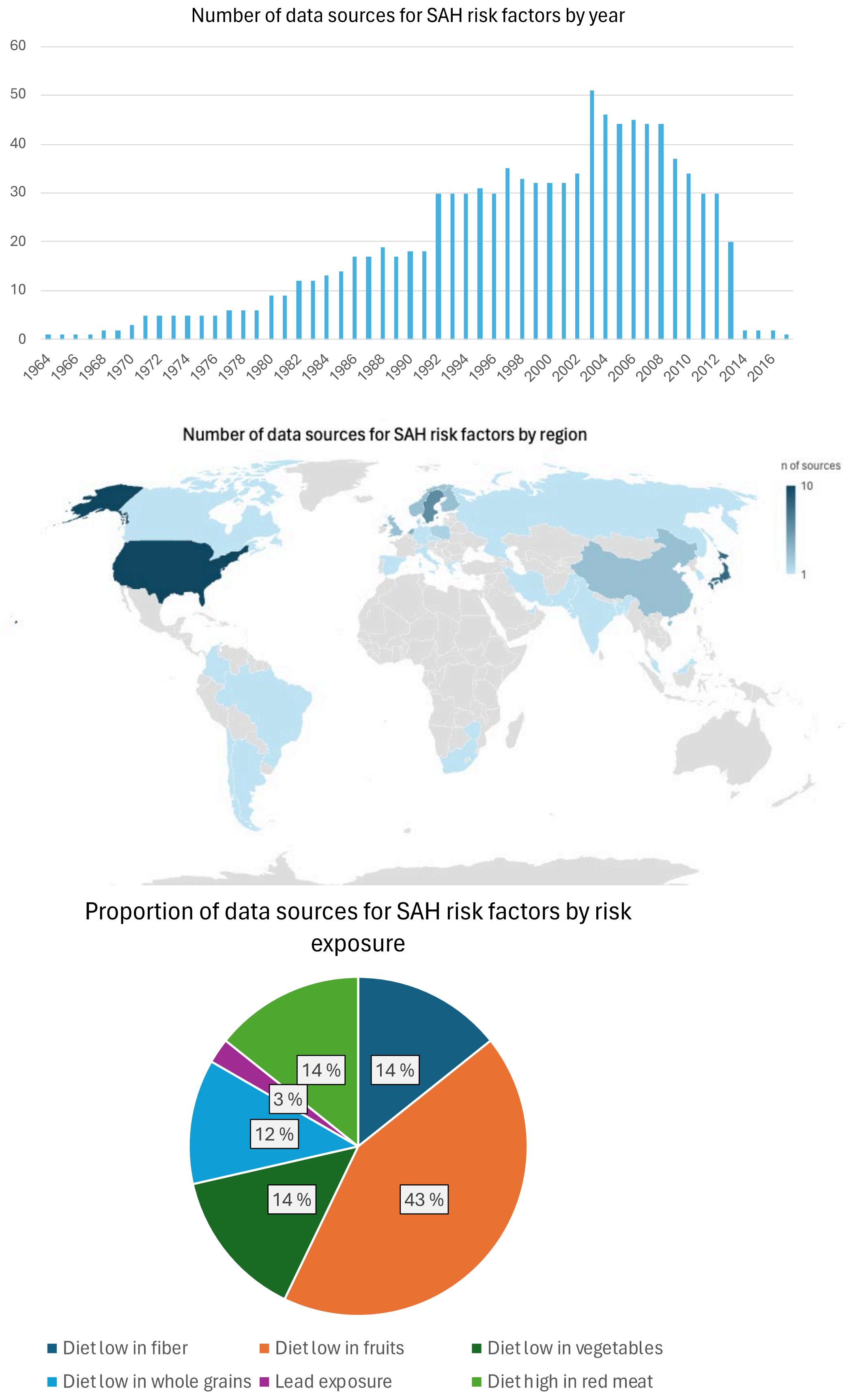

© 2025 GBD 2021 Global Subarachnoid Hemorrhage Risk Factors Collaborators. *JAMA Neurology*.

2.2 Data Modeling

Previous articles describe the details of general, stroke-specific, and SAH-specific data modeling in the GBD studies.[1,2] Primarily, the GBD 2021 study used two statistical modeling tools namely Cause of Death Ensemble modeling (CODEm)[7] and Disease-Model-Bayesian Meta-Regression 2.1 (DisMod-MR)[8] to produce annual burden estimates of SAH across different population groups and geographical locations between 1990 and 2021. While both modeling tools utilize geospatial relationships of relevant covariates (Supplementary Tables 1), CODEm is designed to model fatal estimates by integrating the data of multiple statistical modeling tools and covariate permutations whereas the DisMod-MR uses a Bayesian meta-regression framework to model nonfatal outcomes such as cause-specific incidence and prevalence estimates. Besides direct SAH death diagnoses (e.g., 430 in ICD-9 and I60 in ICD-10), part of the SAH fatalities is gathered via the redistribution of implausible cause-of-death diagnosis codes in vital registrations (so-called garbage codes). For example, some unspecified stroke/cardiovascular deaths and deaths related to non-fatal causes (e.g., hypertension) or symptoms (e.g., convulsions, cardiac arrest, and coma) are assumed to be caused by SAH.[9] Since some of the fatal ICD-8 data points and vital registrations from Tibet were observed to generate implausibly high and inconsistent time trends with the rest of the data for fatal SAH estimates in the GBD 2021 study, they were excluded from the final CODEm models. In terms of non-fatal SAH estimates, systematic reviews of unpublished and published evidence served as a primary method to gather data inputs for the DisMod-MR modeling tool. In addition to the WHO's reference case definition for SAH (non-traumatic first-ever stroke type caused by bleeding into the subarachnoid space of the brain), data inputs with an alternative definition such as SAH caused exclusively by ruptured intracranial aneurysm, were adjusted to the model as described earlier.[1] All modelling analyses in the GBD 2021 were completed by using Python (version 3.10.4), Stata (version 13.1), and R (version 4.2.1) statistical software.

Supplementary Table 1. Selected covariates for SAH-specific modeling in the GBD 2021 study.

Covariate	Model
Smoking prevalence	CODEm
Systolic blood pressure (mmHg)	CODEm + DisMod-MR
Healthcare access and quality index	CODEm
Lag distributed income per capita (I$)	CODEm + DisMod-MR
Alcohol (litres per capita)	CODEm

2.3 Risk Factor Assessment

The GBD 2021 study uses a comparative risk assessment framework to estimate the cause-specific proportions of the burden that are attributable to the identified risk factors.[10] Briefly, this estimation is based on seven interrelated methodological components. First, risk-outcome pairs and associative relative risk (RR) estimates with convincing or probable evidence are identified via systematic literature searches and calculated by using analytical tools based on meta-regression. Second, the exposure levels of each risk factor in each age-sex-location-year category are estimated by using two Bayesian statistical models, namely spatiotemporal Gaussian process regression and DisMod-MR 2.1. Third, based on the available epidemiological evidence, risk factor-specific theoretical minimum risk exposure levels (TMRELs) are determined. TMREL indicates the level of exposure that would minimize the health risk related to each risk factor. Fourth, by using the data on exposure levels, RRs, and TMRELs, population-attributable fractions (PAFs) are calculated for each risk-outcome pair. PAF represents the proportional change in disease burden in case the risk exposure would be reduced to the TMREL. Fifth, summary exposure variables (SEVs; risk-weighted prevalences of exposure by age) are calculated for each risk factor. Sixth, mediation between individual risk factors on the same pathophysiological pathway to an outcome of interest is assessed and considered in risk factor clusters. Lastly, attributable burden estimates are determined by age group, sex, location, and year. For SAH, the GBD 2021 study includes the data on all three risk clusters (i.e., behavioral, environmental/occupational, and metabolic risks) and 14 individual risk factors listed in Supplementary Table 2. However, apart from the dietary risks and lead exposure, RR estimates used for PAF calculations of SAH are based on the stroke literature, not specifically on SAH. Moreover, data on some of the identified risk factors for stroke in general such as high fasting plasma glucose, high low-density lipoprotein cholesterol, and low physical activity are not currently available for SAH. While the PAF calculations of risk clusters consider the interactions between individual risk factors, the PAFs of each risk factor are not mutually exclusive. Therefore, the sum of

© 2025 GBD 2021 Global Subarachnoid Hemorrhage Risk Factors Collaborators. *JAMA Neurology*.

risk factor-specific PAFs may exceed 100% due to the partial or complete overlapping effects of multiple risk factors.

Supplementary Table 2. Fourteen individual risk factors for subarachnoid hemorrhage in the Global Burden of Disease 2021 study. Theoretical minimum risk exposure levels are defined as the absence of risk factors.

Risk factors (cluster)	Definition
Diet high in red meat (B)	Any consumption of red meat including pork, beef, lamb, and goat but excluding poultry, fish, eggs, and all processed meats
Diet high in sodium (B)	Daily consumption of sodium > 5 g
Diet low in fiber (B)	Daily consumption of fiber < 21g
Diet low in fruits (B)	Daily consumption of fruits < 200 g
Diet low in vegetables (B)	Daily consumption of vegetables < 350 g
Secondhand smoke (B)	Daily indoor exposure to second-hand smoking
Smoking (B)	Previous or current smoking of tobacco
Ambient particulate matter pollution (E)	Annual average daily exposure to outdoor air concentration of $PM_{2.5}$ > 8.8µg/m^3
High temperature (E)	Ambient temperature higher than the temperature associated with the minimum mortality risk in a given location and year
Household air pollution from solid fuels (E)	Any exposure to an indoor concentration of $PM_{2.5}$
Lead exposure (E)	Lead concentration of blood > 20 µg/L
Low temperature (E)	Ambient temperature lower than the temperature associated with the minimum mortality risk in a given location and year
High body-mass index (M)	BMI > 23.0 kg/m2
High systolic blood pressure (M)	Systolic blood pressure > 110-115 mm/Hg

B = behavioral risk cluster; BMI = body mass index; E = environmental/occupational risk cluster; M = metabolic risk cluster

2.4 Data Presentation

Previous capstone articles describe the details of standard data presentation methods in the GBD 2021 study. In Supplementary Table 3, we have summarized the key variables and terms used in the present article for the global burden of SAH and its risk factors.

Supplementary Table 3. Key variables/terms used in the current manuscript regarding the global burden of SAH.

Variable/term	Definition
**General terms**	
Age-standardization	Since 2017, the GBD studies have updated the standard population age structure (i.e., the GBD Standard Population) with each release. This is done by calculating the unweighted mean of the age-specific proportional distributions from the most recent GBD population estimates for all countries with populations exceeding 5 million. Age-standardized rates are then derived by calculating the weighted average of the age-specific rates based on these proportions.
Uncertainty interval (UI)	In the GBD 2021, every estimate is calculated 1,000 times, each time sampling from distributions rather than point estimates for data inputs, data transformations and model choice. After ordering the 1,000 values from smallest to largest, the 95% uncertainty interval is determined by the range from 25th and 975th value representing the certainty of a point estimate.
Sociodemographic index (SDI)	A compound measure that represents the regional sociodemographic development by accounting the location- and year-specific data on income, average years of schooling and fertility levels
**Outcome/burden estimates**	
Incidents / Incidence	New SAH cases including non-hospitalized and hospitalized as well as fatal and non-fatal events occurring in a specified population in a given time period. Estimates are presented in absolute numbers (incidents) or age-standardized rates per 100,000 people (incidence) per year.
Prevalent cases / Prevalence	Number of present SAH survivors (alive 28 days after SAH) in a specified population at any particular moment in time. Estimates are presented in absolute numbers (prevalent cases) or age-standardized rates per 100,000 people (prevalence) per year.
Deaths/mortality	Number of deaths attributed to SAH in a specified population and time period. Estimates include deaths among non-hospitalized and hospitalized SAH cases but because the SAH deaths are not linked with SAH incidents, they do not represent case-fatality rates. Moreover, since some of the SAH-related deaths may have occurred months after the actual SAH, the number of annual deaths may be greater than the number of annual incidents in small population groups. Estimates are presented in absolute numbers (deaths) or age-standardized rates per 100,000 people (mortality) per year.
Disability-adjusted life year	Equals the loss of one year of healthy life due to SAH. Calculated by the sum of years of life lost due to premature mortality (YLLs) and years lived with disability (YLDs) in a specified population and time period. Estimates are presented in absolute numbers (DALYs) or age-standardized rates per 100,000 people (DALY rates) per year.

© 2025 GBD 2021 Global Subarachnoid Hemorrhage Risk Factors Collaborators. *JAMA Neurology*.

Years of life lost (YLL)	Represent years lost due to premature mortality caused by SAH. Calculated by subtracting the age at death from the sex-, location- and year-specific life expectancy for an average person at the same age.
Years lived with disability (YLD)	Represent years lived in less than ideal health due to SAH. Calculated by multiplying the prevalence of SAH by the stroke-specific disability weights. Disability weights vary between 0 (perfect health) and 1 (equivalent to death) and are based on several public health surveys in low-, middle-, and high-income countries.
**Population groups and geographical locations**	
Sex (n=2)	Men, Women
Age groups (n=4)	Children (0–14 years old), Young adults (15–49 years old), Old adults (50–74 years old), Very old adults (≥75 years old)
Global Burden of Diseases study super-regions (n=7)	Central Europe, Eastern Europe, and Central Asia; High-income; Latin America and Caribbean; North Africa and the Middle East; South Asia; Southeast Asia, East Asia, and Oceania; Sub-Saharan Africa
Global Burden of Diseases study regions (n=21)	Andean Latin America; Australasia; Caribbean; Central Asia; Central Europe; Central Latin America; Central Sub-Saharan Africa; East Asia; Eastern Europe; Eastern Sub-Saharan Africa; High-income Asia Pacific; High-income North America; North Africa and Middle East; Oceania; South Asia; Southeast Asia; Southern Latin America; Southern Sub-Saharan Africa; Tropical Latin America; Western Europe; Western Sub-Saharan Africa
Countries and territories (n=204)	Afghanistan, Albania, Algeria, American Samoa, Andorra, Angola, Antigua and Barbuda, Argentina, Armenia, Australia, Austria, Azerbaijan, Bahamas, Bahrain, Bangladesh, Barbados, Belarus, Belgium, Belize, Benin, Bermuda, Bhutan, Bolivia (Plurinational State of), Bosnia and Herzegovina, Botswana, Brazil, Brunei Darussalam, Bulgaria, Burkina Faso, Burundi, Côte d'Ivoire, Cabo Verde, Cambodia, Cameroon, Canada, Central African Republic, Chad, Chile, China, Colombia, Comoros, Congo, Cook Islands, Costa Rica, Croatia, Cuba, Cyprus, Czechia, Democratic People's Republic of Korea, Democratic Republic of the Congo, Denmark, Djibouti, Dominica, Dominican Republic, Ecuador, Egypt, El Salvador, Equatorial Guinea, Eritrea, Estonia, Eswatini, Ethiopia, Fiji, Finland, France, Gabon, Gambia, Georgia, Germany, Ghana, Greece, Greenland, Grenada, Guam, Guatemala, Guinea, Guinea-Bissau, Guyana, Haiti, Honduras, Hungary, Iceland, India, Indonesia, Iran (Islamic Republic of), Iraq, Ireland, Israel, Italy, Jamaica, Japan, Jordan, Kazakhstan, Kenya, Kiribati, Kuwait, Kyrgyzstan, Lao People's Democratic Republic, Latvia, Lebanon, Lesotho, Liberia, Libya, Lithuania, Luxembourg, Madagascar, Malawi, Malaysia, Maldives, Mali, Malta, Marshall Islands, Mauritania, Mauritius, Mexico, Micronesia (Federated States of), Monaco, Mongolia, Montenegro, Morocco, Mozambique, Myanmar, Namibia, Nauru, Nepal, Netherlands, New Zealand, Nicaragua, Niger, Nigeria, Niue, North Macedonia, Northern Mariana Islands, Norway, Oman, Pakistan, Palau, Palestine, Panama, Papua New Guinea, Paraguay, Peru, Philippines, Poland, Portugal, Puerto Rico, Qatar, Republic of Korea, Republic of Moldova, Romania, Russian Federation, Rwanda, Saint Kitts and Nevis, Saint Lucia, Saint Vincent and the Grenadines, Samoa, San Marino, Sao Tome and Principe, Saudi Arabia, Senegal, Serbia, Seychelles, Sierra Leone, Singapore, Slovakia, Slovenia, Solomon Islands, Somalia, South Africa, South Sudan, Spain, Sri Lanka, Sudan, Suriname, Sweden, Switzerland, Syrian Arab Republic, Taiwan (Province of China), Tajikistan, Thailand, Timor-Leste, Togo, Tokelau, Tonga, Trinidad and Tobago, Tunisia, Turkey, Turkmenistan, Tuvalu, Uganda, Ukraine, United Arab Emirates, United Kingdom, United Republic of Tanzania, United States of America, United States Virgin Islands, Uruguay, Uzbekistan, Vanuatu, Venezuela (Bolivarian Republic of), Vietnam, Yemen, Zambia, Zimbabwe
**Level 4 causes included in comparisons**	
Stroke types (n=3)	Intracerebral haemorrhage, Ischemic stroke, Subarachnoid haemorrhage
Neurological disorders (n=11)	Dementia, Epilepsy, Intracerebral haemorrhage, Ischemic stroke, Migraine, Motor neuron disease, Multiple sclerosis, Other neurological disorders, Parkinson's disease, Subarachnoid haemorrhage, Tension−type headache
Cardiovascular diseases (n=18)	Alcoholic cardiomyopathy, Aortic aneurysm, Atrial fibrillation and flutter, Endocarditis, Hypertensive heart disease, Intracerebral hemorrhage, Ischemic heart disease, Ischemic stroke, Lower extremity peripheral arterial disease, Myocarditis, Non-rheumatic calcific aortic valve disease, Non-rheumatic degenerative mitral valve disease, Other cardiomyopathies, Other cardiovascular and circulatory diseases, Other non-rheumatic valve diseases, Pulmonary Arterial Hypertension, Rheumatic heart disease, Subarachnoid hemorrhage
Non-communicable diseases (n=192)	Acne vulgaris, Acute glomerulonephritis, Acute lymphoid leukemia, Acute myeloid leukemia, Age-related and other hearing loss, Age-related macular degeneration, Alcohol use disorders, Alcoholic cardiomyopathy, Alopecia areata, Alzheimer's disease and other dementias, Amphetamine use disorders, Anorexia nervosa, Anxiety disorders, Aortic aneurysm, Appendicitis, Asbestosis, Asthma, Atopic dermatitis, Atrial fibrillation and flutter, Attention-deficit/hyperactivity disorder, Autism spectrum disorders, Benign prostatic hyperplasia, Bipolar disorder, Bladder cancer, Brain and central nervous system cancer, Breast cancer, Bulimia nervosa, Burkitt lymphoma, Cannabis use disorders, Caries of deciduous teeth, Caries of permanent teeth, Cataract, Cellulitis, Cervical cancer, Chronic hepatitis B including cirrhosis, Chronic hepatitis C including cirrhosis, Chronic kidney disease due to diabetes mellitus type 1, Chronic kidney disease due to diabetes mellitus type 2, Chronic kidney disease due to glomerulonephritis, Chronic kidney disease due to hypertension, Chronic kidney disease due to other and unspecified causes, Chronic lymphoid leukemia, Chronic myeloid leukemia, Chronic obstructive pulmonary disease, Cirrhosis due to alcohol, Cirrhosis due to other causes, Coal workers pneumoconiosis, Cocaine use disorders, Colon and rectum cancer, Conduct disorder, Congenital heart anomalies, Congenital musculoskeletal and limb anomalies, Contact dermatitis, Decubitus ulcer, Diabetes mellitus type 1, Diabetes mellitus type 2, Digestive congenital anomalies, Down syndrome, Dysthymia, Edentulism, Endocarditis, Endocrine, metabolic, blood, and immune disorders, Endometriosis, Esophageal cancer, Female infertility, Fungal skin diseases, G6PD deficiency, G6PD trait, Gallbladder and biliary diseases, Gallbladder and biliary tract cancer, Gastritis and duodenitis, Gastroesophageal reflux disease, Genital prolapse, Glaucoma, Gout, Hepatoblastoma, Hodgkin lymphoma, Hypertensive heart disease, Idiopathic developmental intellectual disability, Idiopathic epilepsy, Inflammatory bowel disease, Inguinal, femoral, and abdominal hernia, Interstitial lung disease and pulmonary sarcoidosis, Intracerebral haemorrhage, Ischemic heart disease, Ischemic stroke, Kidney cancer, Klinefelter syndrome, Larynx cancer, Lip and oral cavity cancer, Liver cancer due to alcohol use, Liver cancer due to hepatitis B, Liver cancer due to hepatitis C,

© 2025 GBD 2021 Global Subarachnoid Hemorrhage Risk Factors Collaborators. *JAMA Neurology*.

	Liver cancer due to NASH, Liver cancer due to other causes, Low back pain, Lower extremity peripheral arterial disease, Major depressive disorder, Male infertility, Malignant neoplasm of bone and articular cartilage, Malignant skin melanoma, Mesothelioma, Migraine, Motor neuron disease, Multiple myeloma, Multiple sclerosis, Myelodysplastic, myeloproliferative, and other hematopoietic neoplasms, Myocarditis, Nasopharynx cancer, Near vision loss, Neck pain, Neural tube defects, Neuroblastoma and other peripheral nervous cell tumors, Non-melanoma skin cancer (basal-cell carcinoma), Non-melanoma skin cancer (squamous-cell carcinoma), Non-rheumatic calcific aortic valve disease, Non-rheumatic degenerative mitral valve disease, Nonalcoholic fatty liver disease including cirrhosis, Opioid use disorders, Orofacial clefts, Osteoarthritis hand, Osteoarthritis hip, Osteoarthritis knee, Osteoarthritis other, Other benign and in situ neoplasms, Other cardiomyopathy, Other cardiovascular and circulatory diseases, Other chromosomal abnormalities, Other chronic respiratory diseases, Other congenital birth defects, Other digestive diseases, Other drug use disorders, Other eye cancers, Other gynecological diseases, Other hemoglobinopathies and hemolytic anemias, Other leukemia, Other malignant neoplasms, Other mental disorders, Other musculoskeletal disorders, Other neurological disorders, Other non-Hodgkin lymphoma, Other non-rheumatic valve diseases, Other oral disorders, Other pharynx cancer, Other pneumoconiosis, Other sense organ diseases, Other skin and subcutaneous diseases, Other urinary diseases, Other vision loss, Ovarian cancer, Pancreatic cancer, Pancreatitis, Paralytic ileus and intestinal obstruction, Parkinson's disease, Peptic ulcer disease, Periodontal diseases, Polycystic ovarian syndrome, Premenstrual syndrome, Prostate cancer, Pruritus, Psoriasis, Pulmonary Arterial Hypertension, Pyoderma, Refraction disorders, Retinoblastoma, Rheumatic heart disease, Rheumatoid arthritis, Scabies, Schizophrenia, Seborrheic dermatitis, Sickle cell disorders, Sickle cell trait, Silicosis, Soft tissue and other extraosseous sarcomas, Stomach cancer, Subarachnoid haemorrhage, Sudden infant death syndrome, Tension-type headache, Testicular cancer, Thalassemia, Thalassemia trait, Thyroid cancer, Tracheal, bronchus, and lung cancer, Turner syndrome, Urinary tract infections and interstitial nephritis, Urogenital congenital anomalies, Urolithiasis, Urticaria, Uterine cancer, Uterine fibroids, Vascular intestinal disorders, Viral skin diseases
All diseases (n=300)	Acne vulgaris, Acute glomerulonephritis, Acute hepatitis A, Acute hepatitis B, Acute hepatitis C, Acute hepatitis E, Acute lymphoid leukemia, Acute myeloid leukemia, Adverse effects of medical treatment, African trypanosomiasis, Age-related and other hearing loss, Age-related macular degeneration, Alcohol use disorders, Alcoholic cardiomyopathy, Alopecia areata, Alzheimer's disease and other dementias, Amphetamine use disorders, Anorexia nervosa, Anxiety disorders, Aortic aneurysm, Appendicitis, Asbestosis, Ascariasis, Asthma, Atopic dermatitis, Atrial fibrillation and flutter, Attention-deficit/hyperactivity disorder, Autism spectrum disorders, Benign prostatic hyperplasia, Bipolar disorder, Bladder cancer, Brain and central nervous system cancer, Breast cancer, Bulimia nervosa, Burkitt lymphoma, Cannabis use disorders, Caries of deciduous teeth, Caries of permanent teeth, Cataract, Cellulitis, Cervical cancer, Chagas disease, Chlamydial infection, Chronic hepatitis B including cirrhosis, Chronic hepatitis C including cirrhosis, Chronic kidney disease due to diabetes mellitus type 1, Chronic kidney disease due to diabetes mellitus type 2, Chronic kidney disease due to glomerulonephritis, Chronic kidney disease due to hypertension, Chronic kidney disease due to other and unspecified causes, Chronic lymphoid leukemia, Chronic myeloid leukemia, Chronic obstructive pulmonary disease, Cirrhosis due to alcohol, Cirrhosis due to other causes, Coal workers pneumoconiosis, Cocaine use disorders, Colon and rectum cancer, Conduct disorder, Conflict and terrorism, Congenital heart anomalies, Congenital musculoskeletal and limb anomalies, Contact dermatitis, COVID-19, Cutaneous and mucocutaneous leishmaniasis, Cyclist road injuries, Cystic echinococcosis, Cysticercosis, Decubitus ulcer, Dengue, Diabetes mellitus type 1, Diabetes mellitus type 2, Diarrheal diseases, Dietary iron deficiency, Digestive congenital anomalies, Diphtheria, Down syndrome, Drowning, Drug-susceptible tuberculosis, Dysthymia, Ebola, Ectopic pregnancy, Edentulism, Encephalitis, Endocarditis, Endocrine, metabolic, blood, and immune disorders, Endometriosis, Environmental heat and cold exposure, Esophageal cancer, Exposure to forces of nature, Extensively drug-resistant tuberculosis, Falls, Female infertility, Fire, heat, and hot substances, Food-borne trematodiases, Foreign body in eyes, Foreign body in other body part, Fungal skin diseases, G6PD deficiency, G6PD trait, Gallbladder and biliary diseases, Gallbladder and biliary tract cancer, Gastritis and duodenitis, Gastroesophageal reflux disease, Genital herpes, Genital prolapse, Glaucoma, Gonococcal infection, Gout, Guinea worm disease, Hemolytic disease and other neonatal jaundice, Hepatoblastoma, HIV/AIDS - Drug-susceptible Tuberculosis, HIV/AIDS - Extensively drug-resistant Tuberculosis, HIV/AIDS - Multidrug-resistant, Tuberculosis without extensive drug resistance, HIV/AIDS resulting in other diseases, Hodgkin lymphoma, Hookworm disease, Hypertensive heart disease, Idiopathic developmental intellectual disability, Idiopathic epilepsy, Indirect maternal deaths, Inflammatory bowel disease, Inguinal, femoral, and abdominal hernia, Interstitial lung disease and pulmonary sarcoidosis, Intracerebral haemorrhage, Invasive Non-typhoidal Salmonella (iNTS), Iodine deficiency, Ischemic heart disease, Ischemic stroke, Kidney cancer, Klinefelter syndrome, Larynx cancer, Late maternal deaths, Leprosy, Lip and oral cavity cancer, Liver cancer due to alcohol use, Liver cancer due to hepatitis B, Liver cancer due to hepatitis C, Liver cancer due to NASH, Liver cancer due to other causes, Low back pain, Lower extremity peripheral arterial disease, Lower respiratory infections, Lymphatic filariasis, Major depressive disorder, Malaria, Male infertility, Malignant neoplasm of bone and articular cartilage, Malignant skin melanoma, Maternal abortion and miscarriage, Maternal deaths aggravated by HIV/AIDS, Maternal haemorrhage, Maternal hypertensive disorders, Maternal obstructed labor and uterine rupture, Maternal sepsis and other maternal infections, Measles, Meningitis, Mesothelioma, Migraine, Motor neuron disease, Motor vehicle road injuries, Motorcyclist road injuries, Multidrug-resistant tuberculosis without extensive drug resistance, Multiple myeloma, Multiple sclerosis, Myelodysplastic, myeloproliferative, and other hematopoietic neoplasms, Myocarditis, Nasopharynx cancer, Near vision loss, Neck pain, Neonatal encephalopathy due to birth asphyxia and trauma, Neonatal preterm birth, Neonatal sepsis and other neonatal infections, Neural tube defects, Neuroblastoma and other peripheral nervous cell tumors, Non-melanoma skin cancer (basal-cell carcinoma), Non-melanoma skin cancer (squamous-cell carcinoma), Non-rheumatic calcific aortic valve disease, Non-rheumatic degenerative mitral valve disease, Non-venomous animal contact, Nonalcoholic fatty liver disease including cirrhosis, Onchocerciasis, Opioid use disorders, Orofacial clefts, Osteoarthritis hand, Osteoarthritis hip, Osteoarthritis knee, Osteoarthritis other, Other benign and in situ neoplasms, Other cardiomyopathy, Other cardiovascular and circulatory diseases, Other chromosomal abnormalities, Other

© 2025 GBD 2021 Global Subarachnoid Hemorrhage Risk Factors Collaborators. *JAMA Neurology*.

	chronic respiratory diseases, Other congenital birth defects, Other COVID-19 pandemic-related outcomes, Other digestive diseases, Other direct maternal disorders, Other drug use disorders, Other exposure to mechanical forces, Other eye cancers, Other gynecological diseases, Other hemoglobinopathies and hemolytic anemias, Other intestinal infectious diseases, Other leukemia, Other malignant neoplasms, Other mental disorders, Other musculoskeletal disorders, Other neglected tropical diseases, Other neonatal disorders, Other neurological disorders, Other non-Hodgkin lymphoma, Other non-rheumatic valve diseases, Other nutritional deficiencies, Other oral disorders, Other pharynx cancer, Other pneumoconiosis, Other road injuries, Other sense organ diseases, Other sexually transmitted infections, Other skin and subcutaneous diseases, Other transport injuries, Other unintentional injuries, Other unspecified infectious diseases, Other urinary diseases, Other vision loss, Otitis media, Ovarian cancer, Pancreatic cancer, Pancreatitis, Paralytic ileus and intestinal obstruction, Paratyphoid fever, Parkinson's disease, Pedestrian road injuries, Peptic ulcer disease, Periodontal diseases, Pertussis, Physical violence by firearm, Physical violence by other means, Physical violence by sharp object, Poisoning by carbon monoxide, Poisoning by other means, Police conflict and executions, Polycystic ovarian syndrome, Premenstrual syndrome, Prostate cancer, Protein-energy malnutrition, Pruritus, Psoriasis, Pulmonary Arterial Hypertension, Pulmonary aspiration and foreign body in airway, Pyoderma, Rabies, Refraction disorders, Retinoblastoma, Rheumatic heart disease, Rheumatoid arthritis, Scabies, Schistosomiasis, Schizophrenia, Seborrheic dermatitis, Self-harm by firearm, Self-harm by other specified means, Sexual violence, Sickle cell disorders, Sickle cell trait, Silicosis, Soft tissue and other extraosseous sarcomas, Stomach cancer, Subarachnoid haemorrhage, Sudden infant death syndrome, Syphilis, Tension-type headache, Testicular cancer, Tetanus, Thalassemias, Thalassemias trait, Thyroid cancer, Tracheal, bronchus, and lung cancer, Trachoma, Trichomoniasis, Trichuriasis, Turner syndrome, Typhoid fever, Unintentional firearm injuries, Upper respiratory, Urinary tract infections and interstitial nephritis, Urogenital congenital anomalies, Urolithiasis, Urticaria, Uterine cancer, Uterine fibroids, Varicella and herpes zoster, Vascular intestinal disorders, Venomous animal contact, Viral skin diseases, Visceral leishmaniasis, Vitamin A deficiency, Yellow fever, Zika virus

© 2025 GBD 2021 Global Subarachnoid Hemorrhage Risk Factors Collaborators. *JAMA Neurology*.

2.5 Supplementary References

1. GBD Stroke Collaborators. Global, regional, and national burden of stroke and its risk factors, 1990-2019: a systematic analysis for the Global Burden of Disease Study 2019. *Lancet Neurol*. Oct 2021;20(10):795-820. doi:10.1016/S1474-4422(21)00252-0
2. GBD Stroke Risk Factor Collaborators. Global, regional, and national burden of stroke and its risk factors, 1990-2021: a systematic analysis for the Global Burden of Disease Study 2021. *Lancet Neurol*. Oct 2024;23(10):973-1003. doi:10.1016/S1474-4422(24)00369-7
3. Institute for Health Metrics and Evaluation. Protocol for the Global Burden of Diseases, Injuries, and Risk Factors Study (GBD), version 4.0. 2020. http://www.healthdata.org/sites/default/files/files/Projects/GBD/March2020_GBD%20Protocol_v4.pdf (accessed July, 2024).
4. Stevens GA, Alkema L, Black RE, et al. Guidelines for Accurate and Transparent Health Estimates Reporting: the GATHER statement. *Lancet*. Dec 10 2016;388(10062):e19-e23. doi:10.1016/S0140-6736(16)30388-9
5. Etminan N, Chang HS, Hackenberg K, et al. Worldwide Incidence of Aneurysmal Subarachnoid Hemorrhage According to Region, Time Period, Blood Pressure, and Smoking Prevalence in the Population: A Systematic Review and Meta-analysis. *JAMA Neurol*. May 1 2019;76(5):588-597. doi:10.1001/jamaneurol.2019.0006
6. Mahlamaki K, Rautalin I, Korja M. Case Fatality Rates of Subarachnoid Hemorrhage Are Decreasing with Substantial between-Country Variation: A Systematic Review of Population-Based Studies between 1980 and 2020. *Neuroepidemiology*. 2022;56(6):402-412. doi:10.1159/000526983
7. GBD Causes of Death Collaborators. Global burden of 288 causes of death and life expectancy decomposition in 204 countries and territories and 811 subnational locations, 1990-2021: a systematic analysis for the Global Burden of Disease Study 2021. *Lancet*. Apr 3 2024;doi:10.1016/S0140-6736(24)00367-2
8. GBD Diseases Injuries, Collaborators. Global incidence, prevalence, years lived with disability (YLDs), disability-adjusted life-years (DALYs), and healthy life expectancy (HALE) for 371 diseases and injuries in 204 countries and territories and 811 subnational locations, 1990-2021: a systematic analysis for the Global Burden of Disease Study 2021. *Lancet*. May 18 2024;403(10440):2133-2161. doi:10.1016/S0140-6736(24)00757-8
9. Truelsen T, Krarup LH, Iversen HK, et al. Causes of Death Data in the Global Burden of Disease Estimates for Ischemic and Hemorrhagic Stroke. *Neuroepidemiology*. 2015;45(3):152-60. doi:10.1159/000441084
10. GBD Risk Factors Collaborators. Global burden and strength of evidence for 88 risk factors in 204 countries and 811 subnational locations, 1990-2021: a systematic analysis for the Global Burden of Disease Study 2021. *Lancet*. May 18 2024;403(10440):2162-2203. doi:10.1016/S0140-6736(24)00933-4

© 2025 GBD 2021 Global Subarachnoid Hemorrhage Risk Factors Collaborators. *JAMA Neurology*.

3. Supplementary Results

Supplementary Figure 5A-D. A) Incidence, B) prevalence, C) mortality, and D) disability-adjusted life year (DALY) rates of subarachnoid hemorrhage per 100,000 people by age and separately for men and women.

Solid lines represent the point estimates and shaded areas the 95% uncertainty intervals. EN = early neonatal (0-6 days); LN = late neonatal (7–27 days)

A) Incidence

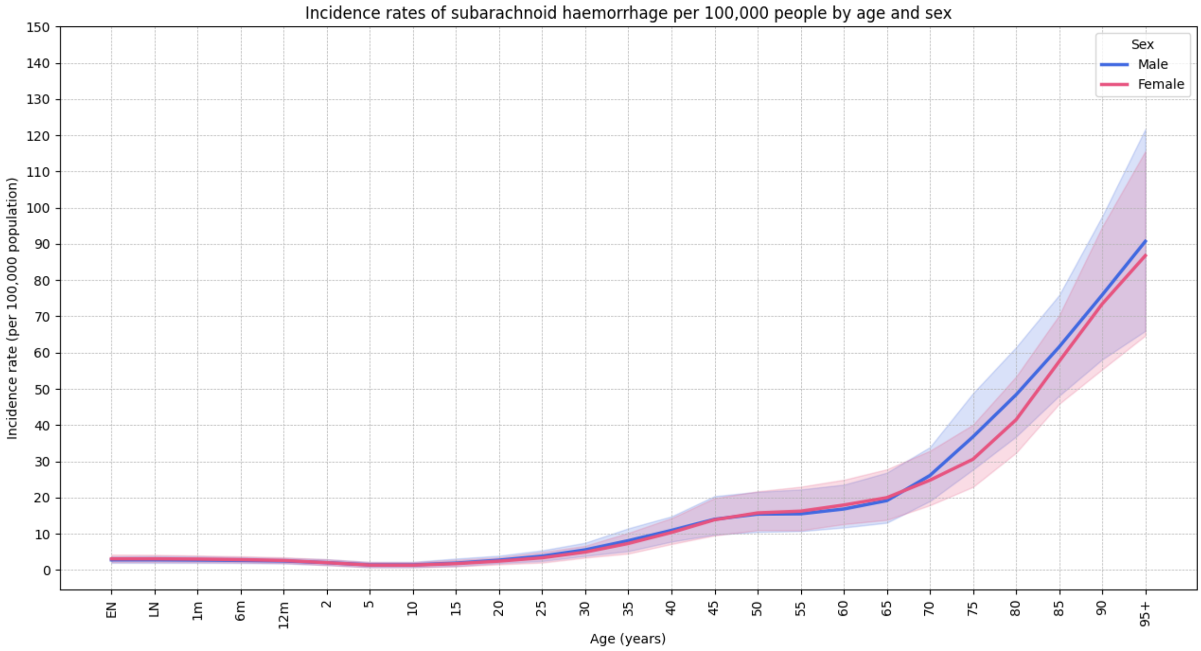

B) Prevalence

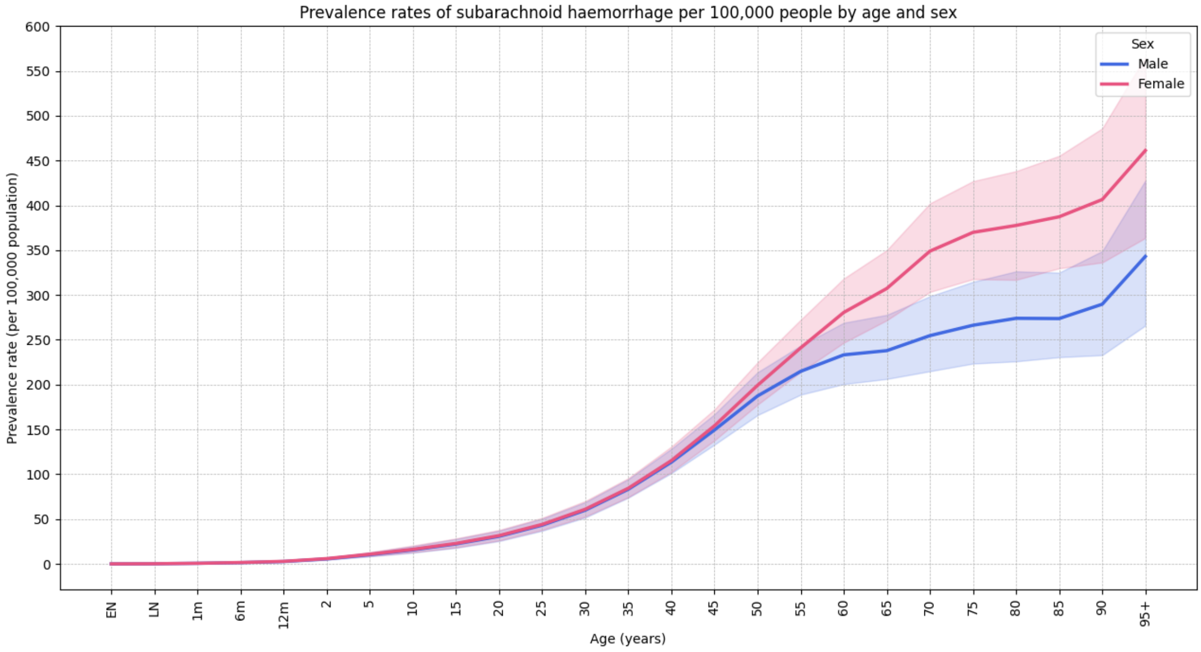

© 2025 GBD 2021 Global Subarachnoid Hemorrhage Risk Factors Collaborators. *JAMA Neurology*.

C) Mortality

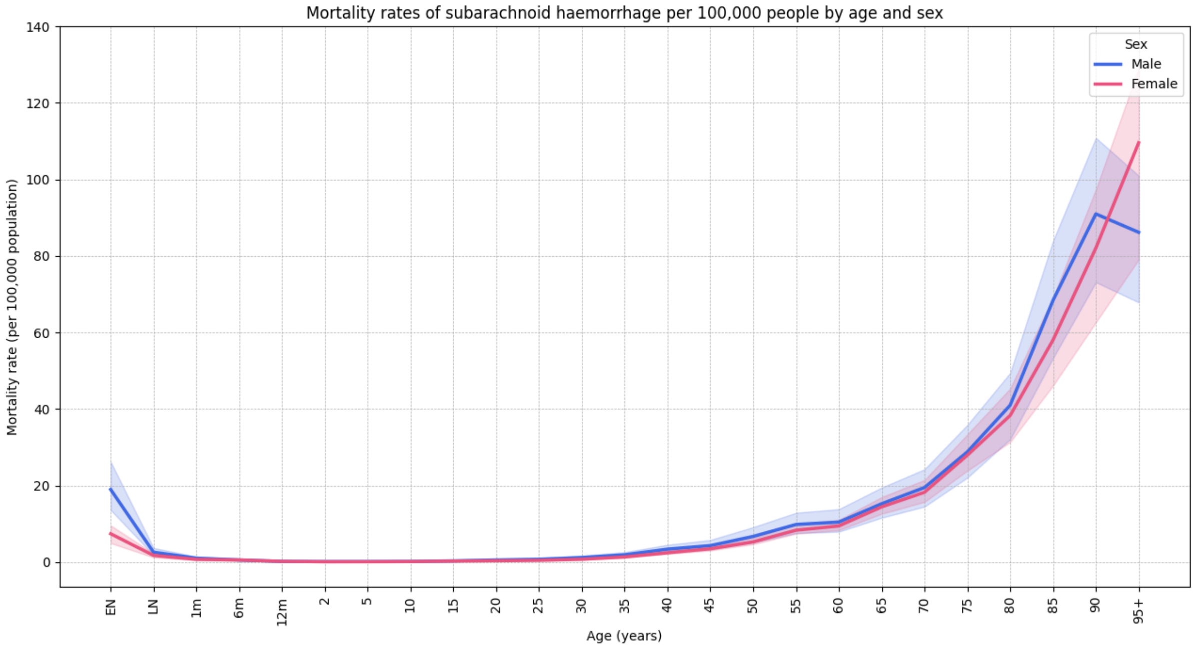

D) Disability-adjusted life years (DALYs)

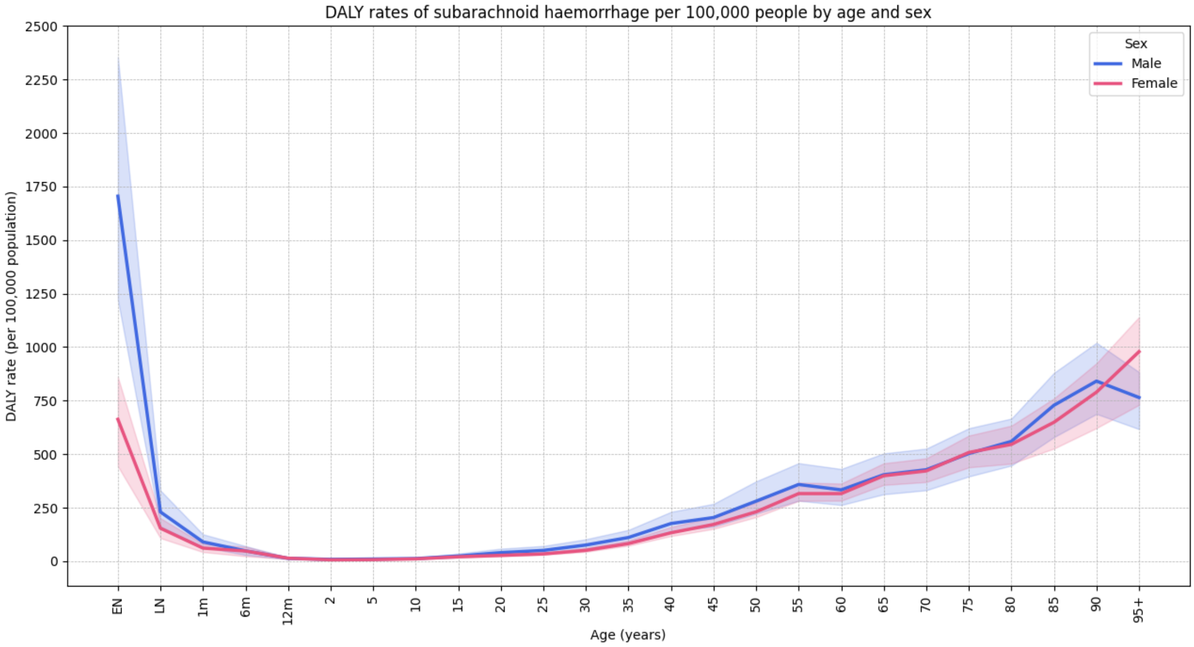

© 2025 GBD 2021 Global Subarachnoid Hemorrhage Risk Factors Collaborators. *JAMA Neurology*.

Supplementary Figure 6. Rankings of risk factors by their age-standardized population-attributable fractions on subarachnoid hemorrhage-related disability-adjusted life years (DALYs) in the world, five sociodemographic index (SDI) levels, seven GBD super-regions and 21 GBD regions.

	High systolic blood pressure	Smoking	Ambient particulate matter pollution	Household air pollution from solid fuels	Diet low in fruits	Diet high in sodium	Lead exposure	High body-mass index	Secondhand smoke	Low temperature	Diet low in fiber	Diet low in vegetables	High temperature	Diet high in red meat
Global	1	2	3	4	5	6	7	8	9	10	11	12	13	14
Low SDI	1	6	5	2	3	8	4	12	9	11	10	7	13	14
Low-middle SDI	1	3	4	2	5	7	6	10	9	13	8	11	12	14
Middle SDI	1	3	2	7	5	4	6	9	8	10	11	13	12	14
High-middle SDI	1	2	3	11	6	4	9	7	8	5	10	13	12	14
High SDI	1	2	4	13	3	6	10	5	9	7	8	12	11	14
Central Europe, Eastern Europe, and Central Asia	1	2	3	11	5	7	10	4	8	6	9	13	12	14
High-income	1	2	6	13	4	7	10	3	9	5	8	12	11	14
Latin America and Caribbean	1	2	4	8	5	7	6	3	10	11	9	12	13	14
North Africa and Middle East	1	3	2	9	8	12	5	4	6	7	11	13	10	14
South Asia	1	5	3	2	4	7	6	13	9	12	8	11	10	14
Southeast Asia, East Asia, and Oceania	1	3	2	5	6	4	7	11	8	9	10	13	12	14
Sub-Saharan Africa	1	6	4	2	3	9	5	8	10	11	12	7	13	14
Andean Latin America	1	4	2	10	5	8	7	3	12	6	9	11	13	14
Australasia	1	2	6	13	4	10	8	3	9	5	7	12	11	14
Caribbean	1	3	4	2	6	10	5	7	11	13	9	8	12	14
Central Asia	1	3	2	9	4	7	10	6	8	5	11	13	12	14
Central Europe	1	2	4	11	7	3	9	5	8	6	10	13	12	14
Central Latin America	1	4	3	8	5	6	7	2	10	9	11	12	13	14
Central Sub-Saharan Africa	1	7	5	2	4	11	6	8	10	12	9	3	13	14
East Asia	1	3	2	7	8	4	5	10	9	6	11	13	12	14
Eastern Europe	1	2	6	11	4	7	10	3	8	5	9	12	13	14
Eastern Sub-Saharan Africa	1	5	8	2	3	7	6	11	9	10	12	4	13	14
High-income Asia Pacific	1	2	3	12	5	4	9	10	8	6	7	13	11	14
High-income North America	1	2	8	13	4	6	10	3	9	5	7	12	11	14
North Africa and Middle East	1	3	2	9	8	12	5	4	6	7	11	13	10	14
Oceania	1	3	8	2	4	7	11	6	5	10	12	9	13	14
South Asia	1	5	3	2	4	7	6	13	9	12	8	11	10	14
Southeast Asia	1	2	3	4	6	5	9	10	8	13	7	11	12	14
Southern Latin America	1	2	3	11	6	7	10	4	9	5	8	13	12	14
Southern Sub-Saharan Africa	1	4	5	2	3	11	8	6	9	10	12	7	13	14
Tropical Latin America	1	2	4	11	5	6	7	3	9	10	8	12	13	14
Western Europe	1	2	6	13	5	7	10	4	9	3	8	11	12	14
Western Sub-Saharan Africa	1	6	3	2	4	8	5	7	11	13	12	9	10	14

© 2025 GBD 2021 Global Subarachnoid Hemorrhage Risk Factors Collaborators. *JAMA Neurology*.

Supplementary Figure 7. Rankings of risk factors by their age-standardized population-attributable fractions on SAH-related disability-adjusted life years (DALYs) in the world, by 5 sociodemographic index (SDI) levels, 7 GBD super-regions and 21 GBD regions, separately for men and women.

Source of figures: https://vizhub.healthdata.org/gbd-compare/

Males, Age-standardized, 2021, Percent of total DALYs

Higher rank — Lower rank

	Earth	Low SDI	Low Mid SDI	Mid SDI	High Mid SDI	High SDI	C & E Europe & C Asia	Latin Am & Caribbean	HI	MENA	S Asia	SE & E Asia & Oceania	Sub-Sah Africa	C Asia	C Europe	E Europe	Australasia	HI Asia Pac	HI N Am	S Latin Am	W Europe	Andean Latin Am	Caribbean	Central Latin Am	Trop Latin Am	C Sub-Sah Africa	E Sub-Sah Africa	S Sub-Sah Africa	W Sub-Sah Africa
**High blood pressure**	1	1	1	1	1	1	1	1	1	1	1	1	1	1	1	1	1	1	1	1	1	1	1	1	1	1	1	1	1
Smoking	2	3	3	2	2	2	2	2	2	2	3	2	3	2	2	2	2	2	2	2	2	3	3	2	2	3	3	2	5
Ambient particulate matter	3	6	4	3	3	5	3	7	3	3	4	3	5	3	4	7	7	4	8	3	6	2	4	4	4	6	7	5	3
High sodium	4	8	7	4	4	3	4	5	5	11	7	4	8	6	3	5	9	3	6	5	7	6	9	5	5	11	8	11	7
Household air pollution	5	2	2	7	11	13	11	13	10	9	2	6	2	8	11	11	13	13	13	11	13	11	2	10	11	2	2	3	2
Low fruit	6	4	5	5	6	4	5	3	6	7	5	7	4	4	7	3	4	5	4	7	5	5	6	7	6	5	4	4	4
Lead	7	5	6	6	8	10	10	10	7	5	6	5	6	9	9	10	6	9	10	10	10	8	5	6	7	7	6	6	6
Low temperature	8	11	13	8	5	7	6	6	11	6	12	8	11	5	5	4	5	6	5	6	3	7	13	8	10	12	10	9	13
Low fiber	9	10	8	9	10	8	9	8	8	12	8	9	12	11	10	9	8	7	7	9	9	9	10	11	9	8	11	12	12
High body-mass index	10	13	11	11	7	6	7	4	4	4	13	11	9	7	6	6	3	10	3	4	4	4	8	3	3	9	12	8	8
Secondhand smoke	11	9	9	10	9	9	8	9	9	8	9	10	10	10	8	8	10	8	9	8	8	12	11	9	8	10	9	10	11
Low vegetables	12	7	10	13	13	12	13	12	12	13	11	13	7	13	13	12	12	12	11	13	11	10	7	12	12	4	5	7	9
High temperature	13	12	12	12	12	11	12	11	13	10	10	12	13	12	12	13	11	11	12	12	12	13	12	13	13	13	13	13	10
High red meat	14	14	14	14	14	14	14	14	14	14	14	14	14	14	14	14	14	14	14	14	14	14	14	14	14	14	14	14	14

Females, Age-standardized, 2021, Percent of total DALYs

Higher rank — Lower rank

	Earth	Low SDI	Low Mid SDI	Mid SDI	High Mid SDI	High SDI	C & E Europe & C Asia	Latin Am & Caribbean	HI	MENA	S Asia	SE & E Asia & Oceania	Sub-Sah Africa	C Asia	C Europe	E Europe	Australasia	HI Asia Pac	HI N Am	S Latin Am	W Europe	Andean Latin Am	Caribbean	Central Latin Am	Trop Latin Am	C Sub-Sah Africa	E Sub-Sah Africa	S Sub-Sah Africa	W Sub-Sah Africa
**High blood pressure**	1	1	1	1	1	1	1	1	1	1	1	1	1	1	1	1	1	1	1	1	1	1	1	1	1	1	1	1	1
Ambient particulate matter	2	5	4	2	2	5	2	5	3	2	4	2	4	2	3	6	6	2	8	4	6	2	4	3	4	5	7	5	3
Household air pollution	3	2	2	5	11	13	11	13	7	8	2	4	2	7	11	11	13	12	13	11	13	8	2	8	10	2	2	2	2
Low fruit	4	3	3	4	6	4	5	4	5	6	3	6	3	4	7	4	4	3	4	5	5	4	5	4	5	4	3	3	4
High sodium	5	7	7	3	3	7	8	7	8	13	7	3	8	8	5	8	10	4	7	9	10	9	11	6	7	10	5	11	7
High body-mass index	6	10	9	8	5	3	3	3	2	3	9	10	7	3	4	2	3	10	3	3	3	3	3	2	3	7	8	4	6
Secondhand smoke	7	8	6	6	8	9	7	9	10	5	6	5	9	6	8	7	9	8	9	8	8	12	10	10	9	9	9	7	10
Lead	8	4	5	7	9	10	10	10	6	4	5	7	6	9	9	10	8	9	10	10	9	6	6	5	6	6	6	9	5
Smoking	9	11	10	11	4	2	4	2	4	9	11	11	10	11	2	3	2	5	2	2	2	11	8	7	2	11	11	10	11
Low temperature	10	12	13	9	7	6	6	6	11	7	13	8	11	5	6	5	5	6	5	6	4	5	13	9	11	12	10	8	13
Low fiber	11	9	8	10	10	8	9	8	9	11	8	9	12	10	10	9	7	7	6	7	7	7	9	11	8	8	12	12	12
Low vegetables	12	6	11	13	13	12	13	12	12	12	12	12	5	13	13	12	12	13	12	13	11	10	7	12	12	3	4	6	8
High temperature	13	13	12	12	12	11	12	11	13	10	10	13	13	12	12	13	11	11	11	12	12	13	12	13	13	13	13	13	9
High red meat	14	14	14	14	14	14	14	14	14	14	14	14	14	14	14	14	14	14	14	14	14	14	14	14	14	14	14	14	14

© 2025 GBD 2021 Global Subarachnoid Hemorrhage Risk Factors Collaborators. *JAMA Neurology*.

Supplementary Figure 8. Age-standardized population-attributable fractions (PAFs) of A) environmental/occupational risks, B) behavioral risks, and C) metabolic risks on disability-adjusted life years related to subarachnoid hemorrhage in 204 countries and territories of the world in 2021.

The circles above the scales represent the estimates from individual countries. Source of figures: https://vizhub.healthdata.org/gbd-compare/

A) Environmental/occupational risks

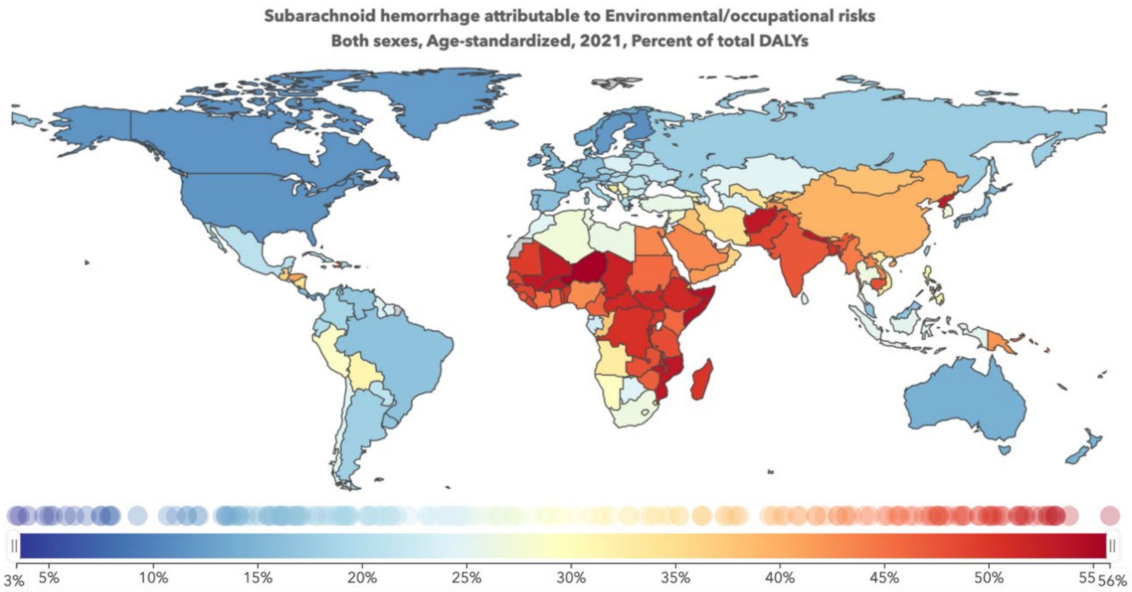

B) Behavioral risks

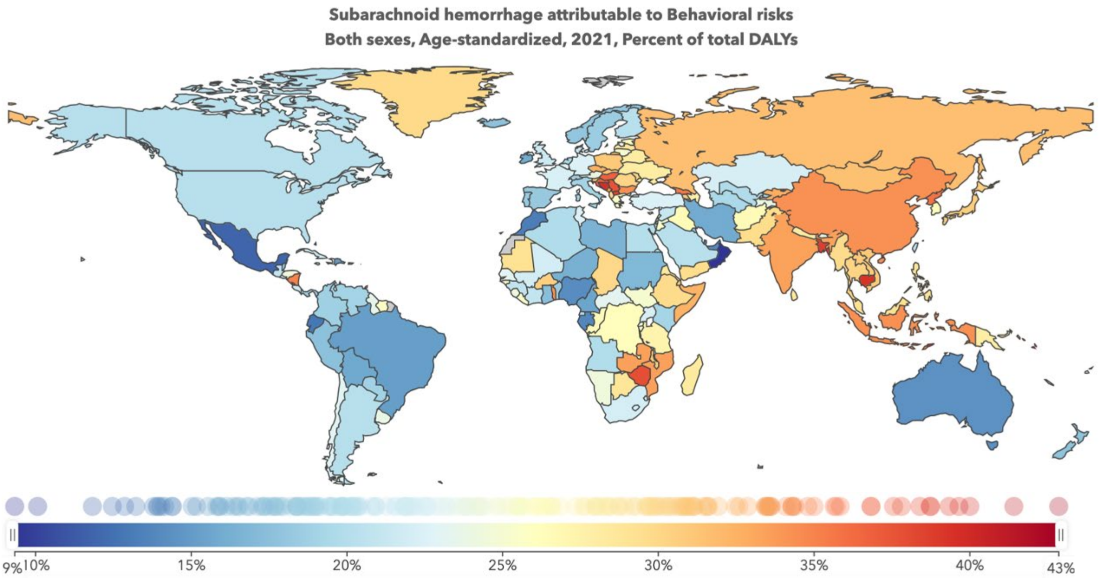

© 2025 GBD 2021 Global Subarachnoid Hemorrhage Risk Factors Collaborators. *JAMA Neurology*.

C) Metabolic risks

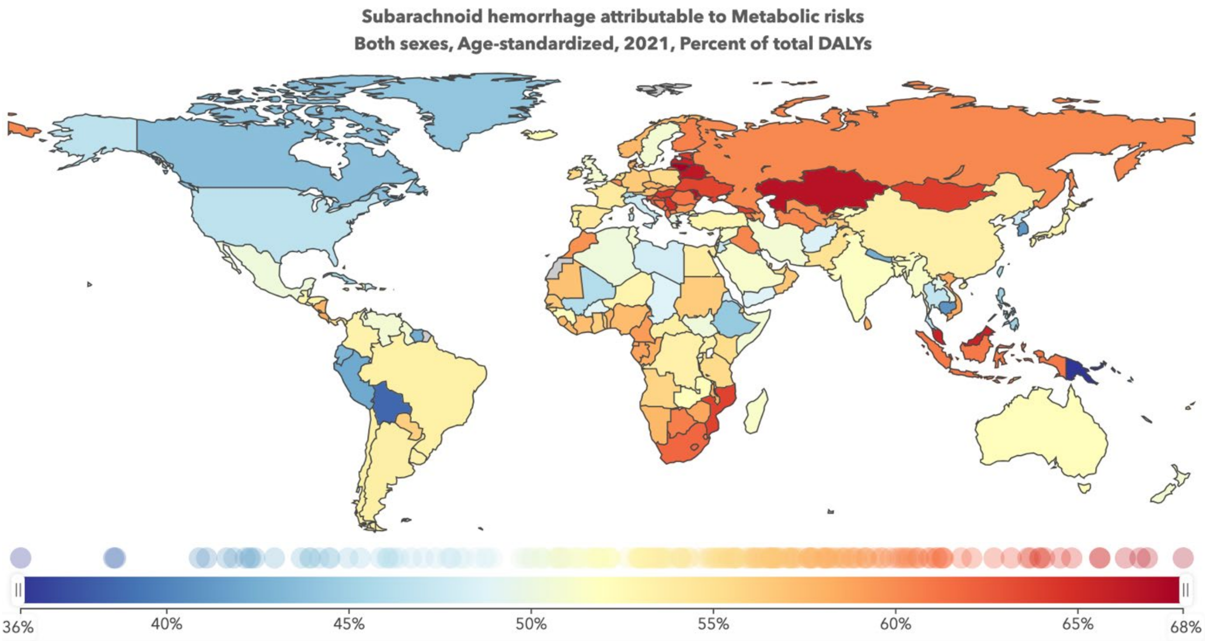

© 2025 GBD 2021 Global Subarachnoid Hemorrhage Risk Factors Collaborators. *JAMA Neurology*.

Supplementary Figure 9. Rankings of causes of death in the age group of 20-54 years across 24 Western European countries in 2021.

Source of figure: https://vizhub.healthdata.org/gbd-compare/

Both sexes, 20-54 years, 2021, Deaths

Higher rank — Lower rank

	**Finland**	Greece	Ireland	Portugal	France	W Europe	Norway	Spain	United Kingdom	Austria	Andorra	Germany	Cyprus	Denmark	Belgium	Netherlands	Iceland	Italy	Malta	Sweden	Luxembourg	Monaco	San Marino	Switzerland	Israel
Self-harm other means	1	8	2	3	1	2	1	4	2	2	3	2	5	1	1	1	1	5	5	1	2	3	3	1	3
Ischemic heart disease	2	1	3	2	5	3	2	2	3	3	4	3	1	2	4	5	2	2	1	3	4	5	6	4	5
Opioid use disorders	3	10	6	32	13	8	3	15	4	10	100	11	15	6	13	16	4	31	11	4	10	89	77	6	20
Alcohol use disorders	4	62	18	30	7	11	11	49	11	9	62	7	41	4	8	12	8	61	45	9	7	66	58	11	28
Breast cancer	5	4	5	5	4	5	5	5	5	5	5	5	3	5	5	4	5	3	3	5	5	4	5	5	2
Lung cancer	6	3	4	4	3	4	4	3	6	4	2	4	4	3	3	3	3	4	4	7	3	1	2	3	4
COVID-19	7	2	1	1	2	1	8	1	1	1	1	1	2	7	2	2	56	1	2	2	1	2	1	2	1
Cirrhosis alcohol	8	15	11	8	12	9	22	10	8	7	11	8	12	10	12	25	30	19	13	14	9	10	9	14	23
Falls	9	21	19	16	9	13	14	14	16	11	17	17	11	13	10	13	9	17	9	16	13	30	21	9	26
Brain cancer	10	5	8	10	8	7	7	7	10	8	6	9	7	9	7	7	6	7	6	8	8	8	4	8	8
Colorectal cancer	11	9	7	6	6	6	6	6	7	6	9	6	9	8	6	6	10	6	7	6	6	7	7	7	7
Pancreatic cancer	12	11	9	13	10	10	9	9	13	12	10	10	17	12	11	9	12	8	8	10	12	9	12	12	11
**Subarachnoid hem**	13	16	16	17	18	18	19	19	19	20	20	20	21	21	22	22	25	26	26	26	27	28	29	29	40
Self-harm by firearm	14	38	46	45	16	39	12	59	103	16	48	51	32	34	33	83	15	44	52	25	30	15	13	10	13
Chronic hepatitis C	15	35	26	19	23	21	39	18	37	17	18	16	25	22	21	36	42	15	21	40	16	22	17	32	34
Alcoholic cardiomyopathy	16	117	64	86	85	62	85	88	62	46	46	37	95	82	80	65	101	113	109	58	72	33	61	58	135
Motor vehicle road inj	17	6	15	12	11	12	16	8	31	14	8	15	6	14	9	15	7	9	20	13	11	16	14	15	9
Intracerebral hem	18	7	29	9	22	15	28	12	20	23	22	18	16	15	17	24	29	11	10	20	14	11	15	34	15
Other lymphoma	19	31	20	22	24	23	21	22	24	22	14	29	23	31	27	27	19	16	15	23	22	19	11	19	14
Other drug use disorders	20	73	12	67	63	41	20	56	18	59	130	48	68	20	48	68	13	91	35	12	31	92	174	36	32
Other neurological	21	26	33	50	29	31	34	32	30	38	31	32	35	40	25	19	14	30	17	17	43	41	52	21	35
Melanoma	22	34	22	39	32	26	10	30	25	18	27	28	29	17	26	11	16	20	25	11	24	29	18	16	18
Idiopathic epilepsy	23	36	21	24	20	24	13	34	22	27	33	23	33	25	15	21	24	27	28	18	15	55	81	17	21
Drowning	24	19	27	47	38	47	30	38	68	51	153	62	13	55	46	45	33	50	37	42	69	24	74	46	43
Other transport inj	25	42	55	29	43	55	24	52	86	44	12	53	22	58	53	81	11	52	47	47	70	35	79	27	82

© 2025 GBD 2021 Global Subarachnoid Hemorrhage Risk Factors Collaborators. *JAMA Neurology*.

Supplementary Figure 10. Changes in the Incidence, Prevalence, Deaths, and Disability-Adjusted Life-Years (DALYs) of Subarachnoid Hemorrhage in the World Between 1990 (Deaths Since 1980) and 2021

A) Absolute numbers

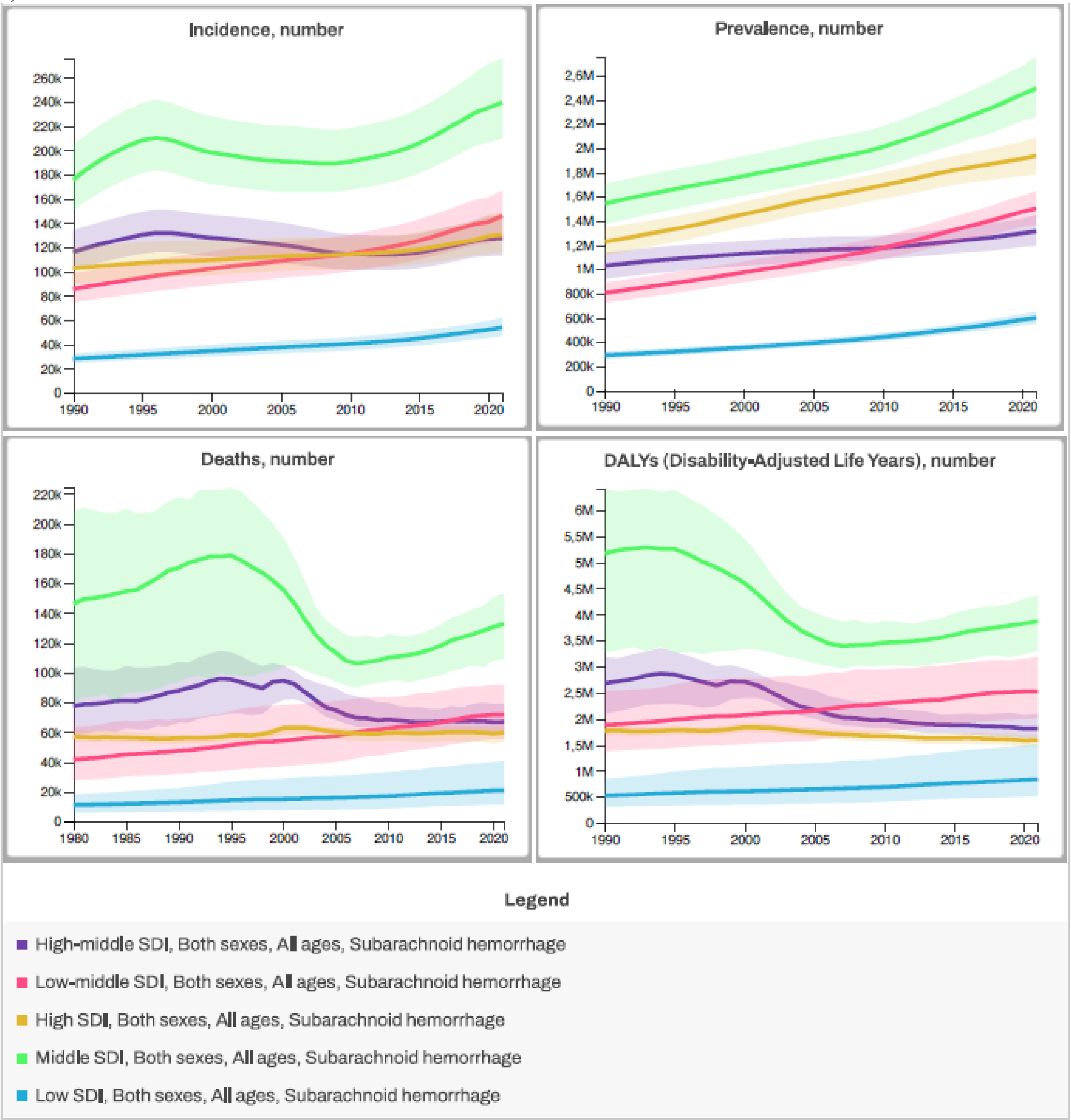

Results are presented as absolute number (A) and age-standardized rates (B) per 100 000 people as well as stratified by Sociodemographic Index levels. Solid lines represent the changes in point estimates, and shaded areas represent 95% uncertainty intervals. Figures created with The Institute for Health Metrics and Evaluation, Global Burden of Diseases Study 2021.

© 2025 GBD 2021 Global Subarachnoid Hemorrhage Risk Factors Collaborators. *JAMA Neurology*.

B) Age-standardized rates

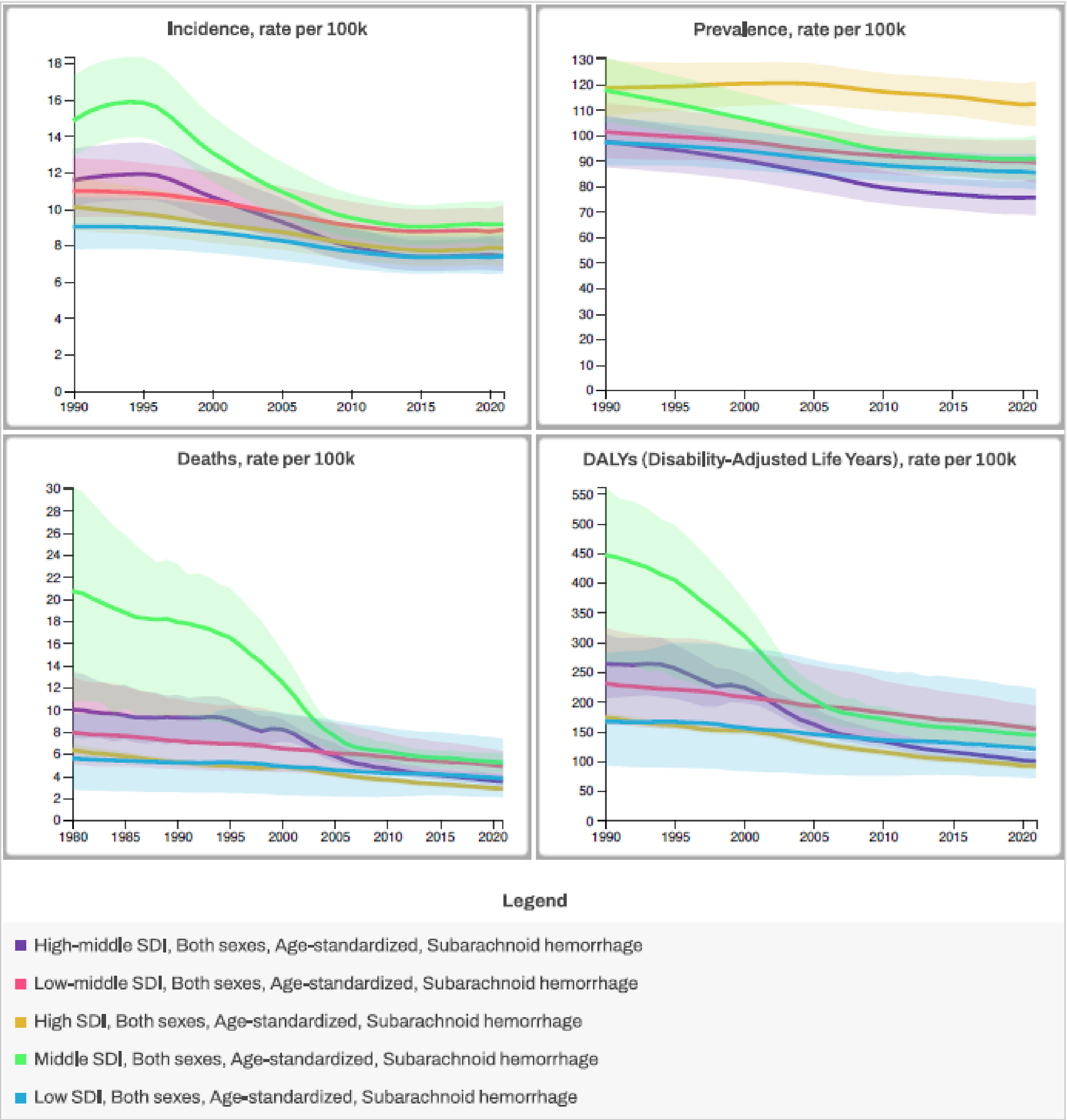

Results are presented as absolute number (A) and age-standardized rates (B) per 100 000 people as well as stratified by Sociodemographic Index levels. Solid lines represent the changes in point estimates, and shaded areas represent 95% uncertainty intervals. Figures created with The Institute for Health Metrics and Evaluation, Global Burden of Diseases Study 2021.

© 2025 GBD 2021 Global Subarachnoid Hemorrhage Risk Factors Collaborators. *JAMA Neurology*.

Supplementary Table 4. Age-standardized incidence, prevalence, mortality, and disability-adjusted life years (DALY) rates (with 95% uncertainty intervals) of subarachnoid hemorrhage per 100,000 people for 204 countries and territories in 2021.

	Incidence	Prevalence	Mortality	DALY rate
Afghanistan	8.88 (7.88–10.14)	83.19 (76.37–89.93)	6.60 (3.24–11.83)	186.26 (101.62–309.66)
Albania	6.73 (5.93–7.56)	67.44 (62.26–73.26)	2.49 (1.61–3.46)	69.05 (50.78–91.71)
Algeria	6.27 (5.48–7.07)	77.49 (71.82–83.72)	2.60 (1.72–3.95)	69.50 (48.57–99.79)
American Samoa	11.56 (10.39–12.83)	151.63 (143.48–159.82)	6.03 (4.83–7.57)	202.28 (164.02–251.73)
Andorra	6.10 (5.27–6.98)	84.59 (78.82–90.33)	1.80 (1.22–2.44)	57.46 (41.75–75.52)
Angola	6.31 (5.46–7.40)	85.41 (79.00–91.44)	2.74 (1.43–5.16)	88.71 (50.99–154.18)
Antigua and Barbuda	10.34 (9.19–11.48)	139.56 (131.03–147.42)	3.99 (3.71–4.30)	118.60 (110.98–127.41)
Argentina	10.86 (9.70–12.25)	125.28 (115.71–137.87)	4.06 (3.72–4.34)	134.14 (124.15–143.62)
Armenia	6.16 (5.46–6.90)	71.14 (65.98–76.17)	2.34 (2.08–2.64)	71.12 (63.51–80.44)
Australia	6.11 (5.37–6.92)	81.22 (75.77–87.05)	2.38 (2.13–2.59)	70.06 (64.90–75.38)
Austria	8.86 (7.58–10.19)	150.16 (141.07–158.41)	2.22 (1.98–2.42)	71.68 (64.60–78.56)
Azerbaijan	5.85 (5.03–6.68)	67.09 (61.95–72.60)	1.10 (0.74–1.57)	39.40 (29.64–51.87)
Bahamas	8.97 (7.98–9.97)	122.41 (115.44–129.67)	3.31 (2.70–4.08)	115.57 (94.93–141.03)
Bahrain	4.48 (3.90–5.04)	59.98 (54.83–65.34)	1.99 (1.45–2.51)	52.26 (41.52–63.45)
Bangladesh	12.74 (11.31–14.63)	122.47 (114.19–132.31)	7.93 (4.69–13.16)	243.46 (154.02–386.81)
Barbados	10.24 (9.21–11.50)	140.97 (132.99–149.01)	4.69 (3.74–5.82)	144.16 (114.34–179.24)
Belarus	7.36 (6.57–8.19)	71.75 (66.81–77.59)	3.93 (3.19–4.68)	123.73 (101.18–146.64)
Belgium	5.72 (5.02–6.53)	71.18 (65.73–77.18)	2.31 (2.09–2.49)	67.19 (62.95–72.38)
Belize	8.43 (7.49–9.44)	115.01 (108.03–121.97)	2.73 (2.39–3.10)	92.36 (81.22–103.89)
Benin	5.41 (4.70–6.35)	84.69 (79.07–90.95)	2.60 (1.22–6.16)	84.74 (44.30–183.61)
Bermuda	8.64 (7.54–9.71)	141.44 (134.53–149.13)	2.24 (1.89–2.70)	72.39 (61.63–86.07)
Bhutan	7.64 (6.68–8.71)	78.42 (72.16–84.84)	3.68 (2.44–5.58)	114.23 (78.27–170.02)
Bolivia (Plurinational State of)	14.43 (12.86–16.20)	171.84 (161.82–181.39)	9.70 (6.98–13.63)	300.13 (219.35–411.66)
Bosnia and Herzegovina	7.73 (6.93–8.59)	76.59 (70.71–82.43)	3.25 (2.38–4.21)	102.57 (77.01–130.54)
Botswana	5.86 (5.12–6.74)	79.12 (73.08–85.24)	1.34 (0.93–2.21)	46.75 (33.73–72.24)
Brazil	10.20 (8.84–11.81)	108.15 (96.34–121.68)	5.45 (5.12–5.68)	177.61 (169.76–184.33)
Brunei Darussalam	15.60 (13.78–17.87)	172.32 (162.82–182.90)	7.00 (5.73–8.50)	224.11 (188.53–267.80)
Bulgaria	8.32 (7.54–9.13)	75.93 (70.11–81.65)	5.09 (4.38–5.86)	154.16 (132.14–178.07)
Burkina Faso	5.00 (4.31–5.83)	80.47 (74.73–86.94)	2.25 (0.82–5.81)	76.70 (34.78–176.90)
Burundi	7.99 (6.86–9.49)	100.87 (93.66–107.96)	3.76 (0.85–13.92)	119.38 (37.30–389.51)
Côte d'Ivoire	5.07 (4.37–5.84)	81.15 (75.22–87.32)	2.45 (1.19–5.34)	87.78 (48.28–175.32)
Cabo Verde	5.18 (4.36–6.08)	91.15 (84.72–97.56)	1.66 (0.81–3.81)	62.53 (36.17–135.13)
Cambodia	10.80 (9.55–12.18)	102.64 (95.11–109.79)	5.88 (4.20–10.09)	165.54 (122.80–242.74)
Cameroon	5.27 (4.56–6.14)	84.35 (78.01–90.64)	2.80 (1.27–6.19)	97.33 (48.64–195.34)
Canada	7.03 (6.11–8.04)	103.20 (96.64–109.98)	2.36 (2.13–2.55)	76.13 (69.97–82.49)
Central African Republic	7.50 (6.45–8.84)	89.29 (81.88–96.53)	4.49 (1.55–11.58)	141.68 (57.82–314.50)
Chad	5.67 (4.90–6.60)	89.41 (83.10–95.41)	3.45 (1.34–8.47)	117.76 (52.85–264.50)
Chile	9.61 (8.46–10.82)	111.39 (103.59–121.03)	3.79 (3.50–4.08)	120.38 (112.85–129.21)
China	7.81 (6.88–8.95)	68.88 (61.53–76.90)	4.72 (3.45–5.95)	115.49 (86.86–142.50)
Colombia	13.97 (12.55–15.71)	178.26 (168.44–187.12)	6.09 (5.08–7.19)	184.51 (155.49–217.65)
Comoros	7.36 (6.39–8.62)	101.07 (94.05–108.35)	2.63 (1.07–6.33)	88.71 (43.07–188.52)
Congo	6.17 (5.35–7.09)	82.64 (76.65–88.61)	2.69 (1.45–5.27)	87.67 (51.87–155.51)
Cook Islands	9.90 (8.77–11.05)	152.97 (145.53–160.75)	2.83 (1.87–3.90)	113.54 (86.33–144.32)
Costa Rica	10.25 (8.95–11.66)	155.37 (146.79–163.67)	3.65 (3.16–4.04)	117.80 (104.96–129.08)
Croatia	5.93 (5.34–6.60)	64.96 (59.48–70.46)	3.39 (2.95–3.91)	93.44 (82.06–106.54)
Cuba	8.44 (7.42–9.54)	130.94 (124.33–137.57)	2.79 (2.39–3.17)	91.85 (80.29–103.71)
Cyprus	7.01 (6.13–7.98)	87.96 (81.91–93.66)	2.65 (2.11–3.36)	68.28 (56.81–81.96)
Czechia	6.11 (5.35–6.81)	72.88 (67.04–78.27)	2.30 (2.01–2.64)	73.74 (64.69–84.50)
Democratic People's Republic of Korea	11.65 (10.29–13.04)	99.29 (92.31–107.05)	7.72 (4.65–14.04)	216.33 (136.18–370.31)
Democratic Republic of the Congo	7.75 (6.67–9.12)	97.68 (90.79–104.81)	3.30 (1.08–10.89)	102.20 (42.99–302.44)
Denmark	7.42 (6.51–8.47)	98.92 (92.58–105.51)	2.73 (2.47–2.95)	76.66 (70.56–81.99)
Djibouti	7.49 (6.45–8.73)	101.23 (94.54–108.39)	2.83 (1.15–6.26)	94.58 (44.72–194.76)
Dominica	8.99 (7.91–10.12)	124.56 (117.12–132.60)	4.42 (3.56–5.39)	136.67 (113.11–166.88)
Dominican Republic	10.97 (9.68–12.47)	141.84 (133.15–150.07)	4.04 (2.98–5.27)	146.74 (114.48–185.95)
Ecuador	14.98 (13.58–16.91)	199.81 (189.65–209.28)	7.69 (6.14–9.47)	234.98 (190.75–288.98)
Egypt	5.52 (4.84–6.20)	65.46 (60.09–71.47)	2.99 (2.05–4.36)	83.75 (60.65–119.58)
El Salvador	9.75 (8.67–10.93)	134.70 (127.09–142.95)	3.37 (2.57–4.34)	118.51 (93.68–147.91)
Equatorial Guinea	5.30 (4.51–6.21)	74.12 (68.51–79.92)	1.56 (0.78–2.63)	54.32 (30.08–83.95)
Eritrea	8.20 (7.10–9.67)	107.71 (100.62–115.04)	3.84 (1.45–9.04)	127.23 (54.59–272.81)
Estonia	6.36 (5.57–7.12)	66.76 (62.09–71.95)	2.41 (2.08–2.71)	70.24 (61.16–79.30)
Eswatini	5.57 (4.90–6.44)	72.43 (67.17–78.35)	2.32 (1.50–3.70)	75.24 (48.72–118.52)
Ethiopia	6.55 (5.60–7.73)	84.80 (75.26–95.06)	2.15 (0.70–6.65)	70.39 (29.41–189.81)
Fiji	16.55 (15.02–18.22)	183.09 (172.52–193.13)	7.83 (5.97–10.32)	254.88 (198.88–321.88)
Finland	10.27 (9.00–11.74)	160.71 (152.76–168.90)	3.35 (3.00–3.61)	110.60 (102.95–119.05)
France	6.48 (5.75–7.26)	87.12 (81.54–93.16)	2.09 (1.89–2.25)	66.55 (61.47–71.66)

© 2025 GBD 2021 Global Subarachnoid Hemorrhage Risk Factors Collaborators. *JAMA Neurology*.

Gabon	5.96 (5.18–6.97)	83.14 (76.87–89.19)	1.95 (1.16–3.29)	64.90 (41.12–105.68)
Gambia	5.27 (4.56–6.15)	85.35 (79.45–91.70)	3.05 (1.31–7.59)	105.25 (51.58–242.97)
Georgia	16.12 (14.71–17.80)	136.10 (127.09–144.83)	8.83 (7.26–10.49)	244.00 (201.68–286.44)
Germany	6.30 (5.42–7.26)	93.33 (86.78–99.32)	2.17 (1.94–2.34)	69.11 (64.47–74.57)
Ghana	6.08 (5.21–7.10)	101.35 (95.05–108.14)	3.19 (1.70–6.19)	113.34 (64.95–211.59)
Greece	7.20 (6.30–8.23)	105.45 (98.72–111.73)	2.86 (2.60–3.05)	86.72 (80.58–93.76)
Greenland	11.62 (10.55–12.93)	111.87 (103.98–120.19)	7.53 (5.65–9.37)	231.20 (182.48–279.81)
Grenada	10.27 (9.19–11.49)	134.96 (127.79–142.43)	4.67 (4.08–5.27)	146.31 (126.09–165.44)
Guam	10.11 (8.99–11.25)	154.07 (146.53–161.85)	2.95 (2.46–3.46)	136.77 (117.70–159.68)
Guatemala	8.94 (8.00–10.10)	127.40 (120.04–134.47)	2.53 (2.16–2.91)	93.75 (80.59–107.15)
Guinea	6.05 (5.19–7.05)	94.71 (88.42–101.56)	3.04 (1.32–6.88)	107.15 (55.14–216.70)
Guinea-Bissau	5.68 (4.91–6.62)	88.00 (81.37–95.10)	4.41 (1.85–9.87)	151.44 (70.21–320.57)
Guyana	11.29 (10.21–12.57)	129.84 (121.80–138.50)	5.51 (4.26–6.96)	191.24 (147.74–242.52)
Haiti	14.75 (13.08–16.88)	150.03 (139.90–160.19)	12.78 (6.59–20.85)	434.98 (237.15–658.46)
Honduras	12.35 (11.06–13.91)	141.46 (132.68–150.27)	11.86 (8.47–15.42)	340.96 (235.34–447.31)
Hungary	5.99 (5.33–6.64)	67.13 (61.82–72.24)	2.17 (1.84–2.50)	75.62 (65.06–86.61)
Iceland	7.24 (6.34–8.32)	104.27 (97.73–110.61)	2.05 (1.76–2.27)	62.92 (56.80–69.61)
India	7.70 (6.65–8.95)	71.80 (63.07–81.19)	3.95 (2.73–5.50)	125.93 (91.47–167.76)
Indonesia	10.94 (9.54–12.64)	109.09 (96.70–122.68)	6.53 (4.72–10.63)	198.84 (154.02–287.20)
Iran (Islamic Republic of)	4.76 (4.15–5.41)	61.11 (54.85–67.86)	1.40 (1.05–1.69)	40.90 (34.14–48.29)
Iraq	5.11 (4.47–5.76)	66.00 (60.66–71.90)	2.36 (1.69–3.07)	74.78 (56.72–97.71)
Ireland	7.53 (6.64–8.52)	101.09 (94.48–107.32)	2.68 (2.36–2.94)	74.87 (68.89–81.33)
Israel	5.65 (4.84–6.54)	80.75 (74.99–86.50)	1.33 (1.18–1.45)	41.16 (37.50–44.92)
Italy	4.84 (4.26–5.39)	62.77 (56.25–69.75)	1.87 (1.66–2.01)	52.22 (48.49–55.95)
Jamaica	11.67 (10.55–13.08)	158.01 (149.38–166.71)	4.75 (3.68–6.04)	149.99 (116.73–190.13)
Japan	15.83 (13.71–18.40)	219.60 (196.31–244.74)	4.21 (3.76–4.49)	150.89 (138.85–162.62)
Jordan	4.69 (4.03–5.37)	68.52 (63.46–74.40)	0.51 (0.36–0.65)	24.18 (19.26–29.41)
Kazakhstan	10.15 (9.12–11.28)	87.86 (81.37–94.49)	6.46 (5.60–7.37)	169.52 (145.47–193.34)
Kenya	6.46 (5.58–7.60)	86.04 (76.66–96.12)	2.07 (0.93–5.05)	68.12 (35.50–148.42)
Kiribati	22.01 (19.58–24.58)	241.17 (227.04–255.27)	9.04 (6.61–13.07)	349.55 (258.11–479.78)
Kuwait	5.55 (4.76–6.31)	79.71 (73.79–85.55)	0.51 (0.42–0.61)	25.93 (21.96–30.34)
Kyrgyzstan	9.56 (8.55–10.68)	91.73 (84.64–97.80)	6.13 (5.19–7.21)	186.36 (156.23–220.95)
Lao People's Democratic Republic	9.87 (8.74–11.12)	99.12 (92.03–106.28)	5.68 (4.03–8.68)	183.57 (135.18–261.22)
Latvia	7.23 (6.39–8.05)	70.86 (65.84–76.29)	3.14 (2.75–3.56)	93.12 (81.10–106.48)
Lebanon	5.55 (4.80–6.29)	78.30 (72.45–84.33)	2.06 (1.62–2.58)	61.49 (51.22–73.63)
Lesotho	6.46 (5.64–7.39)	77.09 (70.95–83.55)	3.49 (2.08–5.80)	106.98 (65.68–176.90)
Liberia	5.51 (4.73–6.47)	86.68 (80.66–93.68)	2.91 (0.97–9.10)	101.07 (41.10–284.17)
Libya	5.09 (4.47–5.76)	68.65 (63.05–74.14)	2.15 (1.29–3.35)	79.74 (49.55–120.40)
Lithuania	6.75 (5.96–7.56)	68.70 (63.95–74.13)	3.03 (2.64–3.41)	91.73 (80.30–102.93)
Luxembourg	5.22 (4.50–6.00)	67.94 (62.75–73.57)	1.58 (1.41–1.76)	45.54 (41.35–50.45)
Madagascar	9.87 (8.43–11.47)	130.43 (121.99–139.49)	5.37 (1.86–15.13)	182.17 (73.64–469.84)
Malawi	6.95 (5.91–8.15)	87.85 (81.48–94.39)	3.51 (1.19–10.68)	114.81 (45.32–308.47)
Malaysia	9.05 (8.05–10.12)	111.26 (104.02–119.00)	3.50 (2.67–4.28)	107.43 (84.91–127.31)
Maldives	8.55 (7.54–9.73)	99.37 (92.52–106.47)	2.64 (2.05–3.31)	79.13 (63.73–97.86)
Mali	5.19 (4.46–6.02)	82.80 (76.91–89.09)	2.48 (1.11–5.53)	87.74 (47.00–178.27)
Malta	6.14 (5.27–7.10)	89.92 (83.52–95.57)	1.18 (1.05–1.32)	45.19 (40.40–50.63)
Marshall Islands	20.18 (18.03–22.51)	211.06 (199.03–223.26)	12.31 (7.83–17.85)	431.34 (286.73–619.46)
Mauritania	5.18 (4.40–6.09)	86.50 (80.17–93.07)	2.25 (0.98–5.16)	77.82 (39.96–164.89)
Mauritius	10.76 (9.55–12.13)	131.33 (122.54–140.12)	5.72 (5.27–6.09)	187.30 (171.59–199.49)
Mexico	9.82 (8.60–11.24)	133.61 (120.26–148.39)	4.04 (3.59–4.49)	130.47 (117.11–144.99)
Micronesia (Federated States of)	19.75 (17.67–22.13)	206.50 (194.12–218.19)	11.87 (7.68–16.99)	406.22 (273.77–555.66)
Monaco	5.60 (4.82–6.42)	79.53 (73.88–85.24)	1.87 (1.34–2.55)	65.82 (49.14–88.01)
Mongolia	13.01 (11.61–14.64)	93.97 (87.17–101.53)	12.41 (9.07–16.05)	312.23 (232.02–397.20)
Montenegro	5.23 (4.58–5.88)	59.11 (54.08–64.00)	2.15 (1.54–2.89)	61.62 (47.52–78.33)
Morocco	6.87 (6.05–7.77)	76.98 (70.71–83.33)	3.43 (2.02–5.41)	97.77 (59.95–148.98)
Mozambique	8.46 (7.27–10.10)	102.14 (94.42–109.95)	5.38 (1.75–15.43)	174.18 (64.61–475.93)
Myanmar	9.48 (8.49–10.69)	97.64 (90.82–104.40)	5.89 (4.15–9.64)	191.83 (142.91–273.19)
Namibia	5.78 (5.05–6.68)	78.07 (72.17–84.07)	1.96 (1.33–3.12)	62.49 (43.55–94.93)
Nauru	16.13 (14.37–17.95)	204.70 (192.62–216.23)	12.87 (9.14–17.22)	476.38 (352.58–635.27)
Nepal	8.88 (7.71–10.34)	83.28 (76.73–90.27)	4.57 (2.67–7.23)	139.47 (88.10–211.95)
Netherlands	6.53 (5.68–7.43)	90.37 (84.14–96.32)	2.27 (2.03–2.45)	65.47 (60.53–70.34)
New Zealand	5.81 (5.07–6.60)	71.98 (64.07–80.26)	2.97 (2.68–3.21)	89.48 (82.69–95.65)
Nicaragua	10.04 (8.78–11.51)	139.38 (130.39–146.85)	2.75 (2.15–3.37)	96.00 (78.21–115.95)
Niger	5.83 (5.06–6.89)	91.00 (84.43–97.84)	2.95 (1.04–7.65)	94.89 (42.70–216.47)
Nigeria	4.46 (3.84–5.20)	74.32 (66.32–82.16)	1.55 (0.75–3.55)	55.39 (31.94–111.94)
Niue	13.10 (11.65–14.70)	173.13 (163.58–182.31)	7.37 (5.75–9.48)	298.12 (246.16–369.82)
North Macedonia	12.93 (11.83–14.06)	102.43 (95.64–109.51)	7.79 (5.94–9.92)	211.45 (166.54–264.51)
Northern Mariana Islands	12.95 (11.65–14.31)	168.91 (160.13–178.12)	7.11 (5.75–8.91)	216.18 (175.42–265.97)
Norway	6.91 (6.02–7.96)	102.78 (92.05–113.34)	2.20 (1.97–2.35)	63.46 (58.36–68.61)
Oman	11.42 (10.19–12.71)	103.73 (96.96–110.22)	1.57 (1.06–2.09)	52.11 (39.30–65.13)
Pakistan	10.37 (9.02–11.96)	98.96 (86.98–112.63)	5.34 (3.48–8.29)	176.88 (123.25–257.80)

© 2025 GBD 2021 Global Subarachnoid Hemorrhage Risk Factors Collaborators. *JAMA Neurology*.

Palau	14.52 (12.97–16.19)	184.28 (174.34–194.05)	6.59 (5.04–8.28)	245.55 (193.08–301.42)
Palestine	5.33 (4.62–6.03)	74.08 (68.33–80.20)	0.97 (0.74–1.17)	34.44 (28.83–40.46)
Panama	11.81 (10.47–13.30)	160.11 (151.58–168.51)	4.97 (3.90–5.97)	153.87 (123.67–182.57)
Papua New Guinea	10.99 (9.77–12.28)	131.22 (123.00–139.38)	9.06 (5.89–13.58)	290.54 (204.77–407.57)
Paraguay	10.88 (9.68–12.15)	123.57 (115.68–131.28)	5.47 (4.10–7.14)	176.82 (136.33–228.17)
Peru	10.45 (9.31–11.74)	158.49 (150.20–167.11)	4.25 (3.04–5.70)	153.50 (113.79–197.66)
Philippines	9.60 (8.37–11.03)	106.64 (94.67–119.65)	4.29 (3.54–5.18)	151.94 (125.29–178.28)
Poland	6.21 (5.40–7.09)	70.00 (61.85–78.81)	2.60 (2.37–2.84)	91.34 (83.49–99.86)
Portugal	5.65 (4.84–6.43)	69.29 (63.56–75.18)	3.10 (2.80–3.35)	85.82 (79.96–92.53)
Puerto Rico	7.83 (6.81–8.85)	126.86 (119.98–133.74)	1.97 (1.62–2.31)	66.93 (56.65–77.65)
Qatar	7.80 (6.85–8.70)	100.79 (94.44–107.20)	3.22 (2.22–4.42)	82.29 (61.54–108.28)
Republic of Korea	10.63 (9.32–12.29)	127.76 (120.90–135.37)	3.42 (2.79–4.02)	107.27 (93.63–123.56)
Republic of Moldova	5.67 (4.91–6.39)	57.23 (52.68–61.98)	2.08 (1.86–2.34)	66.94 (59.78–75.43)
Romania	9.66 (8.82–10.44)	70.97 (64.51–77.26)	5.25 (4.54–5.93)	150.40 (128.51–169.85)
Russian Federation	10.12 (8.88–11.54)	88.47 (78.13–99.73)	5.99 (5.51–6.44)	177.74 (164.71–191.72)
Rwanda	7.26 (6.28–8.56)	94.60 (88.20–101.51)	2.77 (1.01–7.37)	88.92 (39.00–209.75)
Saint Kitts and Nevis	9.97 (8.90–11.15)	129.03 (121.45–137.11)	4.36 (3.66–5.09)	132.11 (111.94–156.24)
Saint Lucia	9.61 (8.56–10.75)	132.71 (125.14–139.95)	3.69 (3.01–4.42)	118.66 (98.19–142.38)
Saint Vincent and the Grenadines	8.31 (7.43–9.22)	115.39 (108.34–122.46)	3.22 (2.83–3.65)	109.86 (96.29–124.86)
Samoa	15.40 (13.78–17.40)	189.56 (179.08–200.11)	8.10 (5.75–11.36)	272.27 (197.64–370.52)
San Marino	5.62 (4.80–6.60)	82.51 (76.64–88.87)	0.69 (0.42–1.02)	29.91 (21.78–40.14)
Sao Tome and Principe	5.96 (5.10–7.02)	100.78 (93.68–107.64)	2.45 (1.12–6.00)	90.59 (45.45–211.51)
Saudi Arabia	4.57 (3.96–5.17)	58.70 (53.59–63.68)	1.32 (0.90–1.75)	46.41 (33.48–59.46)
Senegal	5.13 (4.38–6.04)	84.94 (79.12–91.18)	2.75 (1.27–6.29)	94.31 (48.54–200.08)
Serbia	9.88 (9.04–10.69)	75.65 (69.85–81.41)	4.88 (3.87–6.08)	142.86 (115.43–173.60)
Seychelles	7.19 (6.29–8.12)	93.05 (86.22–100.16)	1.98 (1.31–2.92)	73.17 (54.77–94.85)
Sierra Leone	5.61 (4.80–6.56)	90.10 (83.92–96.52)	2.93 (1.21–8.14)	106.18 (52.39–262.54)
Singapore	8.72 (7.40–10.29)	130.49 (122.69–138.24)	1.32 (1.19–1.44)	57.01 (49.88–63.87)
Slovakia	5.59 (4.86–6.29)	67.19 (61.98–72.72)	2.15 (1.68–2.64)	73.18 (60.56–87.70)
Slovenia	5.27 (4.53–5.98)	65.56 (60.79–70.78)	1.37 (1.15–1.57)	43.32 (37.01–49.25)
Solomon Islands	24.22 (21.64–27.07)	227.03 (213.74–240.19)	11.68 (7.44–16.89)	359.24 (244.30–501.87)
Somalia	9.04 (7.74–10.81)	112.55 (103.93–120.66)	4.62 (0.79–16.89)	146.23 (36.19–474.18)
South Africa	4.63 (4.02–5.39)	66.13 (59.08–73.63)	1.46 (1.20–1.68)	49.67 (42.12–56.90)
South Sudan	6.82 (5.88–8.02)	89.42 (83.17–96.07)	3.14 (1.20–7.70)	101.47 (47.05–219.37)
Spain	6.05 (5.27–6.89)	81.07 (75.69–86.98)	2.27 (2.02–2.47)	63.46 (58.71–68.10)
Sri Lanka	10.69 (9.44–12.00)	112.28 (105.24–119.06)	2.01 (1.34–2.77)	69.07 (51.67–88.03)
Sudan	6.36 (5.62–7.19)	68.68 (63.17–74.62)	3.60 (1.84–6.50)	110.18 (59.09–182.69)
Suriname	10.29 (9.18–11.54)	130.80 (122.93–138.60)	4.44 (3.31–5.75)	155.77 (122.99–195.04)
Sweden	6.48 (5.59–7.44)	89.46 (79.95–99.12)	1.81 (1.58–2.04)	52.92 (47.30–58.72)
Switzerland	5.52 (4.73–6.42)	74.67 (69.29–80.39)	1.66 (1.43–1.84)	46.55 (42.15–51.03)
Syrian Arab Republic	6.73 (5.96–7.48)	98.67 (92.80–104.98)	2.35 (1.56–3.31)	75.11 (54.37–101.19)
Taiwan (Province of China)	6.85 (6.05–7.63)	88.19 (82.07–94.84)	3.84 (2.98–4.78)	107.74 (86.97–132.16)
Tajikistan	8.94 (7.91–10.04)	108.90 (101.54–116.17)	2.30 (2.08–2.49)	80.11 (72.28–87.41)
Thailand	11.16 (10.03–12.44)	89.69 (82.68–96.50)	3.87 (2.75–5.14)	112.59 (83.54–147.39)
Timor-Leste	12.63 (11.30–14.29)	126.06 (117.79–133.79)	8.18 (6.29–10.53)	237.39 (186.44–308.38)
Togo	9.39 (8.25–10.71)	96.75 (89.60–103.82)	6.35 (3.51–11.85)	193.12 (121.46–327.81)
Tokelau	5.45 (4.69–6.37)	86.87 (80.77–93.29)	3.15 (1.34–8.02)	107.37 (50.83–256.83)
Tonga	13.55 (12.03–15.22)	175.54 (165.46–185.73)	7.97 (5.84–11.52)	320.05 (251.00–442.39)
Trinidad and Tobago	10.04 (8.90–11.32)	134.67 (127.42–142.19)	3.91 (2.68–5.69)	134.15 (97.09–189.50)
Tunisia	9.79 (8.80–10.98)	125.48 (117.64–132.74)	4.95 (3.80–6.31)	169.90 (133.29–215.74)
Turkey	6.05 (5.29–6.80)	77.30 (71.35–83.49)	2.14 (1.14–3.59)	60.86 (37.89–96.26)
Turkmenistan	12.15 (10.92–13.50)	106.44 (99.20–113.84)	8.38 (6.32–11.99)	264.08 (202.57–363.86)
Tuvalu	16.93 (15.06–19.00)	187.45 (176.97–198.95)	10.45 (6.82–15.16)	358.85 (245.49–500.52)
Uganda	6.79 (5.83–8.06)	87.52 (81.16–94.10)	2.33 (0.88–6.13)	78.61 (35.91–188.61)
Ukraine	9.20 (8.07–10.49)	82.99 (73.31–93.35)	4.97 (3.78–6.28)	142.97 (107.72–179.64)
United Arab Emirates	5.73 (4.99–6.43)	70.94 (65.22–76.57)	3.69 (2.72–4.85)	86.38 (67.94–109.03)
United Kingdom	7.08 (6.24–8.10)	88.36 (79.31–97.77)	3.01 (2.76–3.14)	85.03 (80.48–89.34)
United Republic of Tanzania	6.92 (5.94–8.15)	91.32 (85.09–97.80)	2.24 (0.87–6.18)	74.75 (35.77–182.11)
United States of America	6.30 (5.49–7.24)	97.57 (88.38–108.09)	3.24 (2.96–3.42)	100.36 (94.75–105.76)
United States Virgin Islands	9.84 (8.78–11.05)	135.20 (127.09–142.72)	2.60 (1.96–3.39)	92.52 (69.94–120.09)
Uruguay	13.40 (12.00–15.00)	144.97 (135.28–155.80)	6.68 (6.18–7.15)	210.03 (196.11–224.86)
Uzbekistan	6.87 (6.11–7.68)	70.07 (65.05–75.12)	4.51 (3.77–5.42)	121.28 (103.75–143.62)
Vanuatu	17.11 (15.29–19.23)	196.00 (185.37–207.67)	9.71 (5.90–14.23)	346.50 (228.12–497.84)
Venezuela (Bolivarian Republic of)	12.35 (11.02–14.08)	169.99 (161.22–178.79)	5.51 (4.11–7.12)	184.51 (138.38–235.54)
Viet Nam	10.49 (9.29–11.92)	107.41 (100.42–114.73)	5.23 (2.92–9.86)	151.17 (100.92–239.12)
Yemen	7.67 (6.81–8.73)	75.76 (69.58–82.51)	5.53 (2.54–10.61)	150.97 (78.71–267.26)
Zambia	7.70 (6.65–8.97)	95.33 (88.71–102.11)	3.63 (1.65–7.67)	113.69 (56.80–225.18)
Zimbabwe	8.46 (7.44–9.72)	111.64 (104.73–118.38)	6.01 (4.00–9.61)	184.88 (127.85–282.71)

© 2025 GBD 2021 Global Subarachnoid Hemorrhage Risk Factors Collaborators. *JAMA Neurology*.

Supplementary Table 5. Age-standardized incidence, prevalence, mortality, and disability-adjusted life year rates of subarachnoid hemorrhage per 100,000 people in 2021 by five sociodemographic index levels, seven Global Burden of Disease super-regions, and 21 Global Burden of Disease regions.

Region	Incidence (95% UI)	Prevalence (95% UI)	Mortality (95% UI)	DALY (95% UI)
By 5 SDI levels				
Low SDI	7.41 (6.46–8.56)	85.38 (78.61–92.74)	3.79 (2.06–7.35)	120.87 (71.27–221.66)
Low-middle SDI	8.86 (7.79–10.17)	89.40 (81.44–98.17)	4.90 (3.77–6.29)	154.18 (122.57–193.50)
Middle SDI	9.16 (8.08–10.43)	90.81 (82.22–99.93)	5.23 (4.29–6.08)	143.43 (122.51–161.79)
High-middle SDI	7.42 (6.59–8.35)	75.57 (68.69–82.70)	3.52 (3.09–4.15)	99.55 (89.94–115.08)
High SDI	7.85 (6.91–8.97)	112.40 (103.72–121.30)	2.89 (2.64–3.04)	91.84 (86.03–97.38)
By 7 GBD super-regions				
Central Europe, Eastern Europe, and Central Asia	8.73 (7.78–9.77)	80.08 (72.61–88.23)	4.69 (4.40–5.00)	140.21 (130.79–149.09)
High-income	8.02 (7.07–9.14)	113.70 (104.90–122.41)	2.97 (2.70–3.12)	94.52 (88.72–100.10)
Latin America and Caribbean	10.80 (9.56–12.23)	133.53 (123.75–144.16)	5.18 (4.75–5.60)	170.29 (157.91–183.84)
North Africa and the Middle East	5.96 (5.27–6.66)	73.37 (67.78–79.19)	2.85 (2.28–3.69)	82.79 (67.60–104.61)
South Asia	8.44 (7.33–9.75)	79.17 (70.58–88.77)	4.47 (3.14–6.23)	142.60 (106.78–189.60)
Southeast Asia, East Asia, and Oceania	8.79 (7.75–10.03)	80.79 (72.86–89.24)	5.10 (4.10–6.23)	136.70 (112.24–162.70)
Sub-Saharan Africa	6.11 (5.30–7.16)	86.31 (79.85–92.85)	2.55 (1.25–6.24)	86.08 (46.99–189.72)
By 21 GBD regions				
Andean Latin America	12.29 (11.04–13.73)	172.03 (164.02–180.40)	6.01 (4.87–7.29)	199.49 (166.06–238.99)
Australasia	6.06 (5.34–6.86)	79.75 (74.31–85.82)	2.48 (2.22–2.68)	73.27 (68.07–78.65)
Caribbean	10.40 (9.34–11.71)	136.89 (129.73–143.78)	5.02 (3.88–6.25)	191.37 (146.14–234.04)
Central Asia	8.97 (8.10–9.98)	82.54 (77.09–88.06)	5.20 (4.73–5.76)	143.22 (129.04–158.53)
Central Europe	7.27 (6.55–8.02)	71.25 (65.34–77.44)	3.39 (3.11–3.65)	106.16 (97.77–114.30)
Central Latin America	11.07 (9.92–12.51)	148.55 (138.64–159.21)	4.76 (4.22–5.31)	151.45 (135.49–170.06)
Central Sub-Saharan Africa	7.29 (6.26–8.51)	93.43 (86.78–100.05)	3.15 (1.24–8.95)	99.08 (47.49–248.36)
East Asia	7.89 (6.94–9.03)	70.06 (62.78–77.98)	4.71 (3.49–5.87)	116.37 (89.68–141.75)
Eastern Europe	9.59 (8.42–10.87)	85.36 (75.88–95.62)	5.49 (5.03–5.93)	162.91 (150.36–175.08)
Eastern Sub-Saharan Africa	7.25 (6.25–8.62)	93.53 (86.32–100.98)	2.92 (1.10–8.24)	96.66 (42.93–245.35)
High-income Asia Pacific	14.09 (12.30–16.39)	193.60 (175.43–212.12)	3.90 (3.48–4.20)	135.70 (124.59–146.65)
High-income North America	6.37 (5.56–7.31)	98.19 (89.57–107.94)	3.15 (2.87–3.31)	97.81 (92.56–103.14)
North Africa and Middle East	5.96 (5.27–6.66)	73.37 (67.78–79.19)	2.85 (2.28–3.69)	82.79 (67.60–104.61)
Oceania	12.52 (11.25–13.97)	146.97 (138.61–155.53)	8.61 (6.03–11.95)	285.62 (209.42–379.65)
South Asia	8.44 (7.33–9.75)	79.17 (70.58–88.77)	4.47 (3.14–6.23)	142.60 (106.78–189.60)
Southeast Asia	10.89 (9.66–12.36)	110.62 (101.73–120.43)	6.02 (5.02–8.42)	182.03 (155.77–235.86)
Southern Latin America	10.65 (9.51–11.93)	122.33 (113.61–133.73)	4.13 (3.82–4.39)	134.19 (125.36–142.61)
Southern Sub-Saharan Africa	5.26 (4.59–6.10)	73.10 (66.70–79.97)	2.05 (1.70–2.62)	69.35 (58.45–86.11)
Tropical Latin America	10.21 (8.88–11.79)	108.52 (96.83–121.84)	5.45 (5.12–5.68)	177.53 (169.49–184.75)
Western Europe	6.31 (5.57–7.13)	86.40 (80.64–92.25)	2.28 (2.07–2.41)	67.75 (63.56–71.94)
Western Sub-Saharan Africa	5.00 (4.34–5.84)	81.64 (75.45–88.12)	2.22 (1.12–5.09)	77.81 (44.39–161.92)

DALY= disability-adjusted life years; GBD = Global Burden of Disease; SDI = sociodemographic index; UI = uncertainty intervals

© 2025 GBD 2021 Global Subarachnoid Hemorrhage Risk Factors Collaborators. *JAMA Neurology*.

Supplementary Table 6. Age-standardized incidence, prevalence, mortality and DALY rates (with 95% uncertainty intervals) of SAH per 100,000 people in 2021 for men by five country-specific sociodemographic index (SDI) levels, seven GBD super-regions and 21 GBD regions.

Region	Incidence	Prevalence	Mortality	DALY rate
By 5 SDI levels				
Low SDI	8.24 (7.16–9.56)	92.54 (85.14–100.70)	4.47 (1.78–11.70)	140.62 (63.49–348.93)
Low-middle SDI	9.30 (8.13–10.62)	90.81 (82.86–99.97)	5.28 (3.21–8.11)	167.14 (109.96–247.01)
Middle SDI	9.57 (8.42–10.93)	84.34 (76.20–93.12)	5.69 (4.12–6.92)	154.34 (120.79–183.71)
High-middle SDI	7.81 (6.90–8.83)	69.45 (62.83–76.60)	3.95 (3.31–5.33)	110.12 (95.56–138.57)
High SDI	6.94 (6.07–7.88)	93.23 (85.54–101.02)	2.76 (2.59–2.89)	87.61 (83.22–92.29)
By 7 GBD super-regions				
Central Europe, Eastern Europe, and Central Asia	9.80 (8.66–11.01)	78.18 (70.43–86.80)	5.38 (5.00–5.78)	165.31 (153.36–177.39)
High-income	7.02 (6.16–7.93)	94.95 (87.13–102.75)	2.86 (2.69–2.96)	90.41 (86.25–94.58)
Latin America and Caribbean	9.32 (8.23–10.52)	112.58 (104.31–121.69)	4.56 (4.16–4.99)	150.29 (138.16–164.37)
North Africa and the Middle East	5.75 (5.10–6.44)	63.69 (58.52–69.27)	2.84 (1.99–4.13)	81.08 (60.92–113.57)
South Asia	9.12 (7.90–10.50)	86.09 (76.95–96.53)	5.09 (2.47–8.35)	161.16 (87.73–248.39)
Southeast Asia, East Asia, and Oceania	9.60 (8.46–10.99)	74.54 (67.13–82.49)	5.82 (4.00–7.84)	152.35 (112.56–195.69)
Sub-Saharan Africa	7.10 (6.14–8.31)	94.93 (87.73–102.51)	3.13 (0.98–11.15)	106.16 (41.75–329.79)
By 21 GBD regions				
Andean Latin America	11.02 (9.82–12.43)	157.27 (148.63–165.20)	5.16 (4.05–6.38)	179.70 (144.13–220.19)
Australasia	5.15 (4.51–5.83)	63.29 (57.92–69.10)	2.37 (2.16–2.58)	67.75 (63.05–72.85)
Caribbean	8.82 (7.86–9.97)	111.62 (104.96–118.25)	4.37 (3.17–5.88)	160.79 (117.34–206.86)
Central Asia	10.20 (9.17–11.31)	86.30 (80.07–92.12)	6.30 (5.68–7.06)	176.40 (158.86–197.43)
Central Europe	7.46 (6.67–8.26)	64.12 (58.57–69.91)	3.54 (3.25–3.85)	110.43 (101.11–120.26)
Central Latin America	9.59 (8.50–10.84)	123.79 (115.25–133.16)	4.40 (3.85–4.98)	143.00 (126.16–162.59)
Central Sub-Saharan Africa	8.58 (7.36–10.06)	102.04 (94.99–110.23)	3.97 (0.87–16.73)	120.36 (37.58–445.13)
East Asia	8.84 (7.73–10.20)	65.24 (58.24–72.82)	5.62 (3.37–7.77)	133.76 (86.14–178.88)
Eastern Europe	11.19 (9.80–12.78)	85.20 (75.03–96.84)	6.54 (5.90–7.16)	200.83 (181.05–220.18)
Eastern Sub-Saharan Africa	8.77 (7.54–10.31)	106.59 (98.17–115.40)	3.95 (0.98–15.05)	127.42 (41.61–429.22)
High-income Asia Pacific	11.93 (10.22–13.76)	167.95 (151.32–185.47)	3.73 (3.49–3.96)	136.84 (128.08–146.82)
High-income North America	5.62 (4.94–6.39)	71.83 (65.36–79.47)	2.97 (2.77–3.13)	89.40 (84.76–94.00)
North Africa and Middle East	5.75 (5.10–6.44)	63.69 (58.52–69.27)	2.84 (1.99–4.13)	81.08 (60.92–113.57)
Oceania	11.55 (10.35–13.11)	149.54 (141.34–159.06)	7.23 (4.18–12.80)	269.77 (173.00–436.66)
South Asia	9.12 (7.90–10.50)	86.09 (76.95–96.53)	5.09 (2.47–8.35)	161.16 (87.73–248.39)
Southeast Asia	11.12 (9.82–12.61)	100.31 (92.00–109.56)	6.00 (4.56–9.23)	190.48 (151.77–268.36)
Southern Latin America	9.01 (8.10–10.17)	106.47 (99.57–113.54)	4.12 (3.80–4.43)	129.99 (120.33–139.57)
Southern Sub-Saharan Africa	5.53 (4.83–6.36)	73.61 (67.00–80.45)	2.24 (1.72–3.16)	76.17 (60.43–104.79)
Tropical Latin America	8.66 (7.50–10.00)	90.05 (80.27–101.04)	4.61 (4.35–4.87)	147.03 (140.10–154.33)
Western Europe	5.81 (5.15–6.54)	77.82 (72.26–83.64)	2.28 (2.14–2.39)	65.66 (62.01–69.54)
Western Sub-Saharan Africa	5.64 (4.87–6.54)	88.14 (81.41–95.50)	2.51 (0.76–8.51)	91.73 (37.43–266.26)

DALY = disability-adjusted life years; GBD = Global Burden of Disease; SDI = sociodemographic index

© 2025 GBD 2021 Global Subarachnoid Hemorrhage Risk Factors Collaborators. *JAMA Neurology*.

Supplementary Table 7. Age-standardized incidence, prevalence, mortality and DALY rates (with 95% uncertainty intervals) of SAH per 100,000 people in 2021 for women by five country-specific sociodemographic index (SDI) levels, seven GBD super-regions and 21 GBD regions.

Region	Incidence	Prevalence	Mortality	DALY rate
By 5 SDI levels				
Low SDI	6.63 (5.76–7.71)	78.39 (71.81–85.12)	3.16 (2.15–6.42)	101.83 (72.93–180.72)
Low-middle SDI	8.47 (7.45–9.72)	87.94 (80.09–96.35)	4.55 (3.59–6.07)	141.55 (112.27–183.05)
Middle SDI	8.81 (7.79–10.04)	96.81 (87.97–106.22)	4.85 (3.94–5.91)	133.13 (113.81–154.51)
High-middle SDI	7.11 (6.27–7.99)	81.07 (73.87–88.47)	3.16 (2.74–3.70)	89.59 (79.90–101.36)
High SDI	8.72 (7.65–10.01)	129.75 (119.95–139.68)	2.97 (2.63–3.18)	95.08 (87.74–102.07)
By 7 GBD super-regions				
Central Europe, Eastern Europe, and Central Asia	7.79 (6.91–8.70)	80.94 (73.73–89.01)	4.07 (3.73–4.40)	117.44 (107.65–126.61)
High-income	8.95 (7.88–10.26)	130.42 (120.69–140.02)	3.05 (2.70–3.24)	97.76 (90.86–104.51)
Latin America and Caribbean	12.15 (10.80–13.80)	152.10 (140.93–163.88)	5.71 (5.23–6.21)	187.89 (172.76–203.62)
North Africa and the Middle East	6.22 (5.50–7.01)	83.58 (77.78–89.57)	2.86 (2.15–3.77)	84.64 (65.68–109.65)
South Asia	7.80 (6.74–9.05)	72.38 (64.41–81.24)	3.90 (2.94–5.49)	124.34 (96.59–166.87)
Southeast Asia, East Asia, and Oceania	8.11 (7.14–9.22)	86.59 (78.26–95.35)	4.54 (3.53–5.70)	122.22 (100.15–146.27)
Sub-Saharan Africa	5.24 (4.52–6.12)	78.37 (72.23–84.27)	2.04 (1.33–4.65)	67.74 (47.97–137.39)
By 21 GBD regions				
Andean Latin America	13.48 (12.02–15.11)	185.62 (176.47–195.18)	6.77 (5.46–8.40)	217.34 (179.04–264.63)
Australasia	6.94 (6.04–7.94)	95.00 (88.97–101.66)	2.59 (2.27–2.83)	78.37 (71.78–85.49)
Caribbean	11.93 (10.65–13.47)	160.15 (151.80–168.07)	5.64 (3.99–7.19)	220.85 (146.49–283.13)
Central Asia	7.86 (7.04–8.77)	79.01 (73.54–84.49)	4.32 (3.86–4.81)	114.41 (102.27–127.13)
Central Europe	7.15 (6.39–7.92)	77.75 (71.65–84.01)	3.24 (2.93–3.55)	101.77 (93.08–110.76)
Central Latin America	12.40 (11.13–14.08)	170.23 (158.67–181.73)	5.05 (4.41–5.74)	158.25 (139.19–180.17)
Central Sub-Saharan Africa	6.22 (5.32–7.35)	84.92 (78.66–91.22)	2.55 (1.38–6.28)	80.51 (49.44–175.09)
East Asia	7.05 (6.16–8.03)	74.62 (67.03–82.85)	4.03 (2.84–5.41)	100.87 (76.37–130.67)
Eastern Europe	8.22 (7.22–9.36)	84.22 (75.17–94.16)	4.62 (4.12–5.14)	130.59 (116.00–144.77)
Eastern Sub-Saharan Africa	5.89 (5.05–6.94)	81.17 (74.82–87.48)	2.03 (1.07–5.46)	68.25 (41.18–161.60)
High-income Asia Pacific	15.95 (13.81–18.66)	214.37 (194.44–235.95)	3.88 (3.28–4.29)	131.39 (118.46–144.12)
High-income North America	7.15 (6.21–8.28)	122.54 (111.50–134.36)	3.30 (2.98–3.50)	105.46 (99.00–112.12)
North Africa and Middle East	6.22 (5.50–7.01)	83.58 (77.78–89.57)	2.86 (2.15–3.77)	84.64 (65.68–109.65)
Oceania	13.50 (12.10–15.08)	144.54 (135.78–153.05)	9.96 (6.78–14.27)	302.38 (215.65–404.09)
South Asia	7.80 (6.74–9.05)	72.38 (64.41–81.24)	3.90 (2.94–5.49)	124.34 (96.59–166.87)
Southeast Asia	10.70 (9.47–12.16)	119.37 (109.99–129.74)	5.91 (4.61–8.95)	171.75 (138.38–232.82)
Southern Latin America	12.17 (10.73–13.85)	137.27 (125.92–153.41)	4.17 (3.81–4.48)	138.22 (128.30–148.36)
Southern Sub-Saharan Africa	5.07 (4.37–5.91)	72.00 (65.35–78.89)	1.89 (1.53–2.34)	63.05 (52.41–75.64)
Tropical Latin America	11.66 (10.10–13.57)	124.99 (111.68–140.14)	6.20 (5.71–6.53)	204.92 (194.01–214.57)
Western Europe	6.81 (5.94–7.75)	94.27 (87.82–100.51)	2.29 (2.05–2.45)	69.78 (64.68–74.36)
Western Sub-Saharan Africa	4.42 (3.79–5.16)	75.78 (69.67–82.07)	1.95 (1.26–4.38)	65.08 (45.87–124.61)

DALY = disability-adjusted life years; GBD = Global Burden of Disease; SDI = sociodemographic index

© 2025 GBD 2021 Global Subarachnoid Hemorrhage Risk Factors Collaborators. *JAMA Neurology*.

Supplementary Table 8. Incidence, prevalence, mortality and DALY rates (with 95% uncertainty intervals) of SAH per 100,000 people in 2021 for children (0–14 years of age) by five country-specific sociodemographic index (SDI) levels, seven GBD super-regions and 21 GBD regions.

Region	Incidence	Prevalence	Mortality	DALY rate
By 5 SDI levels				
Low SDI	1.68 (1.22–2.25)	9.52 (7.87–11.57)	0.21 (0.14–0.30)	19.34 (13.02–27.06)
Low-middle SDI	1.57 (1.09–2.15)	9.33 (7.64–11.50)	0.22 (0.17–0.27)	20.13 (15.69–24.64)
Middle SDI	1.82 (1.24–2.50)	11.29 (9.38–13.64)	0.13 (0.11–0.15)	12.67 (11.02–14.51)
High-middle SDI	1.73 (1.15–2.41)	11.11 (9.22–13.33)	0.07 (0.06–0.08)	7.51 (6.46–9.02)
High SDI	1.43 (0.96–2.03)	10.97 (8.97–13.59)	0.06 (0.06–0.07)	6.96 (6.24–7.69)
By 7 GBD super-regions				
Central Europe, Eastern Europe, and Central Asia	1.46 (0.97–2.06)	8.78 (7.10–10.91)	0.05 (0.04–0.05)	5.42 (4.76–6.13)
High-income	1.28 (0.85–1.80)	9.94 (7.96–12.46)	0.07 (0.06–0.07)	7.02 (6.33–7.72)
Latin America and Caribbean	2.28 (1.61–3.08)	13.99 (11.89–16.59)	0.28 (0.23–0.35)	25.18 (20.29–30.38)
North Africa and the Middle East	2.22 (1.60–2.98)	14.61 (12.51–17.07)	0.24 (0.17–0.36)	22.66 (16.64–32.81)
South Asia	1.29 (0.86–1.81)	7.39 (5.80–9.30)	0.20 (0.14–0.26)	17.86 (13.16–23.05)
Southeast Asia, East Asia, and Oceania	1.83 (1.24–2.52)	11.51 (9.53–13.94)	0.14 (0.12–0.17)	14.25 (12.05–16.69)
Sub-Saharan Africa	1.71 (1.24–2.32)	9.78 (8.09–11.85)	0.13 (0.07–0.22)	12.46 (7.09–20.03)
By 21 GBD regions				
Andean Latin America	2.59 (1.87–3.44)	14.71 (12.62–17.05)	0.40 (0.33–0.50)	35.21 (29.11–43.36)
Australasia	1.23 (0.82–1.73)	8.66 (7.00–10.70)	0.03 (0.02–0.03)	3.32 (2.85–3.87)
Caribbean	3.08 (2.31–3.97)	21.12 (18.84–23.74)	1.05 (0.66–1.48)	92.81 (58.79–130.11)
Central Asia	1.38 (0.94–1.94)	7.40 (5.93–9.19)	0.04 (0.03–0.05)	4.99 (4.13–6.01)
Central Europe	1.46 (0.97–2.06)	10.01 (8.13–12.32)	0.05 (0.05–0.06)	6.34 (5.44–7.26)
Central Latin America	2.31 (1.60–3.13)	14.82 (12.47–17.69)	0.21 (0.17–0.25)	18.57 (15.25–22.59)
Central Sub-Saharan Africa	1.20 (0.82–1.66)	7.01 (5.50–8.73)	0.11 (0.06–0.18)	10.19 (6.25–16.15)
East Asia	1.75 (1.15–2.45)	11.23 (9.16–13.73)	0.06 (0.05–0.08)	7.46 (6.10–9.30)
Eastern Europe	1.53 (0.99–2.15)	9.24 (7.45–11.47)	0.04 (0.04–0.05)	5.30 (4.60–6.02)
Eastern Sub-Saharan Africa	1.25 (0.85–1.72)	7.35 (5.82–9.21)	0.10 (0.05–0.21)	9.23 (4.80–19.14)
High-income Asia Pacific	1.54 (1.02–2.20)	11.37 (9.24–13.90)	0.05 (0.04–0.05)	5.72 (4.93–6.49)
High-income North America	1.28 (0.85–1.81)	11.72 (9.32–14.95)	0.11 (0.10–0.12)	10.98 (9.98–12.03)
North Africa and Middle East	2.22 (1.60–2.98)	14.61 (12.51–17.07)	0.24 (0.17–0.36)	22.66 (16.64–32.81)
Oceania	4.58 (3.67–5.64)	29.93 (27.41–32.76)	1.40 (0.91–2.03)	128.21 (83.82–183.84)
South Asia	1.29 (0.86–1.81)	7.39 (5.80–9.30)	0.20 (0.14–0.26)	17.86 (13.16–23.05)
Southeast Asia	1.87 (1.30–2.56)	11.40 (9.53–13.75)	0.23 (0.18–0.28)	21.41 (17.48–25.61)
Southern Latin America	1.61 (1.09–2.26)	10.38 (8.49–12.80)	0.08 (0.07–0.09)	7.97 (6.96–9.17)
Southern Sub-Saharan Africa	1.39 (0.96–1.92)	8.67 (7.10–10.67)	0.08 (0.06–0.10)	7.52 (5.98–9.35)
Tropical Latin America	1.94 (1.34–2.66)	11.04 (9.03–13.51)	0.16 (0.13–0.19)	14.43 (11.95–17.00)
Western Europe	1.13 (0.75–1.60)	7.77 (6.14–9.74)	0.03 (0.03–0.04)	3.73 (3.27–4.18)
Western Sub-Saharan Africa	2.27 (1.66–3.01)	12.68 (10.74–14.96)	0.17 (0.09–0.27)	16.32 (9.03–24.35)

DALY = disability-adjusted life years; GBD = Global Burden of Disease; SDI = sociodemographic index

© 2025 GBD 2021 Global Subarachnoid Hemorrhage Risk Factors Collaborators. *JAMA Neurology*.

Supplementary Table 9. Incidence, prevalence, mortality and DALY rates (with 95% uncertainty intervals) of SAH per 100,000 people in 2021 for young adults (15–49 years of age) by five country-specific sociodemographic index (SDI) levels, seven GBD super-regions and 21 GBD regions.

Region	Incidence	Prevalence	Mortality	DALY rate
By 5 SDI levels				
Low SDI	4.66 (3.67–5.70)	52.97 (47.31–59.22)	1.06 (0.54–2.13)	62.32 (35.18–117.78)
Low-middle SDI	6.55 (5.22–7.94)	65.67 (58.92–73.55)	1.64 (1.24–2.18)	93.22 (72.93–119.98)
Middle SDI	6.47 (5.10–7.98)	72.82 (64.86–82.03)	1.53 (1.31–1.73)	87.99 (76.92–98.66)
High-middle SDI	5.88 (4.58–7.25)	70.88 (63.46–79.34)	1.22 (1.10–1.36)	72.48 (65.75–81.08)
High SDI	6.30 (4.96–7.85)	78.16 (70.57–86.84)	1.14 (1.10–1.18)	67.46 (63.93–71.21)
By 7 GBD super-regions				
Central Europe, Eastern Europe, and Central Asia	7.89 (6.31–9.61)	78.22 (70.80–86.75)	2.10 (1.95–2.24)	117.91 (109.77–125.72)
High-income	6.56 (5.20–8.16)	82.91 (74.46–92.59)	1.21 (1.18–1.25)	70.58 (67.38–74.20)
Latin America and Caribbean	8.68 (7.09–10.46)	71.40 (63.47–80.34)	2.38 (2.19–2.57)	130.52 (120.34–141.29)
North Africa and the Middle East	4.38 (3.36–5.40)	64.57 (58.63–71.37)	0.76 (0.59–1.00)	48.02 (38.33–61.26)
South Asia	6.62 (5.24–8.15)	95.78 (86.69–105.69)	1.51 (1.09–2.05)	85.54 (63.70–113.99)
Southeast Asia, East Asia, and Oceania	6.10 (4.74–7.59)	61.39 (54.27–70.23)	1.46 (1.17–1.75)	86.71 (71.27–103.24)
Sub-Saharan Africa	3.86 (2.99–4.80)	49.93 (44.46–56.26)	0.78 (0.34–1.88)	47.51 (24.35–105.80)
By 21 GBD regions				
Andean Latin America	10.24 (8.51–11.94)	122.45 (114.49–131.02)	2.58 (2.09–3.15)	148.42 (121.82–178.44)
Australasia	4.77 (3.69–5.94)	57.83 (52.00–64.65)	0.95 (0.86–1.04)	54.40 (49.80–59.71)
Caribbean	8.82 (7.26–10.49)	102.86 (95.56–110.75)	2.78 (1.99–3.69)	153.13 (112.31–200.78)
Central Asia	6.77 (5.36–8.12)	71.84 (66.40–78.15)	1.35 (1.18–1.55)	80.08 (70.00–90.21)
Central Europe	6.32 (5.06–7.57)	79.79 (72.54–87.82)	1.58 (1.44–1.71)	91.62 (83.86–99.15)
Central Latin America	8.23 (6.70–9.94)	100.05 (90.58–110.19)	1.92 (1.69–2.17)	108.22 (95.76–121.62)
Central Sub-Saharan Africa	4.45 (3.41–5.51)	47.99 (43.12–53.71)	0.77 (0.31–2.06)	45.96 (21.84–112.37)
East Asia	5.01 (3.78–6.37)	63.78 (55.60–72.69)	1.06 (0.77–1.34)	64.83 (48.74–78.77)
Eastern Europe	9.31 (7.40–11.45)	90.22 (78.98–103.51)	2.77 (2.54–3.00)	151.47 (139.57–163.74)
Eastern Sub-Saharan Africa	4.41 (3.45–5.44)	49.72 (44.31–55.94)	0.88 (0.31–2.34)	53.43 (22.81–132.14)
High-income Asia Pacific	12.38 (9.75–15.77)	102.11 (89.99–114.74)	2.03 (1.94–2.14)	112.43 (105.54–119.15)
High-income North America	4.82 (3.72–6.01)	79.25 (70.22–89.89)	1.18 (1.11–1.24)	69.95 (65.44–74.55)
North Africa and Middle East	4.38 (3.36–5.40)	64.57 (58.63–71.37)	0.76 (0.59–1.00)	48.02 (38.33–61.26)
Oceania	7.72 (6.30–9.28)	105.48 (98.71–114.00)	3.01 (1.98–4.36)	175.37 (120.93–243.14)
South Asia	6.62 (5.24–8.15)	61.39 (54.27–70.23)	1.51 (1.09–2.05)	85.54 (63.70–113.99)
Southeast Asia	8.10 (6.48–9.87)	84.91 (76.72–94.36)	2.16 (1.79–2.68)	125.63 (105.50–151.34)
Southern Latin America	8.98 (7.43–10.66)	92.60 (86.07–100.08)	1.71 (1.59–1.84)	99.99 (93.06–106.82)
Southern Sub-Saharan Africa	3.55 (2.73–4.37)	46.01 (40.44–52.41)	0.77 (0.59–1.06)	45.65 (36.81–60.09)
Tropical Latin America	8.70 (6.91–10.76)	81.83 (71.82–93.42)	2.76 (2.66–2.86)	145.54 (139.98–151.38)
Western Europe	5.40 (4.23–6.63)	66.29 (60.19–73.10)	0.84 (0.81–0.87)	49.62 (46.94–52.62)
Western Sub-Saharan Africa	3.26 (2.48–4.13)	51.41 (45.69–58.15)	0.69 (0.31–1.59)	42.89 (23.17–90.08)

DALY = disability-adjusted life years; GBD = Global Burden of Disease; SDI = sociodemographic index

© 2025 GBD 2021 Global Subarachnoid Hemorrhage Risk Factors Collaborators. *JAMA Neurology*.

Supplementary Table 10. Incidence, prevalence, mortality and DALY rates (with 95% uncertainty intervals) of SAH per 100,000 people in 2021 for old adults (50–74 years of age) by five country-specific sociodemographic index (SDI) levels, seven GBD super-regions and 21 GBD regions.

Region	Incidence	Prevalence	Mortality	DALY rate
By 5 SDI levels				
Low SDI	17.00 (13.63–21.66)	229.63 (207.56–252.92)	10.89 (5.98–21.27)	348.90 (201.03–658.69)
Low-middle SDI	19.58 (16.04–24.48)	240.28 (215.07–269.35)	14.08 (10.98–18.02)	442.86 (353.15–558.68)
Middle SDI	18.87 (15.42–23.67)	235.66 (208.15–265.15)	12.34 (10.18–14.28)	380.91 (322.49–432.82)
High-middle SDI	14.65 (12.07–18.20)	188.52 (168.33–210.94)	8.19 (7.25–9.66)	257.92 (231.69–301.99)
High SDI	18.82 (15.42–23.37)	317.65 (289.09–348.83)	7.17 (6.82–7.41)	244.85 (229.71–259.29)
By 7 GBD super-regions				
Central Europe, Eastern Europe, and Central Asia	16.71 (13.87–20.46)	199.11 (176.14–223.96)	12.05 (11.27–12.87)	372.74 (347.15–398.07)
High-income	19.49 (16.10–24.09)	328.23 (299.47–358.13)	7.35 (7.02–7.56)	250.19 (236.04–264.96)
Latin America and Caribbean	24.15 (19.93–29.81)	352.17 (319.78–386.68)	14.09 (12.96–15.17)	441.86 (410.35–476.57)
North Africa and the Middle East	10.85 (8.86–13.67)	168.27 (155.98–181.40)	6.00 (4.70–7.79)	193.95 (156.49–244.60)
South Asia	18.54 (15.07–23.33)	214.83 (187.09–246.83)	13.66 (9.84–18.87)	425.53 (314.78–573.59)
Southeast Asia, East Asia, and Oceania	17.44 (14.20–21.86)	206.87 (181.54–233.75)	11.17 (9.09–13.60)	345.90 (289.10–417.24)
Sub-Saharan Africa	14.04 (11.02–18.22)	226.33 (206.51–246.70)	7.03 (3.38–17.41)	238.76 (126.19–546.11)
By 21 GBD regions				
Andean Latin America	26.00 (21.55–31.37)	451.86 (427.01–476.53)	16.14 (12.93–19.67)	501.11 (407.50–607.27)
Australasia	13.27 (10.70–16.73)	222.48 (206.93–238.36)	5.57 (5.06–6.02)	186.49 (171.41–202.63)
Caribbean	22.24 (18.29–27.43)	344.68 (325.74–364.26)	12.88 (9.69–16.40)	407.32 (312.85–513.03)
Central Asia	17.57 (14.57–21.09)	215.95 (200.71–232.42)	12.74 (11.42–14.29)	405.78 (363.13–452.76)
Central Europe	13.21 (11.20–15.60)	164.07 (149.86–180.68)	9.29 (8.49–10.13)	286.40 (262.32–310.11)
Central Latin America	24.91 (20.69–30.50)	393.67 (362.29–424.97)	12.50 (11.03–14.20)	393.61 (350.85–444.44)
Central Sub-Saharan Africa	18.39 (14.43–24.07)	261.86 (242.46–281.93)	8.63 (3.40–25.54)	290.22 (132.90–792.46)
East Asia	15.62 (12.54–19.75)	177.07 (153.09–204.90)	10.27 (7.68–13.07)	308.15 (238.20–382.05)
Eastern Europe	18.47 (14.96–22.89)	214.48 (184.36–248.93)	13.44 (12.34–14.63)	412.96 (377.38–448.11)
Eastern Sub-Saharan Africa	17.73 (13.97–22.87)	258.57 (235.15–281.15)	8.06 (3.01–23.10)	270.95 (119.43–707.04)
High-income Asia Pacific	40.67 (33.48–49.49)	640.51 (572.57–717.50)	10.08 (9.31–10.72)	376.17 (343.18–411.08)
High-income North America	13.99 (11.11–17.84)	259.52 (231.95–289.93)	7.98 (7.58–8.29)	257.79 (245.16–271.24)
North Africa and Middle East	10.85 (8.86–13.67)	168.27 (155.98–181.40)	6.00 (4.70–7.79)	193.95 (156.49–244.60)
Oceania	24.58 (20.29–30.02)	361.17 (338.00–383.98)	19.63 (12.81–28.76)	657.96 (443.90–923.25)
South Asia	18.54 (15.07–23.33)	214.83 (187.09–246.83)	13.66 (9.84–18.87)	425.53 (314.78–573.59)
Southeast Asia	23.25 (19.15–29.00)	301.59 (272.78–333.58)	14.02 (11.69–19.26)	464.53 (392.25–610.51)
Southern Latin America	24.87 (20.90–29.49)	350.15 (323.13–377.65)	11.20 (10.45–11.94)	366.60 (341.76–392.17)
Southern Sub-Saharan Africa	12.10 (9.63–15.59)	189.52 (168.61–211.98)	5.52 (4.54–7.25)	186.42 (156.79–237.57)
Tropical Latin America	23.38 (18.83–29.73)	290.33 (250.51–337.61)	15.44 (14.76–16.10)	483.04 (461.23–504.00)
Western Europe	14.13 (11.60–17.53)	246.49 (230.23–262.88)	5.30 (5.01–5.52)	178.41 (167.07–190.05)
Western Sub-Saharan Africa	10.23 (7.91–13.33)	199.29 (181.86–217.33)	6.11 (2.96–14.02)	211.49 (114.57–450.17)

DALY = disability-adjusted life years; GBD = Global Burden of Disease; SDI = sociodemographic index

© 2025 GBD 2021 Global Subarachnoid Hemorrhage Risk Factors Collaborators. *JAMA Neurology*.

Supplementary Table 11. Incidence, prevalence, mortality and DALY rates (with 95% uncertainty intervals) of SAH per 100,000 people in 2021 for very old adults (≥75 years of age) by five country-specific sociodemographic index (SDI) levels, seven GBD super-regions and 21 GBD regions.

	Incidence	Prevalence	Mortality	DALY rate
By 5 SDI levels				
Low SDI	28.38 (21.41–36.67)	274.83 (231.71–320.78)	26.36 (14.22–51.12)	402.42 (235.47–740.72)
Low-middle SDI	36.57 (28.33–45.93)	239.87 (196.01–289.14)	35.27 (26.16–49.07)	503.49 (383.08–682.60)
Middle SDI	56.43 (45.10–69.62)	284.61 (236.07–339.21)	57.10 (44.47–68.36)	767.64 (606.50–905.37)
High-middle SDI	45.69 (37.70–54.73)	196.40 (161.05–234.07)	41.51 (35.07–49.88)	537.12 (462.41–646.30)
High SDI	38.07 (31.69–45.19)	532.16 (466.28–601.14)	33.78 (27.00–37.28)	482.91 (405.56–531.86)
By 7 GBD super-regions				
Central Europe, Eastern Europe, and Central Asia	52.40 (44.45–61.25)	147.44 (117.00–178.46)	47.87 (43.35–51.13)	603.70 (554.02–643.59)
High-income	37.09 (31.28–43.75)	524.09 (461.01–589.97)	34.43 (27.47–38.00)	486.25 (409.42–534.00)
Latin America and Caribbean	42.32 (34.05–51.15)	519.29 (459.01–584.81)	37.50 (32.69–40.86)	532.13 (474.21–579.26)
North Africa and the Middle East	30.61 (25.08–36.52)	187.13 (158.68–215.52)	32.24 (24.46–42.09)	438.29 (340.49–564.01)
South Asia	32.65 (24.80–41.73)	177.77 (136.93–226.91)	28.60 (17.92–43.39)	412.40 (269.25–606.62)
Southeast Asia, East Asia, and Oceania	61.13 (48.70–75.73)	214.23 (167.94–267.36)	60.47 (46.06–74.71)	802.96 (619.94–988.08)
Sub-Saharan Africa	21.37 (15.93–27.74)	344.36 (300.90–389.00)	18.29 (9.50–44.85)	300.78 (177.41–653.04)
By 21 GBD regions				
Andean Latin America	45.96 (38.10–54.07)	650.27 (601.53–698.48)	44.24 (35.88–52.33)	642.18 (534.31–753.40)
Australasia	32.73 (27.85–38.14)	323.08 (294.54–353.88)	31.28 (25.56–34.76)	411.81 (352.04–453.90)
Caribbean	31.97 (26.73–37.70)	487.07 (449.83–524.27)	27.78 (22.48–33.37)	408.30 (338.61–485.42)
Central Asia	55.12 (48.30–62.88)	176.04 (146.31–206.17)	57.57 (51.37–64.15)	760.45 (681.09–845.05)
Central Europe	49.16 (43.10–55.27)	121.44 (100.42–145.70)	31.81 (28.41–34.28)	415.11 (375.82–445.13)
Central Latin America	49.50 (40.81–58.91)	631.83 (569.56–699.09)	39.53 (34.20–43.88)	569.75 (502.69–629.33)
Central Sub-Saharan Africa	25.17 (19.02–33.28)	382.56 (331.89–436.05)	24.69 (9.44–71.78)	397.48 (187.53–1,031.57)
East Asia	62.16 (49.40–76.87)	188.82 (143.47–240.12)	60.74 (44.13–76.37)	800.45 (581.98–998.39)
Eastern Europe	54.34 (44.08–66.61)	161.88 (124.34–202.76)	58.06 (51.81–63.13)	716.53 (648.55–773.79)
Eastern Sub-Saharan Africa	26.99 (20.10–35.46)	377.15 (320.24–433.62)	21.20 (8.07–61.69)	345.80 (162.12–876.07)
High-income Asia Pacific	49.87 (41.02–60.21)	1,175.06 (1,016.69–1,345.32)	43.31 (32.08–49.63)	713.00 (583.12–813.98)
High-income North America	35.81 (28.96–43.57)	331.79 (287.59–373.75)	34.20 (28.13–37.30)	450.55 (382.70–490.44)
North Africa and Middle East	30.61 (25.08–36.52)	187.13 (158.68–215.52)	32.24 (24.46–42.09)	438.29 (340.49–564.01)
Oceania	63.91 (52.23–76.37)	438.37 (376.47–501.77)	68.70 (46.81–99.17)	986.91 (695.89–1,389.22)
South Asia	32.65 (24.80–41.73)	177.77 (136.93–226.91)	28.60 (17.92–43.39)	412.40 (269.25–606.62)
Southeast Asia	56.82 (46.01–68.77)	317.99 (264.05–376.55)	59.26 (45.67–94.24)	811.88 (645.69–1,252.81)
Southern Latin America	38.51 (33.35–45.01)	340.57 (265.91–485.72)	35.90 (31.18–39.35)	497.24 (441.76–541.01)
Southern Sub-Saharan Africa	21.77 (16.57–27.92)	334.50 (284.55–384.84)	15.91 (13.29–19.49)	265.10 (224.66–315.41)
Tropical Latin America	36.98 (28.56–46.84)	388.04 (312.24–470.24)	36.24 (30.86–39.39)	498.95 (434.81–540.22)
Western Europe	31.25 (27.00–35.69)	321.59 (289.99–353.77)	29.96 (24.31–32.85)	391.43 (332.43–426.47)
Western Sub-Saharan Africa	15.76 (11.87–20.46)	312.14 (278.06–347.57)	15.27 (8.26–35.38)	254.14 (157.78–516.81)

DALY = disability-adjusted life years; GBD = Global Burden of Disease; SDI = sociodemographic index

© 2025 GBD 2021 Global Subarachnoid Hemorrhage Risk Factors Collaborators. *JAMA Neurology*.

Supplementary Table 12. Percentage changes (with 95% uncertainty intervals) of the incidence, prevalence, mortality, and DALYs of subarachnoid hemorrhage in the world between 1990 and 2021, separately for men, women, and different age groups.

	Changes in % (95% uncertainty interval) between 1990 and 2021			
	Incidence	Prevalence	Deaths	DALY
Overall				
In absolute numbers	37.09 (32.20–42.30)	60.21 (56.86–63.39)	-5.89 (-22.78–25.51)	-11.55 (-25.22–10.25)
In age-standardized rates per 100,000 people	-28.78 (-31.59–-25.66)	-16.13 (-17.70–-14.76)	-56.12 (-64.27–-40.74)	-54.61 (-61.88–-42.81)
Men				
In absolute numbers	39.86 (34.40–46.09)	61.68 (57.93–65.11)	-3.98 (-25.98–57.27)	-10.16 (-28.09–30.22)
In age-standardized rates per 100,000 people	-27.41 (-30.67–-23.88)	-14.16 (-15.73–-12.76)	-55.15 (-65.76–-23.58)	-53.56 (-63.18–-30.54)
Women				
In absolute numbers	34.54 (29.99–39.13)	59.01 (55.62–62.07)	-7.67 (-25.96–26.54)	-12.98 (-26.89–14.37)
In age-standardized rates per 100,000 people	-30.39 (-32.91–-27.57)	-17.64 (-19.20–-16.20)	-57.16 (-65.47–-41.07)	-55.80 (-62.98–-42.24)
Children (0–14 years old)				
In absolute numbers	-10.15 (-14.15–-6.71)	-2.46 (-5.04–-0.08)	-69.12 (-75.81–-59.14)	-67.49 (-74.20–-57.70)
In rates per 100,000 people	-22.33 (-25.78–-19.35)	-15.68 (-17.91–-13.63)	-73.31 (-79.09–-64.68)	-71.90 (-77.70–-63.43)
Young adults (15–49 years old)				
In absolute numbers	24.02 (18.59–29.33)	31.89 (28.74–35.01)	-16.80 (-26.71–-3.51)	-14.75 (-24.28–-2.71)
In rates per 100,000 people	-14.87 (-18.60–-11.22)	-9.47 (-11.63—7.33)	-42.89 (-49.69–-33.77)	-41.48 (-48.02—33.22)
Old adults (50–74 years old)				
In absolute numbers	41.31 (34.16–48.90)	78.58 (74.15–83.30)	-13.26 (-29.39–16.31)	-7.84 (-23.92–19.60)
In rates per 100,000 people	-34.04 (-37.37–-30.49)	-16.64 (-18.71–-14.44)	-59.51 (-67.04–-45.71)	-56.98 (-64.49–-44.17)
Very old adults (≥75 years old)				
In absolute numbers	87.45 (72.06–107.96)	134.65 (126.86–144.10)	23.30 (-5.74–89.29)	19.36 (-7.55–78.30)
In rates per 100,000 people	-23.76 (-30.02–-15.42)	-4.57 (-7.73–-0.72)	-49.86 (-61.66–-23.01)	-51.46 (-62.40–-27.48)

DALY = disability-adjusted life years

© 2025 GBD 2021 Global Subarachnoid Hemorrhage Risk Factors Collaborators. *JAMA Neurology*.

Supplementary Table 13. Percentage changes (with 95% uncertainty intervals) in the absolute number of SAH incidents, prevalent cases, deaths, and DALYs between 1990 and 2021 by five country-specific sociodemographic index (SDI) levels, seven GBD super-regions and 21 GBD regions.

	Changes in % (95% UI) between 1990 and 2021			
	Incidence	Prevalence	Deaths	DALY
By 5 SDI levels				
Low SDI	92.06 (86.39–98.31)	105.47 (101.57–109.54)	62.08 (30.98–93.63)	58.84 (32.50–91.64)
Low-middle SDI	70.56 (65.17–76.54)	86.44 (82.47–90.40)	50.97 (24.93–88.24)	34.18 (15.97–57.04)
Middle SDI	36.31 (27.74–43.46)	61.74 (56.66–66.53)	-22.05 (-42.00–26.30)	-25.09 (-41.66–9.57)
High-middle SDI	9.53 (5.29–14.22)	27.64 (24.57–30.59)	-23.73 (-41.75–8.07)	-32.39 (-46.10–-11.06)
High SDI	26.77 (22.08–32.01)	57.70 (54.33–61.01)	7.07 (-1.34–13.12)	-10.09 (-14.75–-6.18)
By 7 GBD super-regions				
Central Europe, Eastern Europe, and Central Asia	12.10 (7.80–16.61)	7.21 (5.11–9.25)	20.25 (11.57–28.99)	2.90 (-3.66–9.44)
High-income	27.35 (22.61–32.93)	55.50 (52.14–58.77)	13.76 (5.56–19.82)	-7.59 (-11.59–-4.09)
Latin America and Caribbean	76.22 (67.92–85.26)	93.46 (88.40–98.13)	99.77 (83.41–115.03)	57.09 (44.17–68.59)
North Africa and the Middle East	58.17 (51.33–66.57)	86.73 (83.59–89.95)	6.86 (-33.25–43.84)	-13.10 (-39.14–10.81)
South Asia	76.24 (69.83–84.43)	95.92 (90.63–101.23)	59.93 (27.68–112.61)	44.06 (20.29–77.71)
Southeast Asia, East Asia, and Oceania	14.92 (7.41–21.58)	40.17 (35.17–44.85)	-38.23 (-56.85–7.33)	-40.94 (-56.43–-7.14)
Sub-Saharan Africa	101.12 (97.06–105.71)	111.32 (107.73–115.06)	68.26 (32.31–103.72)	71.87 (41.89–107.63)
By 21 GBD regions				
Andean Latin America	72.53 (64.81–80.99)	103.32 (98.34–107.93)	83.99 (37.72–133.73)	46.37 (13.36–80.02)
Australasia	55.56 (45.24–67.66)	77.14 (70.11–83.77)	28.33 (17.13–38.94)	4.73 (-2.66–12.16)
Caribbean	55.25 (47.40–63.49)	60.99 (57.80–64.35)	44.57 (20.75–74.27)	27.04 (5.78–53.81)
Central Asia	57.86 (51.92–63.96)	61.36 (57.87–65.18)	86.44 (58.83–114.54)	68.85 (45.23–92.40)
Central Europe	-2.15 (-6.18–2.05)	-9.91 (-11.56–-8.23)	-9.95 (-17.64–-1.95)	-29.59 (-35.31–-23.81)
Central Latin America	107.98 (97.00–119.61)	121.96 (115.99–127.74)	181.66 (150.14–217.01)	116.16 (93.04–142.57)
Central Sub-Saharan Africa	144.47 (134.94–155.60)	150.39 (143.27–157.22)	106.60 (39.55–222.11)	91.92 (29.37–197.39)
East Asia	-1.96 (-9.76–5.29)	21.31 (15.98–26.58)	-50.40 (-67.05–-11.53)	-55.40 (-69.04–-25.88)
Eastern Europe	10.46 (5.24–16.29)	5.22 (2.66–7.67)	26.72 (15.70–38.86)	11.52 (3.15–21.18)
Eastern Sub-Saharan Africa	92.18 (87.41–97.48)	104.27 (99.78–108.57)	51.80 (20.25–85.85)	56.16 (29.58–90.04)
High-income Asia Pacific	33.80 (26.29–42.69)	93.64 (87.12–100.25)	-0.91 (-13.34–10.29)	-16.04 (-23.83–-8.73)
High-income North America	48.04 (40.98–57.40)	68.10 (61.02–75.25)	57.14 (48.11–63.63)	26.00 (20.57–30.50)
North Africa and Middle East	58.17 (51.33–66.57)	86.73 (83.59–89.95)	6.86 (-33.25–43.84)	-13.10 (-39.14–10.81)
Oceania	108.58 (100.30–118.22)	119.00 (114.01–124.27)	79.47 (38.33–135.79)	77.48 (38.45–130.48)
South Asia	76.24 (69.83–84.43)	95.92 (90.63–101.23)	59.93 (27.68–112.61)	44.06 (20.29–77.71)
Southeast Asia	74.65 (68.69–80.82)	89.96 (85.36–94.41)	58.19 (23.91–96.46)	44.42 (20.31–73.84)
Southern Latin America	11.17 (4.86–17.35)	33.61 (27.69–45.42)	-22.56 (-29.50–-14.79)	-31.12 (-36.27–-25.51)
Southern Sub-Saharan Africa	71.25 (64.90–78.28)	79.04 (74.83–83.00)	131.14 (94.14–170.65)	111.45 (84.31–143.03)
Tropical Latin America	55.52 (45.87–65.89)	69.08 (62.59–75.43)	73.14 (62.75–82.47)	35.07 (28.70–41.07)
Western Europe	11.60 (7.36–15.92)	20.94 (18.90–23.15)	7.34 (-0.89–13.29)	-17.05 (-21.30–-13.54)
Western Sub-Saharan Africa	106.06 (101.19–111.20)	116.15 (112.01–119.93)	66.02 (24.03–114.32)	77.23 (38.20–120.85)

DALY = disability-adjusted life years; GBD = Global Burden of Disease; SDI = sociodemographic index

© 2025 GBD 2021 Global Subarachnoid Hemorrhage Risk Factors Collaborators. *JAMA Neurology*.

Supplementary Table 14. Percentage changes (with 95% uncertainty intervals) in the absolute number of SAH incidents, prevalent cases, deaths and DALYs in 204 countries and territories of the world between 1990 and 2021.

	Changes in % (95% uncertainty interval) between 1990 and 2021			
	Incidence	Prevalence	Deaths	DALY
Afghanistan	97.95 (71.44–127.37)	99.52 (89.46–110.76)	10.04 (-35.70–76.38)	36.83 (-6.28–94.64)
Albania	34.52 (22.02–46.88)	18.42 (13.03–23.72)	27.09 (-20.15–99.65)	-9.45 (-38.95–31.72)
Algeria	63.87 (53.35–76.55)	90.63 (83.97–97.69)	51.07 (-10.80–143.10)	16.14 (-21.03–68.65)
American Samoa	27.90 (19.20–37.55)	45.85 (40.68–51.15)	63.56 (20.44–129.39)	35.97 (2.14–85.04)
Andorra	91.34 (78.59–105.69)	104.02 (95.36–112.88)	49.84 (-5.79–134.89)	20.88 (-19.45–77.83)
Angola	164.37 (149.76–178.76)	183.26 (172.86–192.22)	110.21 (44.39–218.55)	99.40 (40.34–204.34)
Antigua and Barbuda	56.06 (44.41–67.99)	71.48 (65.00–78.55)	15.76 (4.20–29.32)	15.61 (4.26–27.79)
Argentina	-0.92 (-8.19–6.30)	20.97 (14.18–33.14)	-38.48 (-44.90–-31.24)	-42.97 (-47.97–-37.36)
Armenia	-11.21 (-17.19–-4.77)	1.16 (-2.81–4.81)	-15.41 (-27.75–-0.74)	-22.99 (-32.75–-11.86)
Australia	64.85 (51.13–81.39)	84.20 (75.81–92.44)	32.59 (20.23–45.14)	7.43 (-0.35–15.96)
Austria	-0.51 (-8.82–13.15)	25.14 (19.08–32.93)	8.79 (-3.02–19.20)	-11.01 (-17.92–-4.78)
Azerbaijan	44.46 (29.23–62.46)	74.51 (67.80–82.20)	28.98 (-20.48–98.68)	28.36 (-10.12–76.77)
Bahamas	85.46 (72.65–99.88)	102.06 (94.23–109.47)	84.97 (46.39–140.14)	67.19 (35.56–114.83)
Bahrain	163.19 (140.86–187.17)	218.91 (205.42–230.69)	76.99 (18.95–152.84)	71.32 (22.25–134.74)
Bangladesh	111.32 (93.88–135.63)	122.39 (113.05–133.28)	44.14 (-4.14–120.16)	20.29 (-17.89–70.21)
Barbados	30.17 (20.86–41.28)	49.40 (43.77–55.32)	37.60 (7.99–73.46)	28.26 (1.06–61.16)
Belarus	-4.24 (-10.60–2.30)	2.77 (-1.75–7.88)	12.94 (-18.14–54.07)	1.62 (-25.30–33.83)
Belgium	24.61 (16.52–33.55)	20.60 (15.91–25.77)	40.20 (26.37–51.92)	5.99 (-1.57–13.38)
Belize	151.43 (135.48–170.29)	177.83 (168.16–186.92)	108.87 (78.32–142.85)	87.43 (62.35–115.85)
Benin	141.13 (128.20–154.55)	158.03 (149.92–165.66)	99.97 (41.86–182.90)	115.40 (58.90–197.20)
Bermuda	12.60 (4.45–21.94)	34.41 (29.97–39.17)	8.28 (-12.73–35.30)	-11.53 (-27.78–6.53)
Bhutan	42.54 (33.35–53.59)	60.18 (53.69–68.11)	27.73 (-12.37–102.93)	4.26 (-26.47–54.50)
Bolivia (Plurinational State of)	70.77 (60.17–83.80)	92.82 (86.44–98.78)	51.28 (3.18–115.99)	21.29 (-13.10–71.52)
Bosnia and Herzegovina	-12.67 (-20.24–-4.07)	-18.29 (-21.71–-14.68)	-40.77 (-62.17–-10.37)	-52.66 (-68.33–-33.29)
Botswana	114.57 (98.98–129.32)	130.49 (122.45–139.18)	35.88 (-5.44–110.28)	47.45 (5.25–112.88)
Brazil	54.64 (44.91–65.29)	68.10 (61.55–74.45)	72.84 (62.35–81.96)	34.47 (27.99–40.85)
Brunei Darussalam	97.37 (74.35–132.49)	140.25 (129.02–150.70)	57.99 (16.84–120.47)	52.87 (15.40–107.47)
Bulgaria	8.78 (1.79–16.56)	-10.46 (-14.14–-6.78)	41.84 (21.62–68.44)	1.87 (-12.57–19.47)
Burkina Faso	112.01 (101.43–125.16)	119.08 (111.51–126.79)	91.95 (40.53–182.44)	97.96 (50.86–169.84)
Burundi	97.89 (87.61–108.47)	106.99 (100.15–115.25)	21.69 (-34.92–120.44)	26.15 (-22.10–118.31)
Côte d'Ivoire	116.40 (104.96–130.11)	135.82 (128.03–143.55)	121.18 (48.62–226.66)	111.03 (49.87–209.60)
Cabo Verde	70.34 (52.77–85.11)	92.05 (86.45–99.35)	53.98 (4.17–141.20)	48.17 (10.74–103.70)
Cambodia	98.53 (85.37–111.72)	116.83 (108.10–125.24)	50.30 (2.04–116.14)	34.56 (-2.51–84.06)
Cameroon	180.72 (168.06–196.87)	191.76 (179.86–202.29)	162.66 (74.06–287.88)	175.65 (92.31–290.09)
Canada	36.27 (26.58–47.41)	51.11 (45.04–56.99)	32.59 (18.46–45.67)	5.45 (-3.80–14.37)
Central African Republic	101.52 (90.78–113.75)	103.51 (96.53–110.38)	72.20 (9.09–159.22)	69.89 (9.80–154.33)
Chad	149.36 (137.04–163.22)	140.37 (131.60–148.99)	136.46 (76.67–241.67)	161.17 (99.70–259.66)
Chile	67.98 (55.96–80.31)	100.02 (90.43–116.26)	74.38 (58.17–90.60)	35.68 (25.44–46.69)
China	-3.60 (-11.38–3.77)	19.80 (14.43–25.17)	-51.58 (-68.15–-13.71)	-56.65 (-70.18–-28.03)
Colombia	111.34 (93.25–129.90)	121.36 (113.06–129.78)	147.26 (104.97–191.65)	85.03 (55.09–116.80)
Comoros	81.48 (71.17–90.78)	101.95 (95.67–108.68)	48.89 (-4.49–125.62)	37.68 (-4.78–101.68)
Congo	119.99 (107.38–134.45)	138.55 (130.10–148.14)	83.14 (25.55–178.77)	84.35 (32.26–176.33)
Cook Islands	-4.03 (-10.25–2.79)	21.11 (17.29–25.31)	-36.41 (-62.94–5.78)	-44.83 (-64.02–-16.38)
Costa Rica	94.77 (79.38–110.12)	127.84 (119.97–135.37)	179.71 (142.63–219.00)	134.51 (105.34–163.61)
Croatia	-20.51 (-27.28–-14.24)	-20.81 (-24.18–-17.02)	-21.73 (-33.32–-9.61)	-42.68 (-50.71–-33.94)
Cuba	11.72 (2.60–21.39)	28.58 (23.67–33.40)	16.99 (-1.07–36.69)	-4.21 (-17.17–10.93)
Cyprus	46.85 (36.06–58.92)	70.91 (64.60–76.99)	-16.39 (-43.28–16.27)	-19.15 (-43.35–5.59)
Czechia	-11.10 (-16.58–-5.56)	-1.52 (-5.40–2.06)	-37.96 (-46.04–-27.64)	-46.87 (-53.19–-38.66)
Democratic People's Republic of Korea	46.88 (37.56–55.55)	49.07 (43.52–55.19)	37.58 (-15.77–148.58)	27.07 (-21.39–123.43)
Democratic Republic of the Congo	146.24 (133.39–160.23)	147.86 (139.04–156.36)	114.50 (27.08–251.91)	94.56 (15.94–221.27)
Denmark	-7.62 (-15.60–0.85)	4.82 (0.97–9.03)	-16.00 (-24.71–-8.00)	-35.02 (-40.68–-29.46)
Djibouti	258.30 (237.35–282.01)	290.49 (276.46–306.07)	224.17 (110.11–468.60)	206.05 (109.14–392.62)
Dominica	3.68 (-3.68–12.18)	13.86 (10.06–17.86)	1.78 (-18.60–29.87)	3.88 (-16.18–31.50)
Dominican Republic	123.84 (104.62–142.89)	125.69 (116.78–134.91)	73.82 (18.08–144.65)	41.93 (4.21–90.22)
Ecuador	111.75 (95.40–127.84)	133.38 (125.37–141.62)	235.61 (163.95–330.04)	137.52 (89.94–196.99)
Egypt	54.65 (46.74–62.49)	77.00 (70.44–82.86)	-25.61 (-56.32–18.18)	-41.30 (-61.11–-16.62)
El Salvador	13.02 (4.98–22.08)	34.34 (30.32–38.95)	8.42 (-29.97–49.06)	-12.31 (-39.64–15.41)
Equatorial Guinea	133.92 (116.02–154.01)	170.55 (158.90–184.18)	2.42 (-47.57–109.96)	18.27 (-31.62–118.64)
Eritrea	104.38 (90.95–119.35)	121.57 (113.00–129.50)	49.90 (6.47–124.25)	46.90 (8.21–114.76)
Estonia	-30.20 (-35.04–-24.83)	-30.41 (-33.53–-27.37)	-10.49 (-25.14–2.48)	-38.93 (-47.79–-29.61)
Eswatini	63.72 (53.68–73.33)	73.57 (66.67–80.35)	76.53 (10.84–170.68)	79.65 (15.86–163.88)
Ethiopia	43.54 (37.72–50.48)	60.89 (54.53–67.08)	1.37 (-31.41–42.04)	3.26 (-23.88–33.75)
Fiji	29.77 (20.35–40.53)	41.20 (36.44–46.13)	34.76 (-1.69–85.39)	18.09 (-10.48–56.32)
Finland	2.91 (-8.43–12.46)	22.10 (17.54–26.37)	-9.06 (-19.09–0.70)	-28.00 (-34.45–-21.80)
France	47.88 (37.90–57.89)	46.65 (40.87–52.65)	30.62 (17.96–41.79)	12.36 (4.56–20.74)

© 2025 GBD 2021 Global Subarachnoid Hemorrhage Risk Factors Collaborators. *JAMA Neurology*.

Gabon	67.46 (58.02–77.18)	79.95 (73.10–87.53)	36.94 (-11.34–113.00)	47.05 (1.91–116.51)
Gambia	126.59 (111.55–138.76)	144.95 (136.93–153.86)	173.64 (78.34–292.76)	156.39 (75.01–255.55)
Georgia	3.50 (-3.82–11.61)	-14.40 (-18.26–-10.65)	46.99 (9.84–102.17)	15.00 (-12.45–50.70)
Germany	-5.02 (-11.48–2.56)	9.62 (5.76–13.85)	-12.21 (-23.21–-0.58)	-28.80 (-35.87–-21.38)
Ghana	128.76 (114.71–144.33)	141.33 (133.82–149.85)	107.93 (48.91–230.20)	95.87 (44.64–186.47)
Greece	28.73 (19.14–38.90)	37.40 (31.94–43.15)	42.97 (23.81–81.91)	18.21 (5.28–42.01)
Greenland	49.03 (-9.63–84.95)	28.53 (-4.91–43.59)	-33.15 (-46.85–-15.45)	-44.26 (-55.20–-31.51)
Grenada	23.62 (14.52–33.59)	41.99 (36.60–46.99)	0.19 (-19.88–29.61)	0.39 (-18.90–27.69)
Guam	44.08 (32.52–56.30)	69.37 (62.54–75.56)	25.73 (4.45–53.34)	21.72 (3.57–41.99)
Guatemala	140.61 (124.60–160.45)	162.22 (152.96–172.54)	250.11 (185.67–324.76)	183.04 (135.78–235.09)
Guinea	91.11 (81.34–102.46)	91.04 (85.03–98.28)	69.26 (18.11–145.24)	75.76 (26.26–154.43)
Guinea-Bissau	75.42 (64.88–86.06)	82.78 (76.32–90.09)	59.60 (7.59–127.05)	60.62 (11.81–129.52)
Guyana	6.20 (-0.59–13.67)	19.94 (15.78–24.70)	10.91 (-18.59–44.87)	0.20 (-25.40–30.10)
Haiti	114.83 (92.70–140.40)	116.71 (109.67–124.54)	59.76 (9.11–133.63)	44.64 (1.00–106.71)
Honduras	150.34 (133.98–167.04)	143.80 (136.21–152.55)	228.44 (125.16–371.55)	137.59 (70.79–230.24)
Hungary	-11.04 (-16.49–-5.02)	-13.54 (-17.12–-9.17)	-29.45 (-39.99–-18.49)	-42.49 (-50.59–-34.12)
Iceland	31.27 (22.77–40.80)	48.45 (43.06–53.47)	11.00 (-2.35–25.34)	-10.74 (-19.04–-1.15)
India	67.21 (61.14–75.13)	88.10 (82.48–93.58)	59.96 (25.28–117.46)	43.21 (15.82–82.09)
Indonesia	68.04 (60.53–77.00)	77.89 (71.28–83.60)	66.15 (27.02–119.51)	47.93 (18.99–89.95)
Iran (Islamic Republic of)	53.23 (42.06–63.71)	92.03 (84.29–100.10)	11.13 (-34.49–46.54)	-10.77 (-40.65–15.19)
Iraq	64.19 (53.73–75.13)	112.50 (103.95–121.13)	37.01 (-20.99–100.86)	19.56 (-20.45–64.97)
Ireland	9.45 (1.35–19.28)	23.87 (18.73–29.82)	-11.40 (-21.05–-2.35)	-26.80 (-32.67–-21.18)
Israel	90.21 (79.56–101.95)	101.66 (93.86–109.48)	94.10 (72.47–116.05)	42.02 (30.44–54.50)
Italy	4.15 (-4.35–15.21)	10.84 (7.31–14.87)	33.76 (19.83–43.02)	-11.97 (-17.88–-7.33)
Jamaica	38.71 (27.80–50.90)	45.01 (39.89–50.38)	30.19 (-3.99–70.12)	8.24 (-18.13–38.43)
Japan	34.77 (25.60–44.62)	92.52 (85.20–100.20)	9.34 (-4.07–17.78)	-7.98 (-14.49–-2.37)
Jordan	212.48 (185.64–240.02)	292.72 (277.72–306.39)	91.17 (17.63–197.71)	87.48 (32.91–155.55)
Kazakhstan	35.42 (28.11–43.64)	32.42 (27.23–37.55)	39.97 (4.26–81.94)	24.80 (-4.99–58.58)
Kenya	129.71 (121.66–138.49)	151.02 (145.23–156.42)	153.93 (97.55–239.27)	149.90 (106.51–209.12)
Kiribati	104.62 (68.49–128.13)	104.42 (95.96–112.91)	64.83 (20.52–125.69)	54.11 (15.10–106.33)
Kuwait	192.43 (167.05–219.11)	237.94 (226.07–249.21)	3.90 (-15.20–26.20)	25.91 (6.95–47.75)
Kyrgyzstan	48.90 (39.58–61.64)	54.21 (48.10–60.65)	47.68 (13.04–87.50)	47.29 (12.84–89.78)
Lao People's Democratic Republic	68.95 (59.43–80.69)	91.56 (84.08–98.96)	-4.30 (-36.33–47.33)	-5.88 (-34.02–39.48)
Latvia	-39.91 (-43.76–-35.96)	-33.19 (-36.68–-29.35)	-21.06 (-32.15–-9.08)	-40.07 (-48.55–-30.67)
Lebanon	54.88 (45.54–64.11)	82.91 (76.51–88.74)	-22.49 (-52.97–18.95)	-33.12 (-56.09–-2.00)
Lesotho	40.10 (30.08–50.37)	36.23 (31.07–41.93)	74.28 (8.66–182.64)	88.97 (20.52–184.39)
Liberia	85.53 (72.20–98.84)	97.11 (89.22–104.93)	87.10 (-0.70–234.71)	85.67 (6.60–242.34)
Libya	63.33 (47.33–77.81)	99.79 (92.84–107.44)	69.69 (-10.29–157.93)	28.14 (-23.74–88.59)
Lithuania	-24.24 (-29.66–-18.89)	-9.35 (-14.15–-2.97)	17.20 (-1.86–37.63)	-16.00 (-29.28–-1.02)
Luxembourg	40.77 (32.40–49.26)	53.78 (47.73–60.00)	-0.32 (-12.23–11.76)	-18.03 (-26.58–-8.97)
Madagascar	140.63 (126.93–156.41)	141.22 (133.13–150.32)	89.67 (29.15–165.22)	101.45 (43.83–186.26)
Malawi	85.50 (75.35–94.96)	91.82 (85.85–99.20)	80.75 (27.70–137.71)	76.57 (29.21–138.23)
Malaysia	68.28 (56.25–79.09)	111.11 (103.49–118.94)	64.22 (7.12–123.78)	56.62 (12.98–99.68)
Maldives	126.94 (102.63–149.82)	188.45 (179.26–199.15)	14.76 (-22.94–62.71)	10.98 (-19.40–50.72)
Mali	119.10 (107.97–131.33)	126.29 (119.26–133.88)	78.17 (30.69–151.24)	88.17 (44.16–149.92)
Malta	26.50 (18.16–37.57)	48.82 (42.93–55.34)	22.71 (8.83–39.76)	3.78 (-6.28–15.74)
Marshall Islands	82.17 (67.04–100.57)	89.49 (83.03–96.34)	54.55 (15.45–108.07)	55.09 (16.68–105.71)
Mauritania	76.46 (67.86–85.65)	93.02 (86.46–99.18)	41.03 (-10.50–114.28)	41.30 (-1.87–100.06)
Mauritius	50.04 (38.51–63.35)	75.83 (68.33–85.19)	61.31 (46.71–74.93)	41.34 (29.01–54.24)
Mexico	114.11 (101.44–128.46)	122.73 (112.96–131.28)	231.40 (195.08–270.00)	149.91 (124.87–175.25)
Micronesia (Federated States of)	26.50 (17.10–39.40)	34.84 (30.14–39.89)	-3.41 (-30.95–37.76)	-5.12 (-31.88–32.12)
Monaco	-10.81 (-16.65–-3.62)	10.45 (6.04–14.85)	-43.87 (-62.80–-12.48)	-42.24 (-59.70–-16.36)
Mongolia	121.48 (106.21–138.82)	111.91 (102.69–122.63)	107.85 (24.80–242.96)	115.41 (32.64–243.33)
Montenegro	10.38 (3.10–17.90)	12.72 (8.30–17.53)	31.49 (-10.27–78.22)	3.73 (-23.77–32.65)
Morocco	47.55 (37.92–58.01)	61.99 (56.09–68.74)	36.16 (-11.43–125.51)	3.13 (-24.39–49.32)
Mozambique	112.02 (96.41–128.27)	108.03 (100.26–116.67)	116.17 (47.94–210.64)	129.35 (63.08–213.26)
Myanmar	32.68 (25.08–41.82)	51.16 (45.83–57.57)	-3.91 (-37.50–42.56)	-12.45 (-40.53–23.03)
Namibia	74.03 (64.66–84.38)	86.19 (80.09–92.82)	73.53 (23.71–145.39)	72.62 (27.83–134.07)
Nauru	20.11 (11.05–31.33)	20.02 (15.94–24.29)	7.99 (-27.69–64.11)	6.08 (-25.98–54.47)
Nepal	72.59 (61.09–84.22)	86.69 (79.43–95.38)	39.86 (-5.19–111.12)	21.10 (-14.83–74.61)
Netherlands	2.18 (-4.70–9.44)	19.74 (14.86–24.31)	-4.35 (-12.67–3.55)	-25.51 (-31.12–-20.50)
New Zealand	18.72 (9.92–28.71)	44.13 (38.50–49.80)	12.12 (2.07–23.24)	-5.21 (-12.27–1.92)
Nicaragua	104.17 (90.88–119.37)	136.73 (127.99–144.95)	83.39 (32.10–137.23)	52.88 (18.96–91.81)
Niger	168.78 (152.98–186.57)	173.24 (164.74–182.58)	127.92 (56.09–238.36)	114.18 (56.52–214.18)
Nigeria	85.28 (79.88–90.67)	95.14 (89.67–100.18)	16.48 (-21.40–76.57)	37.95 (-4.44–98.28)
Niue	-36.29 (-40.11–-31.80)	-20.21 (-23.00–-17.43)	-42.46 (-59.88–-20.70)	-29.86 (-49.37–-5.34)
North Macedonia	49.87 (38.59–62.88)	29.84 (24.63–35.02)	25.00 (-12.46–70.45)	2.95 (-24.37–35.23)
Northern Mariana Islands	32.56 (20.02–46.94)	51.24 (45.95–57.07)	49.94 (8.60–112.81)	17.45 (-13.83–60.54)
Norway	1.17 (-4.19–7.03)	17.97 (14.69–21.18)	-34.30 (-39.35–-30.23)	-41.84 (-45.18–-38.31)
Oman	144.08 (80.71–174.34)	172.13 (163.19–182.74)	-3.57 (-46.96–52.34)	3.89 (-34.09–50.25)
Pakistan	103.72 (92.54–115.27)	121.29 (112.26–129.41)	95.54 (48.97–153.89)	105.66 (61.59–160.57)

© 2025 GBD 2021 Global Subarachnoid Hemorrhage Risk Factors Collaborators. *JAMA Neurology*.

Palau	42.44 (32.98–53.95)	75.95 (69.66–82.54)	38.16 (-5.32–90.42)	28.42 (-8.36–72.73)
Palestine	131.47 (116.53–147.01)	178.94 (168.61–188.92)	23.19 (-17.45–76.27)	23.94 (-4.43–62.95)
Panama	116.55 (101.85–132.11)	130.85 (122.97–138.62)	184.61 (118.20–250.15)	138.83 (91.20–188.41)
Papua New Guinea	145.46 (131.01–162.42)	160.53 (151.66–169.35)	97.70 (40.48–192.55)	98.94 (45.10–184.56)
Paraguay	94.48 (79.36–108.78)	111.10 (103.16–118.83)	86.31 (27.18–152.27)	63.61 (17.40–118.23)
Peru	53.65 (44.46–63.88)	91.60 (85.01–98.31)	47.04 (-7.99–108.07)	25.88 (-13.22–71.14)
Philippines	190.47 (169.21–214.52)	187.09 (174.53–198.96)	182.91 (122.64–247.87)	153.12 (113.84–199.55)
Poland	-18.88 (-23.09–-13.60)	-11.02 (-13.81–-8.16)	-22.82 (-29.91–-15.63)	-37.36 (-42.77–-31.75)
Portugal	8.74 (-9.20–17.68)	1.84 (-2.15–6.43)	13.50 (1.34–23.63)	-19.47 (-25.52–-13.21)
Puerto Rico	5.93 (-1.68–14.40)	23.28 (18.35–27.63)	20.57 (-0.52–43.10)	-6.81 (-20.64–8.84)
Qatar	523.78 (467.33–581.10)	626.21 (601.17–652.08)	183.66 (56.79–387.97)	218.26 (92.67–389.62)
Republic of Korea	26.28 (14.25–40.62)	95.00 (85.00–106.40)	-31.75 (-49.93–-9.22)	-39.74 (-53.76–-22.64)
Republic of Moldova	-12.82 (-17.67–-7.20)	-3.67 (-7.87–1.73)	5.03 (-10.12–23.76)	-7.60 (-21.03–8.60)
Romania	24.66 (17.25–32.55)	-10.53 (-14.05–-6.76)	30.20 (6.50–59.63)	-1.20 (-19.66–17.52)
Russian Federation	22.00 (15.29–29.59)	8.88 (6.02–11.90)	53.76 (41.95–65.71)	25.47 (16.22–35.04)
Rwanda	51.26 (43.54–59.92)	70.14 (64.80–76.38)	-13.54 (-45.20–34.46)	-15.68 (-42.85–26.43)
Saint Kitts and Nevis	37.73 (26.29–48.87)	56.63 (50.80–62.45)	2.75 (-19.87–34.56)	5.68 (-16.99–37.97)
Saint Lucia	64.71 (52.54–78.06)	94.56 (86.50–102.36)	41.80 (13.98–72.35)	28.57 (4.47–55.41)
Saint Vincent and the Grenadines	21.26 (12.35–30.26)	40.53 (35.40–46.78)	38.03 (17.16–60.03)	24.95 (6.06–43.56)
Samoa	40.16 (31.71–50.53)	49.76 (44.78–54.80)	18.29 (-14.05–58.73)	14.25 (-13.23–49.42)
San Marino	42.53 (33.36–51.34)	62.78 (56.01–69.79)	-17.28 (-47.05–25.64)	-11.32 (-36.25–17.82)
Sao Tome and Principe	79.16 (66.86–91.64)	86.03 (79.72–93.22)	83.44 (24.18–157.85)	88.94 (33.90–167.02)
Saudi Arabia	151.00 (124.99–173.93)	204.79 (192.50–216.57)	102.69 (-3.10–246.90)	95.34 (7.66–205.86)
Senegal	91.41 (80.17–104.80)	113.51 (106.29–120.43)	92.08 (36.89–165.86)	76.82 (29.15–146.80)
Serbia	9.04 (1.19–18.49)	-20.66 (-23.70–-17.89)	-23.13 (-45.23–5.02)	-39.56 (-55.92–-18.68)
Seychelles	37.34 (28.83–46.93)	69.55 (63.21–75.57)	-9.71 (-37.18–15.10)	-0.29 (-21.31–21.01)
Sierra Leone	89.58 (79.40–100.81)	95.96 (88.65–103.19)	77.31 (21.80–157.31)	76.49 (23.57–153.55)
Singapore	101.28 (84.26–118.74)	158.38 (148.32–168.62)	0.68 (-10.24–10.41)	9.74 (-0.72–19.97)
Slovakia	-17.91 (-22.65–-13.09)	0.19 (-3.28–4.03)	-21.83 (-39.46–0.12)	-29.67 (-43.28–-12.76)
Slovenia	-2.13 (-8.33–4.53)	4.56 (0.24–8.87)	-21.80 (-34.05–-9.06)	-40.85 (-48.71–-32.44)
Solomon Islands	191.76 (141.33–236.22)	155.38 (145.31–165.81)	112.91 (43.14–257.81)	108.61 (45.37–237.25)
Somalia	155.04 (141.69–169.91)	154.21 (145.43–165.13)	84.99 (6.09–186.11)	88.24 (20.11–186.87)
South Africa	59.87 (52.71–66.24)	73.48 (68.65–77.63)	117.45 (85.18–150.85)	79.69 (57.13–102.49)
South Sudan	49.45 (40.19–57.66)	53.48 (48.51–58.85)	27.56 (-26.24–128.62)	36.61 (-14.60–129.56)
Spain	51.61 (41.38–63.07)	52.70 (46.36–60.40)	100.43 (76.38–120.42)	33.29 (24.19–43.49)
Sri Lanka	47.00 (36.88–57.19)	61.81 (55.95–67.80)	33.27 (-17.70–105.42)	18.51 (-13.86–62.45)
Sudan	50.83 (42.30–60.27)	79.84 (73.67–86.54)	-1.15 (-35.35–54.16)	-10.10 (-37.29–35.13)
Suriname	77.22 (65.11–90.29)	85.52 (78.87–93.04)	71.37 (24.21–128.93)	51.41 (14.33–94.48)
Sweden	-4.15 (-10.47–2.84)	6.96 (2.66–11.45)	-32.96 (-40.56–-25.03)	-42.01 (-47.85–-36.35)
Switzerland	14.88 (6.32–24.90)	42.10 (36.72–48.59)	-0.78 (-11.81–8.95)	-23.13 (-29.56–-15.99)
Syrian Arab Republic	2.52 (-30.00–21.02)	35.86 (30.01–41.61)	-4.90 (-51.53–71.14)	-34.61 (-61.36–9.54)
Taiwan (Province of China)	25.46 (16.07–36.90)	49.63 (44.41–55.28)	-6.14 (-44.56–39.94)	-32.33 (-57.41–-4.03)
Tajikistan	66.92 (48.04–88.56)	70.57 (62.91–77.48)	74.35 (56.50–89.60)	46.53 (34.44–56.96)
Thailand	130.04 (117.22–143.61)	125.56 (116.79–133.83)	58.45 (3.17–145.18)	66.62 (14.78–150.95)
Timor-Leste	61.71 (49.81–76.32)	81.54 (73.38–89.75)	61.96 (11.92–119.29)	42.74 (3.16–90.77)
Togo	96.81 (84.69–110.23)	109.84 (101.93–117.91)	105.20 (33.87–201.50)	65.58 (16.32–137.96)
Tokelau	134.27 (118.64–152.87)	158.97 (150.24–167.91)	172.61 (79.21–300.83)	148.23 (73.83–265.51)
Tonga	-21.18 (-25.70–-15.48)	-10.31 (-13.24–-7.24)	-36.71 (-61.58–-6.67)	-28.25 (-52.44–3.24)
Trinidad and Tobago	7.70 (2.09–13.79)	15.49 (11.74–19.12)	10.29 (-23.35–53.67)	-0.85 (-26.34–31.98)
Tunisia	40.49 (30.39–49.48)	52.35 (46.86–58.42)	55.57 (18.81–103.52)	36.69 (6.51–76.11)
Turkey	50.52 (40.26–61.63)	75.32 (68.42–82.39)	38.72 (-25.42–151.79)	9.51 (-32.77–70.64)
Turkmenistan	141.00 (123.61–158.24)	135.41 (123.20–146.65)	209.47 (132.82–312.06)	186.46 (119.25–274.57)
Tuvalu	22.27 (13.66–32.83)	31.38 (26.77–36.50)	-15.70 (-37.28–11.22)	-22.05 (-41.55–0.88)
Uganda	120.45 (108.33–132.86)	128.14 (120.32–136.02)	46.05 (5.92–126.43)	58.72 (18.65–129.42)
Ukraine	-10.62 (-16.73–-4.27)	-1.08 (-4.98–3.49)	-20.39 (-42.07–2.98)	-17.59 (-40.09–6.85)
United Arab Emirates	476.14 (413.99–543.96)	573.02 (542.06–599.79)	230.31 (90.09–498.41)	237.06 (131.06–471.23)
United Kingdom	0.28 (-4.02–5.67)	6.48 (4.32–8.81)	-15.29 (-20.74–-11.76)	-32.92 (-35.44–-30.68)
United Republic of Tanzania	110.30 (99.66–124.10)	121.77 (113.93–130.20)	52.12 (5.28–124.42)	57.85 (17.41–116.73)
United States of America	49.72 (42.20–60.09)	70.58 (62.72–78.91)	59.91 (50.98–66.93)	28.35 (22.63–33.13)
United States Virgin Islands	5.78 (-3.41–15.45)	19.33 (14.21–24.59)	-26.54 (-46.93–0.92)	-39.77 (-54.67–-18.22)
Uruguay	-0.45 (-8.09–7.50)	3.31 (-2.16–12.52)	-10.77 (-19.42–-0.52)	-27.30 (-33.21–-19.93)
Uzbekistan	106.08 (88.15–124.01)	126.27 (115.85–137.77)	318.28 (222.00–439.08)	224.52 (163.16–306.45)
Vanuatu	155.70 (140.80–172.49)	161.79 (152.02–170.63)	104.43 (41.01–185.40)	97.55 (38.28–167.74)
Venezuela (Bolivarian Republic of)	93.98 (80.40–110.06)	125.08 (116.53–134.02)	172.25 (97.69–256.09)	117.21 (61.31–180.73)
Viet Nam	81.05 (69.12–94.89)	97.89 (90.91–106.28)	45.06 (-13.06–120.53)	45.45 (-3.45–111.43)
Yemen	121.56 (108.24–135.97)	146.03 (138.80–154.38)	77.08 (15.72–162.35)	48.53 (-4.12–126.28)
Zambia	143.07 (130.21–158.08)	146.16 (137.80–155.31)	100.30 (29.86–196.01)	105.87 (42.17–189.91)
Zimbabwe	112.15 (97.81–127.96)	98.82 (91.03–107.11)	183.63 (102.56–285.63)	207.11 (122.08–316.97)

DALY = disability-adjusted life years

© 2025 GBD 2021 Global Subarachnoid Hemorrhage Risk Factors Collaborators. *JAMA Neurology*.

© 2025 GBD 2021 Global Subarachnoid Hemorrhage Risk Factors Collaborators. *JAMA Neurology*.

Supplementary Table 15. Percentage changes (with 95% uncertainty intervals) in the age-standardized incidence, prevalence, mortality, and DALY rates of SAH per 100,000 people between 1990 and 2021 by five country-specific sociodemographic index (SDI) levels, seven GBD super-regions and 21 GBD regions.

	Changes in % (95% UI) between 1990 and 2021			
	Incidence	Prevalence	Mortality	DALY rate
By 5 SDI levels				
Low SDI	-17.96 (-20.62–-15.00)	-12.24 (-13.88–-10.49)	-27.43 (-42.95–-11.20)	-27.48 (-40.94–-14.72)
Low-middle SDI	-19.37 (-22.25–-15.99)	-11.82 (-13.71–-10.15)	-31.80 (-45.75–-11.92)	-33.22 (-43.95–-18.30)
Middle SDI	-38.35 (-42.70–-34.57)	-22.89 (-24.84–-21.24)	-70.78 (-78.64–-50.08)	-67.91 (-75.47–-50.76)
High-middle SDI	-35.96 (-38.55–-33.33)	-22.52 (-24.10–-21.01)	-62.06 (-70.97–-46.44)	-62.22 (-69.83–-50.32)
High SDI	-22.41 (-24.42–-19.96)	-5.22 (-7.09–-3.47)	-44.96 (-48.41–-42.38)	-46.92 (-49.37–-44.86)
By 7 GBD super-regions				
Central Europe, Eastern Europe, and Central Asia	-9.40 (-12.26–-6.56)	-9.43 (-10.92–-8.05)	-13.14 (-19.47–-6.65)	-18.30 (-23.56–-13.11)
High-income	-18.82 (-21.04–-16.10)	-2.59 (-4.45–-0.93)	-40.09 (-43.22–-37.71)	-43.36 (-45.43–-41.57)
Latin America and Caribbean	-17.50 (-20.30–-14.66)	-15.90 (-17.32–-14.49)	-16.98 (-23.82–-10.44)	-26.24 (-32.51–-20.79)
North Africa and the Middle East	-31.70 (-34.57–-28.40)	-22.08 (-23.48–-20.72)	-54.39 (-72.71–-35.22)	-57.91 (-72.30–-45.39)
South Asia	-22.64 (-25.89–-19.19)	-11.79 (-14.16–-9.69)	-33.95 (-49.58–-7.69)	-33.80 (-46.98–-14.05)
Southeast Asia, East Asia, and Oceania	-47.38 (-51.08–-43.92)	-28.25 (-30.33–-26.35)	-76.92 (-84.14–-58.84)	-73.87 (-80.89–-57.75)
Sub-Saharan Africa	-15.52 (-17.42–-13.28)	-11.96 (-13.54–-10.34)	-26.96 (-44.20–-11.22)	-25.77 (-40.95–-12.03)
By 21 GBD regions				
Andean Latin America	-24.63 (-27.64–-21.07)	-17.54 (-19.51–-15.36)	-25.66 (-44.86–-4.72)	-32.37 (-48.04–-15.88)
Australasia	-20.70 (-25.39–-14.72)	-9.33 (-12.39–-6.29)	-45.30 (-49.67–-41.04)	-48.43 (-51.85–-44.79)
Caribbean	-9.25 (-12.62–-5.45)	-9.77 (-11.32–-8.00)	-22.34 (-35.47–-7.26)	-21.77 (-35.07–-5.67)
Central Asia	-3.58 (-7.17–0.05)	-7.27 (-9.20–-5.05)	13.39 (-3.34–30.04)	-0.55 (-14.23–13.10)
Central Europe	-23.32 (-25.72–-21.31)	-19.98 (-21.13–-18.76)	-37.60 (-42.97–-31.95)	-43.87 (-48.41–-39.42)
Central Latin America	-6.56 (-9.30–-3.61)	-8.76 (-10.26–-7.15)	8.86 (-3.00–22.55)	-2.70 (-13.03–9.24)
Central Sub-Saharan Africa	-6.05 (-10.05–-2.18)	-3.17 (-6.02–-0.20)	-12.26 (-40.89–30.96)	-14.96 (-39.03–25.85)
East Asia	-55.53 (-59.16–-52.31)	-35.17 (-37.34–-33.22)	-82.22 (-88.28–-67.16)	-80.65 (-86.69–-67.31)
Eastern Europe	-2.43 (-6.36–1.62)	-3.49 (-5.66–-1.53)	-1.20 (-9.42–8.41)	-3.29 (-10.59–5.00)
Eastern Sub-Saharan Africa	-19.35 (-21.50–-16.65)	-15.02 (-16.86–-13.16)	-35.00 (-49.74–-20.40)	-32.76 (-45.87–-19.23)
High-income Asia Pacific	-18.40 (-22.66–-13.33)	1.07 (-1.77–3.85)	-56.38 (-60.36–-52.57)	-52.24 (-56.01–-48.68)
High-income North America	-10.93 (-14.22–-6.60)	3.26 (-1.06–7.70)	-16.83 (-21.07–-13.40)	-26.05 (-29.05–-23.27)
North Africa and Middle East	-31.70 (-34.57–-28.40)	-22.08 (-23.48–-20.72)	-54.39 (-72.71–-35.22)	-57.91 (-72.30–-45.39)
Oceania	-15.82 (-19.72–-11.72)	-9.42 (-11.62–-7.32)	-30.23 (-45.93–-9.75)	-27.06 (-42.62–-6.78)
South Asia	-22.64 (-25.89–-19.19)	-11.79 (-14.16–-9.69)	-33.95 (-49.58–-7.69)	-33.80 (-46.98–-14.05)
Southeast Asia	-20.10 (-22.45–-17.40)	-13.59 (-15.09–-11.93)	-35.20 (-50.67–-18.90)	-34.46 (-46.40–-21.13)
Southern Latin America	-34.03 (-37.72–-30.38)	-21.21 (-24.59–-14.95)	-58.19 (-61.82–-54.02)	-59.71 (-62.73–-56.45)
Southern Sub-Saharan Africa	-9.48 (-12.31–-5.59)	-10.15 (-12.19–-8.16)	12.72 (-6.81–32.32)	7.14 (-6.58–23.15)
Tropical Latin America	-26.67 (-30.40–-22.56)	-25.61 (-27.85–-23.65)	-27.03 (-31.04–-23.15)	-37.37 (-40.22–-34.44)
Western Europe	-22.28 (-24.30–-19.96)	-13.57 (-14.94–-12.19)	-40.07 (-43.45–-37.30)	-46.29 (-48.45–-44.13)
Western Sub-Saharan Africa	-17.26 (-18.99–-15.32)	-12.61 (-14.32–-11.01)	-30.19 (-48.64–-9.80)	-27.59 (-44.89–-8.59)

DALY = disability-adjusted life years; GBD = Global Burden of Disease; SDI = sociodemographic index

© 2025 GBD 2021 Global Subarachnoid Hemorrhage Risk Factors Collaborators. *JAMA Neurology*.

Supplementary Table 16. Percentage changes (with 95% uncertainty intervals) in the age-standardized incidence, prevalence, mortality and DALY rates of subarachnoid hemorrhage per 100,000 people in 204 countries and territories of the world between 1990 and 2021.

	Changes in % (95% uncertainty interval) between 1990 and 2021			
	Incidence	Prevalence	Mortality	DALY rate
Afghanistan	-14.20 (-25.26–0.10)	-17.49 (-20.68–-14.16)	-33.93 (-61.07–8.46)	-33.63 (-58.41–-1.93)
Albania	-6.97 (-11.82–-1.57)	-9.58 (-12.81–-6.30)	-36.25 (-59.55–-0.29)	-39.75 (-58.52–-14.08)
Algeria	-33.33 (-36.93–-28.84)	-22.87 (-25.37–-19.96)	-43.76 (-68.25–-8.95)	-48.34 (-66.66–-24.03)
American Samoa	-9.35 (-14.82–-3.53)	-5.13 (-8.34–-1.90)	-13.37 (-35.04–17.85)	-10.39 (-31.75–20.59)
Andorra	-12.84 (-16.96–-7.58)	-9.08 (-12.60–-5.22)	-46.01 (-65.72–-14.90)	-48.80 (-65.87–-24.62)
Angola	-18.50 (-23.56–-13.36)	-10.79 (-14.24–-7.35)	-28.08 (-51.24–9.37)	-28.74 (-48.57–2.50)
Antigua and Barbuda	-17.50 (-22.96–-11.21)	-12.64 (-15.83–-9.38)	-39.76 (-45.93–-32.72)	-43.26 (-48.63–-37.22)
Argentina	-38.35 (-43.00–-33.87)	-24.69 (-28.71–-17.99)	-64.26 (-67.94–-60.09)	-64.81 (-67.90–-61.31)
Armenia	-27.99 (-33.01–-22.98)	-16.25 (-19.45–-13.14)	-47.53 (-55.68–-38.37)	-43.83 (-51.32–-35.51)
Australia	-16.49 (-22.80–-8.41)	-6.45 (-10.03–-2.62)	-44.65 (-49.31–-39.63)	-47.66 (-51.11–-43.70)
Austria	-27.65 (-33.00–-18.65)	-12.69 (-16.63–-8.26)	-37.00 (-43.11–-31.38)	-42.93 (-47.13–-38.62)
Azerbaijan	-19.96 (-29.40–-9.14)	-11.04 (-14.40–-7.36)	-31.76 (-57.77–5.40)	-34.39 (-54.02–-9.77)
Bahamas	-9.52 (-14.80–-3.83)	-5.88 (-9.27–-2.51)	-22.46 (-39.07–-0.22)	-23.08 (-37.93–-1.55)
Bahrain	-29.74 (-34.15–-25.25)	-19.96 (-22.83–-17.15)	-59.40 (-72.86–-43.36)	-59.62 (-71.48–-45.61)
Bangladesh	-10.74 (-17.65–-1.20)	-9.46 (-13.24–-5.09)	-42.89 (-61.69–-8.54)	-45.11 (-62.54–-17.04)
Barbados	-16.53 (-21.79–-10.34)	-7.63 (-10.73–-4.01)	-21.58 (-38.69–-1.10)	-25.03 (-40.98–-5.36)
Belarus	-12.27 (-17.44–-6.79)	-5.17 (-9.14–-0.68)	-7.34 (-33.23–26.44)	-10.61 (-33.91–17.81)
Belgium	-11.64 (-16.73–-6.24)	-9.37 (-12.64–-5.88)	-16.74 (-23.16–-10.48)	-29.15 (-33.98–-24.22)
Belize	-12.59 (-17.72–-7.28)	-9.18 (-12.49–-5.81)	-29.15 (-39.65–-17.23)	-33.35 (-42.78–-22.72)
Benin	-13.45 (-17.76–-8.18)	-9.33 (-12.27–-6.47)	-25.81 (-46.67–5.34)	-23.76 (-43.86–4.70)
Bermuda	-23.01 (-27.20–-18.13)	-13.93 (-16.48–-11.19)	-48.11 (-58.42–-35.10)	-48.16 (-57.57–-37.52)
Bhutan	-27.95 (-33.10–-22.00)	-17.73 (-21.26–-13.52)	-43.00 (-62.50–-3.94)	-43.84 (-61.78–-13.22)
Bolivia (Plurinational State of)	-27.33 (-31.79–-21.37)	-23.63 (-26.31–-21.06)	-38.47 (-58.43–-13.46)	-44.27 (-61.39–-21.62)
Bosnia and Herzegovina	-25.58 (-31.70–-19.78)	-16.71 (-20.29–-13.02)	-57.44 (-73.80–-35.28)	-57.84 (-72.04–-40.96)
Botswana	-12.18 (-17.43–-7.16)	-9.11 (-12.23–-5.61)	-49.07 (-63.63–-22.19)	-43.41 (-59.00–-17.37)
Brazil	-26.93 (-30.74–-22.73)	-25.93 (-28.19–-23.95)	-27.02 (-31.18–-23.19)	-37.51 (-40.33–-34.51)
Brunei Darussalam	-32.29 (-39.98–-18.02)	-21.93 (-26.10–-17.79)	-48.74 (-62.15–-29.54)	-47.92 (-59.94–-30.49)
Bulgaria	2.86 (-2.42–9.02)	-0.79 (-4.21–2.64)	18.05 (1.33–39.49)	-0.56 (-15.13–16.89)
Burkina Faso	-10.15 (-14.91–-4.43)	-7.28 (-10.47–-4.00)	-14.87 (-37.23–25.29)	-14.27 (-35.71–18.61)
Burundi	-16.77 (-21.23–-12.23)	-13.22 (-16.43–-9.84)	-42.07 (-68.87–3.49)	-43.11 (-65.37–-1.04)
Côte d'Ivoire	-12.87 (-17.70–-7.61)	-9.66 (-12.55–-6.43)	-17.79 (-43.68–23.08)	-16.77 (-41.38–19.34)
Cabo Verde	-13.01 (-17.58–-8.37)	-7.42 (-10.10–-3.76)	-23.86 (-49.28–27.42)	-27.96 (-47.73–0.86)
Cambodia	-17.52 (-22.70–-11.41)	-10.63 (-13.89–-7.11)	-39.99 (-60.65–-12.26)	-42.16 (-59.45–-20.28)
Cameroon	-7.41 (-12.11–-2.77)	-5.96 (-9.97–-2.70)	-13.76 (-41.72–27.17)	-10.57 (-37.70–26.60)
Canada	-24.57 (-28.91–-19.33)	-16.86 (-20.05–-13.58)	-40.52 (-46.20–-34.51)	-43.89 (-48.55–-38.98)
Central African Republic	-3.35 (-8.79–2.39)	-1.89 (-5.56–1.86)	-11.31 (-40.69–29.63)	-12.44 (-40.20–27.30)
Chad	-6.41 (-11.14–-0.74)	-5.31 (-8.89–-1.86)	1.89 (-22.42–46.04)	1.89 (-21.72–38.32)
Chile	-16.44 (-21.99–-10.86)	-3.54 (-7.83–4.03)	-25.68 (-32.48–-19.12)	-34.01 (-38.86–-28.82)
China	-56.50 (-60.17–-53.32)	-36.16 (-38.34–-34.17)	-82.71 (-88.71–-67.93)	-81.28 (-87.24–-68.37)
Colombia	-8.86 (-15.89–-1.68)	-12.17 (-15.31–-8.88)	-10.25 (-25.34–5.96)	-20.21 (-33.26–-6.50)
Comoros	-17.38 (-21.68–-13.13)	-12.88 (-15.89–-9.83)	-35.80 (-57.32–-3.25)	-35.00 (-54.39–-5.16)
Congo	-19.17 (-24.18–-13.66)	-11.89 (-15.31–-7.72)	-30.05 (-51.82–4.36)	-29.55 (-49.48–2.74)
Cook Islands	-29.63 (-33.61–-25.61)	-15.99 (-18.43–-13.24)	-65.13 (-79.25–-42.22)	-59.92 (-73.50–-38.86)
Costa Rica	-16.60 (-22.37–-11.15)	-10.33 (-13.20–-7.36)	-3.27 (-15.52–10.86)	-6.83 (-18.00–4.66)
Croatia	-31.54 (-36.92–-26.92)	-19.44 (-22.69–-15.72)	-47.44 (-55.23–-39.16)	-53.28 (-59.71–-46.14)
Cuba	-22.28 (-27.08–-17.51)	-15.37 (-18.38–-12.46)	-35.82 (-45.77–-25.05)	-40.11 (-47.94–-30.89)
Cyprus	-33.47 (-38.26–-28.31)	-23.82 (-26.45–-21.06)	-69.87 (-79.11–-58.05)	-67.10 (-76.59–-56.74)
Czechia	-31.80 (-35.48–-28.17)	-19.97 (-23.03–-17.18)	-59.65 (-64.96–-52.62)	-60.60 (-65.41–-54.61)
Democratic People's Republic of Korea	-21.00 (-25.03–-16.19)	-12.84 (-15.93–-9.59)	-36.78 (-60.38–9.81)	-32.51 (-57.51–15.39)
Democratic Republic of the Congo	-0.62 (-5.74–5.06)	0.26 (-3.21–4.12)	-4.34 (-42.61–50.34)	-8.15 (-39.18–41.99)
Denmark	-30.90 (-36.12–-25.38)	-20.85 (-23.87–-17.83)	-47.92 (-53.11–-43.00)	-56.16 (-60.00–-52.24)
Djibouti	-11.77 (-16.27–-6.87)	-8.00 (-11.18–-4.52)	-26.67 (-50.86–23.18)	-24.92 (-46.99–21.34)
Dominica	-16.28 (-21.46–-11.09)	-12.99 (-15.85–-10.05)	-26.78 (-41.41–-7.33)	-23.72 (-38.64–-3.38)
Dominican Republic	9.90 (2.13–17.98)	3.54 (-0.33–7.30)	-24.74 (-49.99–7.30)	-25.95 (-47.98–-0.40)
Ecuador	-10.86 (-16.71–-4.69)	-10.56 (-13.62–-7.54)	31.91 (4.32–68.99)	4.28 (-15.85–31.34)
Egypt	-26.73 (-31.30–-21.87)	-18.49 (-21.70–-15.44)	-60.64 (-77.46–-30.13)	-64.81 (-77.74–-46.60)
El Salvador	-30.80 (-35.58–-25.90)	-23.34 (-25.67–-20.43)	-40.23 (-61.76–-17.62)	-43.73 (-61.94–-25.76)
Equatorial Guinea	-33.92 (-37.70–-29.33)	-20.18 (-23.13–-16.73)	-64.31 (-82.44–-19.92)	-61.15 (-78.80–-25.43)
Eritrea	-12.52 (-17.95–-5.67)	-6.35 (-10.21–-2.68)	-35.37 (-52.31–-6.13)	-35.16 (-51.49–-7.28)
Estonia	-33.82 (-38.01–-29.56)	-28.74 (-31.33–-25.81)	-36.77 (-47.19–-27.12)	-47.93 (-55.48–-39.57)
Eswatini	-4.85 (-10.80–1.70)	-5.52 (-8.84–-1.80)	-12.35 (-42.71–31.23)	-6.16 (-38.13–36.13)
Ethiopia	-37.44 (-39.87–-34.60)	-31.40 (-33.83–-29.06)	-54.38 (-69.12–-34.07)	-53.50 (-66.53–-37.50)
Fiji	-17.92 (-22.89–-11.46)	-14.35 (-17.54–-11.28)	-22.18 (-41.91–6.59)	-24.60 (-42.76–0.14)
Finland	-24.10 (-31.93–-16.89)	-17.68 (-20.66–-14.87)	-49.03 (-54.30–-43.98)	-52.21 (-56.29–-48.34)
France	-0.27 (-5.92–5.16)	0.93 (-2.68–4.71)	-27.33 (-32.97–-21.96)	-25.25 (-30.28–-20.06)

© 2025 GBD 2021 Global Subarachnoid Hemorrhage Risk Factors Collaborators. *JAMA Neurology*.

Gabon	-17.74 (-22.76–-12.37)	-10.30 (-13.75–-6.51)	-27.13 (-53.12–9.33)	-25.40 (-48.57–8.35)
Gambia	-8.51 (-13.09–-3.26)	-7.12 (-10.27–-3.57)	0.76 (-35.30–42.25)	-0.97 (-33.37–36.80)
Georgia	17.82 (10.03–26.70)	7.21 (2.94–11.43)	49.11 (11.66–105.55)	31.99 (0.83–73.43)
Germany	-26.77 (-31.11–-21.80)	-16.03 (-18.94–-12.89)	-45.38 (-51.50–-38.33)	-49.88 (-54.40–-44.60)
Ghana	-7.51 (-12.39–-1.58)	-6.94 (-9.95–-3.75)	-18.97 (-43.37–31.33)	-21.10 (-42.48–18.17)
Greece	-3.29 (-9.38–3.20)	6.50 (2.26–10.64)	-17.66 (-28.14–-3.24)	-15.21 (-24.27–-0.67)
Greenland	3.23 (-38.72–25.83)	-15.52 (-36.52–-6.01)	-61.01 (-68.93–-50.71)	-62.31 (-69.18–-53.34)
Grenada	-20.41 (-25.49–-15.04)	-13.17 (-16.33–-10.32)	-37.23 (-49.44–-18.63)	-41.11 (-52.66–-24.60)
Guam	-18.00 (-22.43–-12.19)	-3.22 (-6.48–0.36)	-53.40 (-61.78–-39.43)	-31.22 (-40.82–-19.03)
Guatemala	-0.45 (-6.75–6.01)	-2.14 (-5.35–1.52)	26.35 (2.40–52.35)	18.79 (-0.90–40.71)
Guinea	-4.97 (-10.11–0.72)	-3.94 (-7.27–-0.03)	-6.69 (-33.98–37.43)	-7.02 (-31.54–31.69)
Guinea-Bissau	-12.44 (-17.12–-7.34)	-10.41 (-13.54–-6.88)	-17.14 (-43.67–14.26)	-18.72 (-44.51–12.77)
Guyana	-20.58 (-25.33–-15.25)	-12.65 (-15.81–-9.38)	-26.66 (-45.79–-4.34)	-26.30 (-44.55–-4.53)
Haiti	-3.91 (-14.21–8.66)	-5.06 (-8.49–-1.15)	-23.73 (-48.53–12.04)	-27.37 (-49.58–4.11)
Honduras	1.90 (-4.83–8.93)	-12.18 (-15.12–-8.79)	30.76 (-11.41–93.34)	2.99 (-28.46–46.36)
Hungary	-23.30 (-27.45–-19.00)	-18.41 (-21.51–-14.80)	-46.55 (-54.89–-37.97)	-51.37 (-58.13–-44.16)
Iceland	-26.00 (-30.16–-21.27)	-17.82 (-20.65–-14.99)	-48.03 (-53.77–-41.25)	-52.57 (-57.10–-47.45)
India	-25.73 (-28.88–-22.23)	-13.31 (-15.80–-11.02)	-33.94 (-49.76–-5.96)	-34.32 (-48.49–-13.05)
Indonesia	-19.11 (-22.37–-15.60)	-18.28 (-20.74–-16.17)	-24.16 (-43.54–0.59)	-28.73 (-43.47–-8.52)
Iran (Islamic Republic of)	-30.87 (-33.06–-28.67)	-16.98 (-18.83–-15.17)	-61.92 (-78.75–-47.47)	-60.76 (-75.16–-49.06)
Iraq	-40.93 (-44.63–-36.99)	-27.24 (-30.31–-24.23)	-45.42 (-70.78–-18.75)	-50.48 (-68.94–-30.00)
Ireland	-39.10 (-43.70–-33.80)	-30.69 (-33.51–-27.34)	-56.87 (-61.22–-52.61)	-61.37 (-64.36–-58.48)
Israel	-17.49 (-21.72–-13.12)	-12.19 (-15.56–-8.89)	-29.09 (-36.35–-21.05)	-41.14 (-45.91–-35.83)
Italy	-27.98 (-33.26–-21.28)	-17.46 (-19.63–-15.17)	-31.61 (-36.50–-27.84)	-44.39 (-47.19–-41.64)
Jamaica	-11.44 (-18.71–-3.74)	-9.72 (-12.78–-6.06)	-24.72 (-44.55–-1.09)	-33.48 (-50.13–-14.05)
Japan	-5.56 (-10.87–-1.18)	12.11 (8.82–15.62)	-47.08 (-50.14–-44.54)	-40.80 (-43.62–-38.18)
Jordan	-27.16 (-33.03–-22.35)	-15.68 (-18.86–-12.81)	-50.97 (-70.94–-21.28)	-47.84 (-63.67–-28.71)
Kazakhstan	2.62 (-3.10–8.51)	-2.86 (-6.47–0.85)	6.84 (-20.64–38.48)	-7.59 (-29.41–17.21)
Kenya	-12.17 (-14.45–-9.50)	-7.73 (-9.71–-5.75)	-7.90 (-30.38–24.92)	-6.34 (-24.90–17.75)
Kiribati	9.92 (-6.37–20.93)	8.88 (3.98–13.93)	-6.77 (-30.85–29.22)	-8.90 (-31.19–20.49)
Kuwait	-13.70 (-17.62–-9.89)	-9.77 (-12.74–-6.91)	-72.42 (-77.41–-66.87)	-64.16 (-69.35–-58.70)
Kyrgyzstan	-14.84 (-20.08–-7.87)	-13.80 (-17.38–-10.00)	-9.96 (-29.96–11.84)	-15.43 (-34.93–7.53)
Lao People's Democratic Republic	-25.67 (-29.60–-20.73)	-15.17 (-18.79–-11.84)	-55.93 (-70.47–-32.34)	-55.49 (-69.48–-32.70)
Latvia	-30.70 (-34.86–-26.93)	-20.65 (-23.96–-17.17)	-30.08 (-40.28–-19.09)	-37.35 (-46.45–-27.35)
Lebanon	-34.62 (-38.81–-30.72)	-20.58 (-23.31–-17.94)	-74.64 (-84.90–-60.57)	-73.01 (-82.43–-60.16)
Lesotho	12.95 (4.19–21.44)	3.62 (-0.43–8.11)	31.95 (-16.15–112.67)	39.66 (-9.67–110.23)
Liberia	-12.81 (-16.80–-7.95)	-10.70 (-13.85–-7.54)	-7.38 (-50.94–59.48)	-9.74 (-48.09–53.24)
Libya	-24.65 (-30.22–-19.80)	-15.71 (-18.40–-12.64)	-17.29 (-56.41–23.97)	-22.65 (-54.92–9.13)
Lithuania	-21.00 (-25.69–-16.56)	-5.20 (-8.82–-0.51)	-8.97 (-23.99–6.56)	-22.57 (-34.65–-8.69)
Luxembourg	-25.36 (-29.93–-21.03)	-16.34 (-19.57–-13.07)	-54.17 (-59.45–-48.47)	-59.45 (-63.66–-54.98)
Madagascar	-5.01 (-10.07–0.80)	-3.68 (-7.24–-0.20)	-20.36 (-45.67–9.30)	-18.52 (-41.11–11.39)
Malawi	-9.44 (-14.38–-4.44)	-6.66 (-9.73–-2.93)	-8.92 (-35.87–16.00)	-7.10 (-31.54–19.88)
Malaysia	-33.01 (-37.50–-28.97)	-17.13 (-20.36–-13.56)	-41.37 (-62.66–-19.70)	-41.02 (-57.89–-23.84)
Maldives	-41.36 (-45.30–-37.44)	-25.94 (-28.69–-22.97)	-69.08 (-79.94–-55.46)	-69.44 (-77.80–-59.06)
Mali	-14.26 (-18.68–-9.62)	-10.25 (-13.02–-7.18)	-25.13 (-45.21–8.35)	-23.53 (-41.96–4.06)
Malta	-21.26 (-25.20–-16.94)	-14.32 (-17.54–-10.81)	-42.93 (-49.01–-35.17)	-39.64 (-45.37–-32.66)
Marshall Islands	-0.73 (-8.32–8.89)	0.70 (-3.15–4.64)	-25.55 (-43.94–-2.83)	-18.66 (-37.72–6.38)
Mauritania	-19.20 (-22.79–-14.69)	-13.46 (-16.60–-10.61)	-34.47 (-58.47–-1.43)	-34.30 (-55.38–-5.76)
Mauritius	-15.49 (-21.25–-9.93)	-7.68 (-11.60–-3.56)	-30.19 (-36.43–-24.56)	-28.11 (-34.23–-21.78)
Mexico	-0.78 (-4.26–3.39)	-6.06 (-8.19–-3.77)	30.58 (16.60–45.07)	16.02 (3.95–27.81)
Micronesia (Federated States of)	-4.62 (-11.16–4.46)	-3.45 (-6.77–0.10)	-31.68 (-50.70–-5.16)	-29.73 (-48.49–-1.50)
Monaco	-29.06 (-32.98–-24.58)	-15.73 (-18.64–-12.87)	-59.66 (-73.32–-37.56)	-55.69 (-69.61–-34.67)
Mongolia	12.10 (3.92–21.59)	-3.51 (-7.42–0.90)	-2.39 (-40.75–62.29)	-3.73 (-41.61–57.54)
Montenegro	-9.31 (-14.20–-4.70)	-7.64 (-10.95–-4.03)	-8.39 (-38.74–23.35)	-22.82 (-43.02–-2.68)
Morocco	-23.66 (-29.30–-18.32)	-19.29 (-22.25–-15.90)	-34.49 (-59.52–15.57)	-42.97 (-60.84–-12.80)
Mozambique	0.01 (-7.43–7.90)	1.30 (-2.78–5.70)	6.17 (-27.40–55.89)	11.91 (-21.78–54.64)
Myanmar	-31.00 (-35.20–-25.93)	-19.69 (-22.75–-16.29)	-51.15 (-67.77–-26.45)	-51.58 (-66.72–-31.34)
Namibia	-15.60 (-19.94–-10.77)	-12.34 (-15.47–-9.10)	-20.33 (-44.99–11.87)	-19.25 (-40.00–8.83)
Nauru	-6.04 (-12.78–2.43)	1.09 (-2.81–4.96)	-10.82 (-42.12–34.30)	-9.37 (-37.80–32.24)
Nepal	-18.21 (-23.19–-13.03)	-12.57 (-15.82–-8.52)	-38.74 (-60.61–-6.93)	-40.04 (-58.80–-12.43)
Netherlands	-29.75 (-34.24–-25.10)	-20.07 (-23.04–-17.05)	-47.80 (-51.82–-43.64)	-54.04 (-57.17–-51.05)
New Zealand	-37.58 (-42.27–-32.29)	-23.27 (-26.20–-20.31)	-47.84 (-52.18–-43.04)	-51.38 (-55.00–-47.82)
Nicaragua	-19.58 (-24.49–-13.40)	-12.94 (-16.10–-9.99)	-31.77 (-51.99–-11.60)	-34.28 (-50.42–-17.53)
Niger	-8.84 (-13.68–-3.38)	-8.94 (-11.80–-5.76)	-17.67 (-42.81–21.89)	-20.85 (-43.19–11.41)
Nigeria	-25.81 (-27.57–-24.05)	-18.74 (-20.76–-16.74)	-48.60 (-65.31–-22.51)	-43.57 (-61.19–-18.25)
Niue	-25.84 (-30.18–-21.14)	-15.00 (-17.93–-12.12)	-31.14 (-51.55–-5.42)	-14.59 (-38.82–15.52)
North Macedonia	7.54 (0.39–17.15)	-10.07 (-13.43–-6.93)	-19.21 (-43.50–9.90)	-32.69 (-50.01–-12.13)
Northern Mariana Islands	-14.49 (-19.78–-8.68)	-9.68 (-12.61–-6.51)	-30.77 (-49.12–-5.76)	-30.07 (-47.23–-7.33)
Norway	-30.40 (-33.87–-26.18)	-18.89 (-21.05–-16.55)	-60.10 (-62.68–-57.68)	-63.31 (-65.53–-61.06)
Oman	5.54 (-25.65–22.80)	-10.89 (-14.77–-7.26)	-59.66 (-78.99–-33.35)	-59.07 (-74.15–-40.22)
Pakistan	-16.69 (-21.07–-12.25)	-8.81 (-12.41–-5.54)	-14.18 (-35.56–14.15)	-10.04 (-30.96–14.57)

© 2025 GBD 2021 Global Subarachnoid Hemorrhage Risk Factors Collaborators. *JAMA Neurology*.

Palau	-20.30 (-24.65–-14.90)	-8.66 (-12.03–-5.33)	-27.03 (-49.53–1.15)	-24.68 (-46.30–0.32)
Palestine	-18.91 (-23.09–-14.77)	-9.77 (-13.03–-6.40)	-47.83 (-66.24–-22.07)	-46.04 (-61.31–-28.35)
Panama	-11.72 (-17.86–-5.11)	-8.32 (-11.48–-5.43)	1.41 (-22.65–24.61)	-3.02 (-22.98–17.03)
Papua New Guinea	-15.65 (-20.49–-9.84)	-7.78 (-11.11–-4.76)	-31.62 (-52.94–-1.21)	-29.39 (-49.10–0.50)
Paraguay	-14.96 (-22.14–-8.89)	-12.16 (-15.54–-8.79)	-23.92 (-49.18–3.74)	-28.68 (-49.66–-5.14)
Peru	-30.93 (-35.53–-26.59)	-19.73 (-22.54–-17.00)	-39.72 (-63.10–-13.00)	-40.52 (-59.81–-18.64)
Philippines	25.04 (18.20–32.09)	23.26 (19.08–26.86)	7.55 (-19.34–35.70)	11.70 (-9.38–33.88)
Poland	-37.51 (-40.17–-34.52)	-28.91 (-30.94–-26.87)	-49.38 (-54.07–-44.72)	-53.59 (-57.71–-49.47)
Portugal	-31.96 (-42.98–-26.77)	-25.85 (-28.90–-22.81)	-43.43 (-47.82–-39.04)	-50.32 (-53.40–-46.67)
Puerto Rico	-19.84 (-23.72–-15.84)	-13.18 (-16.17–-10.22)	-36.62 (-47.47–-24.14)	-37.46 (-47.09–-26.57)
Qatar	-11.81 (-16.92–-5.81)	-10.88 (-13.64–-8.03)	-66.18 (-81.68–-43.02)	-63.95 (-78.60–-41.13)
Republic of Korea	-46.22 (-50.91–-40.31)	-24.68 (-28.87–-20.27)	-77.90 (-84.03–-70.86)	-75.08 (-81.23–-68.22)
Republic of Moldova	-20.11 (-23.87–-15.93)	-10.61 (-14.19–-6.18)	-27.18 (-38.02–-13.97)	-24.45 (-35.41–-11.14)
Romania	-4.40 (-9.45–0.75)	-10.16 (-13.39–-6.60)	-5.43 (-23.27–14.87)	-13.24 (-29.72–3.14)
Russian Federation	2.38 (-2.49–7.37)	-5.10 (-7.50–-2.94)	16.02 (7.14–25.06)	3.95 (-3.90–11.67)
Rwanda	-31.32 (-35.31–-26.61)	-25.66 (-28.33–-22.89)	-58.76 (-74.04–-38.00)	-59.33 (-72.81–-40.17)
Saint Kitts and Nevis	-29.79 (-34.42–-25.12)	-22.19 (-24.78–-19.33)	-45.36 (-56.53–-29.96)	-49.28 (-59.60–-34.88)
Saint Lucia	-23.92 (-28.84–-18.83)	-14.13 (-17.41–-10.76)	-47.64 (-57.83–-36.40)	-46.82 (-56.64–-35.78)
Saint Vincent and the Grenadines	-22.31 (-26.91–-17.70)	-15.42 (-18.20–-12.38)	-24.92 (-36.10–-13.14)	-24.14 (-35.80–-13.10)
Samoa	-10.75 (-16.54–-4.75)	-2.31 (-5.72–0.99)	-28.90 (-48.62–-5.19)	-24.32 (-43.02–-0.36)
San Marino	-18.05 (-22.10–-13.50)	-9.25 (-12.73–-5.90)	-62.95 (-76.87–-43.99)	-52.88 (-66.62–-37.18)
Sao Tome and Principe	-7.10 (-11.62–-2.56)	-5.91 (-9.31–-2.22)	-3.72 (-33.93–31.26)	-4.09 (-30.97–29.40)
Saudi Arabia	-22.01 (-25.79–-18.03)	-11.13 (-13.92–-8.11)	-39.70 (-70.56–2.28)	-39.70 (-67.27–-6.17)
Senegal	-12.23 (-16.36–-7.85)	-8.67 (-12.09–-5.53)	-15.40 (-40.71–13.71)	-18.64 (-39.15–9.09)
Serbia	-18.21 (-23.28–-12.90)	-25.38 (-28.00–-22.99)	-48.73 (-63.04–-30.61)	-51.82 (-64.85–-35.57)
Seychelles	-27.99 (-31.70–-24.17)	-15.82 (-18.79–-12.58)	-54.62 (-69.12–-42.11)	-51.54 (-62.03–-40.75)
Sierra Leone	-6.54 (-12.16–-1.47)	-5.21 (-8.46–-1.96)	-7.69 (-34.48–29.61)	-8.29 (-33.59–27.93)
Singapore	-33.61 (-38.32–-28.58)	-22.93 (-25.96–-20.27)	-71.28 (-74.37–-68.52)	-65.18 (-68.41–-61.98)
Slovakia	-36.93 (-40.74–-33.30)	-22.89 (-25.33–-19.88)	-48.59 (-59.98–-34.50)	-49.57 (-59.37–-37.58)
Slovenia	-28.71 (-32.58–-24.59)	-18.83 (-21.98–-15.74)	-58.83 (-65.16–-52.17)	-61.71 (-66.97–-56.15)
Solomon Islands	20.85 (-0.91–41.09)	6.31 (2.02–10.76)	-19.41 (-43.63–25.25)	-15.97 (-41.51–33.68)
Somalia	-2.93 (-7.85–2.72)	-2.73 (-6.24–1.30)	-28.01 (-56.87–9.53)	-26.24 (-51.13–9.93)
South Africa	-16.78 (-19.51–-13.49)	-15.55 (-17.72–-13.24)	8.23 (-8.46–25.40)	-7.70 (-18.49–3.73)
South Sudan	-11.15 (-15.84–-5.73)	-8.68 (-11.69–-5.33)	-19.20 (-52.63–44.30)	-18.25 (-49.95–37.36)
Spain	-7.84 (-13.41–-1.93)	-1.89 (-6.00–2.65)	-8.01 (-15.60–-0.42)	-26.30 (-30.95–-21.59)
Sri Lanka	-28.43 (-32.77–-23.59)	-16.60 (-19.31–-13.87)	-45.17 (-66.32–-15.81)	-41.25 (-57.69–-20.01)
Sudan	-31.35 (-35.62–-26.93)	-23.29 (-25.94–-20.45)	-49.47 (-68.91–-14.46)	-51.76 (-67.91–-26.28)
Suriname	-10.89 (-16.05–-5.08)	-10.48 (-13.64–-7.19)	-24.05 (-44.79–1.68)	-24.69 (-43.59–-3.07)
Sweden	-29.75 (-33.87–-25.18)	-19.95 (-23.04–-16.89)	-58.90 (-63.40–-54.00)	-61.42 (-65.41–-57.53)
Switzerland	-25.94 (-30.54–-20.30)	-9.18 (-12.65–-5.38)	-50.41 (-55.47–-45.82)	-56.65 (-60.34–-52.59)
Syrian Arab Republic	-22.96 (-36.59–-15.82)	-16.47 (-19.42–-13.52)	-44.30 (-74.80–-1.35)	-51.37 (-72.69–-20.41)
Taiwan (Province of China)	-39.16 (-44.08–-33.46)	-28.84 (-31.47–-26.08)	-60.71 (-77.31–-40.68)	-66.49 (-79.10–-52.18)
Tajikistan	-13.71 (-23.60–-4.44)	-3.54 (-6.97–-0.55)	-35.63 (-41.69–-30.19)	-29.91 (-35.42–-24.72)
Thailand	13.92 (7.40–21.25)	-3.66 (-7.89–-0.03)	-18.68 (-46.64–24.40)	-22.64 (-44.98–14.32)
Timor-Leste	-33.71 (-38.10–-28.18)	-18.47 (-21.77–-15.06)	-49.69 (-65.87–-30.47)	-42.32 (-58.52–-23.24)
Togo	-11.39 (-16.38–-6.42)	-4.68 (-8.11–-1.14)	-22.39 (-49.24–11.41)	-22.09 (-46.00–8.32)
Tokelau	-9.55 (-13.93–-4.80)	-7.82 (-10.76–-4.31)	-5.51 (-35.64–37.16)	-8.34 (-35.41–30.48)
Tonga	-25.38 (-30.20–-18.79)	-14.28 (-17.35–-11.48)	-43.82 (-65.55–-17.82)	-29.38 (-52.81–1.46)
Trinidad and Tobago	-18.01 (-22.29–-13.16)	-9.69 (-12.79–-6.71)	-25.58 (-48.04–3.77)	-23.16 (-43.06–2.53)
Tunisia	-18.99 (-23.61–-13.94)	-15.54 (-18.30–-12.36)	-26.01 (-43.51–-2.75)	-24.04 (-40.71–-1.76)
Turkey	-26.75 (-31.13–-21.57)	-15.30 (-18.20–-12.17)	-46.34 (-72.20–-1.21)	-48.28 (-69.26–-16.11)
Turkmenistan	25.23 (17.77–33.53)	18.61 (13.22–23.82)	49.84 (13.21–99.34)	41.70 (8.98–85.43)
Tuvalu	-16.28 (-21.80–-9.09)	-9.51 (-12.57–-5.91)	-44.76 (-60.46–-27.05)	-42.78 (-56.93–-25.18)
Uganda	-13.66 (-18.91–-8.14)	-9.53 (-12.93–-6.27)	-40.09 (-56.44–-1.30)	-34.96 (-52.05–-4.55)
Ukraine	-8.64 (-14.31–-2.77)	3.17 (-0.74–7.78)	-29.52 (-48.85–-8.25)	-17.86 (-39.75–6.40)
United Arab Emirates	-25.23 (-29.63–-20.95)	-17.63 (-20.31–-14.71)	-42.51 (-73.04–-9.46)	-50.54 (-72.90–-24.22)
United Kingdom	-28.00 (-30.62–-24.80)	-20.03 (-21.61–-18.44)	-47.20 (-49.98–-45.28)	-54.27 (-55.75–-52.84)
United Republic of Tanzania	-14.85 (-19.88–-8.70)	-10.26 (-13.64–-6.51)	-36.74 (-58.12–-2.41)	-34.29 (-53.05–-7.50)
United States of America	-8.99 (-12.73–-3.86)	6.03 (1.06–11.19)	-13.94 (-18.27–-10.31)	-23.92 (-27.05–-21.07)
United States Virgin Islands	-19.26 (-23.92–-14.01)	-15.04 (-17.92–-11.85)	-57.75 (-70.38–-41.00)	-54.01 (-66.72–-37.23)
Uruguay	-25.13 (-30.80–-19.12)	-19.48 (-23.04–-14.19)	-39.18 (-44.61–-32.39)	-45.56 (-50.01–-40.36)
Uzbekistan	2.99 (-6.21–13.16)	-0.68 (-5.12–4.42)	111.13 (60.68–170.21)	56.77 (25.93–95.94)
Vanuatu	2.16 (-3.93–8.52)	4.34 (0.70–8.12)	-22.86 (-43.92–3.87)	-18.52 (-40.82–9.64)
Venezuela (Bolivarian Republic of)	-11.20 (-16.52–-5.17)	-7.93 (-11.25–-4.52)	5.92 (-21.91–38.47)	-2.40 (-27.36–26.28)
Viet Nam	-19.27 (-24.94–-13.85)	-13.19 (-16.36–-9.92)	-39.73 (-64.02–-7.86)	-38.60 (-59.57–-10.52)
Yemen	-17.25 (-22.55–-11.39)	-14.85 (-17.63–-11.81)	-29.73 (-55.32–3.84)	-35.17 (-56.12–-6.23)
Zambia	-6.92 (-12.40–-0.87)	-6.71 (-10.00–-2.97)	-22.19 (-50.23–21.55)	-19.18 (-45.98–15.30)
Zimbabwe	23.85 (15.63–33.69)	17.06 (12.36–22.17)	51.55 (11.75–102.44)	64.56 (20.18–119.06)

DALY = disability-adjusted life years

© 2025 GBD 2021 Global Subarachnoid Hemorrhage Risk Factors Collaborators. *JAMA Neurology*.

Supplementary Table 17. Rankings of subarachnoid hemorrhage (SAH) by the number of global deaths and disability-adjusted life years (DALY) in 1990 and 2021 among all diseases, non-communicable diseases, cardiovascular diseases, neurological disorders, and strokes.

	All diseases	Non-communicable diseases	Cardiovascular diseases	Neurological disorders including strokes	Strokes
Deaths, ranking (% of all) in 1990					
Overall	27 (0.81)	13 (1.40)	6 (3.04)	4 (6.25)	3 (7.45)
Men	32 (0.73)	17 (1.29)	6 (2.97)	4 (6.56)	3 (7.56)
Women	24 (0.91)	15 (1.52)	6 (3.10)	4 (5.99)	3 (7.35)
Deaths, ranking (% of all) in 2021					
Overall	36 (0.52)	23 (0.81)	6 (1.82)	5 (3.58)	3 (4.86)
Men	37 (0.46)	22 (0.74)	6 (1.70)	5 (3.64)	3 (4.60)
Women	31 (0.59)	20 (0.88)	7 (1.95)	4 (3.52)	3 (5.15)
Deaths, decrease in ranking as units (decrease in proportional burden as %) between 1990 and 2021					
Overall	9 (35.80)	10 (42.14)	0 (40.13)	1 (42.72)	0 (34.77)
Men	5 (36.99)	5 (42.64)	0 (42.76)	1 (44.51)	0 (39.15)
Women	7 (35.16)	5 (42.11)	1 (37.10)	0 (41.24)	0 (29.93)
DALY, ranking (% of all with 95% UI) in 1990					
Overall	44 (0.47)	23 (1.05)	6 (4.04)	5 (6.59)	3 (9.91)
Men	51 (0.44)	24 (1.04)	6 (3.81)	5 (6.98)	3 (9.84)
Women	47 (0.49)	25 (1.06)	6 (4.31)	5 (6.24)	3 (9.99)
DALY, ranking (% of all with 95% UI) in 2021					
Overall	59 (0.37)	39 (0.62)	6 (2.48)	6 (3.91)	3 (6.63)
Men	60 (0.35)	35 (0.62)	6 (2.25)	6 (4.07)	3 (6.21)
Women	54 (0.39)	35 (0.61)	6 (2.80)	6 (3.74)	3 (7.15)
DALYs, decrease in ranking as units (decrease in proportional burden as %) between 1990 and 2021					
Overall	15 (21.28)	16 (40.95)	0 (38.61)	1 (40.67)	0 (33.10)
Men	9 (20.45)	11 (40.38)	0 (40.94)	1 (41.69)	0 (36.89)
Women	7 (20.41)	10 (42.45)	0 (35.03)	1 (40.06)	0 (28.43)

DALY = disability-adjusted life years; UI = uncertainty interval

© 2025 GBD 2021 Global Subarachnoid Hemorrhage Risk Factors Collaborators. *JAMA Neurology*.

Supplementary Table 18. Regional rankings and proportions of subarachnoid hemorrhage-related deaths and disability-adjusted life years (DALY) in 2021 among all 300 diseases/injuries by five country-specific sociodemographic index (SDI) levels, seven GBD super-regions and 21 GBD regions.

Colors illustrate the levels of ranking gradually.

	Death ranking	% (95% UI) of all deaths	DALY ranking	% (95% UI) of all DALYs
Global	36	0.52 (0.45–0.59)	59	0.37 (0.32–0.43)
Low SDI	65	0.23 (0.13–0.46)	102	0.15 (0.09–0.28)
Low-middle SDI	40	0.46 (0.36–0.58)	58	0.34 (0.27–0.43)
Middle SDI	26	0.68 (0.56–0.77)	47	0.49 (0.41–0.56)
High-middle SDI	27	0.52 (0.46–0.61)	49	0.42 (0.37–0.49)
High SDI	37	0.57 (0.50–0.60)	51	0.45 (0.40–0.49)
Central Europe, Eastern Europe, and Central Asia	24	0.49 (0.46–0.51)	42	0.43 (0.39–0.46)
High-income	37	0.59 (0.52–0.63)	50	0.47 (0.42–0.51)
Latin America and Caribbean	28	0.65 (0.61–0.68)	49	0.51 (0.46–0.56)
North Africa and Middle East	42	0.30 (0.25–0.39)	74	0.22 (0.18–0.28)
South Asia	34	0.46 (0.32–0.62)	58	0.34 (0.25–0.45)
Southeast Asia, East Asia, and Oceania	24	0.75 (0.61–0.89)	46	0.56 (0.46–0.67)
Sub-Saharan Africa	81	0.15 (0.08–0.36)	123	0.10 (0.06–0.23)
Andean Latin America	25	0.64 (0.55–0.73)	40	0.57 (0.48–0.66)
Australasia	38	0.63 (0.55–0.68)	55	0.41 (0.36–0.47)
Caribbean	33	0.53 (0.42–0.65)	49	0.48 (0.38–0.58)
Central Asia	24	0.55 (0.51–0.60)	54	0.40 (0.36–0.44)
Central Europe	32	0.40 (0.36–0.43)	51	0.38 (0.34–0.41)
Central Latin America	28	0.57 (0.53–0.62)	52	0.45 (0.40–0.49)
Central Sub-Saharan Africa	70	0.17 (0.08–0.45)	113	0.11 (0.06–0.28)
East Asia	22	0.79 (0.59–0.94)	37	0.57 (0.44–0.70)
Eastern Europe	23	0.52 (0.49–0.56)	36	0.46 (0.42–0.50)
Eastern Sub-Saharan Africa	77	0.17 (0.07–0.48)	121	0.12 (0.05–0.30)
High-income Asia Pacific	26	0.99 (0.83–1.09)	24	0.91 (0.81–1.01)
High-income North America	38	0.52 (0.47–0.55)	51	0.38 (0.35–0.42)
Oceania	27	0.65 (0.46–0.90)	42	0.52 (0.40–0.67)
Southeast Asia	27	0.68 (0.56–0.92)	40	0.55 (0.46–0.69)
Southern Latin America	32	0.64 (0.59–0.67)	45	0.53 (0.48–0.58)
Southern Sub-Saharan Africa	77	0.12 (0.10–0.15)	107	0.10 (0.08–0.12)
Tropical Latin America	24	0.76 (0.71–0.79)	38	0.57 (0.52–0.63)
Western Europe	41	0.48 (0.42–0.52)	58	0.37 (0.33–0.41)
Western Sub-Saharan Africa	84	0.13 (0.07–0.30)	123	0.09 (0.05–0.18)

DALY = disability-adjusted life years; SDI = sociodemographic index; UI = uncertainty interval

© 2025 GBD 2021 Global Subarachnoid Hemorrhage Risk Factors Collaborators. *JAMA Neurology*.
