# Supplementary material for: Global, Regional, and National Burden of Nontraumatic Subarachnoid Hemorrhage: The Global Burden of Disease Study 2021
Source: JAMA Neurol. 2025 May 23;82(8):765–87. doi: 10.1001/jamaneurol.2025.1522 (PMC12557468; doi:10.1001/jamaneurol.2025.1522)
Supplement: Supplement 2. — Data Sharing Statement. [file jamaneurol-e251522-s002.pdf]

Data Sharing Statement

Rautalin. Global, Regional, and National Burden of Nontraumatic Subarachnoid Hemorrhage. *JAMA Neurol*. Published May 23, 2025. doi:10.1001/jamaneurol.2025.1522

Data
Data available: Yes
Data types: Data (not involving human participants), Data dictionary
How to access data: https://ghdx.healthdata.org/gbd-2021/sources
When available: beginning date: 01-01-2024

Supporting Documents
Document types: None

Additional Information
Who can access the data: The exact data sources and results are publicly available through the website of the Institute for Health Metrics and Evaluation
Types of analyses: Data made available for download on IHME Websites can be used, shared, modified or built upon by non-commercial users in accordance with the IHME FREE-OF-CHARGE NON-COMMERCIAL USER AGREEMENT. For more information (and inquiries about commercial use), visit IHME Terms and Conditions.
Mechanisms of data availability: Currently available for public
